# Supplementary material for: Avoiding ‘second victims’ in healthcare: what support do staff want for coping with patient safety incidents, what do they get and is it effective? A systematic review
Source: BMJ Open. 2025 Feb 10;15(2):e087512. doi: 10.1136/bmjopen-2024-087512 (PMC12185930; doi:10.1136/bmjopen-2024-087512)
Supplement: Supplementary data [file bmjopen-15-2-s002.pdf]

BMJ Publishing Group Limited (BMJ) disclaims all liability and responsibility arising from any reliance placed on this supplemental material which has been supplied by the author(s)


## Supplementary File 2: Search criteria

### Medline

1 ((critical or patient safety or harmful or clinical) adj incident*).mp.
2 ((avoidable or preventable or unsafe or undesirable) adj1 (event* or outcome* or complication* or death or accident* or injur*)).mp.
3 (serious incident? or critical incident? or never event? or near miss* or sentinel event? or adverse patient occurrence? or serious reportable event? or (serious adj2 event? adj3 (clinical or critical or medical or surgical)) or preventable adverse event? or avoidable adverse event? or ((Medical or surgical) adj2 Adverse adj2 Event?)).mp.
4 1 or 2 or 3 or exp medical errors/
5 ((Health adj2 professional*) or (health adj2 provider*)).mp. or exp health personnel/ or healthcare profession*.mp. or (healthcare adj2 provider*).mp. or healthcare worker*.mp. or health care worker*.mp. or (worker* adj2 (health or healthcare)).mp. or nurs*.ti,ab. or physician*.ti,ab. or surgeon.ti,ab. or midwive*.ti,ab. or clinician*.ti,ab.
6 (4 and 5) or second victim*.af.
7 exp social support/
8 exp Occupational Health Services/
9 Crisis intervention/
10 Counseling/
11 Stress, Psychological/pc, th or Stress Disorders, Post-Traumatic/pc, th
12 support*.ti,kw,kf.
13 ("critical incident stress management" or "critical incident stress reporting system*" or "critical incident stress debrief*").mp.
14 ("Second Victim Experience and Support Tool" or SVEST).mp.
15 (peer group* or (peer* adj3 support*)).af.
16 Debrief*.mp.
17 (support* adj2 (second victim* or organi?ation* or service* or system* or program* or resource* or social)).mp.
18 ((Resilienc* adj2 training) or (resilienc* adj2 intervention*) or (promot* adj2 resilienc*)).mp.
19 "Schwartz Center Rounds".ti,ab,kf,kw.
20 Resilience, psychological/
21 ("Intervention training" or (crisis adj2 training)).mp.
22 "Resilience In Stressful Events".mp.
25 ((Institution* adj2 support) or (Institution* adj2 response*) or (Institution* adj2 respond*)).mp.
26 Simulation Training/
27 (safe* adj2 culture*).mp.
28 psychologic* safe*.mp.
29 just culture*.mp.
30 non-punitive.mp.
31 *organizational culture/
32 organizational culture/ and (px or ed or pc or mt or og).fs.
23 6 and 7
24 6 and 8
33 6 and 9
34 6 and 10
35 6 and 11
36 6 and 12
37 6 and 13
38 6 and 14
39 6 and 15
40 6 and 16
41 6 and 17

| | |
|---|---|
| 44 | 6 and 18 |
| 45 | 6 and 19 |
| 46 | 6 and 20 |
| 47 | 6 and 21 |
| 48 | 6 and 22 |
| 49 | 6 and 23 |
| 50 | 6 and 24 |
| 51 | 6 and 25 |
| 52 | 6 and 26 |
| 53 | 6 and 27 |
| 54 | 6 and 28 |
| 55 | 6 and 29 |
| 56 | 6 and 30 |
| 57 | 6 and 31 |
| 58 | 6 and 32 |
| 59 | or/33-58 |
| 60 | limit 59 to (abstracts and english language and yr="2010 -Current") |



Simms-Ellis R, *et al. BMJ Open* 2025; 15:e087512. doi: 10.1136/bmjopen-2024-087512

## PubMed

| Search | Query |
|---|---|
| #62 | Search: **#45 OR #46 OR #47 OR #48 OR #49 OR #50 OR #51 OR #52 OR #53 OR #54 OR #55 OR #56 OR #57** Filters: **from 2021 - 2022** |
| #61 | Search: **#34 OR #35 OR #36 OR #37 OR #38 OR #39 OR #40 OR #41 OR #42 OR #43 OR #44** Filters: **from 2021 - 2022** |
| #57 | Search: **#6 AND #33** |
| #56 | Search: **#6 AND #32** |
| #55 | Search: **#6 AND #31** |
| #54 | Search: **#6 AND #30** |
| #53 | Search: **#6 AND #29** |
| #52 | Search: **#6 AND #28** |
| #51 | Search: **#6 AND #27** |
| #50 | Search: **#6 AND #26** |
| #49 | Search: **#6 AND #25** |
| #48 | Search: **#6 AND #23** |
| #47 | Search: **#6 AND #22** |
| #46 | Search: **#6 AND #21** |
| #45 | Search: **#6 AND #20** |
| #44 | Search: **#6 AND #19** |
| #43 | Search: **#6 AND #18** |
| #42 | Search: **#6 AND #17** |
| #41 | Search: **#6 AND #16** |
| #40 | Search: **#6 AND #15** |
| #39 | Search: **#6 AND #14** |
| #38 | Search: **#6 AND #13** |

| Search | Query |
|---|---|
| #37 | Search: **#6 AND #12** |
| #36 | Search: **#6 AND #11** |
| #35 | Search: **#6 AND #10** |
| #34 | Search: **#6 AND #9** |
| #33 | Search: **"organizational culture"[Mesh] AND (px[sh] OR ed[sh] OR pc[sh] OR mt[sh] OR og[sh])** |
| #32 | Search: **organizational culture[MeSH Major Topic]** |
| #31 | Search: **"non-punitive"[tw] or "non punitive"[tw]** |
| #30 | Search: **"just culture"[tw] or "just cultures"[tw]** |
| #29 | Search: **"psychological safe"[tw] or "psychological safety"[tw]** |
| #28 | Search: **"safe culture"[tw] or "safety culture"[tw]** |
| #27 | Search: **simulation training[mesh]** |
| #26 | Search: **"Institution support"[all] OR "institutional supports"[all] OR "Institution response"[all] OR [all] OR "institutional responses"[all] OR "institutional response"[all] OR "institution responding"[all] OR "institutional responding"[all] OR "institutions responding"[all] OR "institution responding"[all]** |
| #25 | Search: **#6 and #8** |
| #23 | Search: **"Resilience In Stressful Events"[tw] - Schema: all** |
| #22 | Search: **#6 and #7** |
| #21 | Search: **("debrief"[tw] OR "debriefs"[tw] OR "debriefings"[tw])** |
| #20 | Search: **("Intervention training"[tw] OR ("crisis training"[tw])** |
| #19 | Search: **resilience, psychological[MESH]** |
| #18 | Search: **("Schwartz Center Rounds"[tiab] OR "Schwartz Center Rounds"[ot] OR "Schwartz Center Rounds"[ot])** |
| #17 | Search: **(Resilience[tw] AND training"[tw] OR training[tw] AND resiliency[tw] OR resilience[tw] AND intervention[tw] OR resiliency[tw] AND intervention[tw] OR resilience[tw] AND interventions[tw] OR resiliency[tw] AND interventions[tw] OR promotion[tw] AND resilience[tw] OR promotion"[tw] AND "resiliency"[tw] OR "promoting"[tw] AND "resilience"[tw] OR "promoting"[tw] AND "resiliency"[tw])** |

| Search | Query |
|---|---|
| #16 | Search: **(("support second victim"[tw] OR "support second victims"[tw] OR "support organisation"[tw] OR "support organisations"[tw] OR "support organization"[tw] OR "support organizations"[tw] OR "support service"[tw] OR "support services"[tw] OR "support system"[tw] OR "support systems "[tw] OR "support program"[tw] OR "support programs"[tw] OR "support programme"[tw] OR "support programmes"[tw] OR "support resource "[tw] OR "support resources"[tw] OR "social support"[tw]))** |
| #15 | Search: **("peer group"[all] OR "peer groups"[all] OR "peer support"[all] OR "support peers"[all]))** |
| #14 | Search: **("Second Victim Experience AND Support Tool"[tw] OR "SVEST"[tw])** |
| #13 | Search: **"critical incident stress management"[all] OR "critical incident stress reporting system"[all] OR "critical incident stress reporting systems"[all] OR "critical incident stress debrief"[all] OR "critical incident stress debriefs"[all] OR "critical incident stress debriefing"[all] OR "critical incident stress debriefings"[all]** |
| #12 | Search: **"support"[ti]** |
| #11 | Search: **("Stress, Psychological/Prevention and Control"[mesh] OR "Stress, Psychological/Therapy"[mesh]) OR ("Stress Disorders, Post-Traumatic/Prevention and Control"[mesh] OR "Stress Disorders, Post-Traumatic/Therapy"[mesh])** |
| #10 | Search: **"counseling"[mesh]** |
| #9 | Search: **"crisis intervention"[mesh]** |
| #8 | Search: **"occupational health services"[mesh]** |
| #7 | Search: **"social support"[mesh]** |
| #6 | Search: **(#4 AND #5) or "second victim" or "second victims"** |
| #5 | Search: **("Health professional"[tw] OR "Health professionals"[tw] OR "healthcare professional"[tw] OR "healthcare professionals"[tw] OR "health provider"[tw] OR "healthcare provider"[tw] OR "health provider"[tw] OR "health providers"[tw] OR "healthcare provider"[tw] OR "healthcare providers"[tw] OR "health personnel"[MESH] or "health personnel"[tw] OR "healthcare profession"[tw] OR "healthcare professions"[tw] OR "healthcare worker."[tw] OR "healthcare workers"[tw] OR "health care worker"[tw] OR "health care workers"[tw] OR "health worker"[tw] OR "health workers"[tw] OR nurse[tw] OR nurses[tw] OR physician[tw] OR physicians[tw] OR surgeon[tw] OR surgeons[tw] OR midwives[tw] OR midwife[tw] OR clinician[tw] OR clinicians[tw])** |
| #4 | Search: **#1 or #2 or #3 or "medical errors"[MESH]** |
| #3 | Search: **("serious incident"[tw] OR "serious incidents"[tw] OR "critical incident"[tw] OR "critical incidents"[tw] OR "never event"[tw] OR "never events"[tw] OR "near miss"[tw] OR "near misses"[tw] OR "sentinel event"[tw] OR** |

| Search | Query |
|---|---|
| | **"sentinel events"[tw] OR "adverse patient occurrence"[tw] OR "adverse patient occurrences"[tw] OR "serious reportable event"[tw] OR "serious reportable events"[tw] OR (("serious clinical event"[tw] OR "serious clinical events"[tw] OR "serious critical event"[tw] OR "serious critical events"[tw] OR "serious medical event"[tw] OR "serious medical events"[tw] OR "serious surgical event"[tw] OR "serious surgical events"[tw] OR "preventable adverse event"[tw] OR "preventable adverse events"[tw] OR "avoidable adverse event"[tw] OR "avoidable adverse events "[tw] OR "medical adverse event"[tw] OR "medical adverse events"[tw] OR "surgical adverse event"[tw] OR "surgical adverse events"[tw])** |
| #2 | Search: **("avoidable event"[tw] OR " avoidable events"[tw] OR "avoidable outcome"[tw] OR "avoidable outcomes"[tw] OR "avoidable complication"[tw] OR "avoidable complications"[tw] OR " avoidable death"[tw] OR "avoidable accident"[tw] OR "avoidable accidents"[tw] OR "avoidable injury"[tw] OR "avoidable injuries "[tw] OR "preventable event"[tw] OR " preventable events"[tw] OR "preventable outcome"[tw] OR "preventable outcomes"[tw] OR "preventable complication"[tw] OR "preventable complications"[tw] OR " preventable death"[tw] OR "preventable accident"[tw] OR "preventable accidents"[tw] OR "preventable injury"[tw] OR "preventable injuries"[tw] OR "unsafe event"[tw] OR " unsafe events"[tw] OR "unsafe outcome"[tw] OR "unsafe outcomes"[tw] OR "unsafe complication"[tw] OR "unsafe complications"[tw] OR " unsafe death"[tw] OR "unsafe accident"[tw] OR "unsafe accidents"[tw] OR "unsafe injury"[tw] OR "unsafe injuries"[tw] OR "undesireable event"[tw] OR " undesireable events"[tw] OR "undesireable outcome"[tw] OR "undesireable outcomes"[tw] OR "undesireable complication"[tw] OR "undesireable complications"[tw] OR " undesireable death"[tw] OR "undesireable accident"[tw] OR "undesireable accidents"[tw] OR "undesireable injury"[tw] OR "undesireable injuries"[tw])** |
| #1 | Search: **(("critical incident"[tw] OR "critical incidents"[tw] OR "patient safety incident"[tw] OR "patient safety incidents"[tw] OR "harmful incident"[tw] OR "harmful incidents"[tw] OR "clinical incident"[tw] OR "clinical incidents"[tw]))** |

## Scopus

( ( ALL ( "second victim*" ) ) OR ( ( ( ( TITLE-ABS-KEY ( ( ( critical OR "patient safety" OR harmful OR clinical ) W/1 incident* ) ) ) OR ( TITLE-ABS-KEY ( ( ( avoidable OR preventable OR unsafe OR undesirable ) W/1 ( event* OR out come* OR complication* OR death OR accident* OR injur* ) ) ) ) OR ( TITLE-ABS-KEY ( ( "serious incident*" OR "critical incident*" OR "never event*" OR "near miss*" OR "sentinel event*" OR "adverse patient occurrence*" OR "serious reportable event*" OR ( serious W/2 event* W/3 ( clinical OR critical OR medical OR surgical ) ) OR "preventable adverse event*" OR "avoidable adverse event*" OR ( ( medical OR surgical ) W/2 adverse W/2 event* ) ) ) ) ) OR ( INDEXTE RMS ( "medical errors" ) ) ) AND ( TITLE-ABS-KEY ( ( health W/2 professional* ) OR ( health W/2 provider* ) ) OR INDEXTERMS ( "health personnel" ) OR TITLE-ABS-KEY ( "healthcare profession*" ) OR TITLE-ABS-KEY ( healthcare W/2 provider* ) OR TITLE-ABS-KEY ( "healthcare worker*" ) OR TITLE-ABS-KEY ( "health care worker*" ) OR TITLE-ABS-KEY ( worker* W/2 ( health OR healthcare ) ) OR TITLE-ABS ( nurs* ) OR TITLE-ABS ( physician* ) OR TITLE-ABS ( surgeon ) OR TITLE-ABS ( midwive* ) OR TITLE-ABS ( "clinician*" ) ) ) ) AND ( ( INDEXTERMS ( "Crisis intervention" ) ) OR ( INDEXTERMS ( "counseling" OR "counselling" ) ) OR ( INDEXT ERMS ( "Stress Disorders, Post-Traumatic" ) ) OR ( INDEXTERMS ( "Stress, psychological" ) ) OR ( TITLE ( "support*" ) ) OR ( TITLE-ABS-KEY ( "critical incident stress management" OR "critical incident stress reporting system*" OR "critical incident stress debrief*" ) ) OR ( TITLE-ABS-KEY ( "Second Victim Experience and Support Tool" OR svest ) ) OR ( ALL ( "peer group*" OR ( peer* W/3 support* ) ) ) OR ( TITLE-ABS-KEY ( "Debrief*" ) ) OR ( TITLE-ABS-KEY ( support* W/2 ( "second victim*" OR organi*ation* OR service* OR system* OR program* OR resource* OR social ) ) ) OR ( TITLE-ABS-KEY ( ( resilienc* W/2 training ) OR ( resilienc* W/2 intervention* ) OR ( promot* W/ 2 resilienc* ) ) ) OR ( TITLE-ABS-KEY ( "Schwartz Center Rounds" ) ) OR ( INDEXTERMS ( "Resilience, psychological" ) ) OR ( TITLE-ABS-KEY ( "Intervention training" OR ( crisis W/2 training ) ) ) OR ( ( ( ALL ( "second victim*" ) ) OR ( ( ( ( TITLE-ABS-KEY ( ( ( critical OR "patient safety" OR harmful OR clinical ) W/1 incident* ) ) ) OR ( TITLE-ABS-KEY ( ( ( avoidable OR preventable OR unsafe OR undesirable ) W/1 ( event* OR out come* OR complication* OR death OR accident* OR injur* ) ) ) ) OR ( TITLE-ABS-KEY ( ( "serious incident*" OR "critical incident*" OR "never event*" OR "near miss*" OR "sentinel event*" OR "adverse patient occurrence*" OR "serious reportable event*" OR ( serious W/2 event* W/3 ( clinical OR critical OR medical OR surgical ) ) OR "preventable adverse event*" OR "avoidable adverse event*" OR ( ( medical OR surgical ) W/2 adverse W/2 event* ) ) ) ) ) OR ( INDEXTE RMS ( "medical errors" ) ) ) AND ( TITLE-ABS-KEY ( ( health W/2 professional* ) OR ( health W/2 provider* ) ) OR INDEXTERMS ( "health personnel" ) OR TITLE-ABS-KEY ( "healthcare profession*" ) OR TITLE-ABS-KEY ( healthcare W/2 provider* ) OR TITLE-ABS-KEY ( "healthcare

worker*" ) OR TITLE-ABS-KEY ( "health care worker*" ) OR TITLE-ABS-KEY ( worker* W/2 ( health OR healthcare ) ) OR TITLE-ABS ( nurs* ) OR TITLE-ABS ( physician* ) OR TITLE-ABS ( surgeon ) OR TITLE-ABS ( midwive* ) OR TITLE-ABS ( "clinician*" ) ) ) ) AND ( INDEXTERMS ( "social support" ) ) ) OR ( TITLE-ABS-KEY ( "Resilience In Stressful Events" ) ) OR ( ( ( ALL ( "second victim*" ) ) OR ( ( ( ( TITLE-ABS-KEY ( ( ( critical OR "patient safety" OR harmful OR clinical ) W/1 incident* ) ) ) OR ( TITLE-ABS-KEY ( ( ( avoidable OR preventable OR unsafe OR undesirable ) W/1 ( event* OR out come* OR complication* OR death OR accident* OR injur* ) ) ) ) OR ( TITLE-ABS-KEY ( ( "serious incident*" OR "critical incident*" OR "never event*" OR "near miss*" OR "sentinel event*" OR "adverse patient occurrence*" OR "serious reportable event*" OR ( serious W/2 event* W/3 ( clinical OR critical OR medical OR surgical ) ) OR "preventable adverse event*" OR "avoidable adverse event*" OR ( ( medical OR surgical ) W/2 adverse W/2 event* ) ) ) ) ) OR ( INDEXTERMS ( "medical errors" ) ) ) AND ( TITLE-ABS-KEY ( ( health W/2 professional* ) OR ( health W/2 provider* ) ) OR INDEXTERMS ( "health personnel" ) OR TITLE-ABS-KEY ( "healthcare profession*" ) OR TITLE-ABS-KEY ( healthcare W/2 provider* ) OR TITLE-ABS-KEY ( "healthcare worker*" ) OR TITLE-ABS-KEY ( "health care worker*" ) OR TITLE-ABS-KEY ( worker* W/2 ( health OR healthcare ) ) OR TITLE-ABS ( nurs* ) OR TITLE-ABS ( physician* ) OR TITLE-ABS ( surgeon ) OR TITLE-ABS ( midwive* ) OR TITLE-ABS ( "clinician*" ) ) ) ) AND ( INDEXTERMS ( "Occupational Health Services" ) ) ) OR ( INDEXTERMS ( "Simulation Training" ) ) OR ( TITLE-ABS-KEY ( safe* W/2 culture* ) ) OR ( TITLE-ABS-KEY ( "psychologic* safe*" ) ) OR ( TITLE-ABS-KEY ( "just culture*" ) ) OR ( TITLE-ABS-KEY ( ( institution* W/2 support ) OR ( institution* W/2 response* ) OR ( institution* W/2 respond* ) ) ) OR ( TITLE-ABS-KEY ( "non-punitive" ) ) OR ( INDEXTERMS ( "organizational culture" ) ) ) ) AND ( LIMIT-TO ( PUBYEAR , 2022 ) OR LIMIT-TO ( PUBYEAR , 2021 ) ) AND ( LIMIT-TO ( LANGUAGE , "English" ) ) View less

## Web of Science

# Database: Web of Science Core Collection

# Entitlements:

- WOS.SCI: 1900 to 2022
- WOS.AHCI: 1975 to 2022
- WOS.ESCI: 2015 to 2022
- WOS.ISTP: 1990 to 2022
- WOS.SSCI: 1900 to 2022
- WOS.ISSHP: 1990 to 2022

# Searches:

Search: #32 AND #6 and 2021 or 2022 (Publication Years)

Search: #32 AND #6

Search: #9 OR #10 OR #11 OR #12 OR #13 OR #14 OR #15 OR #16 OR #17 OR #18 OR #19 OR #20 OR #21 OR #22 OR #23 OR #24 OR #25 OR #26 OR #27 OR #28 OR #29 OR #30 OR #31 Results: 979472

#31. Search: TS=("organi$ational culture" )

#30. Search: "non-punitive" (Topic)

#29. Search: "just culture*" (Topic)

#28. Search: "psychologic* safe*" (Topic)

#27. Search: TS=(safe* NEAR/2 culture*)

#26. Search: TS=("simulation training")

#25. Search: TS=((Institution* NEAR/2 support) OR (Institution* NEAR/2 response*) OR (Institution* NEAR/2 respond*))

#24. Search: #6 AND #8

#23. Search: TS=("Resilience in Stressful Events")

#22. Search: #7 AND #6

#21. Search: "Intervention training" OR crisis NEAR/2 training (Topic)

#20. Search: TS=("psychological resilience")

#19. Search: TS="Schwartz Center Rounds"

#18. Search: (TS=((Resilienc* NEAR/2 training) OR (resilienc* NEAR/2 intervention*) OR (promot* NEAR/2 resilienc*)))

#17. Search: TS=(((support* NEAR/2 ("second victim*" OR organi$ation* OR service* OR system* OR program* OR resource* OR social))) )

#16. Search: Debrief* (Topic)

#15. Search: ("peer group*" OR (peer* NEAR/3 support*)) (Topic)

#14. Search: "Second Victim Experience and Support Tool" OR SVEST (Topic)

#13. Search: "critical incident stress management" OR "critical incident stress reporting system*" OR "critical incident stress debrief*" (Topic)

#12. Search: "support*" (Title) OR "support*" (Author Keywords)

#11. Search: TS=(("psychological stress" or "post traumatic stress disorder*" or PTSD) AND (therap* or prevention))

#10. Search: TS=(counselling or counseling)

#9. Search: TS=("crisis intervention"*)

#8. Search: TS=("Occupational Health Services")

#7. Search: TS=("social support")

#6. Search: #5 AND #4 OR ALL=("second victim*")

#5. Search: TS=( ((Health NEAR/2 professional*) OR (health NEAR/2 provider*)) OR "health

personnel" OR "healthcare profession*" OR (healthcare NEAR/2 provider*) OR "healthcare worker*" OR "health care worker*" OR (worker* NEAR/2 (health OR healthcare)) OR nurs* OR
physician* OR surgeon* OR midwife or midwives or mid OR "clinician*")

#4. Search: #3 OR #2 OR #1 OR TS=("medical errors" )

#3. Search: ("serious incident$" OR "critical incident$" OR "never event$" OR "near miss*" OR "sentinel event$" OR "adverse patient occurrence$" OR "serious reportable event$" OR (serious NEAR/2 event$ NEAR/3 (clinical OR critical OR medical OR surgical)) OR "preventable adverse event$" OR "avoidable adverse event$" OR ((Medical OR surgical) NEAR/2 Adverse NEAR/2 Event$)) (Topic)

#2. Search: ((avoidable OR preventable OR unsafe OR undesirable) NEAR/1 (event* OR outcome* OR complication* OR death OR accident* OR injur*)) (Topic)



Simms-Ellis R, *et al. BMJ Open* 2025; 15:e087512. doi: 10.1136/bmjopen-2024-087512

#1. Search: ((critical OR "patient safety" OR harmful OR clinical) NEAR/0 incident*) (Topic)
