# Supplementary material for: Avoiding ‘second victims’ in healthcare: what support do staff want for coping with patient safety incidents, what do they get and is it effective? A systematic review
Source: BMJ Open. 2025 Feb 10;15(2):e087512. doi: 10.1136/bmjopen-2024-087512 (PMC12185930; doi:10.1136/bmjopen-2024-087512)
Supplement: Supplementary data [file bmjopen-15-2-s004.pdf]

Supplementary 4 File: Narrative summary findings from surveys and qualitative studies included at Stage 1 (n=72)

1. What support do staff want for coping with Patient Safety Incidents (RQ1)?

1.1 *__Surveys (n=49; papers 1-49)__*

Thirty-four survey studies explored the support that staff would want following an adverse event to help second victims recover (1, 3, 4, 5, 7, 8, 9, 10, 12, 14, 15, 16, 17, 18, 19, 20, 24, 25, 26, 27, 29, 31, 32, 34, 35, 36, 37, 38, 41, 42, 44, 45, 47, 48).

Peer support was an aspect that arose in many survey studies (n = 23) and was rated as the most desired support strategy by every study that ranked supports (1, 3, 4, 10, 15, 16, 18, 19, 27, 32, 38, 41, 44, 45, 46, 47). Of the survey studies that gave figures, between 59% and 92.1% of the samples would want peer support following an event (4, 15, 18, 19, 27, 41, 44, 45, 47). However, the term ‘peer support’ was used differently between studies. It was primarily referred to as ‘a respected peer to discuss the details of what happened’ in 9 of the 23 studies (4, 16, 28, 33, 38, 39, 45, 46, 47); other definitions included emotional support (8, 9), conversations with peers (10, 19, 20), confidential support (17), talking to someone (32), discussing emotional issues (36, 37) or support from close people on a professional or personal level (42, 43). There were differences in who second victims wanted to receive peer support from, with some preferring conversations with direct colleagues (1, 3, 8, 41, 42) and others desiring to speak with the whole team (18, 31, 41) or other physicians (3). One survey study found that informal emotional support was one of the least wanted methods of support despite being one of the most utilised (7).

There were a variety of forms of support provided by organisations for second victims that were referenced across the survey studies. Of the survey studies that ranked strategies, the support that was rated in the top 3 by the second most studies was having ‘a specified peaceful location that is available to recover and recompose’ (4, 9, 27, 32, 44, 45, 47), with between 61.4% and 78.8% of samples wanting this support. Debriefs with the colleagues involved in the incident were wanted by some participants (7, 14, 16, 18, 35, 36, 48), although it was not always clear how this was different to peer support. Multiple survey studies found that second victims wanted information about the incident, including ethical issues (7, 35), causes and preventative measures (7, 26, 29, 34, 35, 36, 41). Several participants were found to desire crisis interventions (7, 16, 35, 36),

including guidance and advice regarding legal proceedings following the event (7, 16, 26, 34, 36).

Counselling was desired as a method of support for second victims in thirteen survey studies (1, 7, 14, 15, 16, 17, 19, 25, 27, 34, 35, 36, 37). Of the survey studies that ranked support strategies, counselling was rated in the top 3 by the joint third most studies (1, 16, 19, 25, 37, 38), with between 29.9% and 96.1% of samples desiring this support. However, one sample found it to be the least desired of the strategies (4).There were inconsistencies in how this was phrased; some participants wanted to be supported by a professional and receive formal support (1, 7, 14, 15, 16, 18, 19, 24, 25) whilst others simply wanted access to formal psychological services or the opportunity to see a counsellor if they felt like they needed it (4, 7, 17, 34, 35, 36, 37). Similarly, employee assistance programs were sought in some studies, which could provide support to second victims outside the workplace (16, 28, 35, 38, 48).

1.2 <u>Qualitative studies (n=23; papers 50-72)</u>

Eighteen qualitative studies explored staff needs following an adverse event and what support they believed would be most appropriate in helping second victims recover (50, 51, 52, 53, 54, 56, 70, 71, 57, 58, 59, 62, 63, 64, 65, 66, 67, 68). Of these, thirteen studies reported staff as wanting their organisation‘s approach and support to accommodate them and the incident (51, 53, 54, 56, 70, 71, 58, 59, 62, 63, 66, 67, 68), such as being protected, recognised and avoiding blame or criticism (51, 54, 70, 59). Across the qualitative studies, organisational support also included being freed from workloads, reviewing the staff member’s responsibilities (54), allowing tasks and responsibilities to be completed with a colleague (62) and follow-up or long-term support (70), no time limit on recovery (68) recognition that the impact of an event may differ over time (53, 67). Several qualitative/other studies reported that second victims may not seek support even if needed, so organisations and supervisors need to be proactive in reaching out (52, 64). Some staff emphasised a necessity for professional reassurance in emotional recovery (56, 67), with second victims wanting guidance, reassurance and an awareness of management’s confidence in them (54, 66).

Staff from twelve qualitative studies desired emotional support and having someone to talk to (54, 70, 57, 66), with eight specifying peers and colleagues as a desired audience (52, 53, 56, 71,

58, 59, 63, 67, 68) and three looking for managerial support (64, 66, 68). Staff described the ideal supporter as a non-judgemental and empathetic colleague from their direct work environment who could acknowledge and reassure the second victim (70, 58, 67). Structural peer support was suggested as a strategy (52, 63), such as colleagues assisting the second victim with workload or taking time to listen (54), with one study finding that informal peer support was the most desired support method (68).

Eight samples of staff were found to seek formal support, counselling and/or specialist mental health resources (50, 51, 53, 71, 57, 62, 63, 68), which could be accessed by those who had been seriously impacted by an incident (59, 68). Staff across several studies recommended confidential counselling programs provided directly by the organisation (51, 71, 63), although one study reported that there were mixed views about whether this would be accepted within surgical communities (62).

Staff from further six qualitative studies wanted time away from work or a period of leave to help distance themselves from the event and recover (51, 54, 59, 62, 66, 68). Furthermore, six studies found staff seeking practical support in the form of debriefs and discussions (70, 71), administrative support with investigations (56, 57) as well as information, evaluation and learning around the incident (56, 70, 67, 68). Some staff expressed a need to be supported in communicating with the affected patient or family following an incident (62, 68) and be informed of the patient's outcomes (56). Samples from multiple qualitative studies also suggested value in sharing their experiences with other colleagues to share learning around the second victim experience (70, 63, 68).

1.3 Additional information from qualitative studies related to what staff want

- Emotional support following an adverse event was described as the "first measure" following an incident (54) and essential in effectively supporting second victims (54, 57, 59, 66, 67).
- One study emphasised that support should focus on the staff's emotions instead of the cause of the incident (59).
- Many staff across studies reported the need for second victims to feel heard, and that psychosocial support should address the need for them to talk (54, 70, 63, 67).

Simms-Ellis R, *et al. BMJ Open* 2025; 15:e087512. doi: 10.1136/bmjopen-2024-087512

- Second victims were reported to need general concern and empathy around wellbeing from colleagues and superiors (71).
- One study's sample suggested the implementation of a mentoring system, giving staff an opportunity to talk with seniors for problem solving (62).
- Across the studies, staff reported wanting access to psychological support and specialist resources (52, 57, 62, 63, 66).
- Some participants wanted administrative and institutional support to help individuals overcome psychological difficulties, and address adverse events and countermeasures (57, 67).
- Multiple participants raised a concern that the culture of healthcare services served as a barrier for blame-free approaches; environments were described as inconsiderate to emotional wellbeing (71) and vulnerability (58) post-incident, and a shift to a caring, gossip-free culture was needed at organisational and individual levels (58, 59). Leadership is needed to promote a safe atmosphere to process patient safety incidents (71), and one study suggested that communicative skills such as empathy should be essential when hiring senior members of staff (58). Another study reported that learning can only take place in a psychologically safe setting (65).
- Although studies noted a variation in the amount of support needed by second victims, recognition of their actions and collegial trust were reported as the minimum level required (56). Investigations into adverse events were found as a potential barrier to recovery, as they could create a sense of anxiety for second victims (56). Therefore, it was important for staff to receive guidance and support for the investigation process and for supervisors to emphasise that the investigations are for system failures, not punishment (56, 67, 68). One study reported a need to discuss working conditions, as this was not always considered during investigations, which was frustrating for the staff involved (67).
- Administrative and practical needs were also identified in various studies. Some staff reported confusion about the post-incident procedure, thereby necessitating organisational guidance on this process (56, 68).
- One study found that the ideal listener differed depending on the culture of the organisation; in 'open culture' organisations, staff preferred talking to internal colleagues, whereas staff in 'blame cultures' preferred an external individual with the same professional background (68).

A minority of participants from another study suggested that internal peer support may tamper with team relations, and that organisations should have a general, hospital-wide support service, although other participants argued that this was ineffective and a reflection of the hospital culture being insensitive to vulnerability (58). Several studies advocated for peer support being organisation-led instead of relying on individuals to take initiative (51, 71, 67), such as by stimulating intercollegiate conversations around support following an event (58). Follow-up conversations were also suggested by one study in order to account for differing long-term needs (52).

- One study suggested that counselling could be mandatory, as second victims need to address and open up about the event to be competent in the role moving forward (50), with another noting the potential prevalence of PTSD (57).

2. What support do staff receive to cope with Patient Safety Incidents (RQ2)?

2.1 ***<u>Surveys (n=49; papers 1-49)</u>***

Thirty-four survey studies explored the support that staff experienced following an adverse event (1, 2, 3, 5, 6, 8, 10, 11, 12, 13, 16, 17, 20, 21, 22, 23, 24, 28, 30, 31, 33, 35, 36, 38, 39, 40, 41, 42, 43, 45, 46, 47, 48, 49).

Peer support was addressed in twenty-five studies (2, 3, 6, 8, 10, 11, 12, 13, 16, 17, 20, 21, 22, 23, 24, 30, 33, 35, 36, 38, 39, 41, 42, 49) and was rated as both the most experienced (8, 12, 21, 30, 35, 38, 41, 49) and most useful (6, 8, 11, 13, 16, 17, 21) by the majority of studies that ranked supports. Most of the studies that gave figures, reported between 49% and 100% of their samples experiencing peer support (12, 21, 30, 33, 35, 38, 39, 42, 49), although peer support was experienced by less than 1% of samples in two other studies (45, 47). Second victims were found to talk to direct colleagues (1, 2, 3, 21, 22, 23, 24, 35, 39, 41, 49), especially those at a similar rank (13, 20, 41) and those directly involved in the incident (41). Second victims seeking emotional support were primarily looking for peers who could understand the situation in order to manage their own stress, bring relief and prevent burnout (2, 6, 10, 49). Where effectiveness was measured, peer support was largely seen as useful and helpful, including in studies using statistical analyses (2, 6, 8, 17, 20, 35, 36) and quantitative analysis (12), and between 42% and

94.1% of samples found peer support to be useful. Conversely, one statistical sample reported mixed ratings (33).

Participants also reported experiencing a variety of institutional support strategies. Receiving support from supervisors and department leaders was commonly referenced (6, 13, 24, 30, 33, 35, 42, 43, 45, 49) and was found to be both the second most experienced and second most useful support in studies that ranked strategies (6, 13 24, 42, 43). Managerial support was rated as valuable in several studies (6, 13, 43), whilst others found it to be mixed (33) or insufficient (42, 45, 47). Mortality and Morbidity meetings or patient safety incident discussions were referenced in several studies (2, 17, 28, 43, 49), and were the joint second most commonly experienced support amongst studies that ranked strategies (3, 43, 49), with 67.3% of one sample finding them to be helpful for second victims (28). Debriefs were also experienced by staff (3, 8, 17, 35, 48) and were ranked as the joint second most helpful, with 89.4% to 100% of samples finding these to be useful (17, 35, 36, 48). However, others found the meetings to be unsupportive, neutral or negative to recovery (10, 28, 43). Counselling was also addressed in multiple studies and was reported as useful (11, 17, 20, 35), albeit less experienced than other support options (35, 49).

2.2 <u>Qualitative studies (n=23; papers 50-72)</u>

All twenty-three qualitative studies explored the support that staff received following a patient safety incident. Of the ten that reviewed the support provided, all found it to be insufficient or inconsistent (50, 53, 56, 70, 58, 61, 62, 63, 66, 67). One study reported 82% of its staff as having no support and an unfriendly atmosphere (63), whilst staff in another reported that 65% staff did not have all needs addressed, and that 37% had no support whatsoever (70). Support varied both between and within roles; several studies reported a lack of guidance or a consistent process (53, 69, 56, 70, 58, 61, 67), staff being unaware of the support available (70, 58, 61, 63, 66) and certain staff receiving different levels of support based on their position (52, 58, 64, 68).

Twenty-one studies reported experiences with peer support (50, 51, 52, 53, 55, 69, 56, 70, 71, 57, 58, 59, 60, 62, 72, 63, 64, 65, 66, 67, 68), and it was commonly utilised or seen as helpful in all studies when done in the right way. However there is definitely inconsistency in the way peer support is considered as a

'support': some studies imply that it is facilitated by the organisation, yet others imply that it is different from organisational support, and simply involves SVs talking to colleagues.

Seven viewed peer support as the most effective form of support or the support that they would typically/first turn to following an event (53, 70, 71, 62, 63, 64, 67). In several studies, the experience of the listening colleague was crucial to the effectiveness of peer support, with the ideal listener being able to fully understand the nature of the job and the incident itself (50, 69, 71, 57, 59, 60, 62, 72, 65, 66, 67, 68). Multiple participants also emphasised that to best support them, the listener should be non-judgemental and refrain from criticising or blaming the second victim (52, 66, 68). As well as providing emotional support (53), many of the healthcare staff members found that peer support could help the second victim with practical advice and reassure them about their capability in the role, which helped them in regaining professional confidence (53, 58, 59, 60, 62, 72, 65, 68). Other studies noted the benefits of peers sharing mistakes of their own (51, 72) and checking in with the second victim generally (51). Support from other second victims was reported as helpful, but only if they were understanding and non-judgemental of the event (68). Conversely, several participants emphasised that peer support had to be conducted in the right way, as colleagues who were too critical or judgmental (53, 59, 60, 65, 68), minimised the event (53, 60, 72, 68) or were overly reassuring (60, 68) could make the second victims feel worse. Attempts to solve the error (72), peers reaching out due to curiosity rather than compassion (68) and small comments that could make second victims feel in the wrong (65) were also seen as barriers to successful peer support.

Twelve qualitative studies addressed managerial support (50, 52, 69, 70, 71, 59, 60, 61, 63, 65, 66, 67), with four finding it to be helpful (52, 71, 59, 66) yet six others finding it to be insufficient (50, 60, 61, 67) or inconsistent (52, 70). In one study, 22% of participants said that informal emotional support from supervisors was the most helpful strategy, whilst 15% found informal emotional support from managers to be best (although 12% also said this was the least helpful support) (70). Examples of effective managerial support included giving staff space without chasing up incident reports (59), celebrating staff in team meetings to facilitate positivity, providing support via team meetings or individual conversations and generally being proactive and accessible after an event (52), which helped to resolve emotional stress (71, 59). Ineffective managerial support led to feelings of isolation (50, 60) and hindered recovery (61, 67)

Other organisational support methods included debriefs and other discussions around the event, which were referenced by six studies (52, 70, 57, 62, 64, 65). Four of these samples found debriefs to be ineffective due to inconsistencies in their implementation (70, 62, 65), certain staff not being involved and focusing on learning and blaming rather than emotional recovery (52, 65). In one study, 'defusing' was suggested, which was distinctive from formal debriefs and allowed for staff to voice their reactions and prepare for debrief (52). One group of participants referenced Mortality and Morbidity meetings, finding them to be ineffective due to a focus on learning and some staff not being invited (64). Learning was referenced by another six studies (55, 70, 60, 62, 72, 68), with staff finding meaning in discussing the case and evaluating what went wrong (55, 62, 68), teaching others about their experience (60, 68) and developing changes to prevent future incidents (70, 72). Some staff sought help with the administrative and legal aspects of an incident, which was appreciated (68).

Six studies referred to counselling and formal support (50, 55, 56, 70, 63, 68) which was used by staff in five (55, 56, 70, 63, 68). This was seen as helpful in two studies due to it being personalised, objective, empathetic and non-judgmental (56, 68), but seen as the least helpful support by 11% of another sample (70). When these services were not offered by the organisation, participants sought professional help themselves, with 40% of one sample seeking this (63). Taking time away from work helped staff distance themselves from the event in one study (50), but was refused by the organisation in another (54).

2.3 Additional information from qualitative studies related to what staff received

- Certain staff were not invited to Morbidity and Mortality meetings (64) therefore this was seen as an inadequate approach to support all those involved in a PSI.
- In many studies, peer support was seen as helpful for recovery and the emotional wellbeing of the staff involved (51, 52, 53, 55, 69, 56, 70, 71, 58). For some participants, informal peer support helped them feel heard (50, 72). Several studies reported that peer support could be detrimental to the recovery of second victims if done in the wrong way. In one paper, one third of participants experienced issues with their collegial relationships as a result of the failure of peer support, which sometimes led to dispute in the organisation (67). The failure to support the second victim generally was also found to damage working relationships and

leave the individual feeling unsupported and anxious (56). Multiple studies noted that colleagues ‘minimising’ the situation did not help, and that it was important for peers to be open and honest about the incident (53, 60, 72). Studies reported that conversations needed to address the coping needs of the physician at the time (60), and that physicians responded best to peers who listened attentively and showed empathy (72). Negative examples of peer support included criticism, blame, being made to feel incompetent (53, 60), attempts to solve the error (72) and peers reaching out due to curiosity, not compassion (68). In one case, small comments were enough to make second victims feel in the wrong (65). Being protected by friends (60) and having colleagues who were resistant to discussion (68) were also barriers to successful peer support.

- In one study, 86% of participants said they would typically turn to a colleague, and 48% received emotional intercollegiate support following a patient safety incident (70), with 69% seeking peer support in another study (71). and receiving empathy from colleagues was seen as beneficial (51, 59).
- Multiple findings emerged concerning the lack of formal structures in place in some institutions, meaning support was based on the individuals around the second victim (70, 61). The absence of structure was attributed as a source of staff isolation by one study (67).
- Some of the inconsistencies described across the support received were related to the positions of the staff involved. Some participants reported feeling unrepresented in the support process; one study found that support for nurses and ODPs was inadequate in comparison to clinicians involved, and that certain staff were not invited to Morbidity and Mortality meetings (64). In one case, healthcare professionals were excluded from investigations about their incidents, which was detrimental to their recovery (68). One study found that clinicians felt unrepresented in their organisation’s support service, and that the social science professionals that worked in the service would be unable to understand the clinical context (58). A lack of conversation and discussion about an incident was sometimes reported (67), and the silence served as a barrier to reflection and learning (60). One study found many participants perceiving support as unengaging and minimal, leading to feelings of isolation (50). Another described organisational approaches as ‘punitive’ and inconsiderate to the systemic factors that contribute to incidents (62). Regarding legality, one study reported that rules and sanctions were barriers to support, and that organisational

interpretations of these previsions were biased against second victims (61). Conversely, one paper reported staff being supported and taken seriously by the organisation, with the service listening and guiding staff through an event (64).

- Several studies addressed the emotional element involved in support. One study found it to be the most relevant concern following an incident, and that its absence led to isolation for second victims, although it could also be unwanted by certain staff or in certain approaches, such as when it was too soothing or came from someone with minimal experience (68). For some, confidentiality rules made staff reliant on workplace support by preventing them from talking to outsiders, meaning that insufficient workplace support led to feelings of abandonment (65, 67). Emotional support contrasted with Morbidity and Mortality meeting and more practical responses to incidents (72) and was helpful, but organisations sometimes lacked in guidance around it (69) – some staff were unaware of who they could talk to, leading to ineffective support that 85% of one sample was unsatisfied with (63). A lack of emotional support was found to be one of the least helpful responses identified by one study (70).
- Some healthcare staff also turned to managers and supervisors in response to a patient safety incident. In one sample of student nurses, professors were the 'first and primary support' accessed (66), and several studies addressed participants reaching out to managers, supervisors or risk management for support (70, 71). In one study, 22% of participants said that informal emotional support from supervisors was the most helpful strategy, whilst 15% found informal emotional support from managers to be best (although 12% also said this was the least helpful support) (70). Effective support from managers helped to resolve emotional stress (71, 59), facilitate learning from the incident and provide reassurance surrounding competence (60). Several studies reported ineffective managerial support (67), resulting in second victims feeling unsupported and abandoned (50, 60, 66). Proactivity of managers was reported as beneficial in one paper, and included providing support via team meetings, individual conversations, formal peer support and generally being accessible after an event (52). Reactive or passive managers were reported to be too late or non-existent in their support, hindering second victim recovery (52, 61). Other examples of effective managerial support included giving staff space without chasing up incident reports (59) and celebrating staff in team meetings, facilitating trust and a culture of support (52). 'Defusing' was also

suggested, which was distinctive from formal debriefs, allowed for staff to voice their reactions and prepare for debrief (52).

- In some studies, debriefs were suggested as a method for staff to feel less isolated, more supported and help coping generally (64, 65), with incidents being discussed through seniors meetings and interrogations (57). However, the frequency and quality of debriefs varied widely. Debriefs were reported as rare in some studies (62), with only 13% of participants in one study receiving them promptly (70). One study found them to be inconsistent in frequency (sometimes too few, sometimes too many) and participation, with some staff not being invited or deciding not to attend (65). For second victims, this limited the effectiveness of the debriefs and their quality overall, preventing healthcare professionals from receiving guidance. In one case, midwives were found to commonly be excluded from investigations and interprofessional debriefing, with many of them subsequently perceiving investigations as scapegoating and attributing blame (52). Another study noted that debriefs sometimes turned to explicit or implicit blaming of staff for the incident (65), with a focus on learning neglecting the emotional need of second victims. Debriefs were also limited when seniors did not conduct the debriefs (as they only were expected to lead these sessions if they were involved in the incident), forcing healthcare staff to attempt conduction of the debrief themselves (65). 11% of one sample reported debriefs and crisis interventions as the least helpful support (64), and another study noted that any welfare extracted from debriefs is not beneficial to second victims unless later acted upon (64).
- Several studies addressed second victims using their experiences in incidents to facilitate learning and teach others. In one sample, 12% of staff were involved in a learning process, with 11% describing it as the most helpful support (70). Learning was enabled via intercollegiate conversations (60, 72) and analysis of the incident (62, 68), helping to facilitate processing of the incident (72). In one instance, midwives were often excluded from learning opportunities, serving as a barrier to their recovery (52). Some second victims further used their experiences and learning to teach colleagues and prevent future incidents of a similar nature (60, 72, 68). This not only gave second victims an outlet to talk about the experience, but also allowed them to develop system changes (72), and teaching helped facilitate their own recovery (60). Other suggested methods of learning included staff ensuring their skills were sufficient (62), supervision and case discussions (55). Mentoring

was suggested in one case, but effective mentoring was reported as rare (62). One study noted that a focus on learning (especially during Mortality and Morbidity meetings) was ineffective at supporting staff emotionally (64), with another noting that emotional vulnerability that was not dealt with served as a barrier to learning (65).

- Other methods of support were addressed by several studies, including the use of counselling or professional support. Some organisations reportedly provided staff with professional services, allowing serious impacts to be treated and time to adequately recover (50, 55, 56). When these services were not offered by the organisation, participants sought professional help themselves, with 40% of one sample seeking this (63). Counselling was beneficial for second victims as it was "personalised, objective, empathetic and non-judgemental" (68). Some staff reported that conversations with the patients and families involved in incidents was beneficial for recovery, allowing second victims to address the incident directly, apologise and find closure (60, 62, 72). Other staff sought leave to help distance themselves from the incident and recover (50), although one study reported the hospital refusing this request (54). Some nurses in one sample changed their work routines to facilitate processing of the experience, such as having back-up in case they decide they are unable to complete the shift (55). Having time for group reflection that focused on the emotional impact of an incident was suggested (71), as well as creating an atmosphere of shared responsibility and positivity (59). Some staff sought help with the administrative and legal aspects of an incident (68).
- One paper reported that a lack of support from colleagues and seniors could damage intercollegiate trust and relationships, leaving the second victim isolated and anxious (56).

<u>Reference List: Surveys and qualitative studies included at Stage 1 (n=72)</u>

Study ID	Study Reference	Explored what staff wanted	Explored what staff received

1	Baas MA, Scheepstra KW, Stramrood CA, Evers R, Dijksman LM, van Pampus MG. Work-related adverse events leaving their mark: a cross-sectional study among Dutch gynecologists. BMC Psychiatry. 2018 Dec;18:1-8.	X	X
2	Bañeras J, Jorge-Pérez P, Bonanad C, Lluva MT, Moll I, Kinori SG. Second victims and quality of support resources among cardiology professionals. Revista Espanola de Cardiologia (English ed.). 2022 Aug;75(8):686-8.		X
3	Berman L, Rialon KL, Mueller CM, Ottosen M, Weintraub A, Coakley B, Brandt ML, Heiss K. Supporting recovery after adverse events: an essential component of surgeon well-being. Journal of Pediatric Surgery. 2021 May 1;56(5):833-8.	X	X
4	Burlison JD, Scott SD, Browne EK, Thompson SG, Hoffman JM. The second victim experience and support tool (SVEST): validation of an organizational resource for assessing second victim effects and the quality of support resources. Journal of Patient Safety. 2017 Jun;13(2):93.	X	
5	Choi EY, Pyo J, Ock M, Lee H. Profiles of second victim symptoms and desired support strategies among Korean nurses: A latent profile analysis. Journal of Advanced Nursing. 2022 Sep;78(9):2872-83.	X	X
6	Draus C, Mianecki TB, Musgrove H, Bastien DJ, Greggs D, Halash C, Lewis A, Mackenzie W. Perceptions of Nurses Who Are Second Victims in a Hospital Setting. Journal of Nursing Care Quality. 2022 Apr 20;37(2):110-6.		X
7	Edrees HH, Paine LA, Feroli ER, Wu AW. Health care workers as second victims of medical errors. Pol Arch Med Wewn. 2011 Apr 1;121(4):101-8.	X	
8	Fatima S, Soria S, Esteban-Cruciani N. Medical errors during training: how do residents cope?: a descriptive study. BMC Medical Education. 2021 Dec;21(1):1-6.	X	X
9	Finney RE, Torbenson VE, Riggan KA, Weaver AL, Long ME, Allyse MA, Rivera-Chiauzzi EY. Second victim experiences of nurses in obstetrics and gynaecology: A second victim experience and support tool survey. Journal of Nursing Management. 2021 May;29(4):642-52	X	
10	Gupta K, Rivadeneira NA, Lisker S, Chahal K, Gross N, Sarkar U. Multispecialty physician online survey reveals that burnout related to adverse event involvement may be mitigated by peer support. Journal of Patient Safety. 2022 Sep 1;18(6):531-8.	X	X
11	Han K, Bohnen JD, Peponis T, Martinez M, Nandan A, Yeh DD, Lee J, Demoya M, Velmahos G, Kaafarani HM. The surgeon as the second victim? Results of the Boston Intraoperative Adverse Events Surgeons' Attitude (BISA) study. Journal of the American College of Surgeons. 2017 Jun		X

	1;224(6):1048-56.		
12	Harrison R, Lee H, Sharma A. A survey of the impact of patient adverse events and near misses on anaesthetists in Australia and New Zealand. Anaesthesia and Intensive Care. 2018 Sep;46(5):510-5.	X	X
13	Harrison R, Sharma A, Walton M, Esguerra E, Onobrakpor S, Nghia BT, Chinh ND. Responding to adverse patient safety events in Viet Nam. BMC Health Services Research. 2019 Dec;19:1-8.		X
14	Heard GC, Thomas RD, Sanderson PM. In the aftermath: attitudes of anesthesiologists to supportive strategies after an unexpected intraoperative patient death. Anesthesia & Analgesia. 2016 May 1;122(5):1614-24.	X	
15	Huang R, Sun H, Chen G, Li Y, Wang J. Second-victim experience and support among nurses in mainland China. Journal of Nursing Management. 2022 Jan;30(1):260-7.	X	
16	Jain G, Sharma D, Agarwal P, Agrawal V, Yadav SK, Tenzin T, Alamgir MH, Manandhar K, Myint M, Chaudhary AM, Jami AA. “Second Victim” syndrome among the surgeons from South Asia. Indian Journal of Surgery. 2022 Feb;84(1):40-6.	X	X
17	Joesten L, Cipparrone N, Okuno-Jones S, DuBose ER. Assessing the perceived level of institutional support for the second victim after a patient safety event. Journal of Patient Safety. 2015 Jun 1;11(2):73-8.	X	X
18	Kaur AP, Levinson AT, Monteiro JF, Carino GP. The impact of errors on healthcare professionals in the critical care setting. Journal of Critical Care. 2019 Aug 1;52:16-21.	X	
19	Kerkman T, Dijksman LM, Baas MA, Evers R, van Pampus MG, Stramrood CA. Traumatic experiences and the midwifery profession: a cross-sectional study among dutch midwives. Journal of Midwifery and Women's Health. 2019 Jul;64(4):435-42.	X	
20	Khansa I, Pearson GD. Coping and recovery in surgical residents after adverse events: the second victim phenomenon. Plastic and Reconstructive Surgery Global Open. 2022 Mar;10(3).	X	X
21	Lin JS, Olutoye OO, Samora JB. To Err is human, but what happens when surgeons Err?. Journal of Pediatric Surgery. 2023 Mar 1;58(3):496-502.		X
22	Magaldi M, Perdomo JM, López-Baamonde M, Chanzá M, Sanchez D, Gomar C. Second victim phenomenon in a surgical area: online survey. Revista Española de Anestesiología y Reanimación (English Edition). 2021 Nov 1;68(9):504-12.		X
23	Martens J, Van Gerven E, Lannoy K, Panella M, Euwema M,		X

	Sermeus W, De Hert M, Vanhaecht K. Serious reportable events within the inpatient mental health care: Impact on physicians and nurses. Revista de Calidad Asistencial. 2016 Jul 1;31:26-33.		
24	Mathebula LC, Filmalter CJ, Jordaan J, Heyns T. Second victim experiences of healthcare providers after adverse events: A cross-sectional study. Health SA Gesondheid (Online). 2022;27:1-6.	X	X
25	McLennan SR, Engel-Glatter S, Meyer AH, Schwappach DL, Scheidegger DH, Elger BS. The impact of medical errors on Swiss anaesthesiologists: a cross-sectional survey. Acta Anaesthesiologica Scandinavica. 2015 Sep;59(8):990-8.	X	
26	Mohamadi-Bolbanabad A, Moradi G, Piroozi B, Safari H, Asadi H, Nasseri K, Mohammadi H, Afkhamzadeh A. The second victims' experience and related factors among medical staff. International Journal of Workplace Health Management. 2019 May 15;12(3):134-45.	X	
27	Mok WQ, Chin GF, Yap SF, Wang W. A cross-sectional survey on nurses' second victim experience and quality of support resources in Singapore. Journal of Nursing Management. 2020 Mar;28(2):286-93.	X	
28	Nijs K, Seys D, Coppens S, Van De Velde M, Vanhaecht K. Second victim support structures in anaesthesia: a cross-sectional survey in Belgian anaesthesiologists. International Journal for Quality in Health Care. 2021 Apr 1;33(2):mzab058.		X
29	O'Meara S, D'Arcy F, Dowling C, Walsh K. The psychological impact of adverse events on urology trainees. Irish Journal of Medical Science (1971-). 2023 Aug;192(4):1819-24	X	
30	Rivera-Chiauzzi E, Finney RE, Riggan KA, Weaver AL, Long ME, Torbenson VE, Allyse MA. Understanding the second victim experience among multidisciplinary providers in obstetrics and gynecology. Journal of Patient Safety. 2022 Mar 1;18(2):e463-9.		X
31	Rodriquez J, Scott SD. When clinicians drop out and start over after adverse events. The Joint Commission Journal on Quality and Patient Safety. 2018 Mar 1;44(3):137-45.	X	X
32	Santana-Domínguez I, González-De La Torre H, Verdú-Soriano J, Berenguer-Pérez M, Suárez-Sánchez JJ, Martín-Martínez A. Feelings of being a second victim among Spanish midwives and obstetricians. Nursing Open. 2022 Sep;9(5):2356-69.	X	
33	Schrøder K, Edrees HH, Christensen RD, Jørgensen JS, Lamont RF, Hvidt NC. Second victims in the labor ward: Are Danish midwives and obstetricians getting the support they need?. International Journal for Quality in Health Care. 2019 Oct 31;31(8):583-9.		X

34	Shuangjiang Z, Huanhuan H, Ling X, Qinghua Z, Mingzhao X. Second victim experience and support desire among nurses working at regional levels in China. Journal of Nursing Management. 2022 Apr;30(3):767-76.	X	
35	Strametz R, Fendel JC, Koch P, Roesner H, Zilezinski M, Bushuven S, Raspe M. Prevalence of Second Victims, Risk Factors, and Support Strategies among German Nurses (SeViD-II Survey). International Journal of Environmental Research and Public Health. 2021 Oct 10;18(20):10594.	X	X
36	Strametz R, Koch P, Vogelgesang A, Burbridge A, Rösner H, Abloescher M, Huf W, Ettl B, Raspe M. Prevalence of second victims, risk factors and support strategies among young German physicians in internal medicine (SeViD-I survey). Journal of Occupational Medicine and Toxicology. 2021 Dec;16(1):1-1.	X	X
37	Sun L, Deng J, Xu J, Ye X. Rumination's role in second victim nurses' recovery from psychological trauma: a cross-sectional study in China. Frontiers in Psychology. 2022 May 3;13:860902.	X	
38	Torbenson VE, Riggan KA, Weaver AL, Long ME, Finney RE, Allyse MA, Rivera-Chiauzzi E. Second victim experience among OBGYN trainees: what is their desired form of support? Southern Medical Journal. 2021 Apr;114(4):218.	X	X
39	Turner K, Bolderston H, Thomas K, Greville-Harris M, Withers C, McDougall S. Impact of adverse events on surgeons. British Journal of Surgery. 2022 Apr 2;109(4):308-10.		X
40	Van Gerven E, Bruyneel L, Panella M, Euwema M, Sermeus W, Vanhaecht K. Psychological impact and recovery after involvement in a patient safety incident: a repeated measures analysis. BMJ Open. 2016 Aug 1;6(8):e01140.		X
41	Vanhaecht K, Zeeman G, Schouten L, Bruyneel L, Coeckelberghs E, Panella M, Seys D, Dutch Peer Support Collaborative Research Group. Peer support by interprofessional health care providers in aftermath of patient safety incidents: A cross-sectional study. Journal of Nursing Management. 2021 Oct;29(7):2270-7.	X	X
42	Van Slambrouck L, Verschueren R, Seys D, Bruyneel L, Panella M, Vanhaecht K. Second victims among baccalaureate nursing students in the aftermath of a patient safety incident: An exploratory cross-sectional study. Journal of Professional Nursing. 2021 Jul 1;37(4):765-70.	X	X
43	Vinson AE, Mitchell JD. Assessing levels of support for residents following adverse outcomes: a national survey of anesthesia residency programs in the United States. Medical Teacher. 2014 Oct 1;36(10):858-66.		X

44	Winning AM, Merandi J, Rausch JR, Liao N, Hoffman JM, Burlison JD, Gerhardt CA. Validation of the second victim experience and support tool-revised in the neonatal intensive care unit. Journal of Patient Safety. 2021 Dec 1;17(8):531-40.	X	
45	Wolf M, Smith K, Basu M, Heiss K. The Prevalence of Second Victim Syndrome and Emotional Distress in Pediatric Intensive Care Providers. Journal of Pediatric Intensive Care. 2021 Jul 1;12(02):125-30.	X	X
46	Yan L, Tan J, Chen H, Yao L, Li Y, Zhao Q, Xiao M. Experience and support of Chinese healthcare professionals as second victims of patient safety incidents: A cross-sectional study. Perspectives in Psychiatric Care. 2022 Apr;58(2):733-43.		X
47	Zhang X, Li Q, Guo Y, Lee SY. From organisational support to second victim-related distress: Role of patient safety culture. Journal of Nursing Management. 2019 Nov;27(8):1818-25.	X	X
48	Cutler A, Black JD, Sheth SS, Pathy S. When Things Go Wrong: Examining the Frequency and Aftermath of Critical Incidents Among OBGYN Residents. Connecticut Medicine. 2017 May 1;81(5).	X	X
49	Biggs S, Waggett HB, Shabbir J. Impact of surgical complications on the operating surgeon. Colorectal Disease. 2020 Sep;22(9):1169-74.		X
50	Buhlmann M, Ewens B, Rashidi A. Moving on after critical incidents in health care: A qualitative study of the perspectives and experiences of second victims. Journal of Advanced Nursing. Sep 2022;78(9):2960-2972.	X	X
51	Chang HE, Jang H, Bak YI. Clinical nurses' recovery experiences after adverse events in South Korea: A qualitative study. Collegian. 2022;29(4):456-464.	X	X
52	Christoffersen L, Teigen J, Ronningstad C, Following-up midwives after adverse incidents: How front-line management practices help second victims. Midwifery. Jun 2020;85:102669	X	X
53	De Boer J, van Rikxoort S, Bakker AB, Smit BJ. Critical incidents among intensive care unit nurses and their need for support: explorative interviews. Nursing in Critical Care. Jul 2014;19(4):166-74.	X	X
54	Ferrùs L, Silvestre C, Olivera G, Mira JJ. Qualitative study about the experiences of colleagues of health professionals involved in an adverse event. Journal of Patient Safety. 2021 Jan 1;17(1):36-43.	X	X
55	Ganahl S, Knaus M, Wiesenhuetter I, Klemm V, Jabinger EM, Strametz R. Second Victims in Intensive Care—Emotional Stress and Traumatization of Intensive Care Nurses in Western Austria after Adverse Events during the Treatment of Patients. International Journal of Environmental Research and Public		X

	Health. 2022 Mar 18;19(6):3611.		
56	Kable A, Kelly B, Adams J. Effects of adverse events in health care on acute care nurses in an Australian context: A qualitative study. Nursing and Health Sciences Jun 2018;20(2):238-246	X	X
57	Lee W, Pyo J, Jang SG, Choi JE, Ock M. Experiences and responses of second victims of patient safety incidents in Korea: a qualitative study. BMC Health Serv Res Feb 06 2019;19(1):100.	X	X
58	Leferink E, Bos A, Heringa MP, van Rensen EL, Zwart DL. The need and availability of support systems for physicians involved in a serious adverse event. Journal of Hospital Administration. 2018;7(2):23-30.	X	X
59	Luk LA, Lee FK, Lam CS, So HY, Wong YY, Lui WS. Healthcare professional experiences of clinical incident in Hong Kong: a qualitative study. Risk Management and Healthcare Policy. 2021 Mar 8:947-57.	X	X
60	May N, Plews-Ogan M. The role of talking (and keeping silent) in physician coping with medical error: A Qualitative study. Patient Education and Counseling Sep 2012;88(3):449-454.		X
61	Mokhtari Z, Hosseini MA, Khankeh HR, Fallahi-Khoshknab M, Nasrabadi AN. Barriers to support nurses as second victim of medical errors: A qualitative study. Australasian Medical Journal 2018;11(12):556-560.		X
62	Pinto A, Faiz O, Bicknell C, Vincent C. Surgical complications and their implications for surgeons' well-being. British Journal of Surgery. Dec 2013;100(13):1748-55	X	X
63	Rinaldi C, Leigheb F, Vanhaecht K, Donnarumma C, Panella M. Becoming a “second victim” in health care: Pathway of recovery after adverse event. Revista de Calidad Asistencial. 2016 Jul 1;31:11-9.	X	X
64	Serou N, Husband AK, Forrest SP, Slight RD, Slight SP. Support for Healthcare Professionals After Surgical Patient Safety Incidents: A Qualitative Descriptive Study in 5 Teaching Hospitals. J Patient Saf Aug 01 2021;17(5):335-340.	X	X
65	Schrøder K, Janssens A, Hvidt EA. Adverse events as transitional markers-Using liminality to understand experiences of second victims. Social Science and Medicine. 2021 Jan 1;268:113598.	X	X
66	Tavares APM, Barlem JGT, Silveira RSD, Dalmolin GL, Feijó GDS, Machado IA, Paloski GDR, Abreu IM. Support provided to nursing students in the face of patient safety incidents: a qualitative study. Rev Bras Enferm 2022;75(2):e20220009.	X	X
67	Ullström S, Sachs MA, Hansson J, Øvretveit J, Brommels M. Suffering in silence: a qualitative study of second victims of	X	X

	adverse events. BMJ Quality & Safety. 2014 Apr 1;23(4):325-31.		
68	Van Gerven E, Deweer D, Scott SD, Panella M, Euwema M, Sermeus W, Vanhaecht K. Personal, situational and organizational aspects that influence the impact of patient safety incidents: a qualitative study. Revista de Calidad Asistencial. 2016 Jul 1;31:34-46.	X	X
69	Huang H, Chen J, Xiao M, Cao S, Zhao Q. Experiences and responses of nursing students as second victims of patient safety incidents in a clinical setting: A mixed-methods study. Journal of Nursing Management. 2020 Sep;28(6):1317-25.		X
70	Kobe C, Blouin S, Moltzan C, Koul R. The second victim phenomenon: perspective of Canadian radiation therapists. Journal of Medical Imaging and Radiation Sciences. 2019 Mar 1;50(1):87-97.	X	X
71	Kruper A, Domeyer-Klenske A, Treat R, Pilarski A, Kaljo K. Secondary traumatic stress in Ob-Gyn: A mixed methods analysis assessing physician impact and needs. Journal of Surgical Education. 2021 May 1;78(3):1024-34.	X	X
70	Kobe C, Blouin S, Moltzan C, Koul R. The second victim phenomenon: perspective of Canadian radiation therapists. Journal of Medical Imaging and Radiation Sciences. 2019 Mar 1;50(1):87-97.	X	X
71	Kruper A, Domeyer-Klenske A, Treat R, Pilarski A, Kaljo K. Secondary traumatic stress in Ob-Gyn: A mixed methods analysis assessing physician impact and needs. Journal of Surgical Education. 2021 May 1;78(3):1024-34.	X	X
72	Plews-Ogan M, May N, Owens J, Ardelt M, Shapiro J, Bell SK. Wisdom in Medicine: What Helps Physicians After a Medical Error? Academic Medicine. Feb 2016;91(2):233-41		X
	Total studies	52	57
