# Supplementary material for: Avoiding ‘second victims’ in healthcare: what support do staff want for coping with patient safety incidents, what do they get and is it effective? A systematic review
Source: BMJ Open. 2025 Feb 10;15(2):e087512. doi: 10.1136/bmjopen-2024-087512 (PMC12185930; doi:10.1136/bmjopen-2024-087512)
Supplement: Supplementary data [file bmjopen-15-2-s005.pdf]

Supplementary 5 File: Description of fourth and fifth (a) most wanted and (b) most frequently experienced forms of support (continued from main article)

(a) Continuation of most wanted forms of support

#4 most desired: Post-incident debrief to clarify and process what happened (n=18 studies):

Debriefs and team discussions with colleagues involved in the incident were wanted by some participants. However, it was not always clear how this was different to peer support, or whether it referred to an informal clinical debriefing or to more structured Critical Incident Stress Debriefing. Fourteen studies found that second victims wanted information about the incident, including an opportunity to explore any ethical issues, causes and preventative measures, and also to be involved in evaluation and learning around the incident which could contribute to post-traumatic growth.

#5 most desired: Supportive and learning-oriented culture (n=14 studies): Healthcare staff in many studies expressed their desire for a responsive organisational approach that supported them after a patient safety incident and a culture in which they felt protected, recognised and not blamed or criticised. In one study, participants ranked the promotion of an open reporting and learning culture as the most critically important form of support to aid recovery and avoid compounding psychological harm after involvement in a patient safety incident. Raising awareness of the impact of patient safety incidents/errors and preparing staff for involvement in such events, through courses and conferences within one's organisation, were also rated as highly desirable in one study.

(b) Continuation of most experienced forms of support

#4 most experienced: Professional mental health support (n=11): Counselling and other professional mental health support was also mentioned in multiple studies. However, this was experienced less often than other support options. In studies where organisations did not offer such services, participants reported seeking this help themselves (e.g. 40% of one sample; **63)**.

There were indications of stigma associated with this option, with almost a third of physicians (31%) in one mixed methods study (17) reporting feeling embarrassed about needing psychological support.

#5 most experienced: The Mortality and Morbidity meeting (n=7): The Mortality and Morbidity (M&M) meeting was referred to in several studies as a form of support after patient safety incidents. These were the joint second most commonly experienced form of support amongst studies that ranked strategies. Some challenges were noted with this meeting as a support approach, such as not all staff involved in an incident may be invited and the focus on logistics and learning may provide little space to discuss the emotional and professional impact.

Simms-Ellis R, *et al. BMJ Open* 2025; 15:e087512. doi: 10.1136/bmjopen-2024-087512
