# Supplementary material for: Avoiding ‘second victims’ in healthcare: what support do staff want for coping with patient safety incidents, what do they get and is it effective? A systematic review
Source: BMJ Open. 2025 Feb 10;15(2):e087512. doi: 10.1136/bmjopen-2024-087512 (PMC12185930; doi:10.1136/bmjopen-2024-087512)
Supplement: Supplementary data [file bmjopen-15-2-s006.pdf]

## Supplementary Table: Summary of included studies at Stage 2

| First author (reference), country | Intervention name | Intervention description | Study Design (Quality appraisal score) | Sample, Setting | Outcome measure related to effectiveness (measure used) | Outcomes |
|---|---|---|---|---|---|---|
| Johnson [59]<br><br>UK | *No name. 'Resilience coaching intervention to prepare healthcare professionals for stressful healthcare events, particularly adverse events'* | *A psychologist-delivered, Cognitive Behaviour Therapy (CBT)-based, half day workshop (psycho-educational didactic teaching, small group discussion and experiential exercises) followed by a one-hour, individual coaching phone call.* | *Uncontrolled before-after study; mixed methods - semi-structured interviews & questionnaires.*<br><br>*No comparison of users/non-users.*<br><br>*Four data collection points: baseline, after workshop, after coaching call, 4-6 weeks post baseline.*<br>*(32)* | *Midwives, paramedics, paediatric consultants. Trainee obstetrics & gynaecology doctors, trainee paediatric doctors. Physician Associate students, sonography & mammography students (n=66).*<br><br>*Hospital & University.* | *Confidence in coping with adverse events* (3-item, purpose designed measure);<br><br>*Knowledge about resilience, coping strategies, personal strengths* (5-item purpose designed measure T1 & T2 only);<br><br>*Resilience to cope with future adverse events* (Brief Resilience Scale [85]). | *Compared with T1 baseline, statistically significant increases post intervention in (1) Confidence at T2 (unadj. β = 2.43, 95% CI 2.08–2.79, d = 1.55, p < .001), T3 (unadj. β = 2.81, 95% CI 2.42–3.21, d = 1.71, p < .001) and T4 (unadj. β = 2.75, 95% CI 2.31–3.19, d = 1.52, p < .001; (2) Knowledge at T2 (unadj. β = 1.14, 95% CI 0.82–1.46, d = 0.86, p < .001); (3) Resilience at T3 (unadj. β = 2.77, 95% CI 1.82–3.73, d = 0.90, p < .001) and T4 (unadj. β = 2.54, 95% CI 1.45–3.62, d = 0.65, p < .00).* |
| Mira [60]<br><br>Spain | *Mitigating Impact in Second Victims (MISE) online program* | *A flexible online programme: 'informative' package (information and research about patient safety incidents and second victimhood) and 'demonstrative' package (videos showing what to do/not do after patient safety incident, covering emotional, family, work consequences of adverse events, how to inform patient, access support, support others, how to be prepared/ what to do).* | *Uncontrolled before and after study; quantitative.*<br><br>*Survey/test issued pre and post completion.*<br><br>*No comparison of users/non-users.*<br>*(23)* | *Multi-disciplinary: physicians and nurses (n=292)*<br><br>*Online.* | *Knowledge changes regarding:*<br><br>*(1) Informative Package of the website* (12-item purpose designed test completed pre & post intervention);<br><br>*(2) Demonstrative package of the website* (8-item purpose designed test completed pre & post intervention). | *Significant differences between pre- and post- tests results.*<br><br>*Test 1 Informative package: Pre-intervention (M=6.9, SD =2.0) and post-intervention (M=8.8, SD=1.6; t265=–10.0, P<.001).*<br><br>*Test 2 Demonstrative package: Pre-intervention (M=6.3, SD=1.5) and post-intervention (M=7.2, SD=1.0; t265=–6.2, P<.001).* |
| Musunur [61] | *When Bad Things Happen: Training* | *A one-hour education session delivered within the curriculum at medical* | *Uncontrolled before-after study; quantitative.* | *Pre-clerkship medical students (n=300).* | *(1) awareness of resources to cope with and report medical error;* | *Overall responses to education session (all 4 domains): significant difference between* |

| | | | | | | |
|---|---|---|---|---|---|---|
| USA | *Medical Students to Anticipate the Aftermath of Medical Errors* | *school (didactic presentation, video vignettes of seniors discussing error experiences, and guided small group discussion).* | *Survey/test issued pre and post education session, across 2 years – 2016-17.*<br><br>*No comparison of users/non-users.*<br>*(18)* | *University.* | *(2) confidence in ability to recognise & cope with medical error;*<br><br>*(3) knowledge of medical error leading to physician burnout;*<br><br>*(4) attitudes to error, including workplace culture, acknowledging responsibility*<br><br>(ALL: 14-item purpose designed pre and post intervention test/survey). | *post- and pre-session results in 2016 (p<0.001; by 0.34 units of mean change, SD=0.35 units) and 2017 (p<0.001, by 0.33 units of mean change, SD=0.03 units).*<br>*Responses by domain: Significant changes in pre and post session results for (1) awareness of resources and (2) confidence in coping with error - in 2016 and 2017. No significant changes in either 2016 or 2017 in pre and post results for (3) knowledge of medical error or (4) attitudes to error.* |
| Connors [62]<br><br>USA | *RISE program (Resilience in Stressful Events)* | *Peer support programme. Peers, trained in psychological first aid, respond to self- or colleague-referrals within 30 minutes of receiving notification, 24 hours a day, seven days a week. Peers are clinically-based, but tend not to be known to callers, and focus on how the staff member is feeling rather than event details, supporting them to identify coping strategies to aid recovery – usually in person but sometimes by phone.* | *Cross sectional survey; mixed methods – open and fixed response questionnaire.*<br><br>*Compared users/non-users.*<br>*(24)* | *Nurses and nurse leaders (n=412).*<br><br>*Hospital.* | *(1) Professional burnout* (5 items adapted from Emotional Exhaustion domain of Maslach Burnout Inventory [86]);<br><br>*(2) Job satisfaction* (3 items from previously validated questionnaire [87])*;*<br><br>*(3) Personal resilience* (2 of 4 items from Resilience domain in Second Victim Experience & Support Tool (SVEST) [88]). | *<u>Staff Nurses:</u> No significant differences between RISE users/non-users in (2) Job satisfaction (67% vs. 61%; p=0.45). Significant differences in burnout & resilience: (1) Burnout - more RISE users than non-users reported some level of burnout (93% vs 67%, p=0.038). Odds of indicating some level of burnout were 4.3 times greater for recent RISE-users than non-users (p=0.048). (3) Resilience: RISE users (past year) significantly more likely to report resilience (57% vs 36%, p=0.025).*<br>*<u>Nurse Leaders:</u> No significant differences between RISE activators/non-activators in: (1) burnout (40% activators vs 50% non-activators reported at least some burnout; p=0.42) and (2) job satisfaction (75% activators vs. 85% non-activators; p=0.315). Significant differences in (3) Resilience: Non-activators were* |

| | | | | | | |
|---|---|---|---|---|---|---|
| | | | | | | *more resilient than activators (89% vs 50%, p=0.001). Odds of indicating strong resilience were 0.12 times lower for activators than never-activators (p=0.002).* |
| Finney [63]<br><br>USA | *HELP (Healing Emotional Lives of Peers) program* | *Peer support programme. Based on/ informed by the University of Missouri Health Care 'forYOU' model [40]. Provides emotional support to individuals and groups, 24 hours a day, seven days a week. Grounded in Scott's three-tiered interventional model of support. Adaptations: peer supporters trained in all departments & job levels, embedding in every team, not just high-risk areas.* | *Uncontrolled before and after study: quantitative - Full department survey.*<br><br>*No comparison of users/non-users.*<br>*(26)* | *Paediatric healthcare professionals (n=371 [pre-194/373; post-177/403]).*<br><br>*Hospital Paediatric inpatient unit, ICU* | *Impact of programme on Second Victim experience, encompassing: psychological distress; physical distress; colleague support; supervisor support; institutional support; non-work support; professional self-efficacy; absenteeism; turnover intentions* (ALL: SVEST [88]). | *One-year post intervention launch, compared to pre-intervention: Significantly fewer staff agreed institutional support was 'inadequate' (10.9% vs. 0.6%, p<0.001); Significantly fewer reported turnover intentions (18.2% vs 9.2%, p=0.014 (n.b. in table 2 this is cited as p=0.09); No significant differences on any other SVEST dimensions.* |
| Klatt [64]<br><br>USA | *Supporting Our Staff (SOS) Peer Support Program* | *Peer support programme. Based on/informed by the University of Missouri Health Care 'forYOU' model [40]. Adaptations: (a) peer supporters trained in all departments and job levels, embedding in every team, not just high-risk areas.* | *Uncontrolled before-after study: quantitative – organisational survey.*<br><br>*Planned a baseline survey pre-launch but unable to do so. Issued 3&9 months post-launch.*<br><br>*No comparison of users/non-users: compared SVEST responses of those who reported experiencing a recent adverse event at 3- and 9-months.*<br>*(26)* | *Multi-disciplinary (n=unclear).*<br><br>*All clinical areas, inpatient and outpatient settings, hospital quaternary care campus.* | *Impact of program on Second victim experience, encompassing: psychological distress; physical distress; colleague support; supervisor support; institutional support; non-work support; professional self-efficacy; absenteeism; turnover intentions* (ALL: SVEST [88]). | *3 months post-launch, SVEST issued to 6,109 HCPs; n=1,786 responses. At 9 months post-launch, n=1,188 responses. Authors compared responses of those involved in adverse outcome/error in past 3 years at 3- (n=766) and 9-months (n=549).*<br>*No significant differences identified on any SVEST dimension.* |
| Moran [65]<br><br>USA | *Resilience in Stressful Events (RISE) program* | *Peer support programme. Peers, trained in psychological first aid, respond to self- or colleague-referrals within* | *Cross sectional survey and Markov modelling to identify cost-benefits of a hospital having versus not having RISE; quantitative.* | *Nurses (n=36).*<br><br>*Hospital.* | *Net monetary benefit & budget impact of having RISE.*<br>*Cost of running RISE program (*divided total financial cost to hospital of program by number of | *Cost of RISE = $656.25 per nurse user.*<br>*After RISE, decrease in turnover & sickness absence per nurse = $23,232.30.* |

| | | | | | | |
|---|---|---|---|---|---|---|
| | | *30 minutes of receiving notification, 24 hours a day, seven days a week. Peers are clinically-based, but tend not to be known to callers, and focus on how the staff member is feeling rather than event details, supporting them to identify coping strategies to aid recovery – usually in person but sometimes by phone.* | *(23)* | | nurse users in 2015 (n=80) to get average cost per nurse*). Hospital costs for nurse turnover (*literature review) and *nurse sickness absence after involvement in patient safety incident (*hospital's Human Resources data).<br><br>*Probability of resigning and taking a sick day with/without RISE (*13-item purpose designed survey requesting estimations of outcomes from 2 hypothetical patient safety incident scenarios, e.g. How many days off do you think this nurse would take? How likely is it they would need mental health services or quit?). | *Net monetary benefit saving of having the RISE program after 1 year = $22,576.05 per nurse using RISE.*<br><br>*RISE could save a hospital $1.81 million per year. Having RISE provided a positive net monetary benefit compared to not having RISE in 49,987 of the 50,000 (>99%) Monte Carlo simulations, at a mean net monetary benefit $23,360 (95% confidence interval, $3006 - $161,278).* |
| Morris [66]<br><br>Australia | *'Always There' peer support program* | *Peer support programme. Based on/ informed by the University of Missouri Health Care 'forYOU' model [40]. Adaptations: (a) peer supporters trained in all departments and job levels, embedding in every team, not just high-risk areas; (b) slightly reconceptualised Tiers 1 and 2: Tier 1 a 'warm response' - for staff experiencing cumulative/ acute stress; Tier 2 a 'hot response' – practical and emotional support for individuals or teams after a traumatic adverse event (e.g., suicide attempt). Part of broader just culture transformation.* | *Uncontrolled before-after study: quantitative – organisation-wide survey.*<br><br>*Compared users/non-users – 2 years post-launch.*<br>*(19)* | *Clinical and non-clinical staff (n=unclear).*<br><br>*ER, inpatient and community settings, large public mental health service.* | *Second victim experiences of those engaging with program, encompassing: psychological distress; physical distress; colleague support; supervisor support; institutional support; non-work support; professional self-efficacy; absenteeism; turnover intentions* (Second Victim Experience and Support Tool [88], used within 'Voices of the Staff' survey). | *Compared survey results pre and post launch of engagers (n=38) and non-engagers (n=232). No significant differences between groups in all but one SVEST dimensions. Significant differences identified between groups in 2 out of 3 items in 'institutional support' dimension (i.e., not for the full dimension): item 18 "my organisation understands the needs of staff in the aftermath of critical incidents" (81.6% of users vs 57.8% of non-users; p=.005) and item 19 "provides more resources to assist with recovery" (78.9% users vs. 59.9% non-users; p=.024).* |
| Rivera-Chiauzzi [67] | *No name: 'Structured peer support program'.* | *Peer support programme. Based on/ informed by the University of Missouri Health Care 'forYOU'* | *Randomised trial; quantitative – departmental survey.* | *Physicians, Nurses, Physician Assistants (n=42: 23 intervention* | *To develop & evaluate peer support program. Primary outcome: use of resources & perceptions of* | <u>*Effect on recovery stage:*</u> ***At baseline:*** *Most participants - 28/42 (13/23 enhanced; 15/19 routine) - were at Stage 6* |

| | | | | | | |
|---|---|---|---|---|---|---|
| USA | | *model [40]. 4 peer supporters attended 'Second Victim train the trainer workshop' by forYOU team. Trained peers then contacted all staff involved in an adverse event within one week, had in-person, one-to-one confidential chat to assess wellbeing, provide information on second victim recovery stages and EAP. Peer supporters phoned staff randomised to 'Enhanced' group one week later, then monthly up to 6 months until no longer needed.* | *3 timepoints: baseline, 3 months and 6 months post intervention.*<br><br>*Compared users/non-users.*<br>*(20)* | *['enhanced care']; 19 control ['routine care']).*<br><br>*Obstetric and maternal foetal medicine department, Hospital.* | *helpfulness.*<br>*Secondary outcome: effect on recovery stage after PSI and duration of support use (*ALL: Survey, though unclear if questionnaire was adapted from forYOU toolkit or purpose designed. Included demographics, use of informal resources, perceived helpfulness, barriers, current recovery stage*).* | *'Thriving'; 14/42 were at an earlier recovery stage: Stage 1-3 n=7 (enhanced 6/7; routine 1/7); Stage 4-6 [excluding those 'thriving'] n=7 (enhanced 4/7; routine 3/7).* ***At 6 months:*** *100% of participants, regardless of group, reported that they were 'thriving'.*<br><br>***Enhanced group:*** *Most needed less than 3 months' support. 62.5% declined follow-up after first contact; 91.3% declined follow-up after second contact. Significantly more likely than routine group to consider departmental leadership as one of the most helpful resources (p=0.02).* |
| Thompson [68] | *CRNA (Certified registered nurse anesthetists) Peer support Program* | *Peer support programme. Does not report being based on previously established models. 8 volunteers trained to provide peer support 24 hours a day. Support could be activated through self-, colleague- or lead CRNA-referral, or by the peer supporter noticing a colleague's distress.* | *Uncontrolled before and after study: quantitative – departmental survey.*<br><br>*Issued pre- and one-month after trial period to all CRNAs.*<br><br>*No comparison of users/non-users.*<br>*(22)* | *CRNAs. (n=83 [pre-n=51; post-n=32]).*<br><br>*CRNA Department, large academic medical centre.* | *Impact of second victim peer support protocol, compared to current post-adverse event hospital practices, on psychological distress; physical distress; colleague support; supervisor support; institutional support; non-work support; professional self-efficacy; absenteeism; turnover intentions* (SVEST [88]). | *No statistically significant differences were found between SVEST scores pre and post intervention (p>.05).* |
| Wijaya [69]<br><br>Indonesia | *No name 'second victim support program'* | *Peer support programme. Based on/ informed by the University of Missouri Health Care 'forYOU' model [40].* | *Controlled before and after study: quantitative – hospital-wide survey.*<br><br>*Issued: pre-launch; up to 1 year post launch; 1-2 years post-launch.*<br><br>*Compared users/non-users.*<br>*(17)* | *Doctors and nurses. (n=unclear. Unsure if exactly the same 87 participants surveyed across phases)*<br><br>*Hospital.* | *Perceptions of patient safety culture within the hospital* (Hospital Survey on Patient Safety Culture (HSOPSC) [89]). | *Phase 1: N=47 (/87; 54% response); Phase 2: N=60 (/same 87; 69% response); Phase 3: N=65 (/same 87; 75% response rate).*<br>*T1-T2: All HSOPSC ratings increased: Significant increase in patient safety culture (t=4.284, p=0.001, Cohen's d 0.854) large effect size). Compared to control hospital, significant increase in patient* |

| |
|---|
| *safety culture within intervention hospital (DID coefficient 0.738, SE= 0.258, p= 0.007). No significant differences at intervention hospital results between T 2 & T3 indicating sustained effect (t=0.378, p=0.713, Cohen's d 0.0476).* |
