# Supplementary material for: Avoiding ‘second victims’ in healthcare: what support do staff want for coping with patient safety incidents, what do they get and is it effective? A systematic review
Source: BMJ Open. 2025 Feb 10;15(2):e087512. doi: 10.1136/bmjopen-2024-087512 (PMC12185930; doi:10.1136/bmjopen-2024-087512)
Supplement: Author's manuscript [file bmjopen-2024-087512.draft_revisions.pdf]

BMJ Open is committed to open peer review. As part of this commitment we make the peer review history of every article we publish publicly available.

When an article is published we post the peer reviewers' comments and the authors' responses online. We also post the versions of the paper that were used during peer review. These are the versions that the peer review comments apply to.

The versions of the paper that follow are the versions that were submitted during the peer review process. They are not the versions of record or the final published versions. They should not be cited or distributed as the published version of this manuscript.

BMJ Open is an open access journal and the full, final, typeset and author-corrected version of record of the manuscript is available on our site with no access controls, subscription charges or pay-per-view fees (http://bmjopen.bmj.com).

If you have any questions on BMJ Open's open peer review process please email info.bmjopen@bmj.com

BMJ Open

Avoiding 'second victims' in healthcare: What support do staff want for coping with patient safety incidents, what do they get, and is it effective? A systematic review.

Journal:	*BMJ Open*
Manuscript ID	bmjopen-2024-087512
Article Type:	Original research
Date Submitted by the Author:	15-Apr-2024
Complete List of Authors:	Simms-Ellis, Ruth; Bradford Institute for Health Research Yorkshire Quality and Safety Research Group; University of Leeds<br>Sattar, Raabia; Bradford Institute for Health Research Yorkshire Quality and Safety Research Group<br>Sweeting, Elizabeth; Bradford Institute for Health Research, Improvement Academy<br>Hartley, Hannah; Bradford Institute for Health Research Yorkshire Quality and Safety Research Group<br>Morys-Edge, Matthew; University of Leeds<br>Harrison, Reema; Macquarie University, Australian Institute of Health Innovation<br>Lawton, Rebecca; University of Leeds, School of Psychology; Bradford Institute for Health Research Yorkshire Quality and Safety Research Group
Keywords:	Occupational Stress, Health Workforce, MENTAL HEALTH, OCCUPATIONAL & INDUSTRIAL MEDICINE, Organisational development < HEALTH SERVICES ADMINISTRATION & MANAGEMENT

SCHOLARONE™
Manuscripts

BMJ

I, the Submitting Author has the right to grant and does grant on behalf of all authors of the Work (as defined in the below author licence), an exclusive licence and/or a non-exclusive licence for contributions from authors who are: i) UK Crown employees; ii) where BMJ has agreed a CC-BY licence shall apply, and/or iii) in accordance with the terms applicable for US Federal Government officers or employees acting as part of their official duties; on a worldwide, perpetual, irrevocable, royalty-free basis to BMJ Publishing Group Ltd ("BMJ") its licensees and where the relevant Journal is co-owned by BMJ to the co-owners of the Journal, to publish the Work in this journal and any other BMJ products and to exploit all rights, as set out in our licence.

The Submitting Author accepts and understands that any supply made under these terms is made by BMJ to the Submitting Author unless you are acting as an employee on behalf of your employer or a postgraduate student of an affiliated institution which is paying any applicable article publishing charge ("APC") for Open Access articles. Where the Submitting Author wishes to make the Work available on an Open Access basis (and intends to pay the relevant APC), the terms of reuse of such Open Access shall be governed by a Creative Commons licence – details of these licences and which Creative Commons licence will apply to this Work are set out in our licence referred to above.

Other than as permitted in any relevant BMJ Author's Self Archiving Policies, I confirm this Work has not been accepted for publication elsewhere, is not being considered for publication elsewhere and does not duplicate material already published. I confirm all authors consent to publication of this Work and authorise the granting of this licence.

For peer review only

Avoiding 'second victims' in healthcare: What support do staff want for coping with patient safety incidents, what do they get, and is it effective? A systematic review.

Ruth Simms-Ellis [1,2*], Reema Harrison[3], Raabia Sattar[1], Elizabeth Sweeting[4], Hannah Hartley[1], Matthew Morys-Edge[2], Rebecca Lawton[1,2].

Affiliations

[1] Yorkshire Quality and Safety Research Group, Bradford Institute for Health Research, Bradford Royal Infirmary, Bradford, United Kingdom.

[2] School of Psychology, University of Leeds, Leeds, United Kingdom.

[3]Australian Institute of Health Innovation, Macquarie University, Sydney, Australia.

[4] Improvement Academy, Bradford Institute for Health Research, Bradford Royal Infirmary, Bradford, United Kingdom.

* Corresponding author.

Email: r.simms-ellis@leeds.ac.uk (RSE)

For peer review only

ABSTRACT

Objectives: Incontrovertible evidence surrounds the need to support healthcare professionals after patient safety incidents (PSI). However, what characterises effective organisational support is less clearly understood and defined. This review aims to determine what support healthcare professionals want for coping with PSIs, what support interventions/approaches are currently available and which have evidence for effectiveness.

Design: Systematic research review with narrative synthesis. PROSPERO registration number: CRD42022325796 [https://www.crd.york.ac.uk/PROSPERO/display_record.php?RecordID=325796]

Data Sources: Medline, Scopus, PubMed and Web of Science were searched (from 2010-mid 2021; updated December 2022), reference lists of eligible articles and Connected Papers software.

Eligibility Criteria for Selecting Studies: Empirical studies (1) containing information about support frontline healthcare staff want before/after a PSI, OR addressing (2) support currently available, OR (3) the effectiveness of support to help prevent/alleviate consequences of a PSI. Study quality was appraised using the Quality Assessment for Diverse Studies tool.

Results: Ninety-nine studies were identified. Staff most wanted: peer support (n=28), practical support and guidance (n=27) and professional mental health support (n=21). They mostly received: peer support (n=46), managerial support (n=23) and some form of debrief (n=15). Reports of poor PSI support were common. Eleven studies examined intervention effectiveness. Evidence was positive for the effectiveness of preventive/preparatory interventions (n=3), but mixed for peer support programmes designed to alleviate harmful consequences after PSIs (n=8). Study quality varied.

Conclusions: Beyond peer support, organisational support for PSIs appears to be mis-aligned

with staff desires. Gaps exist in providing preparatory/preventive interventions and practical support and guidance. Reliable effectiveness data are lacking. Very few studies incorporated comparison groups or randomisation; most used self-report measures. Despite inconclusive evidence, formal peer support programmes dominate. This review illustrates a critical need to fund robust PSI-related intervention effectiveness studies to provide organisations with the evidence they need to make informed decisions when building PSI support programmes.

Key Words: health personnel, medical errors, occupational stress, occupational health services, organisational culture.

ARTICLE SUMMARY

Strengths and limitations of this study

- Applying a respected multi-level occupational health stress prevention model to patient safety incidents and using this model to map second victim support requirements and organisational support provision were strengths.
- The specific targeting of intervention effectiveness was a strength.
- Given the complexity of the research area, it is possible that our search strategy omitted eligible studies.

FUNDING

The research was supported by the National Institute for Health and Care Research (NIHR) Yorkshire and Humber Patient Safety Translational Research Centre and Patient Safety Research Collaboration. The views expressed are those of the authors, and not necessarily those of the NIHR or the Department of Health and Social Care.

CONFLICTS OF INTEREST

For peer review only

All authors have completed the ICMJE uniform disclosure form at http://www.icmje.org/disclosure-of-interest/ and declare: all authors (except RH) were supported by the National Institute for Health and Care Research (NIHR) Yorkshire and Humber Patient Safety Translational Research Centre and Patient Safety Research Collaboration*;* no financial relationships with any organisations that might have an interest in the submitted work in the previous three years; no other relationships or activities that could appear to have influenced the submitted work.

INTRODUCTION

The international healthcare workforce is in crisis [1] with an estimated global deficit of 9.9 million nurses, midwives and physicians by 2030 [2]. High turnover is a major contributor [3, 4] and this has accelerated during and since the COVID-19 pandemic [5]. Being involved in a 'patient safety incident' is a significant factor in healthcare staff turnover [6-8]: around 12% of healthcare professionals surveyed in one study had left their job after a patient safety incident [9].

Patient safety incidents (PSIs) are unintended or unexpected incidents, which harmed or could have harmed one or more patients receiving healthcare [10]. PSIs occur in around 10% [11] to 14% [12] of patient admissions in hospital and in 2%-3% of patient visits in primary care [13, 14]. Being involved in harming or almost harming a patient can be traumatising for healthcare professionals, who typically enter healthcare to help others and uphold the Hippocratic oath of 'first do not harm' [15]. This human distress is frequently compounded and intensified by harsh retributive blame culture [16-21], a judgemental or critical response from colleagues and supervisors, inadequate support in disclosing what happened to the patient/family, the subsequent investigation process [22] and fears about litigation/malpractice [15].

For peer review only

The first priority after any PSI is to care for the patient and family, as the primary victim. However, there is incontrovertible evidence that healthcare professionals involved in such incidents also require their organisation's support as the silent 'second victims' [23]. With a high number of healthcare professionals across different roles and countries reporting having experienced the second victim phenomenon at some point in their career (recent surveys range from 45% of 205 US obstetrics and gynaecology workers [24] to 91% of 658 Asian surgeons [25]), the scale and universality of this issue is significant.

The potential, harmful impact of PSIs on individual healthcare professionals and healthcare organisations has been well documented through over 20 years of international research into the second victim phenomenon [6, 7, 15, 16, 26-35]. Individuals consistently describe experiencing a range of emotional, cognitive, behavioural and physiological symptoms (see Table 1). Many of these symptoms are commensurate with a clinical diagnosis for PTSD [36] and burnout, which is significantly associated with involvement in a PSI [34, 37]. Impacts on professional functioning include an impaired ability to manage patients in the aftermath of a PSI, reduced confidence, self-doubts, fears about litigation, losing one's job [21, 34], defensive changes in practice, such as avoiding procedures, secrecy/cover ups and ordering up to 30% more tests than necessary [38]. The organisational impact of PSIs include compromised team functioning, patient safety and care quality [34], increased costs from defensive practice and reluctance to report future incidents [38], absenteeism, turnover and the loss of highly trained staff [6].

Table 1: Four symptom groups associated with the 'second victim' experience

Symptom Group	Individual Symptoms
Emotional	Shame; Guilt; Anxiety; Anger; Shock; Depression; Fear.
Cognitive	Flashbacks; Intrusive thoughts and nightmares; Re-imagining; Ruminating on details; Helplessness; Suicidal ideation.

For peer review only

Physiological	Fatigue; Sleep disturbance; Nausea; Headaches; Gastro-intestinal issues.
Behavioural	Hypervigilance; Withdrawal; Irritability; Social avoidance; Emotional numbing; Poor concentration; Diminished memory; Avoidance of triggers.

Whilst there is incontrovertible evidence for the need to support healthcare professionals with PSIs, less clarity surrounds what type of support they actually want and tend to receive, or what characterises an ‘effective’ support intervention [39]. A range of interventions, methods and strategies to support second victims has been identified as available through multiple reviews including scoping [40, 41], literature [42, 43], narrative [44] and integrative [39, 45]. These largely sought to identify and describe second victim support interventions, including any supporting data regarding acceptability, perceived helpfulness, feasibility, implementation challenges, and, in some cases [e.g., 41, 43, 45], outcomes or ‘effectiveness’. Included studies have varied in nature, from descriptions of programme development and peer training workshops to explorations of risk managers’ perceptions of second victim programmes and implementation/usage statistics.

To our knowledge, there has been only one *systematic* review of support resources for second victims [46]. This features 16 studies that report on 12 second victim interventions between 2007 and 2017, and focuses on providing ‘a descriptive overview of second victim support resources’, including ‘their conceptual basis’, benefits for users, peer supporter perceptions and experiences and challenges during implementation. Collectively, these reviews provide extensive coverage of the range of support interventions available internationally. However, there remains a gap in the literature regarding what actually ‘works’ when it comes to supporting staff through PSIs. There is a need to understand, comprehensively and systematically, what type of support healthcare staff want, whether

For peer review only

For peer review only - http://bmjopen.bmj.com/site/about/guidelines.xhtml

organisations are providing support that is both aligned to their 'wants' (a key factor in the uptake of wellbeing interventions [47]) and, critically, effective.

A model for supporting healthcare professionals *after* involvement in PSIs was developed inductively by Scott and colleagues' [48], featuring three tiers: local support from leaders and colleagues; wellbeing support from trained peers and patient safety colleagues; a referral network for staff requiring more help. A recent review [44] has argued for the expansion of this model, based on its findings, to incorporate a 'prevention' tier, to help minimise the impact of PSIs. This is supported by robust evidence within the occupational health psychology literature that the most effective institutional support strategy is multi-levelled and firmly rooted in prevention [49-51]. A multi-level, preventive approach to stress management focuses on minimising the stressors that compromise employee wellbeing (primary level), moderating individuals' reactions when exposed to stressors (secondary level) and providing individualised support to those experiencing strain (tertiary level).

In this systematic review, we used a widely-respected, multi-level stress prevention model [49] to map the empirical evidence surrounding second victim support. Specifically, we aimed to answer the following research questions:

1. What support do healthcare staff want before and after a PSI?
2. What support is currently available to these staff?
3. How effective are existing support mechanisms/interventions designed to: (a) prevent poor psychological outcomes for healthcare staff after PSIs (primary-level); (b) support staff in the aftermath of PSIs (secondary-level); offer psychological support for those affected by involvement in PSIs (tertiary-level).

METHODS

Design

For peer review only - http://bmjopen.bmj.com/site/about/guidelines.xhtml

We used systematic review with narrative synthesis [52] and followed the Preferred Reporting Items for Systematic Reviews and Meta analyses [53] (for PRISMA checklist, see S1 File). The protocol was registered with the International Prospective Register of Systematic Reviews (PROSPERO; CRD42022325796).

Eligibility Criteria

Studies were eligible if they were empirical, published in English between 2010 and 2022 and contained information within any healthcare context about the support frontline healthcare staff want before or after a patient safety incident, OR the support currently available, OR the effectiveness of interventions to help prevent or mitigate second victim phenomena. Articles were excluded if they were grey literature, study protocols, opinion pieces, systematic reviews, meta-analyses, theses, focused on general staff wellbeing, stressful non-patient safety incidents or reducing, or increasing the reporting of, medical errors.

Study identification

Drawing upon search terms used in our recent, related systematic review [31] and with support from a medical information specialist, synonyms and relevant concepts were developed for the two major concepts being explored: second victim and emotional support. A search strategy was developed iteratively and applied to the following electronic databases in June 2021 and updated in December 2022: Medline, Scopus, PubMed and Web of Science. For an example of full search terms used for Medline, see S2 File. Reference lists of eligible articles were also searched and Connected Papers software tool (https://www.connectedpapers.com/) was used to identify additional studies. Covidence systematic review software (Veritas Health Innovation, Melbourne, Australia) was used for study screening and management.

For peer review only - http://bmjopen.bmj.com/site/about/guidelines.xhtml

Study selection and data extraction

After a preliminary scan of the eligible articles, we determined that a two-stage process was required for the review: stage 1 would address research questions 1 and 2; stage 2 to address research question 3. This represented a deviation from our published protocol. Using the eligibility criteria, five of the authors (RS, RL, RS-E, JH, LS) screened first titles and abstracts, then full texts for each potentially relevant study. Regular weekly meetings were held during this period to discuss screening decisions and disagreements, finalise eligible articles for inclusion and undertake a face validity check of the final set of studies. A PRISMA diagram was produced to evidence decisions made (Fig. 1).

Studies were selected if they could answer research question 1, 2, 3 or a combination and coded within Covidence to denote inclusion at stage 1 or stage 2. All authors extracted the following information from the included studies: author, publication year, country, study design, setting, sample, and key findings. In addition, all articles featuring individual interventions were categorised according to the level of organisational support offered, i.e., primary, secondary or tertiary.

At Stage 1, due to the large number of studies, we limited the extraction of 'key findings' to what healthcare staff said they wanted and what support they received. At Stage 2, the extraction of 'key findings' was more comprehensive to allow us to examine effectiveness of specific interventions. We focused on outcomes, not feasibility or acceptability, therefore we did not extract any data regarding user acceptability and intervention usage statistics. Additional 'intervention description' data were also extracted at Stage 2, using the Template for Intervention Description and Replication (TIDieR) criteria [54]. At both stages, data extraction was conducted by all reviewers, with regular discussions to resolve disagreements. A random 20% sample of intervention effectiveness studies included in Stage 2 was double checked independently by a second reviewer (JH).

For peer review only - http://bmjopen.bmj.com/site/about/guidelines.xhtml

Assessment of study quality

Only studies included at Stage 2 were assessed for methodological quality. This was conducted independently by two reviewers (RS, HH) using the 13-item quality assessment tool QuADS [55], selected for its appropriateness for study samples with different methodological approaches.

Data synthesis

In this systematic review, we used an established multi-level stress prevention framework to help us organise and map the empirical evidence available. We adapted Cooper and Cartwright's [49] intervention strategy for general workplace stress to address the specific and unique workplace stressor/hazard of being involved in a patient safety incident as a healthcare professional in a healthcare organisation (Fig. 2; https://secondvictim.co.uk/supporting-second-victims/#why-support-second-victims).

Insert Figure 2 here

Figure 2: *Organisational staff support model for Patient Safety Incidents*

Data at both stages were synthesised according to each research question using narrative synthesis. In addition, each form of support was coded according to the level it targeted: primary (i.e., preventive, proactive), secondary (i.e., aiding natural coping after an incident) or tertiary (i.e., specialist one-to-one remedial support).

At stage 1, where a study reported support in ranked order of preference or most frequently received, we focused on the top three supports in each case. Where rankings were not available, such as in qualitative studies, we focused on the support most frequently reported as preferred and received. At stage 2, an initial description of the intervention effectiveness studies' results was produced, including descriptive statistics and an assessment of study quality. A narrative synthesis was conducted, where studies were organised to

For peer review only - http://bmjopen.bmj.com/site/about/guidelines.xhtml

explore the effectiveness of available support in helping to prevent or alleviate the anticipated or actual psychological consequences of a patient safety incident. Specifically, each intervention was coded as primary-, secondary- or tertiary-level support to create three groups of studies. Each group was reviewed and synthesised separately, before completing a multi-level, holistic examination and synthesis. Study information was systematically tabulated and patterns within and between studies were explored to identify commonalities/differences and potential reasons for these.

RESULTS

Results of the search

We retrieved 4160 articles, 99 of which were included for data extraction (Fig. 1). All 99 studies were included at stage 1. Only 11 [56-66] presented outcome data and purported to assess effectiveness, therefore were subsequently also included in Stage 2.

Insert Figure 1 here

Figure 1: *PRISMA flowchart of stages in the review*

Excluded studies

Studies were predominantly excluded at full text review because they were not empirical (n=39), they did not focus on the impact of PSIs on staff (n=39), or they did not aim to assess staff support interventions for PSIs, healthcare staff perceptions of PSI support, or the effect of PSI support on staff/organisational culture outcomes (n=38). Several exclusions were also made for studies that did not include frontline healthcare staff as participants (n=17), or examined 'organisational support' more broadly as a mediator for organisational outcomes (n=9).

For peer review only - http://bmjopen.bmj.com/site/about/guidelines.xhtml

Characteristics of included studies

Included studies were primarily from the United States (n=41), Europe (n=33), East and South-East Asia (n=13) and Australasia (n=6). Studies were quantitative (n=52), qualitative (n=22) and mixed methods (n=25). The cross-sectional survey dominated (n=73); those with a qualitative component mostly used semi-structured interviews (n=23), focus/online discussion, action groups or both (n=10). Samples were most often multi-disciplinary (n=53) with others featuring uni-disciplinary groups (n=46), which were mostly of physicians (n=22), nurses (n=16). Specialties represented included obstetrics and gynaecology or midwifery (n=13), surgery (n=10), anaesthesiology (n=7), paediatrics (n=5) and intensive/critical care (n=5), psychiatry and mental health (n=3), pharmacy (n=1), radiotherapy (n=1), acute care (n=1), cardiology (n=1), urology (n=1) and internal medicine (n=1). Sample sizes ranged from 8 participants to 3,766 (based on n=92 studies: n=7 sample sizes were not clear).

Stage 1 Review Findings

What support do staff want before and after a PSI? (Research Question 1); What support is currently available to healthcare staff? (Research Question 2).

Seventy-two studies investigated what staff wanted (n=15), received (n=20) or both (n=37). Thirty-five different forms of 'desired' and 11 different types of 'experienced' support were identified. Due to the scale of the data, a decision was made to focus on the top 5 most highly rated and experienced forms of support (see supplemental Table), however a detailed narrative summary of findings and references can be found in S3 File. The top 3 for most desired and most experienced support are described below (see S4 File for top 4 and 5).

The top 3 most desired forms of support (Research Question 1)

For peer review only - http://bmjopen.bmj.com/site/about/guidelines.xhtml

#1 Peer support (n=28 studies): Peer support was the most desired form of support in every study that presented rankings. Specifically valued were peers sharing their own mistakes, checking in regularly and providing practical advice and reassurance about their professional capability to help restore confidence. However, the definition of 'peer support' differed between studies, from 'a respected peer to discuss the details of what happened', 'emotional support from colleagues', 'colleagues taking time to listen or help with workload' to 'conversations with peers'. Differences were also apparent in who second victims wanted peer support from. Some preferred 'informal peer support', from non-judgemental, empathic colleagues from their direct work environment, who fully understood the nature of the job and the incident itself. Others, wanted to speak with the whole team and some physicians wanted to talk only with other physicians.

#2 Practical support and guidance (n=27 studies): Preferences for practical support after a patient safety incident featured heavily across the sample, including rest and recuperation opportunities, temporary work adjustments and technical and procedural guidance. Having a specified peaceful location for recovery and regaining composure appeared as a top 3 most desired form of support in the second highest number of studies. A desire for temporary job adaptations and accommodation was also evident, such as being freed from workloads, having a review of one's responsibilities and being allowed to complete tasks and responsibilities with a colleague. Time away from work was also commonly desired, or a period of leave to help distance oneself and recover from the event. All these were desires for staff to initiate themselves and discuss with managers, rather than being instructed by managers punitively due to loss of trust after a PSI. Numerous studies found that second victims wanted technical guidance and advice with procedures surrounding investigations, legal aspects and support in communicating with the affected patient or family.

For peer review only - http://bmjopen.bmj.com/site/about/guidelines.xhtml

#3 Professional mental health support (n=21 studies): Specialist mental health support or counselling, including access to Employee Assistance Programmes (EAPs), was highly desired: of the studies that ranked support strategies, counselling was rated in the top 3 by the joint third most studies. Staff across several studies wanted confidential counselling to be provided in-house by the organisation, whilst a similar number wanted professional help offsite, through EAPs. In some studies, healthcare staff wanted formal support by a mental health professional, whilst in others, staff wanted an opportunity to *access* this if needed. Ambivalence was also sometimes evident, with concerns about whether this would be acceptable and utilised, particularly by surgical teams.

The top 3 most experienced forms of support (RQ2)

#1 Peer support (n=46 studies): Peer support was the most frequently experienced form of support, ranking most common in studies that ranked support frequency. Healthcare staff gained support speaking to direct colleagues, especially those at a similar grade and those involved in the incident. However, some ambiguity surrounds the way peer support was discussed across the sample. Often insufficient detail is detail is available to consistently identify and define peer support: some studies imply it is facilitated by the organisation in some structured, formal way; others imply it is more informal, talking to colleagues in one's team.

#2 Managerial support (n=23 studies): After peer support, support from supervisors, managers and department leaders was the next most frequently experienced form of support, according to included surveys. Qualitative and mixed methods studies also reported this extensively, detailing how managerial support helped to resolve emotional stress. Examples included giving staff space without chasing up incident reports, providing support via team meetings or one-to-ones and generally being proactive and accessible after an event.

For peer review only - http://bmjopen.bmj.com/site/about/guidelines.xhtml

Managers' guidance and reassurance that they have confidence in individuals involved in an incident was highlighted as particularly important. Conversely, inadequate managerial support compounded isolation and hindered recovery.

#3 Post-incident debrief (n=15): Debriefs were also commonly experienced by staff, however descriptions of what these entailed were not always provided. Where details were available, the format and implementation were inconsistent across studies. For example, qualitative studies highlighted that these might be voluntary, certain staff might not be involved or invited, or the focus was on learning and attributing blame rather than emotional recovery.

Discrepancies between support desired versus experienced

An overarching finding is that healthcare staff commonly experienced their organisation's support as insufficient, contingent on one's role/seniority, non-existent or unclear. Themes of resentment and injustice ran through many of the included studies. There appear to be discrepancies between what healthcare staff want and what they actually receive (see Supplemental Table). 'Peer support' needs appear to be appropriately met overall, but overlooked or neglected is the very highly rated need for 'practical support and guidance'. Similarly unmet is the desire for a supportive, learning-oriented culture; no experiences were reported of primary level interventions. By contrast, the procedural, routine 'Mortality and Morbidity meeting' was not a top 5 most desired support, yet, despite not technically being a wellbeing intervention, was the fifth most commonly experienced form of support.

Specific support interventions reported in empirical studies (Research Question 2)

In addition to the above 72 qualitative studies and surveys, a further 27 studies featured an evaluation of a single support intervention with healthcare staff. These are outlined in Table 2, grouped according to whether they were primary- or secondary-level interventions (no papers evaluating tertiary interventions were identified). Of these 27 intervention papers, 11

For peer review only - http://bmjopen.bmj.com/site/about/guidelines.xhtml

341 examined intervention effectiveness and were included at Stage 2 of the review to address
342 research question 3.

343 **Table 2: Individual interventions to support healthcare staff coping with patient safety**
344 **incidents - reported in empirical research papers**

**Support Level**	**Intervention Name Description**	**Study Reference**	**Setting; *healthcare staff sample***
PRIMARY	The 'When Things Go Wrong' Curriculum	[67]	University; *Medical students*
	'Interactive teaching session addressing personal and professional effects of medical errors'	[58]	University; *Medical students*
	Adverse Events Committee Program: annual 'Safety Education Workshop'	[68]	Hospital; *Psychiatry Residents*
	'Resilience coaching intervention to prepare UK healthcare professionals for the occurrence of stressful events'	[56, 69]	University, Hospital; *Multiple disciplines (qualified staff, trainees)*
	'Mitigating impact in second victims' online programme	[57]	Online [hospital]; *Multiple disciplines*
SECONDARY	Adverse Events Committee Program	[68]	Hospital; *Psychiatry Residents*
	Online, 10-chapter e-book reference guide for responding to & supporting those involved in an incident	[70]	Mental health organisation; *All staff*
	University of Missouri Health Care's 'forYOU' peer support program (wholly or modelled on, using Scott Three-tiered model of Second Victim Support [48])	[48, 60, 63, 64, 66, 71-75]	Hospital, incl. outpatients [61, 63]; *OBGYN staff* [64, 75], *Paediatric staff* [60], *Pharmacy staff* [74], *Neonatal ICU staff* [73], *all staff* [48, 66, 71, 72]
	The Brigham and Women's Hospital Peer Support Service	[76, 77]	Hospital; *Surgeons & trainees* [76], *All staff* [77]
	The Resilience in Stressful Events (RISE) program.	[59, 62, 78, 79]	Hospital; *paediatric staff* [78, 79], *nurses* [59, 62].
	'Swaddle' second victim staff support program (hybrid of above programs)	[80]	Hospital; *All staff*
	Certified Registered Nurse Anaesthetist Peer Support Program	[65]	Hospital; *Certified Registered Nurse Anaesthetists*
	The 'Buddy Study' peer support program	[81]	Hospital; *OBGYN midwives; ER & internal medicine physicians.*
TERTIARY			

345

346 Stage 2 Review Findings

347 How effective are existing support mechanisms/interventions designed to: (a) prevent poor
348 psychological outcomes for healthcare staff after PSIs (primary-prevention); (b) support staff

For peer review only - http://bmjopen.bmj.com/site/about/guidelines.xhtml

in the aftermath of PSIs (secondary-prevention); offer psychological support for those affected by involvement in PSIs (tertiary-intervention) (Research Question 3).

Characteristics of studies included at stage 2

Eleven studies were included at this stage and are summarised in Table 3. Of these 11 studies of intervention effectiveness, three featured primary-level interventions and eight involved secondary level interventions. There were no papers of tertiary level support. Most were conducted in the United States [n=7: 58, 59, 60-62, 64, 65], followed by Australia [63], the UK [56], Spain [57] and Indonesia [66]. Studies were quantitative [n=9: 57, 58, 60, 61, 62, 64-66] and mixed methods [56, 59]. Samples included uni-disciplinary [56, 58, 59, 62, 65] and multi-disciplinary groups in a specific department [60, 64] or whole organisation [57, 61, 63, 66].

For peer review only

For peer review only - http://bmjopen.bmj.com/site/about/guidelines.xhtml

360 **Table 3: Summary of included studies at Stage 2**

First author (reference), country	Intervention name	Intervention description	Study Design (Quality appraisal score)	Sample, Setting	Outcome measure related to effectiveness (measure used)	Outcomes
Johnson [56]<br><br>UK	*No name. 'Resilience coaching intervention to prepare healthcare professionals for stressful healthcare events, particularly adverse events'*	*A psychologist-delivered, CBT-based, half day workshop (psycho-educational didactic teaching, small group discussion and experiential exercises) followed by a one-hour, individual coaching phone call.*	*Uncontrolled before-after study; mixed methods - semi-structured interviews & questionnaires.*<br><br>*No comparison of users/non-users.*<br><br>*Four data collection points: baseline, after workshop, after coaching call, 4-6 weeks post baseline.*<br>*(32)*	*Midwives, paramedics, paediatric consultants. Trainee obstetrics & gynaecology doctors, trainee paediatric doctors. Physician Associate students, sonography & mammography students (n=66).*<br><br>*Hospital & University.*	*Confidence in coping with adverse events* (3-item, purpose designed measure);<br><br>*Knowledge about resilience, coping strategies, personal strengths* (5-item purpose designed measure T1 & T2 only);<br><br>*Resilience to cope with future adverse events* (Brief Resilience Scale [82]).	*Compared with T1 baseline, statistically significant increases post intervention in (1) Confidence at T2 (unadj. $\beta = 2.43$, 95% CI 2.08–2.79, $d = 1.55$, $p < .001$), T3 (unadj. $\beta = 2.81$, 95% CI 2.42–3.21, $d = 1.71$, $p < .001$) and T4 (unadj. $\beta = 2.75$, 95% CI 2.31–3.19, $d = 1.52$, $p < .001$; (2) Knowledge at T2 (unadj. $\beta = 1.14$, 95% CI 0.82–1.46, $d = 0.86$, $p < .001$); (3) Resilience at T3 (unadj. $\beta = 2.77$, 95% CI 1.82–3.73, $d = 0.90$, $p < .001$) and T4 (unadj. $\beta = 2.54$, 95% CI 1.45–3.62, $d = 0.65$, $p < .00$).*
Mira [57]<br><br>Spain	*Mitigating Impact in Second Victims (MISE) online program*	*A flexible online programme: 'informative' package (information and research about patient safety incidents and second victimhood) and 'demonstrative' package (videos showing what to do/not do after patient safety incident, covering emotional, family, work consequences of adverse events, how to inform patient, access support, support others, how to be prepared/ what to do).*	*Uncontrolled before and after study; quantitative.*<br><br>*Survey/test issued pre and post completion.*<br><br>*No comparison of users/non-users.*<br>*(23)*	*Multi-disciplinary: physicians and nurses (n=292)*<br><br>*Online.*	*Knowledge changes regarding:*<br><br>*(1) Informative Package of the website* (12-item purpose designed test completed pre & post intervention);<br><br>*(2) Demonstrative package of the website* (8-item purpose designed test completed pre & post intervention).	*Significant differences between pre- and post- tests results.*<br><br>*Test 1 Informative package: Pre-intervention (M=6.9, SD =2.0) and post-intervention (M=8.8, SD=1.6; t_{265}=–10.0, P<.001).*<br><br>*Test 2 Demonstrative package: Pre-intervention (M=6.3, SD=1.5) and post-intervention (M=7.2, SD=1.0; t_{265}=–6.2, P<.001).*

For peer review only - http://bmjopen.bmj.com/site/about/guidelines.xhtml

Musunur [58]<br><br>USA	*When Bad Things Happen: Training Medical Students to Anticipate the Aftermath of Medical Errors*	*A one-hour education session delivered within the curriculum at medical school (didactic presentation, video vignettes of seniors discussing error experiences, and guided small group discussion).*	*Uncontrolled before-after study; quantitative.*<br><br>*Survey/test issued pre and post education session, across 2 years – 2016-17.*<br><br>*No comparison of users/non-users.*<br>*(18)*	*Pre-clerkship medical students (n=300).*<br><br>*University.*	*(1) awareness of resources to cope with and report medical error;*<br><br>*(2) confidence in ability to recognise & cope with medical error;*<br><br>*(3) knowledge of medical error leading to physician burnout;*<br><br>*(4) attitudes to error, including workplace culture, acknowledging responsibility*<br><br>(ALL: 14-item purpose designed pre and post intervention test/survey).	*Overall responses to education session (all 4 domains): significant difference between post- and pre-session results in 2016 (p<0.001; by 0.34 units of mean change, SD=0.35 units) and 2017 (p<0.001, by 0.33 units of mean change, SD=0.03 units).*<br>*Responses by domain: Significant changes in pre and post session results for (1) awareness of resources and (2) confidence in coping with error - in 2016 and 2017. No significant changes in either 2016 or 2017 in pre and post results for (3) knowledge of medical error or (4) attitudes to error.*
Connors [59]<br><br>USA	*RISE program (Resilience in Stressful Events)*	*Peer support programme. Peers, trained in psychological first aid, respond to self- or colleague-referrals within 30 minutes of receiving notification, 24 hours a day, seven days a week. Peers are clinically-based, but tend not to be known to callers, and focus on how the staff member is feeling rather than event details, supporting them to identify coping strategies to aid recovery – usually in person but sometimes by phone.*	*Cross sectional survey; mixed methods – open and fixed response questionnaire.*<br><br>*Compared users/non-users.*<br>*(24)*	*Nurses and nurse leaders (n=412).*<br><br>*Hospital.*	*(1) Professional burnout* (5 items adapted from Emotional Exhaustion domain of Maslach Burnout Inventory [83]);<br><br>*(2) Job satisfaction* (3 items from previously validated questionnaire [84]);<br><br>*(3) Personal resilience* (2 of 4 items from Resilience domain in SVEST [85]).	*Staff Nurses:* *No significant differences between RISE users/non-users in (2) Job satisfaction (67% vs. 61%; p=0.45). Significant differences in burnout & resilience: (1) Burnout - more RISE users than non-users reported some level of burnout (93% vs 67%, p=0.038). Odds of indicating some level of burnout were 4.3 times greater for recent RISE-users than non-users (p=0.048). (3) Resilience: RISE users (past year) significantly more likely to report resilience (57% vs 36%, p=0.025).*<br>*Nurse Leaders:* *No significant differences between RISE activators/non-activators in: (1) burnout (40% activators vs 50% non-activators reported at*

For peer review only - http://bmjopen.bmj.com/site/about/guidelines.xhtml

						least some burnout; p=0.42) and (2) job satisfaction (75% activators vs. 85% non-activators; p=0.315). Significant differences in (3) Resilience: Non-activators were more resilient than activators (89% vs 50%, p=0.001). Odds of indicating strong resilience were 0.12 times lower for activators than never-activators (p=0.002).
Finney [60]<br><br>USA	*HELP (Healing Emotional Lives of Peers) program*	*Peer support programme. Based on/ informed by the University of Missouri Health Care 'forYOU' model [48]. Provides emotional support to individuals and groups, 24 hours a day, seven days a week. Grounded in Scott's three-tiered interventional model of support. Adaptations: peer supporters trained in all departments & job levels, embedding in every team, not just high-risk areas.*	*Uncontrolled before and after study: quantitative - Full department survey.*<br><br>*No comparison of users/non-users.* *(26)*	*Paediatric healthcare professionals (n=371 [pre-194/373; post-177/403]).*<br><br>*Hospital Paediatric inpatient unit, ICU*	*Impact of programme on Second Victim experience, encompassing: psychological distress; physical distress; colleague support; supervisor support; institutional support; non-work support; professional self-efficacy; absenteeism; turnover intentions* (ALL: Second Victim Experience and Support Tool [85]).	*One-year post intervention launch, compared to pre-intervention: Significantly fewer staff agreed institutional support was 'inadequate' (10.9% vs. 0.6%, p<0.001); Significantly fewer reported turnover intentions (18.2% vs 9.2%, p=0.014 (n.b. in table 2 this is cited as p=0.09); No significant differences on any other SVEST dimensions.*
Klatt [61]<br><br>USA	*Supporting Our Staff (SOS) Peer Support Program*	*Peer support programme. Based on/informed by the University of Missouri Health Care 'forYOU' model [48]. Adaptations: (a) peer supporters trained in all departments and job levels, embedding in every team, not just high-risk areas.*	*Uncontrolled before-after study: quantitative – organisational survey.*<br><br>*Planned a baseline survey pre-launch but unable to do so. Issued 3&9 months post-launch.*<br><br>*No comparison of users/non-users: compared SVEST responses of those who reported experiencing a*	*Multi-disciplinary (n=unclear).*<br><br>*All clinical areas, inpatient and outpatient settings, hospital quaternary care campus.*	*Impact of program on Second victim experience, encompassing: psychological distress; physical distress; colleague support; supervisor support; institutional support; non-work support; professional self-efficacy; absenteeism; turnover intentions* (ALL: Second Victim Experience and Support Tool [85]).	*3 months post-launch, SVEST issued to 6,109 HCPs; n=1,786 responses. At 9 months post-launch, n=1,188 responses. Authors compared responses of those involved in adverse outcome/error in past 3 years at 3- (n=766) and 9-months (n=549). No significant differences identified on any SVEST dimension.*

For peer review only - http://bmjopen.bmj.com/site/about/guidelines.xhtml

			recent adverse event at 3- and 9-months. (26)			
Moran [62]<br><br>USA	*Resilience in Stressful Events (RISE) program*	*Peer support programme. Peers, trained in psychological first aid, respond to self- or colleague-referrals within 30 minutes of receiving notification, 24 hours a day, seven days a week. Peers are clinically-based, but tend not to be known to callers, and focus on how the staff member is feeling rather than event details, supporting them to identify coping strategies to aid recovery – usually in person but sometimes by phone.*	*Cross sectional survey and Markov modelling to identify cost-benefits of a hospital having versus not having RISE; quantitative.* (23)	*Nurses (n=36).*<br><br>*Hospital.*	*Net monetary benefit & budget impact of having RISE.*<br>*Cost of running RISE program* (divided total financial cost to hospital of program by number of nurse users in 2015 (n=80) to get average cost per nurse). *Hospital costs for nurse turnover* (literature review) and *nurse sickness absence after involvement in patient safety incident* (hospital's Human Resources data).<br><br>*Probability of resigning and taking a sick day with/without RISE* (13-item purpose designed survey requesting estimations of outcomes from 2 hypothetical patient safety incident scenarios, e.g. How many days off do you think this nurse would take? How likely is it they would need mental health services or quit?).	*Cost of RISE = $656.25 per nurse user.*<br>*After RISE, decrease in turnover & sickness absence per nurse = $23,232.30.*<br>*Net monetary benefit saving of having the RISE program after 1 year = $22,576.05 per nurse using RISE.*<br><br>*RISE could save a hospital $1.81 million per year. Having RISE provided a positive net monetary benefit compared to not having RISE in 49,987 of the 50,000 (>99%) Monte Carlo simulations, at a mean net monetary benefit $23,360 (95% confidence interval, $3006 - $161,278).*
Morris [63]<br><br>Australia	*'Always There' peer support program*	*Peer support programme. Based on/ informed by the University of Missouri Health Care 'forYOU' model [48]. Adaptations: (a) peer supporters trained in all departments and job levels, embedding in every team, not just high-risk areas; (b) slightly reconceptualised Tiers 1 and 2: Tier 1 a 'warm response' - for staff experiencing cumulative/ acute stress; Tier 2 a 'hot*	*Uncontrolled before-after study: quantitative – organisation-wide survey.*<br><br>*Compared users/non-users – 2 years post-launch.* (19)	*Clinical and non-clinical staff (n=unclear).*<br><br>*ER, inpatient and community settings, large public mental health service.*	*Second victim experiences of those engaging with program, encompassing: psychological distress; physical distress; colleague support; supervisor support; institutional support; non-work support; professional self-efficacy; absenteeism; turnover intentions* (Second Victim Experience and Support Tool [85], used within 'Voices of the Staff' survey).	*Compared survey results pre and post launch of engagers (n=38) and non-engagers (n=232). No significant differences between groups in all but one SVEST dimensions. Significant differences identified between groups in 2 out of 3 items in 'institutional support' dimension (i.e., not for the full dimension): item 18 "my organisation understands the needs of staff in the aftermath of critical incidents" (81.6% of users vs 57.8% of*

For peer review only - http://bmjopen.bmj.com/site/about/guidelines.xhtml

		response' – practical and emotional support for individuals or teams after a traumatic adverse event (e.g., suicide attempt). Part of broader just culture transformation.				*non-users; p=.005) and item 19 "provides more resources to assist with recovery" (78.9% users vs. 59.9% non-users; p=.024).*
Rivera-Chiauzzi [64]<br><br>USA	*No name: 'Structured peer support program'.*	*Peer support programme. Based on/ informed by the University of Missouri Health Care 'forYOU' model [48]. 4 peer supporters attended 'Second Victim train the trainer workshop' by forYOU team. Trained peers then contacted all staff involved in an adverse event within one week, had in-person, one-to-one confidential chat to assess wellbeing, provide information on second victim recovery stages and EAP. Peer supporters phoned staff randomised to 'Enhanced' group one week later, then monthly up to 6 months until no longer needed.*	*Randomised trial; quantitative – departmental survey.*<br><br>*3 timepoints: baseline, 3 months and 6 months post intervention.*<br><br>*Compared users/non-users.*<br>*(20)*	*Physicians, Nurses, Physician Assistants (n=42: 23 intervention ['enhanced care']; 19 control ['routine care']).*<br><br>*Obstetric and maternal foetal medicine department, Hospital.*	*To develop & evaluate peer support program.*<br>*Primary outcome: use of resources & perceptions of helpfulness.*<br>*Secondary outcome: <u>effect on recovery stage after PSI</u> and duration of support use* (ALL: Survey, though unclear if questionnaire was adapted from forYOU toolkit or purpose designed. Included demographics, use of informal resources, perceived helpfulness, barriers, current recovery stage*).*	*<u>Effect on recovery stage:</u>* ***At baseline:*** *Most participants - 28/42 (13/23 enhanced; 15/19 routine) - were at Stage 6 'Thriving'; 14/42 were at an earlier recovery stage: Stage 1-3 n=7 (enhanced 6/7; routine 1/7); Stage 4-6 [excluding those 'thriving'] n=7 (enhanced 4/7; routine 3/7).* ***At 6 months:*** *100% of participants, regardless of group, reported that they were 'thriving'.*<br><br>***Enhanced group:*** *Most needed less than 3 months' support. 62.5% declined follow-up after first contact; 91.3% declined follow-up after second contact. Significantly more likely than routine group to consider departmental leadership as one of the most helpful resources (p=0.02).*
Thompson [65]	*CRNA (Certified registered nurse anesthetists) Peer support Program*	*Peer support programme. Does not report being based on previously established models. 8 volunteers trained to provide peer support 24 hours a day. Support could be activated through self-, colleague- or lead CRNA-referral, or by the*	*Uncontrolled before and after study: quantitative – departmental survey.*<br><br>*Issued pre- and one-month after trial period to all CRNAs.*<br><br>*No comparison of users/non-users.*<br>*(22)*	*CRNAs. (n=83 [pre-n=51; post-n=32]).*<br><br>*CRNA Department, large academic medical centre.*	*Impact of second victim peer support protocol, compared to current post-adverse event hospital practices, on psychological distress; physical distress; colleague support; supervisor support; institutional support; non-work support; professional self-efficacy; absenteeism; turnover intentions*	*No statistically significant differences were found between SVEST scores pre and post intervention (p>.05).*

For peer review only - http://bmjopen.bmj.com/site/about/guidelines.xhtml

		peer supporter noticing a colleague's distress.			(Second Victim Experience and Support Tool [85]).	
Wijaya [66]<br><br>Indonesia	*No name 'second victim support program'*	*Peer support programme. Based on/ informed by the University of Missouri Health Care 'forYOU' model [48].*	*Controlled before and after study: quantitative – hospital-wide survey.*<br><br>*Issued: pre-launch; up to 1 year post launch; 1-2 years post-launch.*<br><br>*Compared users/non-users.*<br>*(17)*	*Doctors and nurses. (n=unclear. Unsure if exactly the same 87 participants surveyed across phases)*<br><br>*Hospital.*	*Perceptions of patient safety culture within the hospital* (Hospital Survey on Patient Safety Culture [86]).	*Phase 1: N=47 (/87; 54% response); Phase 2: N=60 (/same 87; 69% response); Phase 3: N=65 (/same 87; 75% response rate).*<br>*T1-T2: All HSOPSC ratings increased: Significant increase in patient safety culture (t=4.284, p=0.001, Cohen's d 0.854) large effect size).*<br>*Compared to control hospital, significant increase in patient safety culture within intervention hospital (DID coefficient 0.738, SE= 0.258, p= 0.007). No significant differences at intervention hospital results between T 2 & T3 indicating sustained effect (t=0.378, p=0.713, Cohen's d 0.0476).*

For peer review only

For peer review only - http://bmjopen.bmj.com/site/about/guidelines.xhtml

Intervention users were compared with non-users in around a third of studies [59, 63, 64, 66]. A range of follow-ups was included in the sample: from a single survey pre- and post-implementation [60, 63, 65], to multiple follow-ups (3- and 6-months post intervention [64]; 3- and 9-months post launch [61]; 1- and 2-years post launch [66]. Two studies involved a single post-implementation survey [59, 62].

Three studies featured primary-level interventions [56-58]. The formats of these intervention are outlined in Table 3. All aimed to raise awareness of the prevalence of adverse events, understanding of their personal and professional impact, to prepare participants for involvement in an adverse event and ensure they knew how to access resources and support after a PSI. In addition, Mira et al. [57] targeted practical, personal preparation, including how to inform patients and what to say/not say to colleagues and Johnson et al. [56] targeted the development of participants' cognitive flexibility, self-esteem and positive attributional style. All primary-level intervention studies measured changes in users' knowledge (of patient safety incidents, second victimhood and the impact, appropriate responses/actions in the aftermath [57]; of resilience, coping strategies, personal strengths [56]; of medical error leading to physician burnout [58]), two explicitly measured changes in confidence to cope with adverse events [56, 58] and one measured resilience to cope with future events [56]. Changes in awareness of support resources and attitudes to error were also measured by one [58]. Purpose-designed questionnaires/tests were used in all cases, supplemented in one [56] by the Brief Resilience Scale [82].

Eight studies featured secondary-level interventions [59-66], all peer support programmes which aimed to offer tailored, flexible support to staff in the aftermath of an adverse event, with referral to more specialist psychological support if required/desired (for formats, see Table 3). One programme [63] was reported as part of a wider just culture organisational change initiative. Specific programme goals differed, including 'to provide

For peer review only

timely support' after a stressful event [62], 'to decrease second victim distress' [65] and 'to mitigate challenges and improve the wellbeing of staff' immediately after an incident, and, longer term, enhancing resilience, self-care and 'connections with colleagues and the organisation' [63]. Five papers [60, 61, 63, 64, 66] feature programmes based on or informed by the University of Missouri Health Care 'forYOU' framework (grounded in Scott's three-tiered interventional model of support), which aims to promote the healthcare professional's transition through emotional recovery after an incident [48, p. 238]. Some included programmes [60, 61, 63] have slightly adapted this forYOU model to suit their local needs (see Table 3). Two papers relate to the Resilience in Stressful Events (RISE) programme [59, 62], and the final study features the Clinical Registered Nurse Anaesthetist (CRNA) peer support program [65].

In terms of outcomes measures used in secondary-level intervention studies, four [60, 61, 63, 65] measured changes in levels of psychological and physical distress, perceptions of colleague, supervisor and institutional support, professional self-efficacy, absenteeism and turnover intentions, using the Second Victim Experience and Support Tool [85]. One [64] measured changes in current stage of recovery after a PSI based on Scott and colleagues' [7] six-stage second victim recovery trajectory. It is unclear whether this team used an existing or purpose-designed tool. Differences in burnout, job satisfaction and resilience were investigated in one study [59], using a combination of existing measures (abridged Maslach Burnout Inventory [83], Job Satisfaction Scale [84], Brief Resilience Scale [82]). Two studies examined organisational level data: changes in perceptions of organisational culture post implementation [66], using the Hospital Survey on Patient Safety Culture [86] and costs associated with implementing versus not implementing the programme, through modelling probable savings from reduced sickness absence and turnover [62].

For peer review only - http://bmjopen.bmj.com/site/about/guidelines.xhtml

Study Quality

Study quality was variable. Insufficient detail was an overarching issue across these intervention studies, particularly with regard to enabling study replication. Theoretical underpinning was limited (most studies only referred to second victimhood in their introduction) and insufficient description tended to be provided of samples and sampling strategies in relation to aims. There was only one randomised trial in the sample, meaning that most studies did not use a comparison group and that staff/worksites were assigned non-randomly to interventions when testing for effectiveness. These are sources of potential bias. Self-reported data were relied upon in the majority of cases, increasing the potential for response bias. Evidence of stakeholder engagement in design was limited across studies, although most seemed to be led by clinical teams. We did not exclude any study based on its quality appraisal outcome.

Our TIDieR assessment of the intervention descriptions also revealed that theoretical underpinnings were provided in less than half of studies included at stage 2. Details regarding intervention materials, associated procedures/activities and facilitators were well described overall. Just one study explicitly mentioned fidelity assessment of intervention delivery, although a small number of studies referenced regular facilitator meetings for supervision, debriefing, learning and continuous improvement.

How effective are existing ***primary-prevention*** *interventions?*

Evidence was largely positive for the effectiveness of the three primary-level interventions in preparing healthcare staff and trainees for exposure to adverse events. All three studies reported significant improvements in targeted outcomes, particularly in relation to knowledge and awareness of resources in relation to the second victim phenomena (see Table 3). Users of Mira et al.'s [57] MISE online training programme demonstrated significant improvements in their knowledge of basic patient safety concepts, the prevalence and nature of adverse

For peer review only - http://bmjopen.bmj.com/site/about/guidelines.xhtml

events. Similarly, medical students participating in Musunur et al.'s [58] 1-hour teaching session training demonstrated significant improvements in confidence in their ability to recognise and cope with medical error and in their awareness of resources to cope with and report medical error. Participants in Johnson et al.'s [56] half day workshop also showed significant improvements in confidence with coping with adverse events, knowledge about resilience factors, coping strategies and awareness of personal strengths, which were maintained 4-6 weeks after the final workshop.

*How effective are existing **secondary-prevention** interventions?*

Evidence for the effectiveness of secondary level peer-support based interventions was more mixed. With regards to adaptations of the ForYOU programme: Klatt et al [61] identified no significant changes on any SVEST dimension ratings when following up at 3 and 9 months after their programme launch. In the only randomised trial in the sample, Rivera-Chiauzzi et al. [64] found no significant differences at 6 months post-enrolment in the study, between their intervention and control groups regarding ability to recover from an adverse event into the optimal 'thriving' recovery stage: both groups of staff recovered equally well.

More positive findings were reported, however, in Finney et al. [60] and Wijaya, Mohamad and Hafizzurachman [66]. Finney et al. [60] found significant changes in the institutional support and turnover intentions dimensions in SVEST: they report that, post implementation, 'inadequate' ratings of institutional support and turnover intentions fell significantly (however, p values in the article body text (p=0.014 - significant) and Table 2 (p=0.09 – not significant) do not correspond). Wijaya and colleagues [66] reported significant increases in HOSPSC survey ratings in the intervention hospital one year after introducing the programme, which were significantly higher than those of staff in the control hospital. This improved safety culture was sustained two years post-introduction of the programme. Morris et al. [63] found no significant differences between 'Always There' programme users

For peer review only - http://bmjopen.bmj.com/site/about/guidelines.xhtml

and non-users one year post-launch on any overall dimension of SVEST, although users did rate two (out of three) items in the 'institutional support' dimension significantly higher than non-users.

Turning to the RISE programme, two studies evaluated its impact specifically on the nursing workforce in their hospital. Moran et al. [62] calculated that having the RISE programme could save a hospital $1.81 million per year in reducing turnover and absence. Connors and colleagues [59] found no significant difference in job satisfaction between either nurse users and non-users, or between nurse leader activators and non-activators. Compared to non-users, nurses using RISE were significantly more likely to report higher levels of resilience and some level of burnout. Nurse leaders who did not activate RISE for their staff reported significantly higher resilience than those who did.

Finally, Thompson et al.'s [65] quality improvement project found no statistically significant differences between pre- and post-intervention implementation SVEST scores of all departmental certified registered nurse anaesthetists surveyed, after one month of implementing their peer support programme.

DISCUSSION

We used a widely-respected, multi-level stress prevention model [49] to map the empirical evidence for what type of support healthcare professionals *want* for PSIs, what they tend to *receive*, and what organisational strategies are *effective*. Our findings revealed that healthcare professionals want organisational support at multiple levels: a supportive and learning oriented culture (primary-prevention level support), peer support, practical support and guidance, post-incident debriefing to clarify and process what happened (secondary-prevention level support) and access to professional mental health support (tertiary level). However, there is limited evidence that organisations are giving staff what they want:

For peer review only - http://bmjopen.bmj.com/site/about/guidelines.xhtml

overwhelmingly, organisational support for PSIs is perceived as inadequate, non-existent or dependent on one's organisational status. Where support *is* provided, secondary level approaches dominate - largely at a local level from peers and one's line manager. There is a gap in primary level (preparatory, proactive, preventive) approaches which tend to be missing from staff reports.

For healthcare organisations seeking to invest in evidence-based interventions to support staff with PSIs, effectiveness data are lacking. There is more robust evidence for the effectiveness of ***primary interventions*** designed to equip and prepare staff for involvement in PSIs, than for ***secondary interventions***, which aim to mobilise support and promote staff coping *after* involvement in a PSI. However, despite this, secondary level interventions dominate the literature, particularly the popular peer support programmes which have mixed evidence for their effectiveness. No studies were identified featuring a tertiary intervention, that is, individualised support for staff experiencing severe and/or longer-term emotional sequelae and/or disturbed ability to function after a PSI. Whilst there is compelling evidence more broadly that Cognitive Behavioural Therapy and Eye Movement Desensitisation and Reprocessing are effective in treating PTSD [87], data are not available to be able to recommend their particular effectiveness in alleviating second victim-related sequelae.

Our finding that there is a lack of robust empirical evidence for the effectiveness of specific PSI-related interventions is in line with previous research [39-41, 46]. As others have noted, this may be attributable, in part, to being a relatively young research field and an initial focus on intervention feasibility, acceptability and implementation challenges [81]. We identified only one RCT. Few included a theoretical underpinning or basis for their design. Organisational level outcome data (retention, sickness absence, cost effectiveness, organisational culture) featured in just two studies [62, 66]. It is becoming increasingly important for organisations, particularly post-COVID, to have reliable intelligence about how

For peer review only - http://bmjopen.bmj.com/site/about/guidelines.xhtml

best to support staff after PSIs and what works, to justify investment to stakeholders including staff. Therefore, there is a need for the field to evolve rapidly and for robust studies to be funded.

Collectively, the wide range of healthcare staff voices across our included studies corroborate the need for organisational support for PSIs to be multi-levelled and comprehensive. This finding supports Seys et al.'s [44] conclusions in their recent narrative review, and also reflects the wider occupational stress literature more generally [e.g., 49, 50, 51, 88]. A lack of primary level interventions in healthcare organisations to help staff with PSIs is perhaps not surprising: organisations generally tend to prefer secondary and tertiary interventions [49]. Targeting or changing individuals is considered easier, more cost effective, tangible/visible and less disruptive than changing organisations, e.g., changing organisational culture, working conditions, job designs [49, p.10].

However, disregarding primary level support may be problematic for two reasons. Firstly, research surrounding broader work-stress interventions suggests that primary interventions may be more effective at stress reduction than secondary or tertiary approaches [89, 90]. Secondly, multi-level intervention strategies combining individual and organisational level interventions are more effective than uni-level interventions alone [89, 91, 92], therefore organisations risk poorer returns on their investment if they fail to adopt a comprehensive approach. Primary level interventions, particularly those addressing improvements in culture and working practices, appear to be especially important in healthcare settings, because of the significant impact the presiding patient safety culture has on staff's recovery trajectory after a PSI [16].

Organisations that fail to invest in primary level support – specifically, implementing a fair and just organisational culture - but provide a formal peer support programme (secondary level) or EAP (Tertiary level), risk staff viewing these provisions as 'tokenistic',

For peer review only

For peer review only - http://bmjopen.bmj.com/site/about/guidelines.xhtml

or as signifiers that the organisation has abdicated responsibility for its staff because it is not tackling poor culture which is a major source of distress [93]. In fact, when employers ask their staff to identify factors that would have the greatest impact on their wellbeing at work, they overwhelmingly suggest better communication, staffing and interpersonal connectivity, rather than issues targeted by wellbeing programmes [39]. Themes of injustice and resentment were also apparent within many included studies in the review, which, independent of involvement in a PSI, are risk factors for turnover intentions in healthcare staff [94].

In line with previous research, our review found that peer support is a highly desired and commonly received form of support [15, 31, 48]. However, we also identified considerable ambiguity around the term 'peer support' within included studies: it was often unclear whether it referred to informal support from team colleagues or a formal peer support programme. Given that formal peer support programmes are the most common organisational intervention provided, this has highlighted a need to ensure that such programmes are meeting the right requirement. For example, if a model offers peer support from outside one's team, but one's home team is unsupportive and one's leader is critical, then ongoing, day-to-day recovery in that location is likely to be more difficult.

Perhaps unsurprisingly, manager support emerged from this review as the second most commonly received form of organisational support, although there were also numerous accounts of inadequate managerial support compounding feelings of isolation and hindering recovery. This was also found in our related review [31]. The way in which supervisors or leaders treat their staff is crucially important, because, to staff, they 'embody' the organisation: staff form perceptions and experiences of their organisation from their supervisors/leaders behaviour and treatment of them [95]. Leadership quality has long been associated in the field of occupational health psychology with both positive outcomes, such

For peer review only - http://bmjopen.bmj.com/site/about/guidelines.xhtml

as staff wellbeing, psychological safety, and negative outcomes, including stress, heart disease and workplace incidents [90]. In healthcare in particular, compassionate leadership is being increasingly linked to staff wellbeing and positive safety culture [96, 97]. Given managers' pivotal roles in supporting staff with PSIs, there is a strong case for organisations to provide compassionate leadership development in this area within their multi-level PSI-support strategy, as a proactive, preventive, primary level occupational health psychology intervention [90].

To our knowledge this is the first systematic review to apply a multi-level occupational health stress prevention model to examine second victim requirements and organisational support solutions. A second strength of the review is that it specifically addressed intervention effectiveness. However, a number of limitations must be acknowledged. Despite the inclusion of a medical librarian on our review team, it is possible that our search strategy omitted eligible studies. For example, we may have missed important papers which were not in English or featured more unusual terms for PSIs (terminology is non-standard and varies considerably across medical disciplines).

Of significance is that the vast majority of intervention developers and paper authors within our stage 2 sample are clinicians. Secondary level interventions dominate; fewer studies address primary level prevention of second victimhood. This suggests that most interventions are pragmatic, responsive solutions to meet needs recognised at ground level by clinicians themselves. It also suggests that the recognition of and ownership for second victimhood as a serious, intrinsic occupational health risk to be addressed is located in clinical or patient safety teams; it does not reside with organisational senior leadership teams (including Human Resources, Organisational Development and Occupational Health). However, building the much-needed comprehensive multi-level strategies requires

For peer review only

For peer review only - http://bmjopen.bmj.com/site/about/guidelines.xhtml

commitment from senior leadership teams of resources, funding and time; it cannot solely be responsibility of patient safety teams or local clinical departments.

Despite 20 years of investment to reduce the number of PSIs, they continue to persist [98]: ‘to err is human’ [99]. Given the fact that healthcare organisations can never entirely remove this intrinsic hazard, the importance of having an effective, comprehensive institutional support strategy in place for patient safety incidents is evident, to aid adaptive recovery, minimise distress, sickness absence and turnover [6]. There is an incontrovertible, pressing need for healthcare organisations to address the multi-faceted occupational health risk for their staff of being involved in a PSI. Organisations need reliable evidence about what interventions are effective, however this is currently lacking. In order to advance the field, we require more methodologically robust studies, which describe theoretical bases for interventions and involve in their design comparison groups, randomisation, organisational- and individual-level outcomes. In addition, more research is needed into primary and tertiary-level interventions to help healthcare professionals with PSIs.

SUPPORTING INFORMATION

S1 File. PRISMA checklists.

S2 File. Search criteria.

S3 File. Detailed narrative summary findings and reference list for surveys and qualitative studies included at Stage 1.

S4 File. Description of fourth and fifth most desired and experienced forms of support.

ACKNOWLEDGEMENTS

For peer review only - http://bmjopen.bmj.com/site/about/guidelines.xhtml

This paper presents independent research supported by the National Institute for Health and Care Research (NIHR) Yorkshire and Humber Patient Safety Translational Research Centre and Patient Safety Research Collaboration. The views expressed are those of the authors, and not necessarily those of the NIHR or the Department of Health and Social Care. Our sincere thanks to Jane Heyhoe (JH) who supported the team with this research before her departure from academia, and to Stephen Mears, University of New South Wales, Australia for his help with the database searches.

AUTHOR CONTRIBUTIONS

Conceived and designed the experiments: RSE RH RS ES JH RL. Performed the experiments: RSE RH RS ES JH HH RL. Analyzed the data: RSE RH RS ES JH HH MME RL. Contributed reagents/materials/analysis tools: RSE RH RS ES JH HH MME RL. Wrote the paper: RSE RL RH

REFERENCES

1. Mahase E. Invest in health workforce or risk collapse, WHO warns governments. BMJ. 2023;380:713. https://doi.org/10.1136/bmj.p713
2. World Health Organization Regional Office for Europe. Health and care workforce in Europe: time to act. https://www.who.int/europe/publications/i/item/9789289058339. 2022 14 September 2022.https://www.who.int/europe/publications/i/item/9789289058339
3. Castro Lopes S, Guerra-Arias M, Buchan J, Pozo-Martin F, Nove A. A rapid review of the rate of attrition from the health workforce. Human Resources for Health. 2017;15(1):1-9. https://doi.org/10.1186/s12960-017-0195-2
4. Ratwatte M. The NHS workforce crisis is a retention crisis. BMJ. 2023;380:602. https://doi.org/10.1136/bmj.p602

For peer review only - http://bmjopen.bmj.com/site/about/guidelines.xhtml

5. Downey E, Fokeladeh HS, Catton H. What the COVID-19 pandemic has exposed: The findings of five global health workforce professions. Geneva: World Health Organization.; 2023. Report No.: 9240070184
6. Burlison JD, Quillivan RR, Scott SD, Johnson S, Hoffman JM. The effects of the second victim phenomenon on work-related outcomes: connecting self-reported caregiver distress to turnover intentions and absenteeism. Journal of Patient Safety. 2021;17(3):195-9. 10.1097/PTS.0000000000000301
7. Scott SD, Hirschinger LE, Cox KR, McCoig M, Brandt J, Hall LW. The natural history of recovery for the healthcare provider "second victim" after adverse patient events. BMJ Quality & Safety. 2009;18(5):325-30. https://doi.org/10.1136/qshc.2009.032870
8. Van Gerven E, Vander Elst T, Vandenbroeck S, et al. Increased risk of burnout for physicians and nurses involved in a patient safety incident. Medical Care. 2016;54(10):937-43. 10.1097/MLR.0000000000000582
9. Martens J, Van Gerven E, Lannoy K, et al. Serious reportable events within the inpatient mental health care: Impact on physicians and nurses. Revista de Calidad Asistencial. 2016;31:26-33. https://doi.org/10.1016/j.cali.2016.04.004
10. NHS England. Patient Safety. 2023 [Available from: https://www.england.nhs.uk/patient-safety/].
11. Vincent C, Neale G, Woloshynowych M. Adverse events in British hospitals: preliminary retrospective record review. BMJ. 2001;322(7285):517-9. https://doi.org/10.1136/bmj.322.7285.517
12. Connolly W, Rafter N, Conroy RM, Stuart C, Hickey A, Williams DJ. The Irish National Adverse Event Study-2 (INAES-2): longitudinal trends in adverse event rates in the Irish healthcare system. BMJ Quality & Safety. 2021;30(7):547-58. https://doi.org/10.1136/bmjqs-2020-011122
13. Michel P, Brami J, Chanelière M, et al. Patient safety incidents are common in primary care: A national prospective active incident reporting survey. PLOS One. 2017;12(2):e0165455. https://doi.org/10.1371/journal.pone.0165455
14. Panagioti M, Stokes J, Esmail A, et al. Multimorbidity and patient safety incidents in primary care: a systematic review and meta-analysis. PLOS One. 2015;10(8):e0135947. https://doi.org/10.1371/journal.pone.0135947

For peer review only

For peer review only - http://bmjopen.bmj.com/site/about/guidelines.xhtml

15. Wu AW, Shapiro J, Harrison R, et al. The impact of adverse events on clinicians: what's in a name? Journal of Patient Safety. 2020;16(1):65-72. 10.1097/PTS.0000000000000256

16. Quillivan RR, Burlison JD, Browne EK, Scott SD, Hoffman JM. Patient safety culture and the second victim phenomenon: connecting culture to staff distress in nurses. The Joint Commission Journal on Quality and Patient Safety. 2016;42(8):377-86. 10.1016/s1553-7250(16)42053-2

17. Murray JS, Clifford J, Larson S, Lee JK, Sculli GL. Implementing just culture to improve patient safety. Military Medicine. 2023;188(7-8):1596-9. https://doi.org/10.1093/milmed/usac115

18. van Marum S, Verhoeven D, de Rooy D. The barriers and enhancers to trust in a just culture in hospital settings: a systematic review. Journal of Patient Safety. 2022;18(7):e1067-e75. 10.1097/PTS.0000000000001012

19. Turner K, Sveticic J, Grice D, et al. Restorative Just Culture significantly improves stakeholder inclusion, second victim experiences and quality of recommendations in incident responses. J Hosp Adm. 2022;11(2):8-17. https://doi.org/10.5430/jha.v11n2p8

20. Kaur M, De Boer RJ, Oates A, Rafferty J, Dekker S, editors. Restorative just culture: a study of the practical and economic effects of implementing restorative justice in an NHS trust. International Cross-industry Safety Conference (ICSC) - European STAMP Workshop & Conference (ESWC) (ICSC-ESWC 2018); 2019: EDP Sciences. https://doi.org/10.1051/matecconf/201927301007

21. Kim S-A, Kim E-M, Lee J-R. Causes of nurses' second victim distress: An objective analysis. Quality Management in Health Care. 2022;31(3):122-9. 10.1097/QMH.0000000000000330

22. Ramsey L, McHugh S, Simms-Ellis R, Perfetto K, O'Hara JK. Patient and family involvement in serious incident investigations from the perspectives of key stakeholders: a review of the qualitative evidence. Journal of Patient Safety. 2022;18(8):e1203. 10.1097/PTS.0000000000001054

23. Wu AW. Medical error: the second victim: the doctor who makes the mistake needs help too. BMJ. 2000;320(7237):726-7. https://doi.org/10.1136/bmj.320.7237.726

24. Rivera-Chiauzzi E, Finney RE, Riggan KA, et al. Understanding the second victim experience among multidisciplinary providers in obstetrics and gynecology. Journal of Patient Safety. 2022;18(2):e463-e9. 10.1097/PTS.0000000000000850

For peer review only - http://bmjopen.bmj.com/site/about/guidelines.xhtml

25. Jain G, Sharma D, Agarwal P, et al. "Second Victim" syndrome among the surgeons from South Asia. Indian Journal of Surgery. 2022;84(1):40-6. https://doi.org/10.1007/s12262-021-02793-3

26. Aubin D, King S. The healthcare environment: a perfect ecosystem for growing shame. Healthcare Quarterly. 2018;20:31-6.

27. Coughlan B, Powell D, Higgins M. The second victim: a review. European Journal of Obstetrics & Gynecology and Reproductive Biology. 2017;213:11-6. https://doi.org/10.1016/j.ejogrb.2017.04.002

28. Edrees HH, Paine LA, Feroli ER, Wu AW. Health care workers as second victims of medical errors. Pol Arch Med Wewn. 2011;121(4):101-8.

29. Harrison R, Lee H, Sharma A. A survey of the impact of patient adverse events and near misses on anaesthetists in Australia and New Zealand. Anaesthesia and Intensive Care. 2018;46(5):510-5. https://doi.org/10.1177/0310057X1804600513

30. Harrison R, Lawton R, Perlo J, Gardner P, Armitage G, Shapiro J. Emotion and coping in the aftermath of medical error. Journal of Patient Safety. 2015;11(1):28-35. 10.1097/PTS.0b013e3182979b6f

31. Harrison R, Johnson J, McMullan RD, et al. Toward Constructive Change After Making a Medical Error: Recovery From Situations of Error Theory as a Psychosocial Model for Clinician Recovery. Journal of Patient Safety. 2022;18(6):587-604. 10.1097/PTS.0000000000001038

32. Naya K, Aikawa G, Ouchi A, et al. Second victim syndrome in intensive care unit healthcare workers: A systematic review and meta-analysis on types, prevalence, risk factors, and recovery time. PLOS One. 2023;18(10):e0292108. https://doi.org/10.1371/journal.pone.0292108

33. Nydoo P, Pillay BJ, Naicker T, Moodley J. The second victim phenomenon in health care: a literature review. Scandinavian Journal of Public Health. 2020;48(6):629-37. https://doi.org/10.1177/140349481985

34. Vanhaecht K, Seys D, Schouten L, et al. Duration of second victim symptoms in the aftermath of a patient safety incident and association with the level of patient harm: a cross-sectional study in the Netherlands. BMJ Open. 2019;9(7):e029923. https://doi.org/10.1136/bmjopen-2019-029923

35. Wahlberg Å, Högberg U, Emmelin M. Left alone with the emotional surge–A qualitative study of midwives' and obstetricians' experiences of severe events on the labour ward.

For peer review only - http://bmjopen.bmj.com/site/about/guidelines.xhtml

Sexual & Reproductive Healthcare. 2020;23:100483. https://doi.org/10.1016/j.srhc.2019.100483

36. American Psychiatric Association. Trauma- and Stressor-Related Disorders. . Diagnostic and Statistical Manual of Mental Disorders (5th edn, text rev) 2022.

37. Owoc J, Mańczak M, Jabłońska M, Tombarkiewicz M, Olszewski R. Association between physician burnout and self-reported errors: meta-analysis. Journal of Patient Safety. 2022;18(1):e180-e8. 10.1097/PTS.0000000000000724

38. Pellino IM, Pellino G. Consequences of defensive medicine, second victims, and clinical-judicial syndrome on surgeons' medical practice and on health service. Updates in Surgery. 2015;67(4):331-7. https://doi.org/10.1007/s13304-015-0338-8

39. Werthman JA, Brown A, Cole I, et al. Second victim phenomenon and nursing support: An integrative review. Journal of Radiology Nursing. 2021;40(2):139-45. https://doi.org/10.1016/j.jradnu.2020.12.014

40. Quadrado ERS, Tronchin DMR, Maia FdOM. Strategies to support health professionals in the condition of second victim: Scoping review. Revista da Escola de Enfermagem da USP. 2021;55. https://doi.org/10.1590/S1980-220X2019011803669

41. Wade L, Fitzpatrick E, Williams N, Parker R, Hurley KF. Organizational interventions to support second victims in acute care settings: a scoping study. Journal of Patient Safety. 2022;18(1):e61. 10.1097/PTS.0000000000000704

42. Seys D, Scott S, Wu A, et al. Supporting involved health care professionals (second victims) following an adverse health event: a literature review. International Journal of Nursing Studies. 2013;50(5):678-87. https://doi.org/10.1016/j.ijnurstu.2012.07.006

43. Stone M. Second victim support programs for healthcare organizations. Nursing Management. 2020;51(6):38-45. 10.1097/01.NUMA.0000662664.90688.1d

44. Seys D, Panella M, Russotto S, et al. In search of an international multidimensional action plan for second victim support: a narrative review. BMC Health Services Research. 2023;23(1):816. https://doi.org/10.1186/s12913-023-09637-8

45. Neft MW. Support Methods for Healthcare Professionals Who Are Second Victims: An Integrative Review. AANA Journal. 2022;90(3).

46. Busch IM, Moretti F, Campagna I, et al. Promoting the psychological well-being of healthcare providers facing the burden of adverse events: A systematic review of second victim support resources. International Journal of Environmental Research and Public Health. 2021;18(10):5080. https://doi.org/10.3390/ijerph18105080

For peer review only

For peer review only - http://bmjopen.bmj.com/site/about/guidelines.xhtml

47. Spence GB. Workplace wellbeing programs: if you build it they may NOT come... because it's not what they really need! International Journal of Wellbeing. 2015;5 (2) 109-24. http://dx.doi.org/10.5502/ijw.v5i2.7
48. Scott SD, Hirschinger LE, Cox KR, et al. Caring for our own: deploying a systemwide second victim rapid response team. Joint Commission Journal on Quality and Patient Safety. 2010;36(5):233-40. https://doi.org/10.1016/S1553-7250(10)36038-7
49. Cooper CL, Cartwright S. An intervention strategy for workplace stress. Journal of Psychosomatic Research. 1997;43(1):7-16. https://doi.org/10.1016/S0022-3999(96)00392-3
50. Quick JD, Quick JC, Nelson DL. The theory of preventive stress management in organizations. In: Cooper CL, editor. Theories of organizational stress. 1. Oxford, UK: Oxford University Press; 1998. p. 246-68.
51. Martin A, Karanika-Murray M, Biron C, Sanderson K. The psychosocial work environment, employee mental health and organizational interventions: Improving research and practice by taking a multilevel approach. Stress and Health. 2016;32(3):201-15. https://doi.org/10.1002/smi.2593
52. Popay J, Roberts H, Sowden A, et al. Guidance on the conduct of narrative synthesis in systematic reviews. A product from the ESRC methods programme Version. 2006;1(1):b92.
53. Moher D, Liberati A, Tetzlaff J, Altman DG. Preferred reporting items for systematic reviews and meta-analyses: the PRISMA statement. Annals of Internal Medicine. 2009;151(4):264-9. https://doi.org/10.7326/0003-4819-151-4-200908180-001
54. Hoffmann TC, Glasziou PP, Boutron I, et al. Better reporting of interventions: template for intervention description and replication (TIDieR) checklist and guide. BMJ. 2014;348. https://doi.org/10.1136/bmj.g1687
55. Harrison R, Jones B, Gardner P, Lawton R. Quality assessment with diverse studies (QuADS): an appraisal tool for methodological and reporting quality in systematic reviews of mixed-or multi-method studies. BMC Health Services Research. 2021;21(1):1-20. https://doi.org/10.1186/s12913-021-06122-y
56. Johnson J, Simms-Ellis R, Janes G, et al. Can we prepare healthcare professionals and students for involvement in stressful healthcare events? A mixed-methods evaluation of a resilience training intervention. BMC Health Services Research. 2020;20(1):1-14. https://doi.org/10.1186/s12913-020-05948-2

For peer review only
For peer review only - http://bmjopen.bmj.com/site/about/guidelines.xhtml

57. Mira JJ, Carrillo I, Guilabert M, et al. The second victim phenomenon after a clinical error: the design and evaluation of a website to reduce caregivers' emotional responses after a clinical error. Journal of Medical Internet Research. 2017;19(6):e203. https://www.jmir.org/2017/6/e203/

58. Musunur S, Waineo E, Walton E, Deeds K, Levine D. When bad things happen: training medical students to anticipate the aftermath of medical errors. Academic Psychiatry. 2020;44:586-91. https://doi.org/10.1007/s40596-020-01278-x

59. Connors CA, Dukhanin V, March AL, Parks JA, Norvell M, Wu AW. Peer support for nurses as second victims: Resilience, burnout, and job satisfaction. Journal of Patient Safety and Risk Management. 2020;25(1):22-8. https://doi.org/10.1177/2516043519882517

60. Finney RE, Czinski S, Fjerstad K, et al. Evaluation of a second victim peer support program on perceptions of second victim experiences and supportive resources in pediatric clinical specialties using the second victim experience and support tool (SVEST). Journal of Pediatric Nursing. 2021;61:312-7. https://doi.org/10.1016/j.pedn.2021.08.023

61. Klatt TE, Sachs JF, Huang C-C, Pilarski AM. Building a program of expanded peer support for the entire health care team: no one left behind. The Joint Commission Journal on Quality and Patient Safety. 2021;47(12):759-67. https://doi.org/10.1016/j.jcjq.2021.08.010

62. Moran D, Wu AW, Connors C, et al. Cost-benefit analysis of a support program for nursing staff. Journal of Patient Safety. 2020;16(4):e250-e4. 10.1097/PTS.0000000000000376

63. Morris D, Sveticic J, Grice D, Turner K, Graham N. Collaborative approach to supporting staff in a mental healthcare setting:"always there" peer support program. Issues in Mental Health Nursing. 2022;43(1):42-50. https://doi.org/10.1080/01612840.2021.1953651

64. Rivera-Chiauzzi EY, Smith HA, Moore-Murray T, et al. Healing our own: a randomized trial to assess benefits of peer support. Journal of Patient Safety. 2022;18(1):e308-e14. 10.1097/PTS.0000000000000771

65. Thompson M, Hunnicutt R, Broadhead M, Vining B, Aroke EN. Implementation of a certified registered nurse anesthetist second victim peer support program. Journal of

For peer review only

For peer review only - http://bmjopen.bmj.com/site/about/guidelines.xhtml

Perianesthesia Nursing. 2022;37(2):167-73. e1. https://doi.org/10.1016/j.jopan.2021.05.005

66. Wijaya MI, Mohamad AR, Hafizurrachman M. Second victim support program and patient safety culture: A quasi experimental study in Bali International Medical Centre (BIMC) Hospital. Bali Medical Journal. 2018;7(1):220-6.

67. Bell SK, Moorman DW, Delbanco T. Improving the patient, family, and clinician experience after harmful events: the "when things go wrong" curriculum. Academic Medicine. 2010;85(6):1010-7. 10.1097/ACM.0b013e3181dbedd7

68. Padilla V, Healey J, Salinas J, Bryant C, Muñoz O, Durand D. Residents impacted by adverse events: the development of a pilot committee for responding to and supporting psychiatry residents. Academic Psychiatry. 2021;45:463-6. https://doi.org/10.1007/s40596-020-01348-0

69. Janes G, Harrison R, Johnson J, Simms-Ellis R, Mills T, Lawton R. Multiple meanings of resilience: Health professionals' experiences of a dual element training intervention designed to help them prepare for coping with error. Journal of Evaluation in Clinical Practice. 2022;28(2):315-23. https://doi.org/10.1111/jep.13555

70. Austin DM, Pauley G, Ferkins L. Establishing a Guide for Developing Organizational Support in Healthcare Following a Critical or Sentinel Event. Qualitative Health Research 2022;32(11):1607-19. https://doi.org/10.1177/10497323221111644

71. Cobos-Vargas A, Pérez-Pérez P, Núñez-Núñez M, Casado-Fernández E, Bueno-Cavanillas A. Second Victim Support at the Core of Severe Adverse Event Investigation. International Journal of Environmental Research and Public Health. 2022;19(24):16850. https://doi.org/10.3390/ijerph192416850

72. Merandi J, Liao N, Lewe D, et al. Deployment of a second victim peer support program: a replication study. Pediatric Quality & Safety. 2017;2:e031(4). 10.1097/pq9.0000000000000031

73. Merandi J, Winning AM, Liao N, Rogers E, Lewe D, Gerhardt CA. Implementation of a second victim program in the neonatal intensive care unit: an interim analysis of employee satisfaction. Journal of Patient Safety and Risk Management. 2018;23(6):231-8. https://doi.org/10.1177/2516043518809457

74. Krzan KD, Merandi J, Morvay S, Mirtallo J. Implementation of a "second victim" program in a pediatric hospital. American Journal of Health-System Pharmacy. 2015;72(7):563-7. https://doi.org/10.2146/ajhp140650

For peer review only

For peer review only - http://bmjopen.bmj.com/site/about/guidelines.xhtml

75. Finney RE, Jacob A, Johnson J, Messner H, Pulus B, Sviggum H. Implementation of a Second Victim Peer Support Program in a Large Anesthesia Department. AANA Journal. 2021;89(3):235-44.
76. El Hechi MW, Bohnen JD, Westfal M, et al. Design and impact of a novel surgery-specific second victim peer support program. Journal of the American College of Surgeons. 2020;230(6):926-33. https://doi.org/10.1016/j.jamcollsurg.2019.10.015
77. Keyser EA, Weir LF, Valdez MM, Aden JK, Matos RI. Extending peer support across the military health system to decrease clinician burnout. Military Medicine. 2021;186(Supplement_1):153-9. https://doi.org/10.1093/milmed/usaa225
78. Dukhanin V, Edrees HH, Connors CA, Kang E, Norvell M, Wu AW. Case: a second victim support program in pediatrics: successes and challenges to implementation. Journal of Pediatric Nursing. 2018;41:54-9. https://doi.org/10.1016/j.pedn.2018.01.011
79. Edrees H, Connors C, Paine L, Norvell M, Taylor H, Wu AW. Implementing the RISE second victim support programme at the Johns Hopkins Hospital: a case study. BMJ Open. 2016;6(9):e011708. https://doi.org/10.1136/bmjopen-2016-011708
80. Trent M, Waldo K, Wehbe-Janek H, Williams D, Hegefeld W, Havens L. Impact of health care adversity on providers: Lessons learned from a staff support program. Journal of Healthcare Risk Management. 2016;36(2):27-34. https://doi.org/10.1002/jhrm.21239
81. Schrøder K, Bovil T, Jørgensen JS, Abrahamsen C. Evaluation of'the Buddy Study', a peer support program for second victims in healthcare: A survey in two Danish hospital departments. BMC Health Services Research. 2022;22(1):1-10. https://doi.org/10.1186/s12913-022-07973-9
82. Smith BW, Dalen J, Wiggins K, Tooley E, Christopher P, Bernard J. The brief resilience scale: assessing the ability to bounce back. International Journal of Behavioral Medicine. 2008;15:194-200. 10.1080/10705500802222972
83. Riley MR, Mohr DC, Waddimba AC. The reliability and validity of three-item screening measures for burnout: Evidence from group-employed health care practitioners in upstate New York. Stress and Health. 2018;34(1):187-93. https://doi.org/10.1002/smi.2762
84. Judge TA, Scott BA, Ilies R. Hostility, job attitudes, and workplace deviance: test of a multilevel model. Journal of Applied Psychology. 2006;91(1):126-38. https://doi.org/10.1037/0021-9010.91.1.126
85. Burlison JD, Scott SD, Browne EK, Thompson SG, Hoffman JM. The second victim experience and support tool (SVEST): validation of an organizational resource for

For peer review only

For peer review only - http://bmjopen.bmj.com/site/about/guidelines.xhtml

assessing second victim effects and the quality of support resources. Journal of Patient Safety. 2017;13(2):93. 10.1097/PTS.0000000000000129

86. Sorra J, Nieva V. Hospital survey on patient safety culture. . Rockville, MD: Agency for Healthcare Research and Quality (US); 2004. Contract No.: 04-0041

87. Bisson JI, Olff M. Prevention and treatment of PTSD: the current evidence base. European Journal of Psychotraumatology. 2021;12(1):1824381. https://doi.org/10.1080/20008198.2020.1824381

88. Hargrove MB, Quick JC, Nelson DL, Quick JD. The theory of preventive stress management: a 33-year review and evaluation. Stress and Health. 2011;27(3):182-93.

89. Tetrick LE, Winslow CJ. Workplace stress management interventions and health promotion. Annu Rev Organ Psychol Organ Behav. 2015;2(1):583-603. https://doi.org/10.1146/annurev-orgpsych-032414-111341

90. Kelloway EK, Barling J. Leadership development as an intervention in occupational health psychology. Work & Stress. 2010;24(3):260-79. https://doi.org/10.1080/02678373.2010.518441

91. Montano D, Hoven H, Siegrist J. Effects of organisational-level interventions at work on employees’ health: a systematic review. BMC Public Health. 2014;14(1):1-9. https://doi.org/10.1186/1471-2458-14-135

92. Lamontagne AD, Keegel T, Louie AM, Ostry A, Landsbergis PA. A systematic review of the job-stress intervention evaluation literature, 1990–2005. International Journal of Occupational and Environmental Health. 2007;13(3):268-80. https://doi.org/10.1179/oeh.2007.13.3.268

93. Bajorek Z, Bevan S. Demonstrating the effectiveness of workplace counselling: Reviewing the evidence for wellbeing and cost-effectiveness outcomes. https://www.employment-studies.co.uk/system/files/resources/files/553.pdf. Institute for Employment Studies 2020. Report No.: 553

94. Mengstie MM. Perceived organizational justice and turnover intention among hospital healthcare workers. BMC Psychology. 2020;8:1-11. https://doi.org/10.1186/s40359-020-0387-8

95. Eisenberger R, Karagonlar G, Stinglhamber F, et al. Leader–member exchange and affective organizational commitment: The contribution of supervisor's organizational embodiment. Journal of Applied Psychology. 2010;95(6):1085-103. https://doi.org/10.1037/a0020858

For peer review only - http://bmjopen.bmj.com/site/about/guidelines.xhtml

96. Östergård K, Kuha S, Kanste O. Health-care leaders' and professionals' experiences and perceptions of compassionate leadership: A mixed-methods systematic review. Leadership in Health Services. 2023;37(5):49-65. https://doi.org/10.1108/LHS-06-2023-0043

97. de Zulueta P. How do we sustain compassionate healthcare? Compassionate leadership in the time of the COVID-19 pandemic. Clinics in Integrated Care. 2021;8:100071.

98. Panagioti M, Khan K, Keers RN, et al. Prevalence, severity, and nature of preventable patient harm across medical care settings: systematic review and meta-analysis. BMJ. 2019;366:l4185. https://doi.org/10.1136/bmj.l4185

99. Donaldson MS, editor. An overview of to err is human: re-emphasizing the message of patient safety. Rockville (MD): Agency for Healthcare Reseach and Quality (US); 2011.

For peer review only

Figure 1: PRISMA flowchart of stages in the review

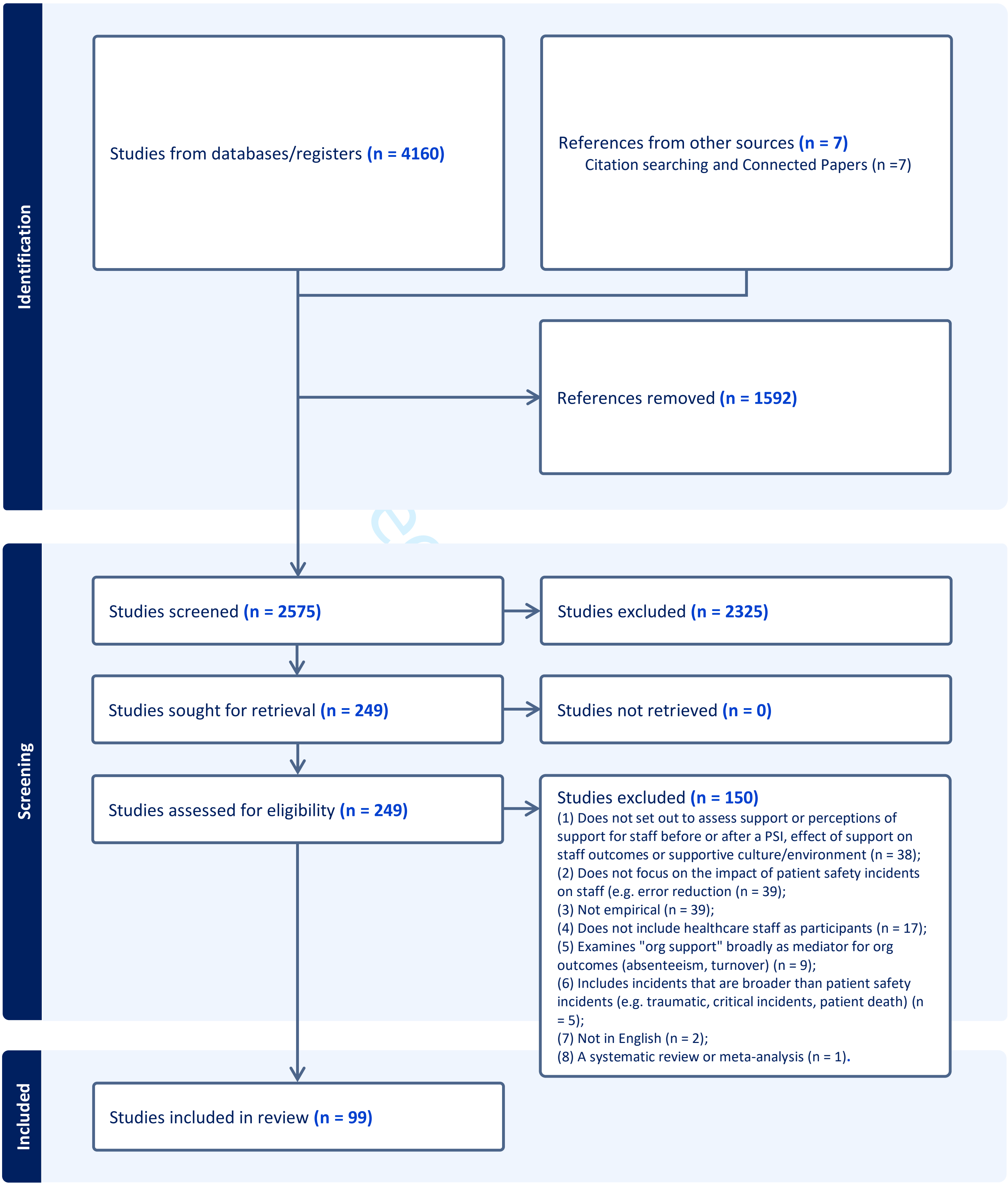

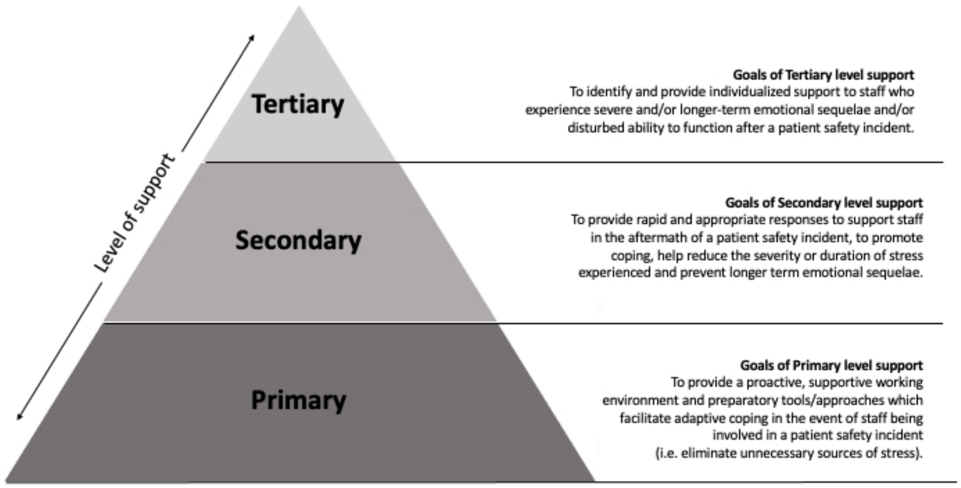

Figure 2: Organisational staff support model for Patient Safety Incidents

338x190mm (54 x 54 DPI)

Supplementary File 2: Search criteria for Medline

1 ((critical or patient safety or harmful or clinical) adj incident*).mp.
2 ((avoidable or preventable or unsafe or undesirable) adj1 (event* or outcome* or complication* or death or accident* or injur*)).mp.
3 (serious incident? or critical incident? or never event? or near miss* or sentinel event? or adverse patient occurrence? or serious reportable event? or (serious adj2 event? adj3 (clinical or critical or medical or surgical)) or preventable adverse event? or avoidable adverse event? or ((Medical or surgical) adj2 Adverse adj2 Event?)).mp.
4 1 or 2 or 3 or exp medical errors/
5 ((Health adj2 professional*) or (health adj2 provider*)).mp. or exp health personnel/ or healthcare profession*.mp. or (healthcare adj2 provider*).mp. or healthcare worker*.mp. or health care worker*.mp. or (worker* adj2 (health or healthcare)).mp. or nurs*.ti,ab. or physician*.ti,ab. or surgeon.ti,ab. or midwive*.ti,ab. or clinician*.ti,ab.
6 (4 and 5) or second victim*.af.
7 exp social support/
8 exp Occupational Health Services/
9 Crisis intervention/
10 Counseling/
11 Stress, Psychological/pc, th or Stress Disorders, Post-Traumatic/pc, th
12 support*.ti,kw,kf.
13 ("critical incident stress management" or "critical incident stress reporting system*" or "critical incident stress debrief*").mp.
14 ("Second Victim Experience and Support Tool" or SVEST).mp.
15 (peer group* or (peer* adj3 support*)).af.
16 Debrief*.mp.
17 (support* adj2 (second victim* or organi?ation* or service* or system* or program* or resource* or social)).mp.
18 ((Resilienc* adj2 training) or (resilienc* adj2 intervention*) or (promot* adj2 resilienc*)).mp.
19 "Schwartz Center Rounds".ti,ab,kf,kw.
20 Resilience, psychological/
21 ("Intervention training" or (crisis adj2 training)).mp.
22 "Resilience In Stressful Events".mp.
25 ((Institution* adj2 support) or (Institution* adj2 response*) or (Institution* adj2 respond*)).mp.
26 Simulation Training/
27 (safe* adj2 culture*).mp.
28 psychologic* safe*.mp.
29 just culture*.mp.
30 non-punitive.mp.
31 *organizational culture/
32 organizational culture/ and (px or ed or pc or mt or og).fs.
23 6 and 7
24 6 and 8
33 6 and 9
34 6 and 10
35 6 and 11
36 6 and 12
37 6 and 13
38 6 and 14
39 6 and 15
40 6 and 16
41 6 and 17
44 6 and 18
45 6 and 19
46 6 and 20

47 6 and 21
48 6 and 22
49 6 and 23
50 6 and 24
51 6 and 25
52 6 and 26
53 6 and 27
54 6 and 28
55 6 and 29
56 6 and 30
57 6 and 31
58 6 and 32
59 or/33-58
60 limit 59 to (abstracts and english language and yr="2010 -Current")

For peer review only

Supplemental Table: Most desired compared with most experienced forms of support for coping with patient safety incidents

Most desired	No of papers in sample *(In Top 3 of surveys; In qual. or mixed methods papers)*	Most experienced	No of papers in sample *(In Top 3 of surveys; In qual. or mixed methods papers)*
**1.** Peer support	**N=28** *(n=16; n=12)*	**1.** Peer support	**N=46** *(n=8; n=38)*
**2.** Practical support and guidance	**N=27** *(n=7; n=20)*	**2.** Managerial support	**N=23** *(n=5; n=18)*
**3.** Professional mental health support	**N=21** *(n=8; n=13)*	**3.** Post-incident debrief to clarify, process	**N=15** *(n=3; n=12)*
**4.** Post-incident debrief to clarify, process	**N=18** (n=8; n=10)	**4.** Professional mental health support	**N=11** *(n=0; n=11)*
**5.** Supportive, learning culture	**N=14** *(n=1; n=13)*	**5.** Mortality and Morbidity meeting	**N=7** *(n=3; n=4)*

Key: Primary Level Support | Secondary Level Support | Tertiary Level Support

Supplementary 3 File: Narrative summary findings from surveys and qualitative studies included at Stage 1 (n=72)

1. What support do staff want for coping with Patient Safety Incidents (RQ1)?

1.1 Surveys (n=49; papers 1-49)

Thirty-four survey studies explored the support that staff would want following an adverse event to help second victims recover (1, 3, 4, 5, 7, 8, 9, 10, 12, 14, 15, 16, 17, 18, 19, 20, 24, 25, 26, 27, 29, 31, 32, 34, 35, 36, 37, 38, 41, 42, 44, 45, 47, 48).

Peer support was an aspect that arose in many survey studies (n = 23) and was rated as the most desired support strategy by every study that ranked supports (1, 3, 4, 10, 15, 16, 18, 19, 27, 32, 38, 41, 44, 45, 46, 47). Of the survey studies that gave figures, between 59% and 92.1% of the samples would want peer support following an event (4, 15, 18, 19, 27, 41, 44, 45, 47). However, the term 'peer support' was used differently between studies. It was primarily referred to as 'a respected peer to discuss the details of what happened' in 9 of the 23 studies (4, 16, 28, 33, 38, 39, 45, 46, 47); other definitions included emotional support (8, 9), conversations with peers (10, 19, 20), confidential support (17), talking to someone (32), discussing emotional issues (36, 37) or support from close people on a professional or personal level (42, 43). There were differences in who second victims wanted to receive peer support from, with some preferring conversations with direct colleagues (1, 3, 8, 41, 42) and others desiring to speak with the whole team (18, 31, 41) or other physicians (3). One survey study found that informal emotional support was one of the least wanted methods of support despite being one of the most utilised (7).

There were a variety of forms of support provided by organisations for second victims that were referenced across the survey studies. Of the survey studies that ranked strategies, the support that was rated in the top 3 by the second most studies was having 'a specified peaceful location that is available to recover and recompose' (4, 9, 27, 32, 44, 45, 47), with between 61.4% and 78.8% of samples wanting this support. Debriefs with the colleagues involved in the incident were wanted by some participants (7, 14, 16, 18, 35, 36, 48), although it was not always clear how this was different to peer support. Multiple survey studies found that second victims wanted information about the incident, including ethical issues (7, 35), causes and preventative measures (7, 26, 29, 34, 35, 36, 41). Several participants were found to desire crisis interventions (7, 16, 35, 36),

For peer review only

including guidance and advice regarding legal proceedings following the event (7, 16, 26, 34, 36).

Counselling was desired as a method of support for second victims in thirteen survey studies (1, 7, 14, 15, 16, 17, 19, 25, 27, 34, 35, 36, 37). Of the survey studies that ranked support strategies, counselling was rated in the top 3 by the joint third most studies (1, 16, 19, 25, 37, 38), with between 29.9% and 96.1% of samples desiring this support. However, one sample found it to be the least desired of the strategies (4).There were inconsistencies in how this was phrased; some participants wanted to be supported by a professional and receive formal support (1, 7, 14, 15, 16, 18, 19, 24, 25) whilst others simply wanted access to formal psychological services or the opportunity to see a counsellor if they felt like they needed it (4, 7, 17, 34, 35, 36, 37). Similarly, employee assistance programs were sought in some studies, which could provide support to second victims outside the workplace (16, 28, 35, 38, 48).

1.2 <u>Qualitative studies (n=23; papers 50-72)</u>

Eighteen qualitative studies explored staff needs following an adverse event and what support they believed would be most appropriate in helping second victims recover (50, 51, 52, 53, 54, 56, 70, 71, 57, 58, 59, 62, 63, 64, 65, 66, 67, 68). Of these, thirteen studies reported staff as wanting their organisation‘s approach and support to accommodate them and the incident (51, 53, 54, 56, 70, 71, 58, 59, 62, 63, 66, 67, 68), such as being protected, recognised and avoiding blame or criticism (51, 54, 70, 59). Across the qualitative studies, organisational support also included being freed from workloads, reviewing the staff member's responsibilities (54), allowing tasks and responsibilities to be completed with a colleague (62) and follow-up or long-term support (70), no time limit on recovery (68) recognition that the impact of an event may differ over time (53, 67). Several qualitative/other studies reported that second victims may not seek support even if needed, so organisations and supervisors need to be proactive in reaching out (52, 64). Some staff emphasised a necessity for professional reassurance in emotional recovery (56, 67), with second victims wanting guidance, reassurance and an awareness of management's confidence in them (54, 66).

Staff from twelve qualitative studies desired emotional support and having someone to talk to (54, 70, 57, 66), with eight specifying peers and colleagues as a desired audience (52, 53, 56, 71,

For peer review only - http://bmjopen.bmj.com/site/about/guidelines.xhtml

58, 59, 63, 67, 68) and three looking for managerial support (64, 66, 68). Staff described the ideal supporter as a non-judgemental and empathetic colleague from their direct work environment who could acknowledge and reassure the second victim (70, 58, 67). Structural peer support was suggested as a strategy (52, 63), such as colleagues assisting the second victim with workload or taking time to listen (54), with one study finding that informal peer support was the most desired support method (68).

Eight samples of staff were found to seek formal support, counselling and/or specialist mental health resources (50, 51, 53, 71, 57, 62, 63, 68), which could be accessed by those who had been seriously impacted by an incident (59, 68). Staff across several studies recommended confidential counselling programs provided directly by the organisation (51, 71, 63), although one study reported that there were mixed views about whether this would be accepted within surgical communities (62).

Staff from further six qualitative studies wanted time away from work or a period of leave to help distance themselves from the event and recover (51, 54, 59, 62, 66, 68). Furthermore, six studies found staff seeking practical support in the form of debriefs and discussions (70, 71), administrative support with investigations (56, 57) as well as information, evaluation and learning around the incident (56, 70, 67, 68). Some staff expressed a need to be supported in communicating with the affected patient or family following an incident (62, 68) and be informed of the patient's outcomes (56). Samples from multiple qualitative studies also suggested value in sharing their experiences with other colleagues to share learning around the second victim experience (70, 63, 68).

1.3 Additional information from qualitative studies related to what staff want

- Emotional support following an adverse event was described as the "first measure" following an incident (54) and essential in effectively supporting second victims (54, 57, 59, 66, 67).
- One study emphasised that support should focus on the staff's emotions instead of the cause of the incident (59).
- Many staff across studies reported the need for second victims to feel heard, and that psychosocial support should address the need for them to talk (54, 70, 63, 67).

For peer review only - http://bmjopen.bmj.com/site/about/guidelines.xhtml

- Second victims were reported to need general concern and empathy around wellbeing from colleagues and superiors (71).
- One study's sample suggested the implementation of a mentoring system, giving staff an opportunity to talk with seniors for problem solving (62).
- Across the studies, staff reported wanting access to psychological support and specialist resources (52, 57, 62, 63, 66).
- Some participants wanted administrative and institutional support to help individuals overcome psychological difficulties, and address adverse events and countermeasures (57, 67).
- Multiple participants raised a concern that the culture of healthcare services served as a barrier for blame-free approaches; environments were described as inconsiderate to emotional wellbeing (71) and vulnerability (58) post-incident, and a shift to a caring, gossip-free culture was needed at organisational and individual levels (58, 59). Leadership is needed to promote a safe atmosphere to process patient safety incidents (71), and one study suggested that communicative skills such as empathy should be essential when hiring senior members of staff (58). Another study reported that learning can only take place in a psychologically safe setting (65).
- Although studies noted a variation in the amount of support needed by second victims, recognition of their actions and collegial trust were reported as the minimum level required (56). Investigations into adverse events were found as a potential barrier to recovery, as they could create a sense of anxiety for second victims (56). Therefore, it was important for staff to receive guidance and support for the investigation process and for supervisors to emphasise that the investigations are for system failures, not punishment (56, 67, 68). One study reported a need to discuss working conditions, as this was not always considered during investigations, which was frustrating for the staff involved (67).
- Administrative and practical needs were also identified in various studies. Some staff reported confusion about the post-incident procedure, thereby necessitating organisational guidance on this process (56, 68).
- One study found that the ideal listener differed depending on the culture of the organisation; in 'open culture' organisations, staff preferred talking to internal colleagues, whereas staff in 'blame cultures' preferred an external individual with the same professional background (68).

For peer review only - http://bmjopen.bmj.com/site/about/guidelines.xhtml

A minority of participants from another study suggested that internal peer support may tamper with team relations, and that organisations should have a general, hospital-wide support service, although other participants argued that this was ineffective and a reflection of the hospital culture being insensitive to vulnerability (58). Several studies advocated for peer support being organisation-led instead of relying on individuals to take initiative (51, 71, 67), such as by stimulating intercollegiate conversations around support following an event (58). Follow-up conversations were also suggested by one study in order to account for differing long-term needs (52).

- One study suggested that counselling could be mandatory, as second victims need to address and open up about the event to be competent in the role moving forward (50), with another noting the potential prevalence of PTSD (57).

2. What support do staff receive to cope with Patient Safety Incidents (RQ2)?

2.1 __Surveys (n=49; papers 1-49)__

Thirty-four survey studies explored the support that staff experienced following an adverse event (1, 2, 3, 5, 6, 8, 10, 11, 12, 13, 16, 17, 20, 21, 22, 23, 24, 28, 30, 31, 33, 35, 36, 38, 39, 40, 41, 42, 43, 45, 46, 47, 48, 49).

Peer support was addressed in twenty-five studies (2, 3, 6, 8, 10, 11, 12, 13, 16, 17, 20, 21, 22, 23, 24, 30, 33, 35, 36, 38, 39, 41, 42, 49) and was rated as both the most experienced (8, 12, 21, 30, 35, 38, 41, 49) and most useful (6, 8, 11, 13, 16, 17, 21) by the majority of studies that ranked supports. Most of the studies that gave figures, reported between 49% and 100% of their samples experiencing peer support (12, 21, 30, 33, 35, 38, 39, 42, 49), although peer support was experienced by less than 1% of samples in two other studies (45, 47). Second victims were found to talk to direct colleagues (1, 2, 3, 21, 22, 23, 24, 35, 39, 41, 49), especially those at a similar rank (13, 20, 41) and those directly involved in the incident (41). Second victims seeking emotional support were primarily looking for peers who could understand the situation in order to manage their own stress, bring relief and prevent burnout (2, 6, 10, 49). Where effectiveness was measured, peer support was largely seen as useful and helpful, including in studies using statistical analyses (2, 6, 8, 17, 20, 35, 36) and quantitative analysis (12), and between 42% and

For peer review only - http://bmjopen.bmj.com/site/about/guidelines.xhtml

94.1% of samples found peer support to be useful. Conversely, one statistical sample reported mixed ratings (33).

Participants also reported experiencing a variety of institutional support strategies. Receiving support from supervisors and department leaders was commonly referenced (6, 13, 24, 30, 33, 35, 42, 43, 45, 49) and was found to be both the second most experienced and second most useful support in studies that ranked strategies (6, 13 24, 42, 43). Managerial support was rated as valuable in several studies (6, 13, 43), whilst others found it to be mixed (33) or insufficient (42, 45, 47). Mortality and Morbidity meetings or patient safety incident discussions were referenced in several studies (2, 17, 28, 43, 49), and were the joint second most commonly experienced support amongst studies that ranked strategies (3, 43, 49), with 67.3% of one sample finding them to be helpful for second victims (28). Debriefs were also experienced by staff (3, 8, 17, 35, 48) and were ranked as the joint second most helpful, with 89.4% to 100% of samples finding these to be useful (17, 35, 36, 48). However, others found the meetings to be unsupportive, neutral or negative to recovery (10, 28, 43). Counselling was also addressed in multiple studies and was reported as useful (11, 17, 20, 35), albeit less experienced than other support options (35, 49).

2.2 <u>Qualitative studies (n=23; papers 50-72)</u>

All twenty-three qualitative studies explored the support that staff received following a patient safety incident. Of the ten that reviewed the support provided, all found it to be insufficient or inconsistent (50, 53, 56, 70, 58, 61, 62, 63, 66, 67). One study reported 82% of its staff as having no support and an unfriendly atmosphere (63), whilst staff in another reported that 65% staff did not have all needs addressed, and that 37% had no support whatsoever (70). Support varied both between and within roles; several studies reported a lack of guidance or a consistent process (53, 69, 56, 70, 58, 61, 67), staff being unaware of the support available (70, 58, 61, 63, 66) and certain staff receiving different levels of support based on their position (52, 58, 64, 68).

Twenty-one studies reported experiences with peer support (50, 51, 52, 53, 55, 69, 56, 70, 71, 57, 58, 59, 60, 62, 72, 63, 64, 65, 66, 67, 68), and it was commonly utilised or seen as helpful in all studies when done in the right way. However there is definitely inconsistency in the way peer support is considered as a

For peer review only - http://bmjopen.bmj.com/site/about/guidelines.xhtml

'support': some studies imply that it is facilitated by the organisation, yet others imply that it is different from organisational support, and simply involves SVs talking to colleagues.

Seven viewed peer support as the most effective form of support or the support that they would typically/first turn to following an event (53, 70, 71, 62, 63, 64, 67). In several studies, the experience of the listening colleague was crucial to the effectiveness of peer support, with the ideal listener being able to fully understand the nature of the job and the incident itself (50, 69, 71, 57, 59, 60, 62, 72, 65, 66, 67, 68). Multiple participants also emphasised that to best support them, the listener should be non-judgemental and refrain from criticising or blaming the second victim (52, 66, 68). As well as providing emotional support (53), many of the healthcare staff members found that peer support could help the second victim with practical advice and reassure them about their capability in the role, which helped them in regaining professional confidence (53, 58, 59, 60, 62, 72, 65, 68). Other studies noted the benefits of peers sharing mistakes of their own (51, 72) and checking in with the second victim generally (51). Support from other second victims was reported as helpful, but only if they were understanding and non-judgemental of the event (68). Conversely, several participants emphasised that peer support had to be conducted in the right way, as colleagues who were too critical or judgmental (53, 59, 60, 65, 68), minimised the event (53, 60, 72, 68) or were overly reassuring (60, 68) could make the second victims feel worse. Attempts to solve the error (72), peers reaching out due to curiosity rather than compassion (68) and small comments that could make second victims feel in the wrong (65) were also seen as barriers to successful peer support.

Twelve qualitative studies addressed managerial support (50, 52, 69, 70, 71, 59, 60, 61, 63, 65, 66, 67), with four finding it to be helpful (52, 71, 59, 66) yet six others finding it to be insufficient (50, 60, 61, 67) or inconsistent (52, 70). In one study, 22% of participants said that informal emotional support from supervisors was the most helpful strategy, whilst 15% found informal emotional support from managers to be best (although 12% also said this was the least helpful support) (70). Examples of effective managerial support included giving staff space without chasing up incident reports (59), celebrating staff in team meetings to facilitate positivity, providing support via team meetings or individual conversations and generally being proactive and accessible after an event (52), which helped to resolve emotional stress (71, 59). Ineffective managerial support led to feelings of isolation (50, 60) and hindered recovery (61, 67)

For peer review only - http://bmjopen.bmj.com/site/about/guidelines.xhtml

Other organisational support methods included debriefs and other discussions around the event, which were referenced by six studies (52, 70, 57, 62, 64, 65). Four of these samples found debriefs to be ineffective due to inconsistencies in their implementation (70, 62, 65), certain staff not being involved and focusing on learning and blaming rather than emotional recovery (52, 65). In one study, 'defusing' was suggested, which was distinctive from formal debriefs and allowed for staff to voice their reactions and prepare for debrief (52). One group of participants referenced Mortality and Morbidity meetings, finding them to be ineffective due to a focus on learning and some staff not being invited (64). Learning was referenced by another six studies (55, 70, 60, 62, 72, 68), with staff finding meaning in discussing the case and evaluating what went wrong (55, 62, 68), teaching others about their experience (60, 68) and developing changes to prevent future incidents (70, 72). Some staff sought help with the administrative and legal aspects of an incident, which was appreciated (68).

Six studies referred to counselling and formal support (50, 55, 56, 70, 63, 68) which was used by staff in five (55, 56, 70, 63, 68). This was seen as helpful in two studies due to it being personalised, objective, empathetic and non-judgmental (56, 68), but seen as the least helpful support by 11% of another sample (70). When these services were not offered by the organisation, participants sought professional help themselves, with 40% of one sample seeking this (63). Taking time away from work helped staff distance themselves from the event in one study (50), but was refused by the organisation in another (54).

2.3 <u>Additional information from qualitative studies related to what staff received</u>

- Certain staff were not invited to Morbidity and Mortality meetings (64) therefore this was seen as an inadequate approach to support all those involved in a PSI.
- In many studies, peer support was seen as helpful for recovery and the emotional wellbeing of the staff involved (51, 52, 53, 55, 69, 56, 70, 71, 58). For some participants, informal peer support helped them feel heard (50, 72). Several studies reported that peer support could be detrimental to the recovery of second victims if done in the wrong way. In one paper, one third of participants experienced issues with their collegial relationships as a result of the failure of peer support, which sometimes led to dispute in the organisation (67). The failure to support the second victim generally was also found to damage working relationships and

For peer review only - http://bmjopen.bmj.com/site/about/guidelines.xhtml

leave the individual feeling unsupported and anxious (56). Multiple studies noted that colleagues 'minimising' the situation did not help, and that it was important for peers to be open and honest about the incident (53, 60, 72). Studies reported that conversations needed to address the coping needs of the physician at the time (60), and that physicians responded best to peers who listened attentively and showed empathy (72). Negative examples of peer support included criticism, blame, being made to feel incompetent (53, 60), attempts to solve the error (72) and peers reaching out due to curiosity, not compassion (68). In one case, small comments were enough to make second victims feel in the wrong (65). Being protected by friends (60) and having colleagues who were resistant to discussion (68) were also barriers to successful peer support.

- In one study, 86% of participants said they would typically turn to a colleague, and 48% received emotional intercollegiate support following a patient safety incident (70), with 69% seeking peer support in another study (71). and receiving empathy from colleagues was seen as beneficial (51, 59).
- Multiple findings emerged concerning the lack of formal structures in place in some institutions, meaning support was based on the individuals around the second victim (70, 61). The absence of structure was attributed as a source of staff isolation by one study (67).
- Some of the inconsistencies described across the support received were related to the positions of the staff involved. Some participants reported feeling unrepresented in the support process; one study found that support for nurses and ODPs was inadequate in comparison to clinicians involved, and that certain staff were not invited to Morbidity and Mortality meetings (64). In one case, healthcare professionals were excluded from investigations about their incidents, which was detrimental to their recovery (68). One study found that clinicians felt unrepresented in their organisation's support service, and that the social science professionals that worked in the service would be unable to understand the clinical context (58). A lack of conversation and discussion about an incident was sometimes reported (67), and the silence served as a barrier to reflection and learning (60). One study found many participants perceiving support as unengaging and minimal, leading to feelings of isolation (50). Another described organisational approaches as 'punitive' and inconsiderate to the systemic factors that contribute to incidents (62). Regarding legality, one study reported that rules and sanctions were barriers to support, and that organisational

For peer review only - http://bmjopen.bmj.com/site/about/guidelines.xhtml

interpretations of these previsions were biased against second victims (61). Conversely, one paper reported staff being supported and taken seriously by the organisation, with the service listening and guiding staff through an event (64).

- Several studies addressed the emotional element involved in support. One study found it to be the most relevant concern following an incident, and that its absence led to isolation for second victims, although it could also be unwanted by certain staff or in certain approaches, such as when it was too soothing or came from someone with minimal experience (68). For some, confidentiality rules made staff reliant on workplace support by preventing them from talking to outsiders, meaning that insufficient workplace support led to feelings of abandonment (65, 67). Emotional support contrasted with Morbidity and Mortality meeting and more practical responses to incidents (72) and was helpful, but organisations sometimes lacked in guidance around it (69) – some staff were unaware of who they could talk to, leading to ineffective support that 85% of one sample was unsatisfied with (63). A lack of emotional support was found to be one of the least helpful responses identified by one study (70).
- Some healthcare staff also turned to managers and supervisors in response to a patient safety incident. In one sample of student nurses, professors were the 'first and primary support' accessed (66), and several studies addressed participants reaching out to managers, supervisors or risk management for support (70, 71). In one study, 22% of participants said that informal emotional support from supervisors was the most helpful strategy, whilst 15% found informal emotional support from managers to be best (although 12% also said this was the least helpful support) (70). Effective support from managers helped to resolve emotional stress (71, 59), facilitate learning from the incident and provide reassurance surrounding competence (60). Several studies reported ineffective managerial support (67), resulting in second victims feeling unsupported and abandoned (50, 60, 66). Proactivity of managers was reported as beneficial in one paper, and included providing support via team meetings, individual conversations, formal peer support and generally being accessible after an event (52). Reactive or passive managers were reported to be too late or non-existent in their support, hindering second victim recovery (52, 61). Other examples of effective managerial support included giving staff space without chasing up incident reports (59) and celebrating staff in team meetings, facilitating trust and a culture of support (52). 'Defusing' was also

For peer review only - http://bmjopen.bmj.com/site/about/guidelines.xhtml

suggested, which was distinctive from formal debriefs, allowed for staff to voice their reactions and prepare for debrief (52).

- In some studies, debriefs were suggested as a method for staff to feel less isolated, more supported and help coping generally (64, 65), with incidents being discussed through seniors meetings and interrogations (57). However, the frequency and quality of debriefs varied widely. Debriefs were reported as rare in some studies (62), with only 13% of participants in one study receiving them promptly (70). One study found them to be inconsistent in frequency (sometimes too few, sometimes too many) and participation, with some staff not being invited or deciding not to attend (65). For second victims, this limited the effectiveness of the debriefs and their quality overall, preventing healthcare professionals from receiving guidance. In one case, midwives were found to commonly be excluded from investigations and interprofessional debriefing, with many of them subsequently perceiving investigations as scapegoating and attributing blame (52). Another study noted that debriefs sometimes turned to explicit or implicit blaming of staff for the incident (65), with a focus on learning neglecting the emotional need of second victims. Debriefs were also limited when seniors did not conduct the debriefs (as they only were expected to lead these sessions if they were involved in the incident), forcing healthcare staff to attempt conduction of the debrief themselves (65). 11% of one sample reported debriefs and crisis interventions as the least helpful support (64), and another study noted that any welfare extracted from debriefs is not beneficial to second victims unless later acted upon (64).
- Several studies addressed second victims using their experiences in incidents to facilitate learning and teach others. In one sample, 12% of staff were involved in a learning process, with 11% describing it as the most helpful support (70). Learning was enabled via intercollegiate conversations (60, 72) and analysis of the incident (62, 68), helping to facilitate processing of the incident (72). In one instance, midwives were often excluded from learning opportunities, serving as a barrier to their recovery (52). Some second victims further used their experiences and learning to teach colleagues and prevent future incidents of a similar nature (60, 72, 68). This not only gave second victims an outlet to talk about the experience, but also allowed them to develop system changes (72), and teaching helped facilitate their own recovery (60). Other suggested methods of learning included staff ensuring their skills were sufficient (62), supervision and case discussions (55). Mentoring

For peer review only - http://bmjopen.bmj.com/site/about/guidelines.xhtml

was suggested in one case, but effective mentoring was reported as rare (62). One study noted that a focus on learning (especially during Mortality and Morbidity meetings) was ineffective at supporting staff emotionally (64), with another noting that emotional vulnerability that was not dealt with served as a barrier to learning (65).

- Other methods of support were addressed by several studies, including the use of counselling or professional support. Some organisations reportedly provided staff with professional services, allowing serious impacts to be treated and time to adequately recover (50, 55, 56). When these services were not offered by the organisation, participants sought professional help themselves, with 40% of one sample seeking this (63). Counselling was beneficial for second victims as it was "personalised, objective, empathetic and non-judgemental" (68). Some staff reported that conversations with the patients and families involved in incidents was beneficial for recovery, allowing second victims to address the incident directly, apologise and find closure (60, 62, 72). Other staff sought leave to help distance themselves from the incident and recover (50), although one study reported the hospital refusing this request (54). Some nurses in one sample changed their work routines to facilitate processing of the experience, such as having back-up in case they decide they are unable to complete the shift (55). Having time for group reflection that focused on the emotional impact of an incident was suggested (71), as well as creating an atmosphere of shared responsibility and positivity (59). Some staff sought help with the administrative and legal aspects of an incident (68).
- One paper reported that a lack of support from colleagues and seniors could damage intercollegiate trust and relationships, leaving the second victim isolated and anxious (56).

Reference List: Surveys and qualitative studies included at Stage 1 (n=72)

Study ID	Study Reference	Explored what staff wanted	Explored what staff received

For peer review only - http://bmjopen.bmj.com/site/about/guidelines.xhtml

1	Baas MA, Scheepstra KW, Stramrood CA, Evers R, Dijksman LM, van Pampus MG. Work-related adverse events leaving their mark: a cross-sectional study among Dutch gynecologists. BMC Psychiatry. 2018 Dec;18:1-8.	X	X
2	Bañeras J, Jorge-Pérez P, Bonanad C, Lluva MT, Moll I, Kinori SG. Second victims and quality of support resources among cardiology professionals. Revista Espanola de Cardiologia (English ed.). 2022 Aug;75(8):686-8.		X
3	Berman L, Rialon KL, Mueller CM, Ottosen M, Weintraub A, Coakley B, Brandt ML, Heiss K. Supporting recovery after adverse events: an essential component of surgeon well-being. Journal of Pediatric Surgery. 2021 May 1;56(5):833-8.	X	X
4	Burlison JD, Scott SD, Browne EK, Thompson SG, Hoffman JM. The second victim experience and support tool (SVEST): validation of an organizational resource for assessing second victim effects and the quality of support resources. Journal of Patient Safety. 2017 Jun;13(2):93.	X	
5	Choi EY, Pyo J, Ock M, Lee H. Profiles of second victim symptoms and desired support strategies among Korean nurses: A latent profile analysis. Journal of Advanced Nursing. 2022 Sep;78(9):2872-83.	X	X
6	Draus C, Mianecki TB, Musgrove H, Bastien DJ, Greggs D, Halash C, Lewis A, Mackenzie W. Perceptions of Nurses Who Are Second Victims in a Hospital Setting. Journal of Nursing Care Quality. 2022 Apr 20;37(2):110-6.		X
7	Edrees HH, Paine LA, Feroli ER, Wu AW. Health care workers as second victims of medical errors. Pol Arch Med Wewn. 2011 Apr 1;121(4):101-8.	X	
8	Fatima S, Soria S, Esteban-Cruciani N. Medical errors during training: how do residents cope?: a descriptive study. BMC Medical Education. 2021 Dec;21(1):1-6.	X	X
9	Finney RE, Torbenson VE, Riggan KA, Weaver AL, Long ME, Allyse MA, Rivera-Chiauzzi EY. Second victim experiences of nurses in obstetrics and gynaecology: A second victim experience and support tool survey. Journal of Nursing Management. 2021 May;29(4):642-52	X	
10	Gupta K, Rivadeneira NA, Lisker S, Chahal K, Gross N, Sarkar U. Multispecialty physician online survey reveals that burnout related to adverse event involvement may be mitigated by peer support. Journal of Patient Safety. 2022 Sep 1;18(6):531-8.	X	X
11	Han K, Bohnen JD, Peponis T, Martinez M, Nandan A, Yeh DD, Lee J, Demoya M, Velmahos G, Kaafarani HM. The surgeon as the second victim? Results of the Boston Intraoperative Adverse Events Surgeons' Attitude (BISA)		X

For peer review only - http://bmjopen.bmj.com/site/about/guidelines.xhtml

	study. Journal of the American College of Surgeons. 2017 Jun 1;224(6):1048-56.		
12	Harrison R, Lee H, Sharma A. A survey of the impact of patient adverse events and near misses on anaesthetists in Australia and New Zealand. Anaesthesia and Intensive Care. 2018 Sep;46(5):510-5.	X	X
13	Harrison R, Sharma A, Walton M, Esguerra E, Onobrakpor S, Nghia BT, Chinh ND. Responding to adverse patient safety events in Viet Nam. BMC Health Services Research. 2019 Dec;19:1-8.		X
14	Heard GC, Thomas RD, Sanderson PM. In the aftermath: attitudes of anesthesiologists to supportive strategies after an unexpected intraoperative patient death. Anesthesia & Analgesia. 2016 May 1;122(5):1614-24.	X	
15	Huang R, Sun H, Chen G, Li Y, Wang J. Second-victim experience and support among nurses in mainland China. Journal of Nursing Management. 2022 Jan;30(1):260-7.	X	
16	Jain G, Sharma D, Agarwal P, Agrawal V, Yadav SK, Tenzin T, Alamgir MH, Manandhar K, Myint M, Chaudhary AM, Jami AA. “Second Victim” syndrome among the surgeons from South Asia. Indian Journal of Surgery. 2022 Feb;84(1):40-6.	X	X
17	Joesten L, Cipparrone N, Okuno-Jones S, DuBose ER. Assessing the perceived level of institutional support for the second victim after a patient safety event. Journal of Patient Safety. 2015 Jun 1;11(2):73-8.	X	X
18	Kaur AP, Levinson AT, Monteiro JF, Carino GP. The impact of errors on healthcare professionals in the critical care setting. Journal of Critical Care. 2019 Aug 1;52:16-21.	X	
19	Kerkman T, Dijksman LM, Baas MA, Evers R, van Pampus MG, Stramrood CA. Traumatic experiences and the midwifery profession: a cross-sectional study among dutch midwives. Journal of Midwifery and Women's Health. 2019 Jul;64(4):435-42.	X	
20	Khansa I, Pearson GD. Coping and recovery in surgical residents after adverse events: the second victim phenomenon. Plastic and Reconstructive Surgery Global Open. 2022 Mar;10(3).	X	X
21	Lin JS, Olutoye OO, Samora JB. To Err is human, but what happens when surgeons Err?. Journal of Pediatric Surgery. 2023 Mar 1;58(3):496-502.		X
22	Magaldi M, Perdomo JM, López-Baamonde M, Chanzá M, Sanchez D, Gomar C. Second victim phenomenon in a surgical area: online survey. Revista Española de Anestesiología y Reanimación (English Edition). 2021 Nov 1;68(9):504-12.		X

For peer review only - http://bmjopen.bmj.com/site/about/guidelines.xhtml

23	Martens J, Van Gerven E, Lannoy K, Panella M, Euwema M, Sermeus W, De Hert M, Vanhaecht K. Serious reportable events within the inpatient mental health care: Impact on physicians and nurses. Revista de Calidad Asistencial. 2016 Jul 1;31:26-33.		X
24	Mathebula LC, Filmalter CJ, Jordaan J, Heyns T. Second victim experiences of healthcare providers after adverse events: A cross-sectional study. Health SA Gesondheid (Online). 2022;27:1-6.	X	X
25	McLennan SR, Engel-Glatter S, Meyer AH, Schwappach DL, Scheidegger DH, Elger BS. The impact of medical errors on Swiss anaesthesiologists: a cross-sectional survey. Acta Anaesthesiologica Scandinavica. 2015 Sep;59(8):990-8.	X	
26	Mohamadi-Bolbanabad A, Moradi G, Piroozi B, Safari H, Asadi H, Nasseri K, Mohammadi H, Afkhamzadeh A. The second victims' experience and related factors among medical staff. International Journal of Workplace Health Management. 2019 May 15;12(3):134-45.	X	
27	Mok WQ, Chin GF, Yap SF, Wang W. A cross-sectional survey on nurses' second victim experience and quality of support resources in Singapore. Journal of Nursing Management. 2020 Mar;28(2):286-93.	X	
28	Nijs K, Seys D, Coppens S, Van De Velde M, Vanhaecht K. Second victim support structures in anaesthesia: a cross-sectional survey in Belgian anaesthesiologists. International Journal for Quality in Health Care. 2021 Apr 1;33(2):mzab058.		X
29	O'Meara S, D'Arcy F, Dowling C, Walsh K. The psychological impact of adverse events on urology trainees. Irish Journal of Medical Science (1971-). 2023 Aug;192(4):1819-24	X	
30	Rivera-Chiauzzi E, Finney RE, Riggan KA, Weaver AL, Long ME, Torbenson VE, Allyse MA. Understanding the second victim experience among multidisciplinary providers in obstetrics and gynecology. Journal of Patient Safety. 2022 Mar 1;18(2):e463-9.		X
31	Rodriquez J, Scott SD. When clinicians drop out and start over after adverse events. The Joint Commission Journal on Quality and Patient Safety. 2018 Mar 1;44(3):137-45.	X	X
32	Santana-Domínguez I, González-De La Torre H, Verdú-Soriano J, Berenguer-Pérez M, Suárez-Sánchez JJ, Martín-Martínez A. Feelings of being a second victim among Spanish midwives and obstetricians. Nursing Open. 2022 Sep;9(5):2356-69.	X	
33	Schrøder K, Edrees HH, Christensen RD, Jørgensen JS, Lamont RF, Hvidt NC. Second victims in the labor ward: Are Danish midwives and obstetricians getting the support they need?.		X

For peer review only - http://bmjopen.bmj.com/site/about/guidelines.xhtml

	International Journal for Quality in Health Care. 2019 Oct 31;31(8):583-9.		
34	Shuangjiang Z, Huanhuan H, Ling X, Qinghua Z, Mingzhao X. Second victim experience and support desire among nurses working at regional levels in China. Journal of Nursing Management. 2022 Apr;30(3):767-76.	X	
35	Strametz R, Fendel JC, Koch P, Roesner H, Zilezinski M, Bushuven S, Raspe M. Prevalence of Second Victims, Risk Factors, and Support Strategies among German Nurses (SeViD-II Survey). International Journal of Environmental Research and Public Health. 2021 Oct 10;18(20):10594.	X	X
36	Strametz R, Koch P, Vogelgesang A, Burbridge A, Rösner H, Abloescher M, Huf W, Ettl B, Raspe M. Prevalence of second victims, risk factors and support strategies among young German physicians in internal medicine (SeViD-I survey). Journal of Occupational Medicine and Toxicology. 2021 Dec;16(1):1-1.	X	X
37	Sun L, Deng J, Xu J, Ye X. Rumination's role in second victim nurses' recovery from psychological trauma: a cross-sectional study in China. Frontiers in Psychology. 2022 May 3;13:860902.	X	
38	Torbenson VE, Riggan KA, Weaver AL, Long ME, Finney RE, Allyse MA, Rivera-Chiauzzi E. Second victim experience among OBGYN trainees: what is their desired form of support? Southern Medical Journal. 2021 Apr;114(4):218.	X	X
39	Turner K, Bolderston H, Thomas K, Greville-Harris M, Withers C, McDougall S. Impact of adverse events on surgeons. British Journal of Surgery. 2022 Apr 2;109(4):308-10.		X
40	Van Gerven E, Bruyneel L, Panella M, Euwema M, Sermeus W, Vanhaecht K. Psychological impact and recovery after involvement in a patient safety incident: a repeated measures analysis. BMJ Open. 2016 Aug 1;6(8):e01140.		X
41	Vanhaecht K, Zeeman G, Schouten L, Bruyneel L, Coeckelberghs E, Panella M, Seys D, Dutch Peer Support Collaborative Research Group. Peer support by interprofessional health care providers in aftermath of patient safety incidents: A cross-sectional study. Journal of Nursing Management. 2021 Oct;29(7):2270-7.	X	X
42	Van Slambrouck L, Verschueren R, Seys D, Bruyneel L, Panella M, Vanhaecht K. Second victims among baccalaureate nursing students in the aftermath of a patient safety incident: An exploratory cross-sectional study. Journal of Professional Nursing. 2021 Jul 1;37(4):765-70.	X	X
43	Vinson AE, Mitchell JD. Assessing levels of support for residents following adverse outcomes: a national survey of		X

For peer review only - http://bmjopen.bmj.com/site/about/guidelines.xhtml

	anesthesia residency programs in the United States. Medical Teacher. 2014 Oct 1;36(10):858-66.		
44	Winning AM, Merandi J, Rausch JR, Liao N, Hoffman JM, Burlison JD, Gerhardt CA. Validation of the second victim experience and support tool-revised in the neonatal intensive care unit. Journal of Patient Safety. 2021 Dec 1;17(8):531-40.	X	
45	Wolf M, Smith K, Basu M, Heiss K. The Prevalence of Second Victim Syndrome and Emotional Distress in Pediatric Intensive Care Providers. Journal of Pediatric Intensive Care. 2021 Jul 1;12(02):125-30.	X	X
46	Yan L, Tan J, Chen H, Yao L, Li Y, Zhao Q, Xiao M. Experience and support of Chinese healthcare professionals as second victims of patient safety incidents: A cross-sectional study. Perspectives in Psychiatric Care. 2022 Apr;58(2):733-43.		X
47	Zhang X, Li Q, Guo Y, Lee SY. From organisational support to second victim-related distress: Role of patient safety culture. Journal of Nursing Management. 2019 Nov;27(8):1818-25.	X	X
48	Cutler A, Black JD, Sheth SS, Pathy S. When Things Go Wrong: Examining the Frequency and Aftermath of Critical Incidents Among OBGYN Residents. Connecticut Medicine. 2017 May 1;81(5).	X	X
49	Biggs S, Waggett HB, Shabbir J. Impact of surgical complications on the operating surgeon. Colorectal Disease. 2020 Sep;22(9):1169-74.		X
50	Buhlmann M, Ewens B, Rashidi A. Moving on after critical incidents in health care: A qualitative study of the perspectives and experiences of second victims. Journal of Advanced Nursing. Sep 2022;78(9):2960-2972.	X	X
51	Chang HE, Jang H, Bak YI. Clinical nurses' recovery experiences after adverse events in South Korea: A qualitative study. Collegian. 2022;29(4):456-464.	X	X
52	Christoffersen L, Teigen J, Ronningstad C, Following-up midwives after adverse incidents: How front-line management practices help second victims. Midwifery. Jun 2020;85:102669	X	X
53	De Boer J, van Rikxoort S, Bakker AB, Smit BJ. Critical incidents among intensive care unit nurses and their need for support: explorative interviews. Nursing in Critical Care. Jul 2014;19(4):166-74.	X	X
54	Ferrùs L, Silvestre C, Olivera G, Mira JJ. Qualitative study about the experiences of colleagues of health professionals involved in an adverse event. Journal of Patient Safety. 2021 Jan 1;17(1):36-43.	X	X
55	Ganahl S, Knaus M, Wiesenhuetter I, Klemm V, Jabinger EM, Strametz R. Second Victims in Intensive Care—Emotional		X

For peer review only - http://bmjopen.bmj.com/site/about/guidelines.xhtml

	Stress and Traumatization of Intensive Care Nurses in Western Austria after Adverse Events during the Treatment of Patients. International Journal of Environmental Research and Public Health. 2022 Mar 18;19(6):3611.		
56	Kable A, Kelly B, Adams J. Effects of adverse events in health care on acute care nurses in an Australian context: A qualitative study. Nursing and Health Sciences Jun 2018;20(2):238-246	X	X
57	Lee W, Pyo J, Jang SG, Choi JE, Ock M. Experiences and responses of second victims of patient safety incidents in Korea: a qualitative study. BMC Health Serv Res Feb 06 2019;19(1):100.	X	X
58	Leferink E, Bos A, Heringa MP, van Rensen EL, Zwart DL. The need and availability of support systems for physicians involved in a serious adverse event. Journal of Hospital Administration. 2018;7(2):23-30.	X	X
59	Luk LA, Lee FK, Lam CS, So HY, Wong YY, Lui WS. Healthcare professional experiences of clinical incident in Hong Kong: a qualitative study. Risk Management and Healthcare Policy. 2021 Mar 8:947-57.	X	X
60	May N, Plews-Ogan M. The role of talking (and keeping silent) in physician coping with medical error: A Qualitative study. Patient Education and Counseling Sep 2012;88(3):449-454.		X
61	Mokhtari Z, Hosseini MA, Khankeh HR, Fallahi-Khoshknab M, Nasrabadi AN. Barriers to support nurses as second victim of medical errors: A qualitative study. Australasian Medical Journal 2018;11(12):556-560.		X
62	Pinto A, Faiz O, Bicknell C, Vincent C. Surgical complications and their implications for surgeons' well-being. British Journal of Surgery. Dec 2013;100(13):1748-55	X	X
63	Rinaldi C, Leigheb F, Vanhaecht K, Donnarumma C, Panella M. Becoming a "second victim" in health care: Pathway of recovery after adverse event. Revista de Calidad Asistencial. 2016 Jul 1;31:11-9.	X	X
64	Serou N, Husband AK, Forrest SP, Slight RD, Slight SP. Support for Healthcare Professionals After Surgical Patient Safety Incidents: A Qualitative Descriptive Study in 5 Teaching Hospitals. J Patient Saf Aug 01 2021;17(5):335-340.	X	X
65	Schrøder K, Janssens A, Hvidt EA. Adverse events as transitional markers-Using liminality to understand experiences of second victims. Social Science and Medicine. 2021 Jan 1;268:113598.	X	X
66	Tavares APM, Barlem JGT, Silveira RSD, Dalmolin GL, Feijó GDS, Machado IA, Paloski GDR, Abreu IM. Support provided to nursing students in the face of patient safety incidents: a qualitative study. Rev Bras Enferm 2022;75(2):e20220009.	X	X

For peer review only

For peer review only - http://bmjopen.bmj.com/site/about/guidelines.xhtml

67	Ullström S, Sachs MA, Hansson J, Øvretveit J, Brommels M. Suffering in silence: a qualitative study of second victims of adverse events. BMJ Quality & Safety. 2014 Apr 1;23(4):325-31.	X	X
68	Van Gerven E, Deweer D, Scott SD, Panella M, Euwema M, Sermeus W, Vanhaecht K. Personal, situational and organizational aspects that influence the impact of patient safety incidents: a qualitative study. Revista de Calidad Asistencial. 2016 Jul 1;31:34-46.	X	X
69	Huang H, Chen J, Xiao M, Cao S, Zhao Q. Experiences and responses of nursing students as second victims of patient safety incidents in a clinical setting: A mixed-methods study. Journal of Nursing Management. 2020 Sep;28(6):1317-25.		X
70	Kobe C, Blouin S, Moltzan C, Koul R. The second victim phenomenon: perspective of Canadian radiation therapists. Journal of Medical Imaging and Radiation Sciences. 2019 Mar 1;50(1):87-97.	X	X
71	Kruper A, Domeyer-Klenske A, Treat R, Pilarski A, Kaljo K. Secondary traumatic stress in Ob-Gyn: A mixed methods analysis assessing physician impact and needs. Journal of Surgical Education. 2021 May 1;78(3):1024-34.	X	X
70	Kobe C, Blouin S, Moltzan C, Koul R. The second victim phenomenon: perspective of Canadian radiation therapists. Journal of Medical Imaging and Radiation Sciences. 2019 Mar 1;50(1):87-97.	X	X
71	Kruper A, Domeyer-Klenske A, Treat R, Pilarski A, Kaljo K. Secondary traumatic stress in Ob-Gyn: A mixed methods analysis assessing physician impact and needs. Journal of Surgical Education. 2021 May 1;78(3):1024-34.	X	X
72	Plews-Ogan M, May N, Owens J, Ardelt M, Shapiro J, Bell SK. Wisdom in Medicine: What Helps Physicians After a Medical Error? Academic Medicine. Feb 2016;91(2):233-41		X
	Total studies	52	57

For peer review only

Supplementary 4 File: Description of fourth and fifth (a) most wanted and (b) most frequently experienced forms of support (continued from main article)

(a) Continuation of most wanted forms of support

#4 most desired: Post-incident debrief to clarify and process what happened (n=18 studies):

Debriefs and team discussions with colleagues involved in the incident were wanted by some participants. However, it was not always clear how this was different to peer support, or whether it referred to an informal clinical debriefing or to more structured Critical Incident Stress Debriefing. Fourteen studies found that second victims wanted information about the incident, including an opportunity to explore any ethical issues, causes and preventative measures, and also to be involved in evaluation and learning around the incident which could contribute to post-traumatic growth.

#5 most desired: Supportive and learning-oriented culture (n=14 studies): Healthcare staff in many studies expressed their desire for a responsive organisational approach that supported them after a patient safety incident and a culture in which they felt protected, recognised and not blamed or criticised. In one study, participants ranked the promotion of an open reporting and learning culture as the most critically important form of support to aid recovery and avoid compounding psychological harm after involvement in a patient safety incident. Raising awareness of the impact of patient safety incidents/errors and preparing staff for involvement in such events, through courses and conferences within one's organisation, were also rated as highly desirable in one study.

(b) Continuation of most experienced forms of support

#4 most experienced: Professional mental health support (n=11): Counselling and other professional mental health support was also mentioned in multiple studies. However, this was experienced less often than other support options. In studies where organisations did not offer such services, participants reported seeking this help themselves (e.g. 40% of one sample; **63)**.

For peer review only - http://bmjopen.bmj.com/site/about/guidelines.xhtml

There were indications of stigma associated with this option, with almost a third of physicians (31%) in one mixed methods study (17) reporting feeling embarrassed about needing psychological support.

#5 most experienced: The Mortality and Morbidity meeting (n=7): The Mortality and Morbidity (M&M) meeting was referred to in several studies as a form of support after patient safety incidents. These were the joint second most commonly experienced form of support amongst studies that ranked strategies. Some challenges were noted with this meeting as a support approach, such as not all staff involved in an incident may be invited and the focus on logistics and learning may provide little space to discuss the emotional and professional impact.

For peer review only

For peer review only - http://bmjopen.bmj.com/site/about/guidelines.xhtml

PRISMA 2020 for Abstracts Checklist

Section and Topic	Item #	Checklist item	Reported (Yes/No)
**TITLE**			
Title	1	Identify the report as a systematic review.	Yes
**BACKGROUND**			
Objectives	2	Provide an explicit statement of the main objective(s) or question(s) the review addresses.	Yes
**METHODS**			
Eligibility criteria	3	Specify the inclusion and exclusion criteria for the review.	Yes
Information sources	4	Specify the information sources (e.g. databases, registers) used to identify studies and the date when each was last searched.	Yes
Risk of bias	5	Specify the methods used to assess risk of bias in the included studies.	Yes
Synthesis of results	6	Specify the methods used to present and synthesise results.	Yes
**RESULTS**			
Included studies	7	Give the total number of included studies and participants and summarise relevant characteristics of studies.	Yes
Synthesis of results	8	Present results for main outcomes, preferably indicating the number of included studies and participants for each. If meta-analysis was done, report the summary estimate and confidence/credible interval. If comparing groups, indicate the direction of the effect (i.e. which group is favoured).	Yes
**DISCUSSION**			
Limitations of evidence	9	Provide a brief summary of the limitations of the evidence included in the review (e.g. study risk of bias, inconsistency and imprecision).	Yes
Interpretation	10	Provide a general interpretation of the results and important implications.	Yes
**OTHER**			
Funding	11	Specify the primary source of funding for the review.	Yes
Registration	12	Provide the register name and registration number.	Yes

From: Page MJ, McKenzie JE, Bossuyt PM, Boutron I, Hoffmann TC, Mulrow CD, et al. The PRISMA 2020 statement: an updated guideline for reporting systematic reviews. BMJ 2021;372:n71. doi: 10.1136/bmj.n71

For more information, visit: http://www.prisma-statement.org/

For peer review only

Supplementary 1 File: Completed PRISMA 2020 Checklist

Section and Topic	Item #	Checklist item	Location where item is reported
**TITLE**			
Title	1	Identify the report as a systematic review.	1
**ABSTRACT**			
Abstract	2	See the PRISMA 2020 for Abstracts checklist.	Used this
**INTRODUCTION**			
Rationale	3	Describe the rationale for the review in the context of existing knowledge.	5-6
Objectives	4	Provide an explicit statement of the objective(s) or question(s) the review addresses.	6-7
**METHODS**			
Eligibility criteria	5	Specify the inclusion and exclusion criteria for the review and how studies were grouped for the syntheses.	7, 9-10
Information sources	6	Specify all databases, registers, websites, organisations, reference lists and other sources searched or consulted to identify studies. Specify the date when each source was last searched or consulted.	7-8
Search strategy	7	Present the full search strategies for all databases, registers and websites, including any filters and limits used.	7 (S2_File)
Selection process	8	Specify the methods used to decide whether a study met the inclusion criteria of the review, including how many reviewers screened each record and each report retrieved, whether they worked independently, and if applicable, details of automation tools used in the process.	8-9
Data collection process	9	Specify the methods used to collect data from reports, including how many reviewers collected data from each report, whether they worked independently, any processes for obtaining or confirming data from study investigators, and if applicable, details of automation tools used in the process.	8-9
Data items	10a	List and define all outcomes for which data were sought. Specify whether all results that were compatible with each outcome domain in each study were sought (e.g. for all measures, time points, analyses), and if not, the methods used to decide which results to collect.	8
	10b	List and define all other variables for which data were sought (e.g. participant and intervention characteristics, funding sources). Describe any assumptions made about any missing or unclear information.	8-9
Study risk of bias assessment	11	Specify the methods used to assess risk of bias in the included studies, including details of the tool(s) used, how many reviewers assessed each study and whether they worked independently, and if applicable, details of automation tools used in the process.	9
Effect measures	12	Specify for each outcome the effect measure(s) (e.g. risk ratio, mean difference) used in the synthesis or presentation of results.	N/A
Synthesis methods	13a	Describe the processes used to decide which studies were eligible for each synthesis (e.g. tabulating the study intervention characteristics and comparing against the planned groups for each synthesis (item #5)).	9-10
	13b	Describe any methods required to prepare the data for presentation or synthesis, such as handling of missing summary statistics, or data conversions.	N/A
	13c	Describe any methods used to tabulate or visually display results of individual studies and syntheses.	10
	13d	Describe any methods used to synthesize results and provide a rationale for the choice(s). If meta-analysis was performed, describe the model(s), method(s) to identify the presence and extent of statistical heterogeneity, and software package(s) used.	9-10
	13e	Describe any methods used to explore possible causes of heterogeneity among study results (e.g. subgroup analysis, meta-regression).	N/A
	13f	Describe any sensitivity analyses conducted to assess robustness of the synthesized results.	N/A
Reporting bias assessment	14	Describe any methods used to assess risk of bias due to missing results in a synthesis (arising from reporting biases).	N/A
Certainty assessment	15	Describe any methods used to assess certainty (or confidence) in the body of evidence for an outcome.	N/A

For peer review only

PRISMA 2020 Checklist

Section and Topic	Item #	Checklist item	Location where item is reported
**RESULTS**			
Study selection	16a	Describe the results of the search and selection process, from the number of records identified in the search to the number of studies included in the review, ideally using a flow diagram.	10
	16b	Cite studies that might appear to meet the inclusion criteria, but which were excluded, and explain why they were excluded.	10-11
Study characteristics	17	Cite each included study and present its characteristics.	10-11; 17; S3 File
Risk of bias in studies	18	Present assessments of risk of bias for each included study.	19-20
Results of individual studies	19	For all outcomes, present, for each study: (a) summary statistics for each group (where appropriate) and (b) an effect estimate and its precision (e.g. confidence/credible interval), ideally using structured tables or plots.	12-13, 17, S3 File, S1 Table 4
Results of syntheses	20a	For each synthesis, briefly summarise the characteristics and risk of bias among contributing studies.	19
	20b	Present results of all statistical syntheses conducted. If meta-analysis was done, present for each the summary estimate and its precision (e.g. confidence/credible interval) and measures of statistical heterogeneity. If comparing groups, describe the direction of the effect.	N/A
	20c	Present results of all investigations of possible causes of heterogeneity among study results.	19
	20d	Present results of all sensitivity analyses conducted to assess the robustness of the synthesized results.	N/A
Reporting biases	21	Present assessments of risk of bias due to missing results (arising from reporting biases) for each synthesis assessed.	N/A
Certainty of evidence	22	Present assessments of certainty (or confidence) in the body of evidence for each outcome assessed.	N/A
**DISCUSSION**			
Discussion	23a	Provide a general interpretation of the results in the context of other evidence.	23-25
	23b	Discuss any limitations of the evidence included in the review.	23
	23c	Discuss any limitations of the review processes used.	26
	23d	Discuss implications of the results for practice, policy, and future research.	26
**OTHER INFORMATION**			
Registration and protocol	24a	Provide registration information for the review, including register name and registration number, or state that the review was not registered.	3, 7
	24b	Indicate where the review protocol can be accessed, or state that a protocol was not prepared.	3, 7
	24c	Describe and explain any amendments to information provided at registration or in the protocol.	8
Support	25	Describe sources of financial or non-financial support for the review, and the role of the funders or sponsors in the review.	27
Competing interests	26	Declare any competing interests of review authors.	27
Availability of data, code and other materials	27	Report which of the following are publicly available and where they can be found: template data collection forms; data extracted from included studies; data used for all analyses; analytic code; any other materials used in the review.	Detailed narrative data synthesis - S3 File

From: Page MJ, McKenzie JE, Bossuyt PM, Boutron I, Hoffmann TC, Mulrow CD, et al. The PRISMA 2020 statement: an updated guideline for reporting systematic reviews. BMJ 2021;372:n71. doi: 10.1136/bmj.n71

For more information, visit: http://www.prisma-statement.org/

For peer review only - http://bmjopen.bmj.com/site/about/guidelines.xhtml

BMJ Open

Avoiding 'second victims' in healthcare: What support do staff want for coping with patient safety incidents, what do they get, and is it effective? A systematic review.

Journal:	*BMJ Open*
Manuscript ID	bmjopen-2024-087512.R1
Article Type:	Original research
Date Submitted by the Author:	19-Dec-2024
Complete List of Authors:	Simms-Ellis, Ruth; Bradford Institute for Health Research Yorkshire Quality and Safety Research Group; University of Leeds<br>Harrison, Reema; Macquarie University, Australian Institute of Health Innovation<br>Sattar, Raabia; Bradford Institute for Health Research Yorkshire Quality and Safety Research Group<br>Sweeting, Elizabeth; Bradford Institute for Health Research, Improvement Academy<br>Hartley, Hannah; Bradford Institute for Health Research Yorkshire Quality and Safety Research Group<br>Morys-Edge, Matthew; University of Leeds<br>Lawton, Rebecca; University of Leeds, School of Psychology; Bradford Institute for Health Research Yorkshire Quality and Safety Research Group
**Primary Subject Heading**:	Occupational and environmental medicine
Secondary Subject Heading:	Mental health
Keywords:	Occupational Stress, Health Workforce, MENTAL HEALTH, OCCUPATIONAL & INDUSTRIAL MEDICINE, Organisational development < HEALTH SERVICES ADMINISTRATION & MANAGEMENT

SCHOLARONE™
Manuscripts

I, the Submitting Author has the right to grant and does grant on behalf of all authors of the Work (as defined in the below author licence), an exclusive licence and/or a non-exclusive licence for contributions from authors who are: i) UK Crown employees; ii) where BMJ has agreed a CC-BY licence shall apply, and/or iii) in accordance with the terms applicable for US Federal Government officers or employees acting as part of their official duties; on a worldwide, perpetual, irrevocable, royalty-free basis to BMJ Publishing Group Ltd ("BMJ") its licensees and where the relevant Journal is co-owned by BMJ to the co-owners of the Journal, to publish the Work in this journal and any other BMJ products and to exploit all rights, as set out in our licence.

The Submitting Author accepts and understands that any supply made under these terms is made by BMJ to the Submitting Author unless you are acting as an employee on behalf of your employer or a postgraduate student of an affiliated institution which is paying any applicable article publishing charge ("APC") for Open Access articles. Where the Submitting Author wishes to make the Work available on an Open Access basis (and intends to pay the relevant APC), the terms of reuse of such Open Access shall be governed by a Creative Commons licence – details of these licences and which Creative Commons licence will apply to this Work are set out in our licence referred to above.

Other than as permitted in any relevant BMJ Author's Self Archiving Policies, I confirm this Work has not been accepted for publication elsewhere, is not being considered for publication elsewhere and does not duplicate material already published. I confirm all authors consent to publication of this Work and authorise the granting of this licence.

For peer review only

Avoiding 'second victims' in healthcare: What support do staff want for coping with patient safety incidents, what do they get, and is it effective? A systematic review.

Ruth Simms-Ellis [1,2*], Reema Harrison[3], Raabia Sattar[1], Elizabeth Sweeting[4], Hannah Hartley[1], Matthew Morys-Edge[2], Rebecca Lawton[1,2].

Affiliations

[1] Yorkshire Quality and Safety Research Group, Bradford Institute for Health Research, Bradford Royal Infirmary, Bradford, United Kingdom.

[2] School of Psychology, University of Leeds, Leeds, United Kingdom.

[3] Australian Institute of Health Innovation, Macquarie University, Sydney, Australia.

[4] Improvement Academy, Bradford Institute for Health Research, Bradford Royal Infirmary, Bradford, United Kingdom.

* Corresponding author.

Email: r.simms-ellis@leeds.ac.uk (RSE)

For peer review only

For peer review only - http://bmjopen.bmj.com/site/about/guidelines.xhtml

ABSTRACT

Objectives: Incontrovertible evidence surrounds the need to support healthcare professionals after patient safety incidents (PSI). However, what characterises effective organisational support is less clearly understood and defined. This review aims to determine what support healthcare professionals want for coping with PSIs, what support interventions/approaches are currently available and which have evidence for effectiveness.

Design: Systematic research review with narrative synthesis. PROSPERO registration number: CRD42022325796 [https://www.crd.york.ac.uk/PROSPERO/display_record.php?RecordID=325796]

Data Sources: Medline, Scopus, PubMed and Web of Science were searched (from 2010-mid 2021; updated December 2022), reference lists of eligible articles and Connected Papers software.

Eligibility Criteria for Selecting Studies: Empirical studies (1) containing information about support frontline healthcare staff want before/after a PSI, OR addressing (2) support currently available, OR (3) the effectiveness of support to help prevent/alleviate consequences of a PSI. Study quality was appraised using the Quality Assessment for Diverse Studies tool.

Results: Ninety-nine studies were identified. Staff most wanted: peer support (n=28), practical support and guidance (n=27) and professional mental health support (n=21). They mostly received: peer support (n=46), managerial support (n=23) and some form of debrief (n=15). Reports of poor PSI support were common. Eleven studies examined intervention effectiveness. Evidence was positive for the effectiveness of preventive/preparatory interventions (n=3), but mixed for peer support programmes designed to alleviate harmful consequences after PSIs (n=8). Study quality varied.

Conclusions: Beyond peer support, organisational support for PSIs appears to be mis-aligned

with staff desires. Gaps exist in providing preparatory/preventive interventions and practical support and guidance. Reliable effectiveness data are lacking. Very few studies incorporated comparison groups or randomisation; most used self-report measures. Despite inconclusive evidence, formal peer support programmes dominate. This review illustrates a critical need to fund robust PSI-related intervention effectiveness studies to provide organisations with the evidence they need to make informed decisions when building PSI support programmes.

Key Words: health personnel, medical errors, occupational stress, occupational health services, organisational culture.

ARTICLE SUMMARY

Strengths and limitations of this study

- We applied a respected multi-level occupational health stress prevention model to patient safety incidents and used this model to map second victim support requirements and organisational support provision.
- We specifically targeted intervention effectiveness.
- Data extraction, quality assessment and narrative synthesis were undertaken independently by two reviewers to address potential issues of subjective judgment and lack of transparency. Our reporting was also guided by the Synthesis without Meta-analysis (SWiM) framework.
- Our search strategy may have omitted eligible studies, given the complexity of the research area, our focus on papers published in English between 2010 and 2022, and our choice and number of bibliographic search engines.
- Our final sample comprised studies that were heterogeneous in terms of quality, design, sample sizes, data description and analysis and outcomes measured.

FUNDING

For peer review only - http://bmjopen.bmj.com/site/about/guidelines.xhtml

The research was supported by the National Institute for Health and Care Research (NIHR) Yorkshire and Humber Patient Safety Translational Research Centre (PSTRC-2016-006) and NIHR Yorkshire and Humber Patient Safety Research Collaboration. The views expressed are those of the authors, and not necessarily those of the NIHR or the Department of Health and Social Care.

CONFLICTS OF INTEREST

All authors have completed the ICMJE uniform disclosure form at *http://www.icmje.org/disclosure-of-interest/* and declare: all authors (except RH) were supported by the National Institute for Health and Care Research (NIHR) Yorkshire and Humber Patient Safety Translational Research Centre (PSTRC-2016-006) and NIHR Yorkshire and Humber Patient Safety Research Collaboration*;* no financial relationships with any organisations that might have an interest in the submitted work in the previous three years; no other relationships or activities that could appear to have influenced the submitted work.

INTRODUCTION

The international healthcare workforce is in crisis [1] with an estimated global deficit of 9.9 million nurses, midwives and physicians by 2030 [2]. High turnover is a major contributor [3, 4] and this has accelerated during and since the COVID-19 pandemic [5]. Being involved in a 'patient safety incident' is a significant factor in healthcare staff turnover [6-8]: around 12% of healthcare professionals surveyed in one study had left their job after a patient safety incident [9].

Patient safety incidents (PSIs) are unintended or unexpected incidents, which harmed or could have harmed one or more patients receiving healthcare [10]. PSIs occur in around 10% [11] to 14% [12] of patient admissions in hospital and in 2%-3% of patient visits in

For peer review only

primary care [13, 14]. Being involved in harming or almost harming a patient can be traumatising for healthcare professionals, who typically enter healthcare to help others and uphold the Hippocratic oath of ‘first do not harm’ [15]. This human distress is frequently compounded and intensified by harsh retributive blame culture [16-21], a judgemental or critical response from colleagues and supervisors, inadequate support in disclosing what happened to the patient/family, the subsequent investigation process [22] and fears about litigation/malpractice [15].

The first priority after any PSI is to care for the patient and family, as the primary victim. However, there is incontrovertible evidence that healthcare professionals involved in such incidents also require their organisation’s support as the silent ‘second victims’ [23]. With a high number of healthcare professionals across different roles and countries reporting having experienced the second victim phenomenon at some point in their career (recent surveys range from 45% of 205 US obstetrics and gynaecology workers [24] to 91% of 658 Asian surgeons [25]), the scale and universality of this issue is significant.

The potential, harmful impact of PSIs on individual healthcare professionals and healthcare organisations has been well documented through over 20 years of international research into the second victim phenomenon [6, 7, 15, 16, 26-35]. Individuals consistently describe experiencing a range of emotional, cognitive, behavioural and physiological symptoms (see Table 1). Many of these symptoms are commensurate with a clinical diagnosis for Post-Traumatic Stress Disorder (PTSD) [36] and burnout, which is significantly associated with involvement in a PSI [34, 37]. Impacts on professional functioning include an impaired ability to manage patients in the aftermath of a PSI, reduced confidence, self-doubts, fears about litigation, losing one’s job [21, 34], defensive changes in practice, such as avoiding procedures, secrecy/cover ups and ordering up to 30% more tests than necessary [38]. The organisational impact of PSIs include compromised team functioning, patient safety

For peer review only

For peer review only - http://bmjopen.bmj.com/site/about/guidelines.xhtml

and care quality [34], increased costs from defensive practice and reluctance to report future incidents [38], absenteeism, turnover and the loss of highly trained staff [6].

Table 1: Four symptom groups associated with the 'second victim' experience

Symptom Group	Individual Symptoms
Emotional	Shame; Guilt; Anxiety; Anger; Shock; Depression; Fear.
Cognitive	Flashbacks; Intrusive thoughts and nightmares; Re-imagining; Ruminating on details; Helplessness; Suicidal ideation.
Physiological	Fatigue; Sleep disturbance; Nausea; Headaches; Gastro-intestinal issues.
Behavioural	Hypervigilance; Withdrawal; Irritability; Social avoidance; Emotional numbing; Poor concentration; Diminished memory; Avoidance of triggers.

Whilst there is incontrovertible evidence for the need to support healthcare professionals with PSIs, less clarity surrounds what type of support they actually want and tend to receive, or what characterises an 'effective' support intervention [39]. A recommended model for supporting healthcare professionals *after* involvement in PSIs was developed inductively by Scott and colleagues' [40], featuring three tiers: local support from leaders and colleagues; wellbeing support from trained peers and patient safety colleagues; a referral network for staff requiring more help. Seys et al.'s [41] subsequent early review of the literature surrounding second victim support corroborated this multi-level approach. Their recent, updated review [42] also recommended incorporating a further 'prevention' tier to Scott's model help minimise the impact of PSIs. This multi-level approach, firmly rooted in prevention reflects best practice in stress management more broadly [43-45], which advocates: minimising the stressors that compromise employee wellbeing (primary

For peer review only

For peer review only - http://bmjopen.bmj.com/site/about/guidelines.xhtml

prevention level), moderating individuals' reactions when exposed to stressors (secondary level) and providing individualised support to those experiencing strain (tertiary level).

However, numerous reviews to date examining what support is actually available for second victims (including scoping [46, 47], literature [41, 48], narrative [42], integrative [39, 49] and systematic [50]) have identified that such support is rare and limited. Even when support is available, various challenges affect whether and/or how it is used and received. These include a 'blame' culture [49], staff resistance to usage, reluctance to ask for help, low awareness of availability [39, 50] and a heavy reliance on volunteers due to lack of funding and resources, which can compromise availability and sustainability [39, 49, 50].

Collectively these reviews provide extensive coverage of the range of support interventions available internationally (more than 40 were documented in one review [46]). However, reviews to date have largely aimed to identify and describe second victim support interventions available, rather than to critically evaluate outcomes. Included papers have varied in nature, including descriptions of programme development and peer training workshops, explorations of risk managers' perceptions of second victim programmes and reports of implementation and/or usage data. Whilst mostly reporting acceptability, perceived helpfulness, feasibility or implementation challenges, some of these reviews [e.g., 48, 49, 50] have also considered in their aims intervention benefits or what has proved helpful/not helpful. However, only one [47] cites the investigation of intervention outcomes for healthcare professionals and organisations as an aim. Searching the literature up to 2018, Wade and colleagues' scoping review [47] concluded that 'there is little scientific rigor from which to determine whether these programs are indeed meeting their goals and objectives' (p. e70). As others have also noted [39, 46, 48, 50], we currently have limited understanding of what actually 'works' when it comes to supporting staff through PSIs.

For peer review only

There is broad agreement that supporting staff with PSIs is the responsibility of senior organisational leaders, that support should be comprehensive, part of a wider just, restorative patient safety culture, sustainable and well-funded [39, 41, 42, 46-50]. However, for organisations to undertake such an investment and provide quality support, they require more robust evidence. There is a need to understand, comprehensively and systematically, what type of support staff actually want and what interventions can be provided to meet their desires (a key factor in the uptake of wellbeing interventions [51]) that are effective.

In this systematic review, we used a widely-respected, multi-level stress prevention model [43] to map and synthesise the empirical evidence surrounding second victim support. Specifically, we aimed to answer the following research questions:

1. What support do healthcare staff want before and after a PSI?
2. What support is currently available to these staff?
3. How effective are existing support mechanisms/interventions designed to: (a) prevent poor psychological outcomes for healthcare staff after PSIs (primary-level); (b) support staff in the aftermath of PSIs (secondary-level); offer psychological support for those affected by involvement in PSIs (tertiary-level).

METHODS

Design

We used systematic review with narrative synthesis [52] and followed the Preferred Reporting Items for Systematic Reviews and Meta analyses [53] (for PRISMA checklist, see S1 File). The protocol was registered with the International Prospective Register of Systematic Reviews (PROSPERO; CRD42022325796).

Patient and Public Involvement

For peer review only

No patients or members of the public were involved in the design, conduct or reporting of this review. However, we plan to disseminate our findings through our patient partner networks, which will be made available to people in a range of ways. Key messages will also be shared through social media**.**

Eligibility Criteria

Studies were eligible if they were empirical and contained information within any healthcare context about the support frontline healthcare staff want before or after a patient safety incident, OR the support currently available, OR the effectiveness of interventions to help prevent or mitigate second victim phenomena. Searches were limited to studies published in English between January 2010 and December 2022. Our focus was much less on understanding the prevalence of workforce responses to error and investigations (the types of publications that emerged shortly after the term 'second victim' was coined by Albert Wu in 2000 [23]) and much more on the types of interventions and their effectiveness. A number of key papers on staff support for PSIs were published in 2010, including the Institute for Healthcare Improvement's white paper on respectful management of PSIs [54], Scott et al.'s [40] seminal paper on organisational support for second victims and our original systematic review on coping with medical error [55]. Therefore, to make the review focused and manageable, we chose to focus on publications since this time. Articles were excluded if they were grey literature, study protocols, opinion pieces, systematic reviews, meta-analyses, theses, focused on general staff well being, stressful non-patient safety incidents or reducing, or increasing the reporting of, medical errors.

Study identification

Drawing upon search terms used in our recent, related systematic review [31] and with support from a medical information specialist, synonyms and relevant concepts were

For peer review only - http://bmjopen.bmj.com/site/about/guidelines.xhtml

developed for the two major concepts being explored: second victim and emotional support. A search strategy was developed iteratively and applied to the following electronic databases in June 2021 and updated in December 2022: Medline, Scopus, PubMed and Web of Science (For all search strategies, see S2 File). Reference lists of eligible articles were also searched and Connected Papers software tool (https://www.connectedpapers.com/) was used to identify additional studies. Covidence systematic review software (Veritas Health Innovation, Melbourne, Australia) was used for study screening and management.

Study selection and data extraction

After a preliminary scan of the eligible articles, we determined that a two-stage process was required for the review: Stage 1 would address research questions 1 and 2; Stage 2 to address research question 3. This represented a deviation from our published protocol. Using the eligibility criteria, four of the authors (RS, RL, RS-E, LS) with support from Dr Jane Heyhoe (JH), screened first titles and abstracts, then full texts for each potentially relevant study. Regular weekly meetings were held during this period to discuss screening decisions and disagreements, finalise eligible articles for inclusion and undertake a face validity check of the final set of studies. A PRISMA diagram was produced to evidence decisions made (Fig. 1).

Studies were selected if they could answer research question 1, 2, 3 or a combination and coded within Covidence to denote inclusion at Stage 1 or Stage 2. All authors extracted the following information from the included studies: author, publication year, country, study design, setting, sample, and key findings (qualitative and/or quantitative). In addition, all articles featuring individual interventions were categorised according to the level of organisational support offered, i.e., primary, secondary or tertiary.

At Stage 1, due to the large number of studies, we limited the extraction of ‘key findings’ to what healthcare staff said they wanted and what support they received. At Stage

For peer review only

2, the extraction of 'key findings' was more comprehensive to allow us to examine effectiveness of specific interventions. Our Stage 2 reporting is guided by the synthesis without meta-analysis (SWiM) in systematic reviews [56] framework (for SWiM checklist, see S3 file). We focused on outcomes, such as changes in confidence, job satisfaction or burnout. We did not focus on feasibility or acceptability, therefore we did not extract any data regarding user acceptability and intervention usage. Additional 'intervention description' data were also extracted at Stage 2, using the Template for Intervention Description and Replication (TIDieR) criteria [57]. At both stages, data extraction was conducted by all reviewers, with regular discussions to resolve disagreements. A random 20% sample of intervention effectiveness studies included in Stage 2 was double checked independently by a second reviewer (JH).

Assessment of study quality

Only studies included at Stage 2 were assessed for methodological quality. This was conducted independently by two reviewers (RS, HH) using the 13-item quality assessment with diverse studies tool (QuADS) [58], selected for its appropriateness for study samples with different methodological approaches.

Data synthesis

In this systematic review, we used an established multi-level stress prevention framework to help us organise and map the empirical evidence available. We adapted Cooper and Cartwright's [43] intervention strategy for general workplace stress to address the specific and unique workplace stressor/hazard of being involved in a patient safety incident as a healthcare professional in a healthcare organisation (Fig. 2; https://secondvictim.co.uk/supporting-second-victims/#why-support-second-victims).

Insert Figure 2 here

For peer review only - http://bmjopen.bmj.com/site/about/guidelines.xhtml

***Figure 2:** Organisational staff support model for Patient Safety Incidents*

Data at both stages were synthesised according to each research question using narrative synthesis. In addition, each form of support was coded according to the level it targeted: primary (i.e., preventive, proactive), secondary (i.e., aiding natural coping after an incident) or tertiary (i.e., specialist one-to-one remedial support).

At Stage 1, where a study reported support in ranked order of preference or most frequently received, we focused on the top three supports in each case. Where rankings were not available, such as in qualitative studies, we focused on the support most frequently reported as preferred and received. At Stage 2, an initial description of the intervention effectiveness studies' results was produced, including descriptive statistics and an assessment of study quality. A narrative synthesis was conducted, where studies were organised to explore the effectiveness of available support in helping to prevent or alleviate the anticipated or actual psychological consequences of a patient safety incident. Specifically, each intervention was coded as primary-, secondary- or tertiary-level support to create three groups of studies. Each group was reviewed and synthesised separately, before completing a multi-level, holistic examination and synthesis. Regular meetings were held to discuss and refine emerging findings. Study information was systematically tabulated by: intervention name, description, study design and quality appraisal score, sample and setting, outcome measure related to effectiveness (e.g., burnout, job satisfaction, confidence in coping), measure used and outcomes. Wherever available, we extracted P values as our standardised metric in examining intervention effects. Patterns within and between studies were explored to identify commonalities/differences and potential reasons for these.

RESULTS

For peer review only - http://bmjopen.bmj.com/site/about/guidelines.xhtml

Results of the search

We retrieved 4160 articles, 99 of which were included for data extraction (Fig. 1). All 99 studies were included at Stage 1. Only 11 [59-69] presented outcome data and purported to assess effectiveness, therefore were subsequently also included in Stage 2.

Insert Figure 1 here

Figure 1: *PRISMA flowchart of stages in the review*

Excluded studies

Studies were predominantly excluded at full text review because they were not empirical (n=39), they did not focus on the impact of PSIs on staff (n=39), or they did not aim to assess staff support interventions for PSIs, healthcare staff perceptions of PSI support, or the effect of PSI support on staff/organisational culture outcomes (n=38). Several exclusions were also made for studies that did not include frontline healthcare staff as participants (n=17), or examined 'organisational support' more broadly as a mediator for organisational outcomes (n=9).

Characteristics of included studies

Included studies were primarily from the United States (n=41), Europe (n=33), East and South-East Asia (n=13) and Australasia (n=6). Studies were quantitative (n=52), qualitative (n=22) and mixed methods (n=25). The cross-sectional survey dominated (n=73); those with a qualitative component mostly used semi-structured interviews (n=23), focus/online discussion, action groups or both (n=10). Samples were most often multi-disciplinary (n=53) with others featuring uni-disciplinary groups (n=46), which were mostly of physicians (n=22), nurses (n=16). Specialties represented included obstetrics and gynaecology or midwifery (n=13), surgery (n=10), anaesthesiology (n=7), paediatrics (n=5) and intensive/critical care (n=5), psychiatry and mental health (n=3), pharmacy (n=1),

For peer review only

For peer review only - http://bmjopen.bmj.com/site/about/guidelines.xhtml

radiotherapy (n=1), acute care (n=1), cardiology (n=1), urology (n=1) and internal medicine (n=1). Sample sizes ranged from 8 participants to 3,766 (based on n=92 studies: n=7 sample sizes were not clear).

Stage 1 Review Findings

What support do staff want before and after a PSI? (Research Question 1); What support is currently available to healthcare staff? (Research Question 2).

Seventy-two studies investigated what staff wanted (n=15), received (n=20) or both (n=37). Thirty-five different forms of 'desired' and 11 different types of 'experienced' support were identified. Due to the scale of the data, a decision was made to focus on the top 5 most highly rated and experienced forms of support (see Table 2), however a detailed narrative summary of findings and references can be found in S4 File. The top 3 for most desired and most experienced support are described below (see S5 File for top 4 and 5).

The top 3 most desired forms of support (Research Question 1)

#1 Peer support (n=28 studies): Peer support was the most desired form of support in every study that presented rankings. Specifically valued were peers sharing their own mistakes, checking in regularly and providing practical advice and reassurance about their professional capability to help restore confidence. However, the definition of 'peer support' differed between studies, from 'a respected peer to discuss the details of what happened', 'emotional support from colleagues', 'colleagues taking time to listen or help with workload' to 'conversations with peers'. Differences were also apparent in who second victims wanted peer support from. Some preferred 'informal peer support', from non-judgemental, empathic colleagues from their direct work environment, who fully understood the nature of the job and the incident itself. Others, wanted to speak with the whole team and some physicians wanted to talk only with other physicians.

For peer review only

#2 Practical support and guidance (n=27 studies): Preferences for practical support after a patient safety incident featured heavily across the sample, including rest and recuperation opportunities, temporary work adjustments and technical and procedural guidance. Having a specified peaceful location for recovery and regaining composure appeared as a top 3 most desired form of support in the second highest number of studies. A desire for temporary job adaptations and accommodation was also evident, such as being freed from workloads, having a review of one's responsibilities and being allowed to complete tasks and responsibilities with a colleague. Time away from work was also commonly desired, or a period of leave to help distance oneself and recover from the event. All these were desires for staff to initiate themselves and discuss with managers, rather than being instructed by managers punitively due to loss of trust after a PSI. Numerous studies found that second victims wanted technical guidance and advice with procedures surrounding investigations, legal aspects and support in communicating with the affected patient or family.

#3 Professional mental health support (n=21 studies): Specialist mental health support or counselling, including access to Employee Assistance Programmes (EAPs), was highly desired: of the studies that ranked support strategies, counselling was rated in the top 3 by the joint third most studies. Staff across several studies wanted confidential counselling to be provided in-house by the organisation, whilst a similar number wanted professional help offsite, through EAPs. In some studies, healthcare staff wanted formal support by a mental health professional, whilst in others, staff wanted an opportunity to *access* this if needed. Ambivalence was also sometimes evident, with concerns about whether this would be acceptable and utilised, particularly by surgical teams.

For peer review only - http://bmjopen.bmj.com/site/about/guidelines.xhtml

The top 3 most experienced forms of support (RQ2)

#1 Peer support (n=46 studies): Peer support was the most frequently experienced form of support, ranking most common in studies that ranked support frequency. Healthcare staff gained support speaking to direct colleagues, especially those at a similar grade and those involved in the incident. However, some ambiguity surrounds the way peer support was discussed across the sample. Often insufficient detail is detail is available to consistently identify and define peer support: some studies imply it is facilitated by the organisation in some structured, formal way; others imply it is more informal, talking to colleagues in one's team.

#2 Managerial support (n=23 studies): After peer support, support from supervisors, managers and department leaders was the next most frequently experienced form of support, according to included surveys. Qualitative and mixed methods studies also reported this extensively, detailing how managerial support helped to resolve emotional stress. Examples included giving staff space without chasing up incident reports, providing support via team meetings or one-to-ones and generally being proactive and accessible after an event. Managers' guidance and reassurance that they have confidence in individuals involved in an incident was highlighted as particularly important. Conversely, inadequate managerial support compounded isolation and hindered recovery.

#3 Post-incident debrief (n=15): Debriefs were also commonly experienced by staff, however descriptions of what these entailed were not always provided. Where details were available, the format and implementation were inconsistent across studies. For example, qualitative studies highlighted that these might be voluntary, certain staff might not be involved or invited, or the focus was on learning and attributing blame rather than emotional recovery.

For peer review only

For peer review only - http://bmjopen.bmj.com/site/about/guidelines.xhtml

Discrepancies between support desired versus experienced

An overarching finding is that healthcare staff commonly experienced their organisation's support as insufficient, contingent on one's role/seniority, non-existent or unclear. Themes of resentment and injustice ran through many of the included studies. There appear to be discrepancies between what healthcare staff want and what they actually receive (see Table 2). 'Peer support' needs appear to be appropriately met overall, but overlooked or neglected is the very highly rated need for 'practical support and guidance'. Similarly unmet is the desire for a supportive, learning-oriented culture; no experiences were reported of primary level interventions. By contrast, the procedural, routine 'Mortality and Morbidity meeting' was not a top 5 most desired support, yet, despite not technically being a wellbeing intervention, was the fifth most commonly experienced form of support.

Table 2: Most desired compared with most experienced forms of support for coping with patient safety incidents

**Most desired**	**No of papers in sample** *(In Top 3 of surveys; In qual. or mixed methods papers)*	**Most experienced**	**No of papers in sample** *(In Top 3 of surveys; In qual. or mixed methods papers)*
**1.** Peer support	**N=28** *(n=16; n=12)*	**1.** Peer support	**N=46** *(n=8; n=38)*
**2.** Practical support and guidance	**N=27** *(n=7; n=20)*	**2.** Managerial support	**N=23** *(n=5; n=18)*
**3.** Professional mental health support	**N=21** *(n=8; n=13)*	**3.** Post-incident debrief to clarify, process	**N=15** *(n=3; n=12)*
**4.** Post-incident debrief to clarify, process	**N=18** (n=8; n=10)	**4.** Professional mental health support	**N=11** *(n=0; n=11)*
**5.** Supportive, learning culture	**N=14** *(n=1; n=13)*	**5.** Mortality and Morbidity meeting	**N=7** *(n=3; n=4)*

Key: Primary Level Support | Secondary Level Support | Tertiary Level Support

For peer review only - http://bmjopen.bmj.com/site/about/guidelines.xhtml

Specific support interventions reported in empirical studies (Research Question 2)

In addition to the above 72 qualitative studies and surveys, a further 27 studies featured an evaluation of a single support intervention with healthcare staff. These are outlined in Table 3, grouped according to whether they were primary- or secondary-level interventions (no papers evaluating tertiary interventions were identified). Of these 27 intervention papers, 11 examined intervention effectiveness and were included at Stage 2 of the review to address research question 3.

Table 3: Individual interventions to support healthcare staff coping with patient safety incidents - reported in empirical research papers

Support Level	Intervention Name Description	Study Reference	Setting; *healthcare staff sample*
PRIMARY	The 'When Things Go Wrong' Curriculum	[70]	University; *Medical students*
	'Interactive teaching session addressing personal and professional effects of medical errors'	[61]	University; *Medical students*
	Adverse Events Committee Program: annual 'Safety Education Workshop'	[71]	Hospital; *Psychiatry Residents*
	'Resilience coaching intervention to prepare UK healthcare professionals for the occurrence of stressful events'	[59, 72]	University, Hospital; *Multiple disciplines (qualified staff, trainees)*
	'Mitigating impact in second victims' online programme	[60]	Online [hospital]; *Multiple disciplines*
SECONDARY	Adverse Events Committee Program	[71]	Hospital; *Psychiatry Residents*
	Online, 10-chapter e-book reference guide for responding to & supporting those involved in an incident	[73]	Mental health organisation; *All staff*
	University of Missouri Health Care's 'forYOU' peer support program (wholly or modelled on, using Scott Three-tiered model of Second Victim Support [40])	[40, 63, 66, 67, 69, 74-78]	Hospital, incl. outpatients [64, 66]; *OBGYN staff* [67, 78], *Paediatric staff* [63], *Pharmacy staff* [77], *Neonatal ICU staff* [76], *all staff* [40, 69, 74, 75]
	The Brigham and Women's Hospital Peer Support Service	[79, 80]	Hospital; *Surgeons & trainees* [79], *All staff* [80]
	The Resilience in Stressful Events (RISE) program.	[62, 65, 81, 82]	Hospital; *paediatric staff* [81, 82], *nurses* [62, 65].
	'Swaddle' second victim staff support program (hybrid of above programs)	[83]	Hospital; *All staff*
	Certified Registered Nurse Anaesthetist Peer Support Program	[68]	Hospital; *Certified Registered Nurse Anaesthetists*

For peer review only - http://bmjopen.bmj.com/site/about/guidelines.xhtml

	The ‘Buddy Study’ peer support program	[84]	Hospital; *OBGYN midwives; ER & internal medicine physicians.*
TERTIARY	None		

Stage 2 Review Findings

How effective are existing support mechanisms/interventions designed to: (a) prevent poor psychological outcomes for healthcare staff after PSIs (primary-prevention); (b) support staff in the aftermath of PSIs (secondary-prevention); offer psychological support for those affected by involvement in PSIs (tertiary-intervention) (Research Question 3).

Characteristics of studies included at Stage 2

Eleven studies were included at this stage and are summarised in the Supplemental Table provided. Of these 11 studies of intervention effectiveness, three featured primary-level interventions and eight involved secondary level interventions. There were no papers of tertiary level support. Most were conducted in the United States [n=7: 61, 62, 63-65, 67, 68], followed by Australia [66], the UK [59], Spain [60] and Indonesia [69]. Studies were quantitative [n=9: 60, 61, 63, 64, 65, 67-69] and mixed methods [59, 62]. Samples included uni-disciplinary [59, 61, 62, 65, 68] and multi-disciplinary groups in a specific department [63, 67] or whole organisation [60, 64, 66, 69].

Intervention users were compared with non-users in around a third of studies [62, 66, 67, 69]. A range of follow-ups was included in the sample: from a single survey pre- and post-implementation [63, 66, 68], to multiple follow-ups (3- and 6-months post intervention [67]; 3- and 9-months post launch [64]; 1- and 2-years post launch [69]. Two studies involved a single post-implementation survey [62, 65].

Three studies featured primary-level interventions [59-61]. The formats of these intervention are outlined in the Supplemental Table provided. All aimed to raise awareness of the prevalence of adverse events, understanding of their personal and professional impact, to

For peer review only - http://bmjopen.bmj.com/site/about/guidelines.xhtml

prepare participants for involvement in an adverse event and ensure they knew how to access resources and support after a PSI. In addition, Mira et al. [60] targeted practical, personal preparation, including how to inform patients and what to say/not say to colleagues and Johnson et al. [59] targeted the development of participants' cognitive flexibility, self-esteem and positive attributional style. All primary-level intervention studies measured changes in users' knowledge (of patient safety incidents, second victimhood and the impact, appropriate responses/actions in the aftermath [60]; of resilience, coping strategies, personal strengths [59]; of medical error leading to physician burnout [61]), two explicitly measured changes in confidence to cope with adverse events [59, 61] and one measured resilience to cope with future events [59]. Changes in awareness of support resources and attitudes to error were also measured by one [61]. Purpose-designed questionnaires/tests were used in all cases, supplemented in one [59] by the Brief Resilience Scale [85].

Eight studies featured secondary-level interventions [62-69], all peer support programmes which aimed to offer tailored, flexible support to staff in the aftermath of an adverse event, with referral to more specialist psychological support if required/desired (for formats, see Supplemental Table). The scope varies between programmes. One programme [66] was reported as part of a wider just culture organisational change initiative. Specific programme goals differed, including 'to provide timely support' after a stressful event [65], 'to decrease second victim distress' [68] and 'to mitigate challenges and improve the wellbeing of staff' immediately after an incident, and, longer term, enhancing resilience, self-care and 'connections with colleagues and the organisation' [66]. Five papers [63, 64, 66, 67, 69] feature programmes based on or informed by the University of Missouri Health Care 'forYOU' framework (grounded in Scott's three-tiered interventional model of support), which aims to promote the healthcare professional's transition through emotional recovery after an incident [40, p. 238]. Some included programmes [63, 64, 66] have slightly adapted

For peer review only - http://bmjopen.bmj.com/site/about/guidelines.xhtml

this forYOU model to suit their local needs (see Supplemental Table). Two papers relate to the Resilience in Stressful Events (RISE) programme [62, 65], and the final study features the Clinical Registered Nurse Anaesthetist (CRNA) peer support program [68].

In terms of outcomes measures used in secondary-level intervention studies, four [63, 64, 66, 68] measured changes in levels of psychological and physical distress, perceptions of colleague, supervisor and institutional support, professional self-efficacy, absenteeism and turnover intentions, using the Second Victim Experience and Support Tool (SVEST) [86]. One [67] measured changes in current stage of recovery after a PSI based on Scott and colleagues' [7] six-stage second victim recovery trajectory. It is unclear whether this team used an existing or purpose-designed tool. Differences in burnout, job satisfaction and resilience were investigated in one study [62], using a combination of existing measures (abridged Maslach Burnout Inventory [87] , Job Satisfaction Scale [88], Brief Resilience Scale [85]). Two studies examined organisational level data: changes in perceptions of organisational culture post implementation [69], using the Hospital Survey on Patient Safety Culture [89] and costs associated with implementing versus not implementing the programme, through modelling probable savings from reduced sickness absence and turnover [65].

Study Quality

Study quality was variable. Insufficient detail was an overarching issue across these intervention studies, particularly with regard to enabling study replication. Theoretical underpinning was limited (most studies only referred to second victimhood in their introduction) and insufficient description tended to be provided of samples and sampling strategies in relation to aims. There was only one randomised trial in the sample, meaning that most studies did not use a comparison group and that staff/worksites were assigned non-randomly to interventions when testing for effectiveness. These are sources of potential bias.

For peer review only

Self-reported data were relied upon in the majority of cases, increasing the potential for response bias. Evidence of stakeholder engagement in design was limited across studies, although most seemed to be led by clinical teams. We did not exclude any study based on its quality appraisal outcome.

Our TIDieR assessment of the intervention descriptions also revealed that theoretical underpinnings were provided in less than half of studies included at Stage 2. Details regarding intervention materials, associated procedures/activities and facilitators were well described overall. Just one study explicitly mentioned fidelity assessment of intervention delivery, although a small number of studies referenced regular facilitator meetings for supervision, debriefing, learning and continuous improvement.

How effective are existing ***primary-prevention*** *interventions?*

Evidence was largely positive for the effectiveness of the three primary-level interventions in preparing healthcare staff and trainees for exposure to adverse events. All three studies reported significant improvements in targeted outcomes, particularly in relation to knowledge and awareness of resources in relation to the second victim phenomena (see Supplemental Table). Users of Mira et al.'s [60] MISE online training programme demonstrated significant improvements in their knowledge of basic patient safety concepts, the prevalence and nature of adverse events. Similarly, medical students participating in Musunur et al.'s [61] 1-hour teaching session training demonstrated significant improvements in confidence in their ability to recognise and cope with medical error and in their awareness of resources to cope with and report medical error. Participants in Johnson et al.'s [59] half day workshop also showed significant improvements in confidence with coping with adverse events, knowledge about resilience factors, coping strategies and awareness of personal strengths, which were maintained 4-6 weeks after the final workshop.

For peer review only - http://bmjopen.bmj.com/site/about/guidelines.xhtml

How effective are existing ***secondary-prevention*** *interventions?*

Evidence for the effectiveness of secondary level peer-support based interventions was more mixed. With regards to adaptations of the ForYOU programme: Klatt et al [64] identified no significant changes on any SVEST dimension ratings when following up at 3 and 9 months after their programme launch. In the only randomised trial in the sample, Rivera-Chiauzzi et al. [67] found no significant differences at 6 months post-enrolment in the study, between their intervention and control groups regarding ability to recover from an adverse event into the optimal 'thriving' recovery stage: both groups of staff recovered equally well.

More positive findings were reported, however, in Finney et al. [63] and Wijaya, Mohamad and Hafizzurachman [69]. Finney et al. [63] found significant changes in the institutional support and turnover intentions dimensions in SVEST: they report that, post implementation, 'inadequate' ratings of institutional support and turnover intentions fell significantly (however, p values in the article body text (p=0.014 - significant) and Table 2 (p=0.09 – not significant) do not correspond). Wijaya and colleagues [69] reported significant increases in Hospital Survey on Patient Safety Culture (HSOPSC) ratings in the intervention hospital one year after introducing the programme, which were significantly higher than those of staff in the control hospital. This improved safety culture was sustained two years post-introduction of the programme. Morris et al. [66] found no significant differences between 'Always There' programme users and non-users one year post-launch on any overall dimension of SVEST, although users did rate two (out of three) items in the 'institutional support' dimension significantly higher than non-users.

Turning to the RISE programme, two studies evaluated its impact specifically on the nursing workforce in their hospital. Moran et al. [65] calculated that having the RISE programme could save a hospital $1.81 million per year in reducing turnover and absence. Connors and colleagues [62] found no significant difference in job satisfaction between either

For peer review only - http://bmjopen.bmj.com/site/about/guidelines.xhtml

nurse users and non-users, or between nurse leader activators and non-activators. Compared to non-users, nurses using RISE were significantly more likely to report higher levels of resilience and some level of burnout. Nurse leaders who did not activate RISE for their staff reported significantly higher resilience than those who did.

Finally, Thompson et al.'s [68] quality improvement project found no statistically significant differences between pre- and post-intervention implementation SVEST scores of all departmental certified registered nurse anaesthetists surveyed, after one month of implementing their peer support programme.

DISCUSSION

We used a widely-respected, multi-level stress prevention model [43] to map the empirical evidence for what type of support healthcare professionals *want* for PSIs, what they tend to *receive*, and what organisational strategies are *effective*. Our findings revealed that healthcare professionals want organisational support at multiple levels: a supportive and learning oriented culture (primary-prevention level support), peer support, practical support and guidance, post-incident debriefing to clarify and process what happened (secondary-prevention level support) and access to professional mental health support (tertiary level). However, there is limited evidence that organisations are giving staff what they want: overwhelmingly, organisational support for PSIs is perceived as inadequate, non-existent or dependent on one's organisational status. Where support *is* provided, secondary level approaches dominate - largely at a local level from peers and one's line manager. There is a gap in primary level (preparatory, proactive, preventive) approaches which tend to be missing from staff reports.

For healthcare organisations seeking to invest in evidence-based interventions to support staff with PSIs, effectiveness data are lacking. There is more robust evidence for the

For peer review only - http://bmjopen.bmj.com/site/about/guidelines.xhtml

effectiveness of ***primary interventions*** designed to equip and prepare staff for involvement in PSIs, than for ***secondary interventions***, which aim to mobilise support and promote staff coping *after* involvement in a PSI. However, despite this, secondary level interventions dominate the literature, particularly the popular peer support programmes which have mixed evidence for their effectiveness. No studies were identified featuring a tertiary intervention, that is, individualised support for staff experiencing severe and/or longer-term emotional sequelae and/or disturbed ability to function after a PSI. Whilst there is compelling evidence more broadly that Cognitive Behavioural Therapy and Eye Movement Desensitisation and Reprocessing are effective in treating PTSD [90], data are not available to be able to recommend their particular effectiveness in alleviating second victim-related sequelae.

Our Stage 1 findings regarding what staff *want* and what they *receive* reflect those of previous reviews. Their overall desire for comprehensive support, within a just culture, to be able to navigate PSIs adaptively is consistent with Seys and colleagues' [42] conclusion that organisational action plans need to incorporate preventive strategies, peer support and professional and clinical support. Our finding that staff rarely *receive* formal organisational support is also consistent with previous reviews [e.g., 39, 48, 50], and a recent scoping review examining support for surgeons [91]: support tends to be 'piecemeal' and informal, with staff mostly relying solely on the good will and support of their clinical colleagues in the aftermath.

Our Stage 2 finding that there is a lack of robust empirical evidence for the effectiveness of specific PSI-related interventions is also in line with previous research [39, 46-48, 50]. As others have noted, this may be attributable, in part, to being a relatively young research field and an initial focus on intervention feasibility, acceptability and implementation challenges [47, 84]. We identified only one RCT. Few included a theoretical underpinning or basis for their design. Organisational level outcome data (retention, sickness

For peer review only - http://bmjopen.bmj.com/site/about/guidelines.xhtml

absence, cost effectiveness, organisational culture) featured in just two studies [65, 69]. It is becoming increasingly important for organisations, particularly post-COVID, to have reliable intelligence about how best to support staff after PSIs and what works, to justify investment to stakeholders including staff. Therefore, there is a need for the field to evolve rapidly and for robust studies to be funded.

Collectively, the wide range of healthcare staff voices across our included studies corroborate the need for organisational support for PSIs to be multi-levelled and comprehensive. This finding supports not only previous reviews of second victim support [41, 42, 48-50], but also reflects the wider occupational stress literature more generally [e.g., 43, 44, 45, 92]. A lack of primary level interventions in healthcare organisations to help staff with PSIs is perhaps not surprising: organisations generally tend to prefer secondary and tertiary interventions [43]. Targeting or changing individuals is considered easier, more cost effective, tangible/visible and less disruptive than changing organisations, e.g., changing organisational culture, working conditions, job designs [43, p.10]. Indeed, it is noteworthy that the interventions and approaches within our sample tended to target the individual, rather than focusing on teams (where incidents happen and must be dealt with). As Busch and colleagues [50] surmise in their systematic review of second victim support interventions, the emphasis appears to be on reducing distress after a PSI, trying to "fix what has gone wrong in the healthcare provider" (p.12).

However, disregarding primary level support may be problematic for two reasons. Firstly, research surrounding broader work-stress interventions suggests that primary interventions may be more effective at stress reduction than secondary or tertiary approaches [93, 94]. Secondly, multi-level intervention strategies combining individual and organisational level interventions are more effective than uni-level interventions alone [93, 95, 96], therefore organisations risk poorer returns on their investment if they fail to adopt a

For peer review only - http://bmjopen.bmj.com/site/about/guidelines.xhtml

comprehensive approach. Primary level interventions, particularly those addressing improvements in culture and working practices, appear to be especially important in healthcare settings, because of the significant impact the presiding patient safety culture has on staff's recovery trajectory after a PSI [16]. Indeed, previous reviews have repeatedly underlined that a systemic approach to patient safety and a just, restorative culture are essential foundations for the provision of quality staff support for PSIs [39, 41, 42, 46, 50]. A recent scoping review of factors underpinning the effective implementation of interventions to support staff with PSIs (and other stressful clinical events) also identified that an organisational culture based on trust, non-judgement and multi-disciplinary action is vital for success [97].

Organisations that fail to invest in primary level support – specifically, implementing a fair and just organisational culture - but provide a formal peer support programme (secondary level) or EAP (Tertiary level), risk staff viewing these provisions as 'tokenistic', or as signifiers that the organisation has abdicated responsibility for its staff because it is not tackling poor culture which is a major source of distress [98]. In fact, when employers ask their staff to identify factors that would have the greatest impact on their wellbeing at work, they overwhelmingly suggest better communication, staffing and interpersonal connectivity, rather than issues targeted by wellbeing programmes [39]. Themes of injustice and resentment were also apparent within many included studies in the review, which, independent of involvement in a PSI, are risk factors for turnover intentions in healthcare staff [99].

In line with previous research, our review found that peer support is a highly desired and commonly received form of support [15, 31, 40, 49, 50]. However, we also identified considerable ambiguity around the term 'peer support' within included studies: it was often unclear whether it referred to informal support from team colleagues or a formal peer support

For peer review only - http://bmjopen.bmj.com/site/about/guidelines.xhtml

programme. Given that formal peer support programmes are the most common organisational intervention provided, this has highlighted a need to ensure that such programmes are meeting the right requirement, particularly since evidence for effectiveness remains elusive [47]. For example, if a model offers peer support from outside one's team, but one's home team is unsupportive and one's leader is critical, then ongoing, day-to-day recovery in that location is likely to be more difficult.

Perhaps unsurprisingly, manager support emerged from this review as the second most commonly received form of organisational support, although there were also numerous accounts of inadequate managerial support compounding feelings of isolation and hindering recovery. This was also found in our related review [31]. The way in which supervisors or leaders treat their staff is crucially important, because, to staff, they 'embody' the organisation: staff form perceptions and experiences of their organisation from their supervisors/leaders behaviour and treatment of them [100]. Leadership quality has long been associated in the field of occupational health psychology with both positive outcomes, such as staff wellbeing, psychological safety, and negative outcomes, including stress, heart disease and workplace incidents [94]. In healthcare in particular, compassionate leadership is being increasingly linked to staff wellbeing and positive safety culture [101, 102]. The vital contribution leaders make to the implementation success (or failure) of organisational support programmes for PSIs was also highlighted recently by Guerra-Paiva et al. [97]. Given managers' pivotal roles in supporting staff with PSIs, there is a strong case for organisations to provide compassionate leadership development in this area within their multi-level PSI-support strategy, as a proactive, preventive, primary level occupational health psychology intervention [94].

Of significance is that the vast majority of intervention developers and paper authors within our Stage 2 sample are clinicians. Secondary level interventions dominate; fewer

For peer review only - http://bmjopen.bmj.com/site/about/guidelines.xhtml

studies address primary level prevention of second victimhood. This suggests that most interventions are pragmatic, responsive solutions to meet needs recognised at ground level by clinicians themselves. It also suggests that the recognition of and ownership for second victimhood as a serious, intrinsic occupational health risk to be addressed is located in clinical or patient safety teams; it does not reside with organisational senior leadership teams. However, as other reviewers have identified [41, 42, 49, 50] building the much-needed comprehensive multi-level strategies requires commitment from senior leadership teams of resources, funding and time; it cannot solely be responsibility of patient safety teams or local clinical departments.

Strengths and limitations of this study

To our knowledge this is the first systematic review to apply an established, occupational health stress prevention model to examine second victim requirements and organisational support solutions. This model is likely to be familiar to Organisational Development, Human Resources and Occupational Health teams in healthcare organisations, which we hope will aid dissemination and consideration at senior organisational levels. A second strength of the review is that it specifically addressed intervention effectiveness.

However, a number of limitations must be acknowledged. First, despite including a medical librarian on our review team, it is possible that our search strategy did not locate all the relevant studies, due to focusing on papers in English, a broad area and outcomes, or our choice and number of bibliographic search engines. As other reviewers have noted [e.g., 47] nomenclature varies significantly within this literature, therefore we may have missed important papers which featured more unusual or unspecific terms. For these reasons, we may also have excluded relevant papers at the title and abstract screening stage, particularly those featuring primary prevention approaches, because these may have had broader goals and included many different types of organisational process.

For peer review only - http://bmjopen.bmj.com/site/about/guidelines.xhtml

660 Second, whilst starting our searches from 2010 aided the review focus and
661 manageability, it may have omitted relevant older papers, and since our last update in
662 December 2022, other relevant papers may have been published that we have not captured.
663 We are aware of two recent empirical papers related to the Austrian 'KOHi' collegial help
664 programme [103, 104] which is modelled on RISE and forYOU. However, neither paper
665 reports outcome/effectiveness data, therefore would not have been included in Stage 2 of our
666 review.

667 A third limitation is that some methodological steps we undertook are known to be
668 prone to subjective judgment and lack of transparency, particularly data extraction, quality
669 assessment and narrative synthesis. To address this, screening, data extraction and assessment
670 of methodological quality were conducted independently by two reviewers, and 20% of
671 completed data extraction templates were independently double checked by a second
672 reviewer. Regular meetings were held throughout to discuss any resolve disagreements, to
673 review and refine emerging findings, and outcomes documented to provide an audit trail of
674 our decisions. Our reporting of Stage 2 findings was guided by the SWiM framework.

675 Finally, our sample included studies that were heterogeneous in terms of design,
676 sample sizes, data description and analysis, outcomes measured. A high proportion of papers
677 featured cross sectional data collected through self-report questionnaires, which are
678 susceptible to non-response and recall bias. Study quality was also variable. As other
679 reviewers have highlighted [47, 50], these factors currently render robust meta-analyses of
680 interventions impossible, and there is a need for the field to agree on consensus on
681 appropriate outcome measures and tools.

682 **Practical Implications**

683 The findings from this systematic review provide compelling evidence that healthcare
684 professionals are routinely exposed to compounded harm after PSIs because of a lack of

For peer review only

685 institutional-level responsibility and support. A quality PSI support programme should be an integral part of every healthcare organisation's strategy, policies and practices, rather than operating through the goodwill of passionate volunteers. Such programmes require the full support of senior leadership teams, with strong investment and promotion for sustainability [41, 49, 50, 97]. Providing quality support to healthcare professionals for PSIs not only promotes adaptive recovery: it also has the potential to reduce defensive practice, promote healing, open up possibilities for restorative approaches, and importantly, retain staff at a time when there are shortages of trained healthcare professionals worldwide.

693 This review provides organisations with robust, evidence-based data on what staff want, where there are currently gaps and what works – to inform the development of PSI support initiatives. It is hoped that presenting the findings through a familiar and respected organisational stress management framework may support a wider dialogue of the need to provide quality support for healthcare professionals, beyond patient safety and clinical teams to senior leadership teams, including Organisational Development, Human Resources, Occupational Health and Educational leaders.

700 Conclusion

701 Despite 20 years of investment to reduce the number of PSIs, they continue to persist [105]: 'to err is human' [106]. Given the fact that healthcare organisations can never entirely remove this intrinsic hazard, the importance of having an effective, comprehensive institutional support strategy in place for patient safety incidents is evident, to aid adaptive recovery, minimise distress, sickness absence and turnover [6]. There is an incontrovertible, pressing need for healthcare organisations to address the multi-faceted occupational health risk for their staff of being involved in a PSI. Organisations need reliable evidence about what interventions are effective, however this is currently lacking. In order to advance the field, we require more methodologically robust studies, which describe theoretical bases for

For peer review only - http://bmjopen.bmj.com/site/about/guidelines.xhtml

interventions and involve in their design comparison groups, randomisation, organisational- and individual-level outcomes. In addition, more research is needed into primary and tertiary-level interventions to help healthcare professionals with PSIs.

FIGURE LEGENDS

Fig.1: This diagram shows the systematic process we followed to include papers we captured in our review.

Fig 2: This diagram illustrates a multi-level pyramid of support organisations can provide to healthcare staff, with corresponding goals, to aid adaptive coping with Patient Safety Incidents. The fundamental importance of Primary level support is reflected in its foundational, base position in the pyramid. Tertiary support – i.e., tailored, one-to-one care – is conceptualised as the pyramid's summit to reflect that it supplements Primary- and Secondary-level support, rather than stands alone.

SUPPORTING INFORMATION

S1 File. PRISMA checklists.

S2 File. SWiM checklist.

S3 File. Search criteria.

S4 File. Detailed narrative summary findings and reference list for surveys and qualitative studies included at Stage 1.

S5 File. Description of fourth and fifth most desired and experienced forms of support.

Supplemental Table. Summary of included studies at Stage 2.

For peer review only

For peer review only - http://bmjopen.bmj.com/site/about/guidelines.xhtml

ACKNOWLEDGEMENTS

This paper presents independent research supported by the National Institute for Health and Care Research (NIHR) Yorkshire and Humber Patient Safety Translational Research Centre and Patient Safety Research Collaboration. The views expressed are those of the authors, and not necessarily those of the NIHR or the Department of Health and Social Care. Our sincere thanks to Dr. Jane Heyhoe (JH) who supported the team with this research before her departure from academia, and to Stephen Mears, University of New South Wales, Australia for his help with the database searches.

AUTHOR CONTRIBUTIONS

Conceived and designed the experiments: RSE RH RS ES JH RL. Performed the experiments: RSE RH RS ES JH HH RL. Analyzed the data: RSE RH RS ES JH HH MME RL. Contributed to materials/analysis tools: RSE RH RS ES JH HH MME RL. Wrote the paper: RSE RL RH. Guarantor: RSE.

REFERENCES

1. Mahase E. Invest in health workforce or risk collapse, WHO warns governments. BMJ. 2023;380:713. https://doi.org/10.1136/bmj.p713
2. World Health Organization Regional Office for Europe. Health and care workforce in Europe: time to act. https://www.who.int/europe/publications/i/item/9789289058339. 2022 14 September 2022 .https://www.who.int/europe/publications/i/item/9789289058339
3. Castro Lopes S, Guerra-Arias M, Buchan J, Pozo-Martin F, Nove A. A rapid review of the rate of attrition from the health workforce. Human Resources for Health. 2017;15(1):1-9. https://doi.org/10.1186/s12960-017-0195-2
4. Ratwatte M. The NHS workforce crisis is a retention crisis. BMJ. 2023;380:602. https://doi.org/10.1136/bmj.p602

For peer review only - http://bmjopen.bmj.com/site/about/guidelines.xhtml

5. Downey E, Fokeladeh HS, Catton H. What the COVID-19 pandemic has exposed: The findings of five global health workforce professions. Geneva: World Health Organization.; 2023. Report No.: 9240070184
6. Burlison JD, Quillivan RR, Scott SD, Johnson S, Hoffman JM. The effects of the second victim phenomenon on work-related outcomes: connecting self-reported caregiver distress to turnover intentions and absenteeism. Journal of Patient Safety. 2021;17(3):195-9. 10.1097/PTS.0000000000000301
7. Scott SD, Hirschinger LE, Cox KR, McCoig M, Brandt J, Hall LW. The natural history of recovery for the healthcare provider "second victim" after adverse patient events. BMJ Quality & Safety. 2009;18(5):325-30. https://doi.org/10.1136/qshc.2009.032870
8. Van Gerven E, Vander Elst T, Vandenbroeck S, et al. Increased risk of burnout for physicians and nurses involved in a patient safety incident. Medical Care. 2016;54(10):937-43. 10.1097/MLR.0000000000000582
9. Martens J, Van Gerven E, Lannoy K, et al. Serious reportable events within the inpatient mental health care: Impact on physicians and nurses. Revista de Calidad Asistencial. 2016;31:26-33. https://doi.org/10.1016/j.cali.2016.04.004
10. NHS England. Patient Safety. 2023 [Available from: https://www.england.nhs.uk/patient-safety/].
11. Vincent C, Neale G, Woloshynowych M. Adverse events in British hospitals: preliminary retrospective record review. BMJ. 2001;322(7285):517-9. https://doi.org/10.1136/bmj.322.7285.517
12. Connolly W, Rafter N, Conroy RM, Stuart C, Hickey A, Williams DJ. The Irish National Adverse Event Study-2 (INAES-2): longitudinal trends in adverse event rates in the Irish healthcare system. BMJ Quality & Safety. 2021;30(7):547-58. https://doi.org/10.1136/bmjqs-2020-011122
13. Michel P, Brami J, Chanelière M, et al. Patient safety incidents are common in primary care: A national prospective active incident reporting survey. PLOS One. 2017;12(2):e0165455. https://doi.org/10.1371/journal.pone.0165455
14. Panagioti M, Stokes J, Esmail A, et al. Multimorbidity and patient safety incidents in primary care: a systematic review and meta-analysis. PLOS One. 2015;10(8):e0135947. https://doi.org/10.1371/journal.pone.0135947

For peer review only - http://bmjopen.bmj.com/site/about/guidelines.xhtml

15. Wu AW, Shapiro J, Harrison R, et al. The impact of adverse events on clinicians: what's in a name? Journal of Patient Safety. 2020;16(1):65-72. 10.1097/PTS.0000000000000256
16. Quillivan RR, Burlison JD, Browne EK, Scott SD, Hoffman JM. Patient safety culture and the second victim phenomenon: connecting culture to staff distress in nurses. The Joint Commission Journal on Quality and Patient Safety. 2016;42(8):377-86. 10.1016/s1553-7250(16)42053-2
17. Murray JS, Clifford J, Larson S, Lee JK, Sculli GL. Implementing just culture to improve patient safety. Military Medicine. 2023;188(7-8):1596-9. https://doi.org/10.1093/milmed/usac115
18. van Marum S, Verhoeven D, de Rooy D. The barriers and enhancers to trust in a just culture in hospital settings: a systematic review. Journal of Patient Safety. 2022;18(7):e1067-e75. 10.1097/PTS.0000000000001012
19. Turner K, Sveticic J, Grice D, et al. Restorative Just Culture significantly improves stakeholder inclusion, second victim experiences and quality of recommendations in incident responses. J Hosp Adm. 2022;11(2):8-17. https://doi.org/10.5430/jha.v11n2p8
20. Kaur M, De Boer RJ, Oates A, Rafferty J, Dekker S, editors. Restorative just culture: a study of the practical and economic effects of implementing restorative justice in an NHS trust. International Cross-industry Safety Conference (ICSC) - European STAMP Workshop & Conference (ESWC) (ICSC-ESWC 2018); 2019: EDP Sciences. https://doi.org/10.1051/matecconf/201927301007
21. Kim S-A, Kim E-M, Lee J-R. Causes of nurses' second victim distress: An objective analysis. Quality Management in Health Care. 2022;31(3):122-9. 10.1097/QMH.0000000000000330
22. Ramsey L, McHugh S, Simms-Ellis R, Perfetto K, O'Hara JK. Patient and family involvement in serious incident investigations from the perspectives of key stakeholders: a review of the qualitative evidence. Journal of Patient Safety. 2022;18(8):e1203. 10.1097/PTS.0000000000001054
23. Wu AW. Medical error: the second victim: the doctor who makes the mistake needs help too. BMJ. 2000;320(7237):726-7. https://doi.org/10.1136/bmj.320.7237.726
24. Rivera-Chiauzzi E, Finney RE, Riggan KA, et al. Understanding the second victim experience among multidisciplinary providers in obstetrics and gynecology. Journal of Patient Safety. 2022;18(2):e463-e9. 10.1097/PTS.0000000000000850

For peer review only

For peer review only - http://bmjopen.bmj.com/site/about/guidelines.xhtml

25. Jain G, Sharma D, Agarwal P, et al. "Second Victim" syndrome among the surgeons from South Asia. Indian Journal of Surgery. 2022;84(1):40-6. https://doi.org/10.1007/s12262-021-02793-3
26. Aubin D, King S. The healthcare environment: a perfect ecosystem for growing shame. Healthcare Quarterly. 2018;20:31-6.
27. Coughlan B, Powell D, Higgins M. The second victim: a review. European Journal of Obstetrics & Gynecology and Reproductive Biology. 2017;213:11-6. https://doi.org/10.1016/j.ejogrb.2017.04.002
28. Edrees HH, Paine LA, Feroli ER, Wu AW. Health care workers as second victims of medical errors. Pol Arch Med Wewn. 2011;121(4):101-8.
29. Harrison R, Lee H, Sharma A. A survey of the impact of patient adverse events and near misses on anaesthetists in Australia and New Zealand. Anaesthesia and Intensive Care. 2018;46(5):510-5. https://doi.org/10.1177/0310057X1804600513
30. Harrison R, Lawton R, Perlo J, Gardner P, Armitage G, Shapiro J. Emotion and coping in the aftermath of medical error. Journal of Patient Safety. 2015;11(1):28-35. 10.1097/PTS.0b013e3182979b6f
31. Harrison R, Johnson J, McMullan RD, et al. Toward Constructive Change After Making a Medical Error: Recovery From Situations of Error Theory as a Psychosocial Model for Clinician Recovery. Journal of Patient Safety. 2022;18(6):587-604. 10.1097/PTS.0000000000001038
32. Naya K, Aikawa G, Ouchi A, et al. Second victim syndrome in intensive care unit healthcare workers: A systematic review and meta-analysis on types, prevalence, risk factors, and recovery time. PLOS One. 2023;18(10):e0292108. https://doi.org/10.1371/journal.pone.0292108
33. Nydoo P, Pillay BJ, Naicker T, Moodley J. The second victim phenomenon in health care: a literature review. Scandinavian Journal of Public Health. 2020;48(6):629-37. https://doi.org/10.1177/140349481985
34. Vanhaecht K, Seys D, Schouten L, et al. Duration of second victim symptoms in the aftermath of a patient safety incident and association with the level of patient harm: a cross-sectional study in the Netherlands. BMJ Open. 2019;9(7):e029923. https://doi.org/10.1136/bmjopen-2019-029923
35. Wahlberg Å, Högberg U, Emmelin M. Left alone with the emotional surge–A qualitative study of midwives' and obstetricians' experiences of severe events on the labour ward.

For peer review only - http://bmjopen.bmj.com/site/about/guidelines.xhtml

Sexual & Reproductive Healthcare. 2020;23:100483. https://doi.org/10.1016/j.srhc.2019.100483

36. American Psychiatric Association. Trauma- and Stressor-Related Disorders. . Diagnostic and Statistical Manual of Mental Disorders (5th edn, text rev) 2022.
37. Owoc J, Mańczak M, Jabłońska M, Tombarkiewicz M, Olszewski R. Association between physician burnout and self-reported errors: meta-analysis. Journal of Patient Safety. 2022;18(1):e180-e8. 10.1097/PTS.0000000000000724
38. Pellino IM, Pellino G. Consequences of defensive medicine, second victims, and clinical-judicial syndrome on surgeons' medical practice and on health service. Updates in Surgery. 2015;67(4):331-7. https://doi.org/10.1007/s13304-015-0338-8
39. Werthman JA, Brown A, Cole I, et al. Second victim phenomenon and nursing support: An integrative review. Journal of Radiology Nursing. 2021;40(2):139-45. https://doi.org/10.1016/j.jradnu.2020.12.014
40. Scott SD, Hirschinger LE, Cox KR, et al. Caring for our own: deploying a systemwide second victim rapid response team. Joint Commission Journal on Quality and Patient Safety. 2010;36(5):233-40. https://doi.org/10.1016/S1553-7250(10)36038-7
41. Seys D, Scott S, Wu A, et al. Supporting involved health care professionals (second victims) following an adverse health event: a literature review. International Journal of Nursing Studies. 2013;50(5):678-87. https://doi.org/10.1016/j.ijnurstu.2012.07.006
42. Seys D, Panella M, Russotto S, et al. In search of an international multidimensional action plan for second victim support: a narrative review. BMC Health Services Research. 2023;23(1):816. https://doi.org/10.1186/s12913-023-09637-8
43. Cooper CL, Cartwright S. An intervention strategy for workplace stress. Journal of Psychosomatic Research. 1997;43(1):7-16. https://doi.org/10.1016/S0022-3999(96)00392-3
44. Quick JD, Quick JC, Nelson DL. The theory of preventive stress management in organizations. In: Cooper CL, editor. Theories of organizational stress. 1. Oxford, UK: Oxford University Press; 1998. p. 246-68.
45. Martin A, Karanika-Murray M, Biron C, Sanderson K. The psychosocial work environment, employee mental health and organizational interventions: Improving research and practice by taking a multilevel approach. Stress and Health. 2016;32(3):201-15. https://doi.org/10.1002/smi.2593

For peer review only

For peer review only - http://bmjopen.bmj.com/site/about/guidelines.xhtml

46. Quadrado ERS, Tronchin DMR, Maia FdOM. Strategies to support health professionals in the condition of second victim: Scoping review. Revista da Escola de Enfermagem da USP. 2021;55. https://doi.org/10.1590/S1980-220X2019011803669

47. Wade L, Fitzpatrick E, Williams N, Parker R, Hurley KF. Organizational interventions to support second victims in acute care settings: a scoping study. Journal of Patient Safety. 2022;18(1):e61. 10.1097/PTS.0000000000000704

48. Stone M. Second victim support programs for healthcare organizations. Nursing Management. 2020;51(6):38-45. 10.1097/01.NUMA.0000662664.90688.1d

49. Neft MW. Support Methods for Healthcare Professionals Who Are Second Victims: An Integrative Review. AANA Journal. 2022;90(3).

50. Busch IM, Moretti F, Campagna I, et al. Promoting the psychological well-being of healthcare providers facing the burden of adverse events: A systematic review of second victim support resources. International Journal of Environmental Research and Public Health. 2021;18(10):5080. https://doi.org/10.3390/ijerph18105080

51. Spence GB. Workplace wellbeing programs: if you build it they may NOT come... because it's not what they really need! International Journal of Wellbeing. 2015;5 (2) 109-24. http://dx.doi.org/10.5502/ijw.v5i2.7

52. Popay J, Roberts H, Sowden A, et al. Guidance on the conduct of narrative synthesis in systematic reviews. A product from the ESRC methods programme Version. 2006;1(1):b92.

53. Moher D, Liberati A, Tetzlaff J, Altman DG. Preferred reporting items for systematic reviews and meta-analyses: the PRISMA statement. Annals of Internal Medicine. 2009;151(4):264-9. https://doi.org/10.7326/0003-4819-151-4-200908180-001

54. Conway J, Federico F, Stewart K, Campbell M. Respectful management of serious clinical adverse events. . Cambridge, MA: Institute for Healthcare Improvement; 2010.

55. Sirriyeh R, Lawton R, Gardner P, Armitage G. Coping with medical error: a systematic review of papers to assess the effects of involvement in medical errors on healthcare professionals' psychological well-being. Quality and Safety in Health Care. 2010;19(6):e43-e. https://doi.org/10.1136/qshc.2009.035253

56. Campbell M, McKenzie JE, Sowden A, et al. Synthesis without meta-analysis (SWiM) in systematic reviews: reporting guideline. BMJ. 2020;368. https://doi.org/10.1136/bmj.l6890

For peer review only

57. Hoffmann TC, Glasziou PP, Boutron I, et al. Better reporting of interventions: template for intervention description and replication (TIDieR) checklist and guide. BMJ. 2014;348. https://doi.org/10.1136/bmj.g1687

58. Harrison R, Jones B, Gardner P, Lawton R. Quality assessment with diverse studies (QuADS): an appraisal tool for methodological and reporting quality in systematic reviews of mixed-or multi-method studies. BMC Health Services Research. 2021;21(1):1-20. https://doi.org/10.1186/s12913-021-06122-y

59. Johnson J, Simms-Ellis R, Janes G, et al. Can we prepare healthcare professionals and students for involvement in stressful healthcare events? A mixed-methods evaluation of a resilience training intervention. BMC Health Services Research. 2020;20(1):1-14. https://doi.org/10.1186/s12913-020-05948-2

60. Mira JJ, Carrillo I, Guilabert M, et al. The second victim phenomenon after a clinical error: the design and evaluation of a website to reduce caregivers' emotional responses after a clinical error. Journal of Medical Internet Research. 2017;19(6):e203. https://www.jmir.org/2017/6/e203/

61. Musunur S, Waineo E, Walton E, Deeds K, Levine D. When bad things happen: training medical students to anticipate the aftermath of medical errors. Academic Psychiatry. 2020;44:586-91. https://doi.org/10.1007/s40596-020-01278-x

62. Connors CA, Dukhanin V, March AL, Parks JA, Norvell M, Wu AW. Peer support for nurses as second victims: Resilience, burnout, and job satisfaction. Journal of Patient Safety and Risk Management. 2020;25(1):22-8. https://doi.org/10.1177/2516043519882517

63. Finney RE, Czinski S, Fjerstad K, et al. Evaluation of a second victim peer support program on perceptions of second victim experiences and supportive resources in pediatric clinical specialties using the second victim experience and support tool (SVEST). Journal of Pediatric Nursing. 2021;61:312-7. https://doi.org/10.1016/j.pedn.2021.08.023

64. Klatt TE, Sachs JF, Huang C-C, Pilarski AM. Building a program of expanded peer support for the entire health care team: no one left behind. The Joint Commission Journal on Quality and Patient Safety. 2021;47(12):759-67. https://doi.org/10.1016/j.jcjq.2021.08.010

For peer review only

65. Moran D, Wu AW, Connors C, et al. Cost-benefit analysis of a support program for nursing staff. Journal of Patient Safety. 2020;16(4):e250-e4. 10.1097/PTS.0000000000000376
66. Morris D, Sveticic J, Grice D, Turner K, Graham N. Collaborative approach to supporting staff in a mental healthcare setting:"always there" peer support program. Issues in Mental Health Nursing. 2022;43(1):42-50. https://doi.org/10.1080/01612840.2021.1953651
67. Rivera-Chiauzzi EY, Smith HA, Moore-Murray T, et al. Healing our own: a randomized trial to assess benefits of peer support. Journal of Patient Safety. 2022;18(1):e308-e14. 10.1097/PTS.0000000000000771
68. Thompson M, Hunnicutt R, Broadhead M, Vining B, Aroke EN. Implementation of a certified registered nurse anesthetist second victim peer support program. Journal of Perianesthesia Nursing. 2022;37(2):167-73. e1. https://doi.org/10.1016/j.jopan.2021.05.005
69. Wijaya MI, Mohamad AR, Hafizurrachman M. Second victim support program and patient safety culture: A quasi experimental study in Bali International Medical Centre (BIMC) Hospital. Bali Medical Journal. 2018;7(1):220-6.
70. Bell SK, Moorman DW, Delbanco T. Improving the patient, family, and clinician experience after harmful events: the "when things go wrong" curriculum. Academic Medicine. 2010;85(6):1010-7. 10.1097/ACM.0b013e3181dbedd7
71. Padilla V, Healey J, Salinas J, Bryant C, Muñoz O, Durand D. Residents impacted by adverse events: the development of a pilot committee for responding to and supporting psychiatry residents. Academic Psychiatry. 2021;45:463-6. https://doi.org/10.1007/s40596-020-01348-0
72. Janes G, Harrison R, Johnson J, Simms-Ellis R, Mills T, Lawton R. Multiple meanings of resilience: Health professionals' experiences of a dual element training intervention designed to help them prepare for coping with error. Journal of Evaluation in Clinical Practice. 2022;28(2):315-23. https://doi.org/10.1111/jep.13555
73. Austin DM, Pauley G, Ferkins L. Establishing a Guide for Developing Organizational Support in Healthcare Following a Critical or Sentinel Event. Qualitative Health Research 2022;32(11):1607-19. https://doi.org/10.1177/10497323221111644
74. Cobos-Vargas A, Pérez-Pérez P, Núñez-Núñez M, Casado-Fernández E, Bueno-Cavanillas A. Second Victim Support at the Core of Severe Adverse Event Investigation.

For peer review only

For peer review only - http://bmjopen.bmj.com/site/about/guidelines.xhtml

International Journal of Environmental Research and Public Health. 2022;19(24):16850. https://doi.org/10.3390/ijerph192416850

75. Merandi J, Liao N, Lewe D, et al. Deployment of a second victim peer support program: a replication study. Pediatric Quality & Safety. 2017;2:e031(4). 10.1097/pq9.0000000000000031

76. Merandi J, Winning AM, Liao N, Rogers E, Lewe D, Gerhardt CA. Implementation of a second victim program in the neonatal intensive care unit: an interim analysis of employee satisfaction. Journal of Patient Safety and Risk Management. 2018;23(6):231-8. https://doi.org/10.1177/2516043518809457

77. Krzan KD, Merandi J, Morvay S, Mirtallo J. Implementation of a "second victim" program in a pediatric hospital. American Journal of Health-System Pharmacy. 2015;72(7):563-7. https://doi.org/10.2146/ajhp140650

78. Finney RE, Jacob A, Johnson J, Messner H, Pulus B, Sviggum H. Implementation of a Second Victim Peer Support Program in a Large Anesthesia Department. AANA Journal. 2021;89(3):235-44.

79. El Hechi MW, Bohnen JD, Westfal M, et al. Design and impact of a novel surgery-specific second victim peer support program. Journal of the American College of Surgeons. 2020;230(6):926-33. https://doi.org/10.1016/j.jamcollsurg.2019.10.015

80. Keyser EA, Weir LF, Valdez MM, Aden JK, Matos RI. Extending peer support across the military health system to decrease clinician burnout. Military Medicine. 2021;186(Supplement_1):153-9. https://doi.org/10.1093/milmed/usaa225

81. Dukhanin V, Edrees HH, Connors CA, Kang E, Norvell M, Wu AW. Case: a second victim support program in pediatrics: successes and challenges to implementation. Journal of Pediatric Nursing. 2018;41:54-9. https://doi.org/10.1016/j.pedn.2018.01.011

82. Edrees H, Connors C, Paine L, Norvell M, Taylor H, Wu AW. Implementing the RISE second victim support programme at the Johns Hopkins Hospital: a case study. BMJ Open. 2016;6(9):e011708. https://doi.org/10.1136/bmjopen-2016-011708

83. Trent M, Waldo K, Wehbe-Janek H, Williams D, Hegefeld W, Havens L. Impact of health care adversity on providers: Lessons learned from a staff support program. Journal of Healthcare Risk Management. 2016;36(2):27-34. https://doi.org/10.1002/jhrm.21239

84. Schrøder K, Bovil T, Jørgensen JS, Abrahamsen C. Evaluation of'the Buddy Study', a peer support program for second victims in healthcare: A survey in two Danish hospital

For peer review only

departments. BMC Health Services Research. 2022;22(1):1-10. https://doi.org/10.1186/s12913-022-07973-9

85. Smith BW, Dalen J, Wiggins K, Tooley E, Christopher P, Bernard J. The brief resilience scale: assessing the ability to bounce back. International Journal of Behavioral Medicine. 2008;15:194-200. 10.1080/10705500802222972

86. Burlison JD, Scott SD, Browne EK, Thompson SG, Hoffman JM. The second victim experience and support tool (SVEST): validation of an organizational resource for assessing second victim effects and the quality of support resources. Journal of Patient Safety. 2017;13(2):93. 10.1097/PTS.0000000000000129

87. Riley MR, Mohr DC, Waddimba AC. The reliability and validity of three-item screening measures for burnout: Evidence from group-employed health care practitioners in upstate New York. Stress and Health. 2018;34(1):187-93. https://doi.org/10.1002/smi.2762

88. Judge TA, Scott BA, Ilies R. Hostility, job attitudes, and workplace deviance: test of a multilevel model. Journal of Applied Psychology. 2006;91(1):126-38. https://doi.org/10.1037/0021-9010.91.1.126

89. Sorra J, Nieva V. Hospital survey on patient safety culture. . Rockville, MD: Agency for Healthcare Research and Quality (US); 2004. Contract No.: 04-0041

90. Bisson JI, Olff M. Prevention and treatment of PTSD: the current evidence base. European Journal of Psychotraumatology. 2021;12(1):1824381. https://doi.org/10.1080/20008198.2020.1824381

91. Chong RIH, Yaow CYL, Chong NZ-Y, et al. Scoping review of the second victim syndrome among surgeons: understanding the impact, responses, and support systems. The American Journal of Surgery. 2024;229:5-14. https://doi.org/10.1016/j.amjsurg.2023.09.045

92. Hargrove MB, Quick JC, Nelson DL, Quick JD. The theory of preventive stress management: a 33-year review and evaluation. Stress and Health. 2011;27(3):182-93.

93. Tetrick LE, Winslow CJ. Workplace stress management interventions and health promotion. Annu Rev Organ Psychol Organ Behav. 2015;2(1):583-603. https://doi.org/10.1146/annurev-orgpsych-032414-111341

94. Kelloway EK, Barling J. Leadership development as an intervention in occupational health psychology. Work & Stress. 2010;24(3):260-79. https://doi.org/10.1080/02678373.2010.518441

For peer review only

For peer review only - http://bmjopen.bmj.com/site/about/guidelines.xhtml

95. Montano D, Hoven H, Siegrist J. Effects of organisational-level interventions at work on employees' health: a systematic review. BMC Public Health. 2014;14(1):1-9. https://doi.org/10.1186/1471-2458-14-135

96. Lamontagne AD, Keegel T, Louie AM, Ostry A, Landsbergis PA. A systematic review of the job-stress intervention evaluation literature, 1990–2005. International Journal of Occupational and Environmental Health. 2007;13(3):268-80. https://doi.org/10.1179/oeh.2007.13.3.268

97. Guerra-Paiva S, Lobão MJ, Simões DG, et al. Key factors for effective implementation of healthcare workers support interventions after patient safety incidents in health organisations: a scoping review. BMJ open. 2023;13(12):e078118.

98. Bajorek Z, Bevan S. Demonstrating the effectiveness of workplace counselling: Reviewing the evidence for wellbeing and cost-effectiveness outcomes. https://www.employment-studies.co.uk/system/files/resources/files/553.pdf. Institute for Employment Studies 2020. Report No.: 553

99. Mengstie MM. Perceived organizational justice and turnover intention among hospital healthcare workers. BMC Psychology. 2020;8:1-11. https://doi.org/10.1186/s40359-020-0387-8

100. Eisenberger R, Karagonlar G, Stinglhamber F, et al. Leader–member exchange and affective organizational commitment: The contribution of supervisor's organizational embodiment. Journal of Applied Psychology. 2010;95(6):1085-103. https://doi.org/10.1037/a0020858

101. Östergård K, Kuha S, Kanste O. Health-care leaders' and professionals' experiences and perceptions of compassionate leadership: A mixed-methods systematic review. Leadership in Health Services. 2023;37(5):49-65. https://doi.org/10.1108/LHS-06-2023-0043

102. de Zulueta P. How do we sustain compassionate healthcare? Compassionate leadership in the time of the COVID-19 pandemic. Clinics in Integrated Care. 2021;8:100071.

103. Klemm V, Rösner H, Krommer E, Strametz R. Organizational peer support for second victims in Vienna, Austria: The KoHi-project. European Journal of Public Health. 2024;34(Supplement_3):ckae144. 1859.

104. Krommer E, Klemm V, Trifunovic-Koenig M, et al. Peer Supporters' Experience of Supporting Second Victims (KoHi-III-Study). Preprints. 2024. https://doi.org/10.20944/preprints202405.1064.v1

For peer review only

For peer review only - http://bmjopen.bmj.com/site/about/guidelines.xhtml

105. Panagioti M, Khan K, Keers RN, et al. Prevalence, severity, and nature of preventable patient harm across medical care settings: systematic review and meta-analysis. BMJ. 2019;366:l4185. https://doi.org/10.1136/bmj.l4185

106. Donaldson MS, editor. An overview of to err is human: re-emphasizing the message of patient safety. Rockville (MD): Agency for Healthcare Reseach and Quality (US); 2011.

For peer review only

For peer review only - http://bmjopen.bmj.com/site/about/guidelines.xhtml

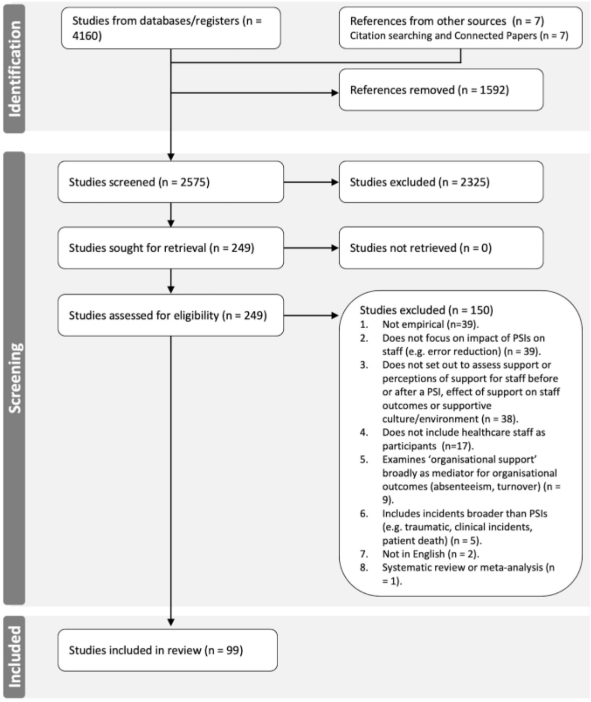

Figure 1: PRISMA flowchart of stages in the review

190x338mm (300 x 300 DPI)

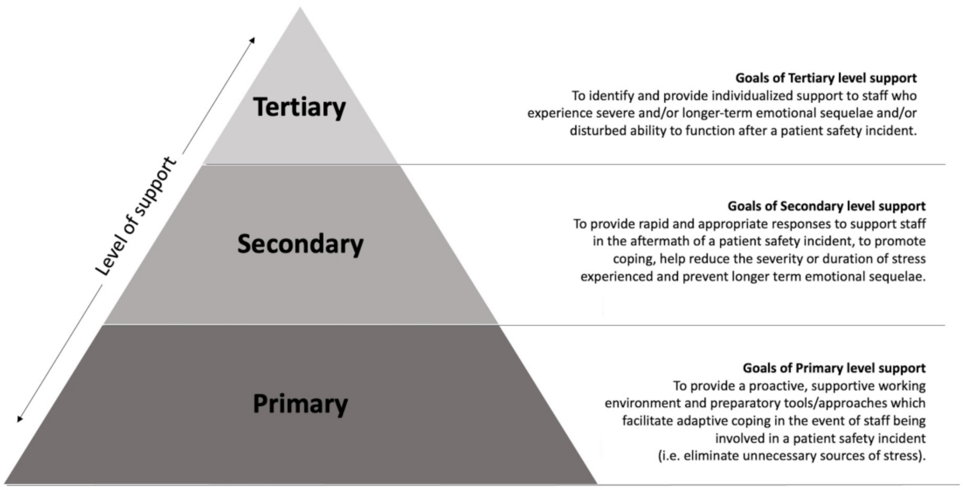

Figure 2: Organisational staff support model for Patient Safety Incidents

338x190mm (300 x 300 DPI)

PRISMA 2020 for Abstracts Checklist

Section and Topic	Item #	Checklist item	Reported (Yes/No)
**TITLE**			
Title	1	Identify the report as a systematic review.	Yes
**BACKGROUND**			
Objectives	2	Provide an explicit statement of the main objective(s) or question(s) the review addresses.	Yes
**METHODS**			
Eligibility criteria	3	Specify the inclusion and exclusion criteria for the review.	Yes
Information sources	4	Specify the information sources (e.g. databases, registers) used to identify studies and the date when each was last searched.	Yes
Risk of bias	5	Specify the methods used to assess risk of bias in the included studies.	Yes
Synthesis of results	6	Specify the methods used to present and synthesise results.	Yes
**RESULTS**			
Included studies	7	Give the total number of included studies and participants and summarise relevant characteristics of studies.	Yes
Synthesis of results	8	Present results for main outcomes, preferably indicating the number of included studies and participants for each. If meta-analysis was done, report the summary estimate and confidence/credible interval. If comparing groups, indicate the direction of the effect (i.e. which group is favoured).	Yes
**DISCUSSION**			
Limitations of evidence	9	Provide a brief summary of the limitations of the evidence included in the review (e.g. study risk of bias, inconsistency and imprecision).	Yes
Interpretation	10	Provide a general interpretation of the results and important implications.	Yes
**OTHER**			
Funding	11	Specify the primary source of funding for the review.	Yes
Registration	12	Provide the register name and registration number.	Yes

From: Page MJ, McKenzie JE, Bossuyt PM, Boutron I, Hoffmann TC, Mulrow CD, et al. The PRISMA 2020 statement: an updated guideline for reporting systematic reviews. BMJ 2021;372:n71. doi: 10.1136/bmj.n71

For more information, visit: http://www.prisma-statement.org/

For peer review only

Supplementary 1 File: Completed PRISMA 2020 Checklist

Section and Topic	Item #	Checklist item	Location where item is reported
**TITLE**			
Title	1	Identify the report as a systematic review.	1
**ABSTRACT**			
Abstract	2	See the PRISMA 2020 for Abstracts checklist.	Used this
**INTRODUCTION**			
Rationale	3	Describe the rationale for the review in the context of existing knowledge.	5-6
Objectives	4	Provide an explicit statement of the objective(s) or question(s) the review addresses.	6-7
**METHODS**			
Eligibility criteria	5	Specify the inclusion and exclusion criteria for the review and how studies were grouped for the syntheses.	7, 9-10
Information sources	6	Specify all databases, registers, websites, organisations, reference lists and other sources searched or consulted to identify studies. Specify the date when each source was last searched or consulted.	7-8
Search strategy	7	Present the full search strategies for all databases, registers and websites, including any filters and limits used.	7 (S2_File)
Selection process	8	Specify the methods used to decide whether a study met the inclusion criteria of the review, including how many reviewers screened each record and each report retrieved, whether they worked independently, and if applicable, details of automation tools used in the process.	8-9
Data collection process	9	Specify the methods used to collect data from reports, including how many reviewers collected data from each report, whether they worked independently, any processes for obtaining or confirming data from study investigators, and if applicable, details of automation tools used in the process.	8-9
Data items	10a	List and define all outcomes for which data were sought. Specify whether all results that were compatible with each outcome domain in each study were sought (e.g. for all measures, time points, analyses), and if not, the methods used to decide which results to collect.	8
	10b	List and define all other variables for which data were sought (e.g. participant and intervention characteristics, funding sources). Describe any assumptions made about any missing or unclear information.	8-9
Study risk of bias assessment	11	Specify the methods used to assess risk of bias in the included studies, including details of the tool(s) used, how many reviewers assessed each study and whether they worked independently, and if applicable, details of automation tools used in the process.	9
Effect measures	12	Specify for each outcome the effect measure(s) (e.g. risk ratio, mean difference) used in the synthesis or presentation of results.	N/A
Synthesis methods	13a	Describe the processes used to decide which studies were eligible for each synthesis (e.g. tabulating the study intervention characteristics and comparing against the planned groups for each synthesis (item #5)).	9-10
	13b	Describe any methods required to prepare the data for presentation or synthesis, such as handling of missing summary statistics, or data conversions.	N/A
	13c	Describe any methods used to tabulate or visually display results of individual studies and syntheses.	10
	13d	Describe any methods used to synthesize results and provide a rationale for the choice(s). If meta-analysis was performed, describe the model(s), method(s) to identify the presence and extent of statistical heterogeneity, and software package(s) used.	9-10
	13e	Describe any methods used to explore possible causes of heterogeneity among study results (e.g. subgroup analysis, meta-regression).	N/A
	13f	Describe any sensitivity analyses conducted to assess robustness of the synthesized results.	N/A
Reporting bias assessment	14	Describe any methods used to assess risk of bias due to missing results in a synthesis (arising from reporting biases).	N/A
Certainty assessment	15	Describe any methods used to assess certainty (or confidence) in the body of evidence for an outcome.	N/A

For peer review only - http://bmjopen.bmj.com/site/about/guidelines.xhtml

PRISMA 2020 Checklist

Section and Topic	Item #	Checklist item	Location where item is reported
**RESULTS**			
Study selection	16a	Describe the results of the search and selection process, from the number of records identified in the search to the number of studies included in the review, ideally using a flow diagram.	10
	16b	Cite studies that might appear to meet the inclusion criteria, but which were excluded, and explain why they were excluded.	10-11
Study characteristics	17	Cite each included study and present its characteristics.	10-11; 17; S3 File
Risk of bias in studies	18	Present assessments of risk of bias for each included study.	19-20
Results of individual studies	19	For all outcomes, present, for each study: (a) summary statistics for each group (where appropriate) and (b) an effect estimate and its precision (e.g. confidence/credible interval), ideally using structured tables or plots.	12-13, 17, S3 File, S1 Table 4
Results of syntheses	20a	For each synthesis, briefly summarise the characteristics and risk of bias among contributing studies.	19
	20b	Present results of all statistical syntheses conducted. If meta-analysis was done, present for each the summary estimate and its precision (e.g. confidence/credible interval) and measures of statistical heterogeneity. If comparing groups, describe the direction of the effect.	N/A
	20c	Present results of all investigations of possible causes of heterogeneity among study results.	19
	20d	Present results of all sensitivity analyses conducted to assess the robustness of the synthesized results.	N/A
Reporting biases	21	Present assessments of risk of bias due to missing results (arising from reporting biases) for each synthesis assessed.	N/A
Certainty of evidence	22	Present assessments of certainty (or confidence) in the body of evidence for each outcome assessed.	N/A
**DISCUSSION**			
Discussion	23a	Provide a general interpretation of the results in the context of other evidence.	23-25
	23b	Discuss any limitations of the evidence included in the review.	23
	23c	Discuss any limitations of the review processes used.	26
	23d	Discuss implications of the results for practice, policy, and future research.	26
**OTHER INFORMATION**			
Registration and protocol	24a	Provide registration information for the review, including register name and registration number, or state that the review was not registered.	3, 7
	24b	Indicate where the review protocol can be accessed, or state that a protocol was not prepared.	3, 7
	24c	Describe and explain any amendments to information provided at registration or in the protocol.	8
Support	25	Describe sources of financial or non-financial support for the review, and the role of the funders or sponsors in the review.	27
Competing interests	26	Declare any competing interests of review authors.	27
Availability of data, code and other materials	27	Report which of the following are publicly available and where they can be found: template data collection forms; data extracted from included studies; data used for all analyses; analytic code; any other materials used in the review.	Detailed narrative data synthesis - S3 File

From: Page MJ, McKenzie JE, Bossuyt PM, Boutron I, Hoffmann TC, Mulrow CD, et al. The PRISMA 2020 statement: an updated guideline for reporting systematic reviews. BMJ 2021;372:n71. doi: 10.1136/bmj.n71

For more information, visit: http://www.prisma-statement.org/

For peer review only - http://bmjopen.bmj.com/site/about/guidelines.xhtml

Supplementary File 2: Search criteria

Medline

1 ((critical or patient safety or harmful or clinical) adj incident*).mp.
2 ((avoidable or preventable or unsafe or undesirable) adj1 (event* or outcome* or complication* or death or accident* or injur*)).mp.
3 (serious incident? or critical incident? or never event? or near miss* or sentinel event? or adverse patient occurrence? or serious reportable event? or (serious adj2 event? adj3 (clinical or critical or medical or surgical)) or preventable adverse event? or avoidable adverse event? or ((Medical or surgical) adj2 Adverse adj2 Event?)).mp.
4 1 or 2 or 3 or exp medical errors/
5 ((Health adj2 professional*) or (health adj2 provider*)).mp. or exp health personnel/ or healthcare profession*.mp. or (healthcare adj2 provider*).mp. or healthcare worker*.mp. or health care worker*.mp. or (worker* adj2 (health or healthcare)).mp. or nurs*.ti,ab. or physician*.ti,ab. or surgeon.ti,ab. or midwive*.ti,ab. or clinician*.ti,ab.
6 (4 and 5) or second victim*.af.
7 exp social support/
8 exp Occupational Health Services/
9 Crisis intervention/
10 Counseling/
11 Stress, Psychological/pc, th or Stress Disorders, Post-Traumatic/pc, th
12 support*.ti,kw,kf.
13 ("critical incident stress management" or "critical incident stress reporting system*" or "critical incident stress debrief*").mp.
14 ("Second Victim Experience and Support Tool" or SVEST).mp.
15 (peer group* or (peer* adj3 support*)).af.
16 Debrief*.mp.
17 (support* adj2 (second victim* or organi?ation* or service* or system* or program* or resource* or social)).mp.
18 ((Resilienc* adj2 training) or (resilienc* adj2 intervention*) or (promot* adj2 resilienc*)).mp.
19 "Schwartz Center Rounds".ti,ab,kf,kw.
20 Resilience, psychological/
21 ("Intervention training" or (crisis adj2 training)).mp.
22 "Resilience In Stressful Events".mp.
25 ((Institution* adj2 support) or (Institution* adj2 response*) or (Institution* adj2 respond*)).mp.
26 Simulation Training/
27 (safe* adj2 culture*).mp.
28 psychologic* safe*.mp.
29 just culture*.mp.
30 non-punitive.mp.
31 *organizational culture/
32 organizational culture/ and (px or ed or pc or mt or og).fs.
23 6 and 7
24 6 and 8
33 6 and 9
34 6 and 10
35 6 and 11
36 6 and 12
37 6 and 13
38 6 and 14
39 6 and 15
40 6 and 16
41 6 and 17

For peer review only - http://bmjopen.bmj.com/site/about/guidelines.xhtml

44 6 and 18
45 6 and 19
46 6 and 20
47 6 and 21
48 6 and 22
49 6 and 23
50 6 and 24
51 6 and 25
52 6 and 26
53 6 and 27
54 6 and 28
55 6 and 29
56 6 and 30
57 6 and 31
58 6 and 32
59 or/33-58
60 limit 59 to (abstracts and english language and yr="2010 -Current")

For peer review only

PubMed

Search	Query
#62	Search: **#45 OR #46 OR #47 OR #48 OR #49 OR #50 OR #51 OR #52 OR #53 OR #54 OR #55 OR #56 OR #57** Filters: **from 2021 - 2022**
#61	Search: **#34 OR #35 OR #36 OR #37 OR #38 OR #39 OR #40 OR #41 OR #42 OR #43 OR #44** Filters: **from 2021 - 2022**
#57	Search: **#6 AND #33**
#56	Search: **#6 AND #32**
#55	Search: **#6 AND #31**
#54	Search: **#6 AND #30**
#53	Search: **#6 AND #29**
#52	Search: **#6 AND #28**
#51	Search: **#6 AND #27**
#50	Search: **#6 AND #26**
#49	Search: **#6 AND #25**
#48	Search: **#6 AND #23**
#47	Search: **#6 AND #22**
#46	Search: **#6 AND #21**
#45	Search: **#6 AND #20**
#44	Search: **#6 AND #19**
#43	Search: **#6 AND #18**
#42	Search: **#6 AND #17**
#41	Search: **#6 AND #16**
#40	Search: **#6 AND #15**
#39	Search: **#6 AND #14**
#38	Search: **#6 AND #13**

Search	Query
#37	Search: **#6 AND #12**
#36	Search: **#6 AND #11**
#35	Search: **#6 AND #10**
#34	Search: **#6 AND #9**
#33	Search: **"organizational culture"[Mesh] AND (px[sh] OR ed[sh] OR pc[sh] OR mt[sh] OR og[sh])**
#32	Search: **organizational culture[MeSH Major Topic]**
#31	Search: **"non-punitive"[tw] or "non punitive"[tw]**
#30	Search: **"just culture"[tw] or "just cultures"[tw]**
#29	Search: **"psychological safe"[tw] or "psychological safety"[tw]**
#28	Search: **"safe culture"[tw] or "safety culture"[tw]**
#27	Search: **simulation training[mesh]**
#26	Search: **"Institution support"[all] OR "institutional supports"[all] OR "Institution response"[all] OR [all] OR "institutional responses"[all] OR "institutional response"[all] OR "institution responding"[all] OR "institutional responding"[all] OR "institutions responding"[all] OR "institution responding"[all]**
#25	Search: **#6 and #8**
#23	Search: **"Resilience In Stressful Events"[tw] - Schema: all**
#22	Search: **#6 and #7**
#21	Search: **("debrief"[tw] OR "debriefs"[tw] OR "debriefings"[tw])**
#20	Search: **("Intervention training"[tw] OR ("crisis training"[tw])**
#19	Search: **resilience, psychological[MESH]**
#18	Search: **("Schwartz Center Rounds"[tiab] OR "Schwartz Center Rounds"[ot] OR "Schwartz Center Rounds"[ot])**
#17	Search: **(Resilience[tw] AND training"[tw] OR training[tw] AND resiliency[tw] OR resilience[tw] AND intervention[tw] OR resiliency[tw] AND intervention[tw] OR resilience[tw] AND interventions[tw] OR resiliency[tw] AND interventions[tw] OR promotion[tw] AND resilience[tw] OR promotion"[tw] AND "resiliency"[tw] OR "promoting"[tw] AND "resilience"[tw] OR "promoting"[tw] AND "resiliency"[tw])**

For peer review only - http://bmjopen.bmj.com/site/about/guidelines.xhtml

Search	Query
#16	Search: **(("support second victim"[tw] OR "support second victims"[tw] OR "support organisation"[tw] OR "support organisations"[tw] OR "support organization"[tw] OR "support organizations"[tw] OR "support service"[tw] OR "support services"[tw] OR "support system"[tw] OR "support systems "[tw] OR "support program"[tw] OR "support programs"[tw] OR "support programme"[tw] OR "support programmes"[tw] OR "support resource "[tw] OR "support resources"[tw] OR "social support"[tw]))**
#15	Search: **("peer group"[all] OR "peer groups"[all] OR "peer support"[all] OR "support peers"[all]))**
#14	Search: **("Second Victim Experience AND Support Tool"[tw] OR "SVEST"[tw])**
#13	Search: **"critical incident stress management"[all] OR "critical incident stress reporting system"[all] OR "critical incident stress reporting systems"[all] OR "critical incident stress debrief"[all] OR "critical incident stress debriefs"[all] OR "critical incident stress debriefing"[all] OR "critical incident stress debriefings"[all]**
#12	Search: **"support"[ti]**
#11	Search: **("Stress, Psychological/Prevention and Control"[mesh] OR "Stress, Psychological/Therapy"[mesh]) OR ("Stress Disorders, Post-Traumatic/Prevention and Control"[mesh] OR "Stress Disorders, Post-Traumatic/Therapy"[mesh])**
#10	Search: **"counseling"[mesh]**
#9	Search: **"crisis intervention"[mesh]**
#8	Search: **"occupational health services"[mesh]**
#7	Search: **"social support"[mesh]**
#6	Search: **(#4 AND #5) or "second victim" or "second victims"**
#5	Search: **("Health professional"[tw] OR "Health professionals"[tw] OR "healthcare professional"[tw] OR "healthcare professionals"[tw] OR "health provider"[tw] OR "healthcare provider"[tw] OR "health provider"[tw] OR "health providers"[tw] OR "healthcare provider"[tw] OR "healthcare providers"[tw] OR "health personnel"[MESH] or "health personnel"[tw] OR "healthcare profession"[tw] OR "healthcare professions"[tw] OR "healthcare worker."[tw] OR "healthcare workers"[tw] OR "health care worker"[tw] OR "health care workers"[tw] OR "health worker"[tw] OR "health workers"[tw] OR nurse[tw] OR nurses[tw] OR physician[tw] OR physicians[tw] OR surgeon[tw] OR surgeons[tw] OR midwives[tw] OR midwife[tw] OR clinician[tw] OR clinicians[tw])**
#4	Search: **#1 or #2 or #3 or "medical errors"[MESH]**
#3	Search: **("serious incident"[tw] OR "serious incidents"[tw] OR "critical incident"[tw] OR "critical incidents"[tw] OR "never event"[tw] OR "never**

For peer review only - http://bmjopen.bmj.com/site/about/guidelines.xhtml

Search	Query
	events"[tw] OR "near miss"[tw] OR "near misses"[tw] OR "sentinel event"[tw] OR "sentinel events"[tw] OR "adverse patient occurrence"[tw] OR "adverse patient occurrences"[tw] OR "serious reportable event"[tw] OR "serious reportable events"[tw] OR (("serious clinical event"[tw] OR "serious clinical events"[tw] OR "serious critical event"[tw] OR "serious critical events"[tw] OR "serious medical event"[tw] OR "serious medical events"[tw] OR "serious surgical event"[tw] OR "serious surgical events"[tw] OR "preventable adverse event"[tw] OR "preventable adverse events"[tw] OR "avoidable adverse event"[tw] OR "avoidable adverse events "[tw] OR "medical adverse event"[tw] OR "medical adverse events"[tw] OR "surgical adverse event"[tw] OR "surgical adverse events"[tw])
#2	Search: ("avoidable event"[tw] OR " avoidable events"[tw] OR "avoidable outcome"[tw] OR "avoidable outcomes"[tw] OR "avoidable complication"[tw] OR "avoidable complications"[tw] OR " avoidable death"[tw] OR "avoidable accident"[tw] OR "avoidable accidents"[tw] OR "avoidable injury"[tw] OR "avoidable injuries "[tw] OR "preventable event"[tw] OR " preventable events"[tw] OR "preventable outcome"[tw] OR "preventable outcomes"[tw] OR "preventable complication"[tw] OR "preventable complications"[tw] OR " preventable death"[tw] OR "preventable accident"[tw] OR "preventable accidents"[tw] OR "preventable injury"[tw] OR "preventable injuries"[tw] OR "unsafe event"[tw] OR " unsafe events"[tw] OR "unsafe outcome"[tw] OR "unsafe outcomes"[tw] OR "unsafe complication"[tw] OR "unsafe complications"[tw] OR " unsafe death"[tw] OR "unsafe accident"[tw] OR "unsafe accidents"[tw] OR "unsafe injury"[tw] OR "unsafe injuries"[tw] OR "undesireable event"[tw] OR " undesireable events"[tw] OR "undesireable outcome"[tw] OR "undesireable outcomes"[tw] OR "undesireable complication"[tw] OR "undesireable complications"[tw] OR " undesireable death"[tw] OR "undesireable accident"[tw] OR "undesireable accidents"[tw] OR "undesireable injury"[tw] OR "undesireable injuries"[tw])
#1	Search: (("critical incident"[tw] OR "critical incidents"[tw] OR "patient safety incident"[tw] OR "patient safety incidents"[tw] OR "harmful incident"[tw] OR "harmful incidents"[tw] OR "clinical incident"[tw] OR "clinical incidents"[tw]))

For peer review only - http://bmjopen.bmj.com/site/about/guidelines.xhtml

Scopus

((ALL ("second victim*")) OR ((((TITLE-ABS-KEY (((critical OR "patient safety" OR harmful OR clinical) W/1 incident*))) OR (TITLE-ABS-KEY (((avoidable OR preventable OR unsafe OR undesirable) W/1 (event* OR out come* OR complication* OR death OR accident* OR injur*)))) OR (TITLE-ABS-KEY (("serious incident*" OR "critical incident*" OR "never event*" OR "near miss*" OR "sentinel event*" OR "adverse patient occurrence*" OR "serious reportable event*" OR (serious W/2 event* W/3 (clinical OR critical OR medical OR surgical)) OR "preventable adverse event*" OR "avoidable adverse event*" OR ((medical OR surgical) W/2 adverse W/2 event*))))) OR (INDEXTERMS ("medical errors"))) AND (TITLE-ABS-KEY ((health W/2 professional*) OR (health W/2 provider*)) OR INDEXTERMS ("health personnel") OR TITLE-ABS-KEY ("healthcare profession*") OR TITLE-ABS-KEY (healthcare W/2 provider*) OR TITLE-ABS-KEY ("healthcare worker*") OR TITLE-ABS-KEY ("health care worker*") OR TITLE-ABS-KEY (worker* W/2 (health OR healthcare)) OR TITLE-ABS (nurs*) OR TITLE-ABS (physician*) OR TITLE-ABS (surgeon) OR TITLE-ABS (midwive*) OR TITLE-ABS ("clinician*")))) AND ((INDEXTERMS ("Crisis intervention")) OR (INDEXTERMS ("counseling" OR "counselling")) OR (INDEXTERMS ("Stress Disorders, Post-Traumatic")) OR (INDEXTERMS ("Stress, psychological")) OR (TITLE ("support*")) OR (TITLE-ABS-KEY ("critical incident stress management" OR "critical incident stress reporting system*" OR "critical incident stress debrief*")) OR (TITLE-ABS-KEY ("Second Victim Experience and Support Tool" OR svest)) OR (ALL ("peer group*" OR (peer* W/3 support*))) OR (TITLE-ABS-KEY ("Debrief*")) OR (TITLE-ABS-KEY (support* W/2 ("second victim*" OR organi*ation* OR service* OR system* OR program* OR resource* OR social))) OR (TITLE-ABS-KEY ((resilienc* W/2 training) OR (resilienc* W/2 intervention*) OR (promot* W/2 resilienc*))) OR (TITLE-ABS-KEY ("Schwartz Center Rounds")) OR (INDEXTERMS ("Resilience, psychological")) OR (TITLE-ABS-KEY ("Intervention training" OR (crisis W/2 training))) OR (((ALL ("second victim*")) OR ((((TITLE-ABS-KEY (((critical OR "patient safety" OR harmful OR clinical) W/1 incident*))) OR (TITLE-ABS-KEY (((avoidable OR preventable OR unsafe OR undesirable) W/1 (event* OR out come* OR complication* OR death OR accident* OR injur*)))) OR (TITLE-ABS-KEY (("serious incident*" OR "critical incident*" OR "never event*" OR "near miss*" OR "sentinel event*" OR "adverse patient occurrence*" OR "serious reportable event*" OR (serious W/2 event* W/3 (clinical OR critical OR medical OR surgical)) OR "preventable adverse event*" OR "avoidable adverse event*" OR ((medical OR surgical) W/2 adverse W/2 event*))))) OR (INDEXTERMS ("medical errors"))) AND (TITLE-ABS-KEY ((health W/2 professional*) OR (health W/2 provider*)) OR INDEXTERMS ("health personnel") OR TITLE-ABS-KEY ("healthcare profession*") OR TITLE-ABS-KEY (healthcare W/2 provider*) OR TITLE-ABS-KEY ("healthcare

worker*") OR TITLE-ABS-KEY ("health care worker*") OR TITLE-ABS-KEY (worker* W/2 (health OR healthcare)) OR TITLE-ABS (nurs*) OR TITLE-ABS (physician*) OR TITLE-ABS (surgeon) OR TITLE-ABS (midwive*) OR TITLE-ABS ("clinician*")))) AND (INDEXTERMS ("social support"))) OR (TITLE-ABS-KEY ("Resilience In Stressful Events")) OR (((ALL ("second victim*")) OR ((((TITLE-ABS-KEY (((critical OR "patient safety" OR harmful OR clinical) W/1 incident*))) OR (TITLE-ABS-KEY (((avoidable OR preventable OR unsafe OR undesirable) W/1 (event* OR out come* OR complication* OR death OR accident* OR injur*)))) OR (TITLE-ABS-KEY (("serious incident*" OR "critical incident*" OR "never event*" OR "near miss*" OR "sentinel event*" OR "adverse patient occurrence*" OR "serious reportable event*" OR (serious W/2 event* W/3 (clinical OR critical OR medical OR surgical)) OR "preventable adverse event*" OR "avoidable adverse event*" OR ((medical OR surgical) W/2 adverse W/2 event*))))) OR (INDEXTERMS ("medical errors"))) AND (TITLE-ABS-KEY ((health W/2 professional*) OR (health W/2 provider*)) OR INDEXTERMS ("health personnel") OR TITLE-ABS-KEY ("healthcare profession*") OR TITLE-ABS-KEY (healthcare W/2 provider*) OR TITLE-ABS-KEY ("healthcare worker*") OR TITLE-ABS-KEY ("health care worker*") OR TITLE-ABS-KEY (worker* W/2 (health OR healthcare)) OR TITLE-ABS (nurs*) OR TITLE-ABS (physician*) OR TITLE-ABS (surgeon) OR TITLE-ABS (midwive*) OR TITLE-ABS ("clinician*")))) AND (INDEXTERMS ("Occupational Health Services"))) OR (INDEXTERMS ("Simulation Training")) OR (TITLE-ABS-KEY (safe* W/2 culture*)) OR (TITLE-ABS-KEY ("psychologic* safe*")) OR (TITLE-ABS-KEY ("just culture*")) OR (TITLE-ABS-KEY ((institution* W/2 support) OR (institution* W/2 response*) OR (institution* W/2 respond*))) OR (TITLE-ABS-KEY ("non-punitive")) OR (INDEXTERMS ("organizational culture"))) AND (LIMIT-TO (PUBYEAR , 2022) OR LIMIT-TO (PUBYEAR , 2021)) AND (LIMIT-TO (LANGUAGE , "English")) View less

For peer review only

Web of Science

Database: Web of Science Core Collection

Entitlements:

- WOS.SCI: 1900 to 2022
- WOS.AHCI: 1975 to 2022
- WOS.ESCI: 2015 to 2022
- WOS.ISTP: 1990 to 2022
- WOS.SSCI: 1900 to 2022
- WOS.ISSHP: 1990 to 2022

Searches:

Search: #32 AND #6 and 2021 or 2022 (Publication Years)

Search: #32 AND #6

Search: #9 OR #10 OR #11 OR #12 OR #13 OR #14 OR #15 OR #16 OR #17 OR #18 OR #19 OR #20 OR #21 OR #22 OR #23 OR #24 OR #25 OR #26 OR #27 OR #28 OR #29 OR #30 OR #31 Results: 979472

#31. Search: TS=("organi$ational culture")

#30. Search: "non-punitive" (Topic)

#29. Search: "just culture*" (Topic)

#28. Search: "psychologic* safe*" (Topic)

#27. Search: TS=(safe* NEAR/2 culture*)

#26. Search: TS=("simulation training")

#25. Search: TS=((Institution* NEAR/2 support) OR (Institution* NEAR/2 response*) OR (Institution* NEAR/2 respond*))

#24. Search: #6 AND #8

#23. Search: TS=("Resilience in Stressful Events")

#22. Search: #7 AND #6

#21. Search: "Intervention training" OR crisis NEAR/2 training (Topic)

#20. Search: TS=("psychological resilience")

#19. Search: TS="Schwartz Center Rounds"

For peer review only

#18. Search: (TS=((Resilienc* NEAR/2 training) OR (resilienc* NEAR/2 intervention*) OR (promot* NEAR/2 resilienc*)))

#17. Search: TS=(((support* NEAR/2 ("second victim*" OR organi$ation* OR service* OR system* OR program* OR resource* OR social))))

#16. Search: Debrief* (Topic)

#15. Search: ("peer group*" OR (peer* NEAR/3 support*)) (Topic)

#14. Search: "Second Victim Experience and Support Tool" OR SVEST (Topic)

#13. Search: "critical incident stress management" OR "critical incident stress reporting system*" OR "critical incident stress debrief*" (Topic)

#12. Search: "support*" (Title) OR "support*" (Author Keywords)

#11. Search: TS=(("psychological stress" or "post traumatic stress disorder*" or PTSD) AND (therap* or prevention))

#10. Search: TS=(counselling or counseling)

#9. Search: TS=("crisis intervention"*)

#8. Search: TS=("Occupational Health Services")

#7. Search: TS=("social support")

#6. Search: #5 AND #4 OR ALL=("second victim*")

#5. Search: TS=(((Health NEAR/2 professional*) OR (health NEAR/2 provider*)) OR "health

personnel" OR "healthcare profession*" OR (healthcare NEAR/2 provider*) OR "healthcare worker*" OR "health care worker*" OR (worker* NEAR/2 (health OR healthcare)) OR nurs* OR
physician* OR surgeon* OR midwife or midwives or mid OR "clinician*")

#4. Search: #3 OR #2 OR #1 OR TS=("medical errors")

#3. Search: ("serious incident$" OR "critical incident$" OR "never event$" OR "near miss*" OR "sentinel event$" OR "adverse patient occurrence$" OR "serious reportable event$" OR (serious NEAR/2 event$ NEAR/3 (clinical OR critical OR medical OR surgical)) OR "preventable adverse event$" OR "avoidable adverse event$" OR ((Medical OR surgical) NEAR/2 Adverse NEAR/2 Event$)) (Topic)

#2. Search: ((avoidable OR preventable OR unsafe OR undesirable) NEAR/1 (event* OR outcome* OR complication* OR death OR accident* OR injur*)) (Topic)

For peer review only - http://bmjopen.bmj.com/site/about/guidelines.xhtml

#1. Search: ((critical OR "patient safety" OR harmful OR clinical) NEAR/0 incident*) (Topic)

For peer review only

For peer review only - http://bmjopen.bmj.com/site/about/guidelines.xhtml

Supplementary File 3: Synthesis Without Meta-analysis (SWiM) reporting items

The citation for the Synthesis Without Meta-analysis explanation and elaboration article is: Campbell M, McKenzie JE, Sowden A, Katikireddi SV, Brennan SE, Ellis S, Hartmann-Boyce J, Ryan R, Shepperd S, Thomas J, Welch V, Thomson H. Synthesis without meta-analysis (SWiM) in systematic reviews: reporting guideline BMJ 2020;368:l6890 http://dx.doi.org/10.1136/bmj.l6890

**SWiM is intended to complement and be used as an extension to PRISMA**			
**SWiM reporting item**	**Item description**	**Page in manuscript where item is reported**	**Other***
Methods			
**1** Grouping studies for synthesis	1a) Provide a description of, and rationale for, the groups used in the synthesis (e.g., groupings of populations, interventions, outcomes, study design)	Pages 9, 10	International prospective register of systematic reviews - PROSPERO registration number: CRD42022325796 https://www.crd.york.ac.uk/PROSPERO/display_record.php?RecordID=325796]
	1b) Detail and provide rationale for any changes made subsequent to the protocol in the groups used in the synthesis	Page 9, 10	
**2** Describe the standardised metric and transformation methods used	Describe the standardised metric for each outcome. Explain why the metric(s) was chosen, and describe any methods used to transform the intervention effects, as reported in the study, to the standardised metric, citing any methodological guidance consulted	Pages 10, 11	International prospective register of systematic reviews - PROSPERO registration number: CRD42022325796 https://www.crd.york.ac.uk/PROSPERO/display_record.php?RecordID=325796]
**3** Describe the synthesis methods	Describe and justify the methods used to synthesise the effects for each outcome when it was not possible to undertake a meta-analysis of effect estimates	Pages 9, 10, 11	International prospective register of systematic reviews - PROSPERO registration number: CRD42022325796 https://www.crd.york.ac.uk/PROSPERO/display_record.php?RecordID=325796]
**4** Criteria used to prioritise results	Where applicable, provide the criteria used, with supporting justification, to select the particular studies, or a particular	Pages 9, 10, 11	International prospective register of systematic reviews - PROSPERO registration number: CRD42022325796 https://www.crd.york.ac.uk/PROSPERO/display_record.php?RecordID=325796]

For peer review only

Supplementary File 3: Synthesis Without Meta-analysis (SWiM) reporting items

for summary and synthesis	study, for the main synthesis or to draw conclusions from the synthesis (e.g., based on study design, risk of bias assessments, directness in relation to the review question)		
**5** Investigation of heterogeneity in reported effects	State the method(s) used to examine heterogeneity in reported effects when it was not possible to undertake a meta-analysis of effect estimates and its extensions to investigate heterogeneity	Page 11	International prospective register of systematic reviews - PROSPERO registration number: CRD42022325796 https://www.crd.york.ac.uk/PROSPERO/display_record.php?RecordID=325796]
**6** Certainty of evidence	Describe the methods used to assess certainty of the synthesis findings	Page 11	International prospective register of systematic reviews - PROSPERO registration number: CRD42022325796 https://www.crd.york.ac.uk/PROSPERO/display_record.php?RecordID=325796]
**7** Data presentation methods	Describe the graphical and tabular methods used to present the effects (e.g., tables, forest plots, harvest plots).<br><br>Specify key study characteristics (e.g., study design, risk of bias) used to order the studies, in the text and any tables or graphs, clearly referencing the studies included	Page 12, 17	
Results			
**8** Reporting results	For each comparison and outcome, provide a description of the synthesised findings, and the certainty of the findings. Describe the result in language that is consistent with the question the synthesis addresses, and indicate which studies contribute to the synthesis	Stage 1: Pages 13-17.<br><br>Stage 2: Pages 18-29.	Full detail provided in Supplementary Files 4 and 5 linked to the paper.
Discussion			

For peer review only - http://bmjopen.bmj.com/site/about/guidelines.xhtml

For peer review only

Supplementary File 3: Synthesis Without Meta-analysis (SWiM) reporting items

**9** Limitations of the synthesis	Report the limitations of the synthesis methods used and/or the groupings used in the synthesis, and how these affect the conclusions that can be drawn in relation to the original review question	Page 34, 35	

PRISMA=Preferred Reporting Items for Systematic Reviews and Meta-Analyses.
*If the information is not provided in the systematic review, give details of where this information is available (e.g., protocol, other published papers (provide citation details), or website (provide the URL)).

For peer review only

For peer review only - http://bmjopen.bmj.com/site/about/guidelines.xhtml

Supplementary 4 File: Narrative summary findings from surveys and qualitative studies included at Stage 1 (n=72)

1. What support do staff want for coping with Patient Safety Incidents (RQ1)?

1.1 Surveys (n=49; papers 1-49)

Thirty-four survey studies explored the support that staff would want following an adverse event to help second victims recover (1, 3, 4, 5, 7, 8, 9, 10, 12, 14, 15, 16, 17, 18, 19, 20, 24, 25, 26, 27, 29, 31, 32, 34, 35, 36, 37, 38, 41, 42, 44, 45, 47, 48).

Peer support was an aspect that arose in many survey studies (n = 23) and was rated as the most desired support strategy by every study that ranked supports (1, 3, 4, 10, 15, 16, 18, 19, 27, 32, 38, 41, 44, 45, 46, 47). Of the survey studies that gave figures, between 59% and 92.1% of the samples would want peer support following an event (4, 15, 18, 19, 27, 41, 44, 45, 47). However, the term 'peer support' was used differently between studies. It was primarily referred to as 'a respected peer to discuss the details of what happened' in 9 of the 23 studies (4, 16, 28, 33, 38, 39, 45, 46, 47); other definitions included emotional support (8, 9), conversations with peers (10, 19, 20), confidential support (17), talking to someone (32), discussing emotional issues (36, 37) or support from close people on a professional or personal level (42, 43). There were differences in who second victims wanted to receive peer support from, with some preferring conversations with direct colleagues (1, 3, 8, 41, 42) and others desiring to speak with the whole team (18, 31, 41) or other physicians (3). One survey study found that informal emotional support was one of the least wanted methods of support despite being one of the most utilised (7).

There were a variety of forms of support provided by organisations for second victims that were referenced across the survey studies. Of the survey studies that ranked strategies, the support that was rated in the top 3 by the second most studies was having 'a specified peaceful location that is available to recover and recompose' (4, 9, 27, 32, 44, 45, 47), with between 61.4% and 78.8% of samples wanting this support. Debriefs with the colleagues involved in the incident were wanted by some participants (7, 14, 16, 18, 35, 36, 48), although it was not always clear how this was different to peer support. Multiple survey studies found that second victims wanted information about the incident, including ethical issues (7, 35), causes and preventative measures (7, 26, 29, 34, 35, 36, 41). Several participants were found to desire crisis interventions (7, 16, 35, 36),

For peer review only

including guidance and advice regarding legal proceedings following the event (7, 16, 26, 34, 36).

Counselling was desired as a method of support for second victims in thirteen survey studies (1, 7, 14, 15, 16, 17, 19, 25, 27, 34, 35, 36, 37). Of the survey studies that ranked support strategies, counselling was rated in the top 3 by the joint third most studies (1, 16, 19, 25, 37, 38), with between 29.9% and 96.1% of samples desiring this support. However, one sample found it to be the least desired of the strategies (4).There were inconsistencies in how this was phrased; some participants wanted to be supported by a professional and receive formal support (1, 7, 14, 15, 16, 18, 19, 24, 25) whilst others simply wanted access to formal psychological services or the opportunity to see a counsellor if they felt like they needed it (4, 7, 17, 34, 35, 36, 37). Similarly, employee assistance programs were sought in some studies, which could provide support to second victims outside the workplace (16, 28, 35, 38, 48).

1.2 __Qualitative studies (n=23; papers 50-72)__

Eighteen qualitative studies explored staff needs following an adverse event and what support they believed would be most appropriate in helping second victims recover (50, 51, 52, 53, 54, 56, 70, 71, 57, 58, 59, 62, 63, 64, 65, 66, 67, 68). Of these, thirteen studies reported staff as wanting their organisation‘s approach and support to accommodate them and the incident (51, 53, 54, 56, 70, 71, 58, 59, 62, 63, 66, 67, 68), such as being protected, recognised and avoiding blame or criticism (51, 54, 70, 59). Across the qualitative studies, organisational support also included being freed from workloads, reviewing the staff member's responsibilities (54), allowing tasks and responsibilities to be completed with a colleague (62) and follow-up or long-term support (70), no time limit on recovery (68) recognition that the impact of an event may differ over time (53, 67). Several qualitative/other studies reported that second victims may not seek support even if needed, so organisations and supervisors need to be proactive in reaching out (52, 64). Some staff emphasised a necessity for professional reassurance in emotional recovery (56, 67), with second victims wanting guidance, reassurance and an awareness of management's confidence in them (54, 66).

Staff from twelve qualitative studies desired emotional support and having someone to talk to (54, 70, 57, 66), with eight specifying peers and colleagues as a desired audience (52, 53, 56, 71,

For peer review only - http://bmjopen.bmj.com/site/about/guidelines.xhtml

58, 59, 63, 67, 68) and three looking for managerial support (64, 66, 68). Staff described the ideal supporter as a non-judgemental and empathetic colleague from their direct work environment who could acknowledge and reassure the second victim (70, 58, 67). Structural peer support was suggested as a strategy (52, 63), such as colleagues assisting the second victim with workload or taking time to listen (54), with one study finding that informal peer support was the most desired support method (68).

Eight samples of staff were found to seek formal support, counselling and/or specialist mental health resources (50, 51, 53, 71, 57, 62, 63, 68), which could be accessed by those who had been seriously impacted by an incident (59, 68). Staff across several studies recommended confidential counselling programs provided directly by the organisation (51, 71, 63), although one study reported that there were mixed views about whether this would be accepted within surgical communities (62).

Staff from further six qualitative studies wanted time away from work or a period of leave to help distance themselves from the event and recover (51, 54, 59, 62, 66, 68). Furthermore, six studies found staff seeking practical support in the form of debriefs and discussions (70, 71), administrative support with investigations (56, 57) as well as information, evaluation and learning around the incident (56, 70, 67, 68). Some staff expressed a need to be supported in communicating with the affected patient or family following an incident (62, 68) and be informed of the patient's outcomes (56). Samples from multiple qualitative studies also suggested value in sharing their experiences with other colleagues to share learning around the second victim experience (70, 63, 68).

1.3 Additional information from qualitative studies related to what staff want

- Emotional support following an adverse event was described as the "first measure" following an incident (54) and essential in effectively supporting second victims (54, 57, 59, 66, 67).
- One study emphasised that support should focus on the staff's emotions instead of the cause of the incident (59).
- Many staff across studies reported the need for second victims to feel heard, and that psychosocial support should address the need for them to talk (54, 70, 63, 67).

For peer review only - http://bmjopen.bmj.com/site/about/guidelines.xhtml

- Second victims were reported to need general concern and empathy around wellbeing from colleagues and superiors (71).
- One study's sample suggested the implementation of a mentoring system, giving staff an opportunity to talk with seniors for problem solving (62).
- Across the studies, staff reported wanting access to psychological support and specialist resources (52, 57, 62, 63, 66).
- Some participants wanted administrative and institutional support to help individuals overcome psychological difficulties, and address adverse events and countermeasures (57, 67).
- Multiple participants raised a concern that the culture of healthcare services served as a barrier for blame-free approaches; environments were described as inconsiderate to emotional wellbeing (71) and vulnerability (58) post-incident, and a shift to a caring, gossip-free culture was needed at organisational and individual levels (58, 59). Leadership is needed to promote a safe atmosphere to process patient safety incidents (71), and one study suggested that communicative skills such as empathy should be essential when hiring senior members of staff (58). Another study reported that learning can only take place in a psychologically safe setting (65).
- Although studies noted a variation in the amount of support needed by second victims, recognition of their actions and collegial trust were reported as the minimum level required (56). Investigations into adverse events were found as a potential barrier to recovery, as they could create a sense of anxiety for second victims (56). Therefore, it was important for staff to receive guidance and support for the investigation process and for supervisors to emphasise that the investigations are for system failures, not punishment (56, 67, 68). One study reported a need to discuss working conditions, as this was not always considered during investigations, which was frustrating for the staff involved (67).
- Administrative and practical needs were also identified in various studies. Some staff reported confusion about the post-incident procedure, thereby necessitating organisational guidance on this process (56, 68).
- One study found that the ideal listener differed depending on the culture of the organisation; in 'open culture' organisations, staff preferred talking to internal colleagues, whereas staff in 'blame cultures' preferred an external individual with the same professional background (68).

For peer review only - http://bmjopen.bmj.com/site/about/guidelines.xhtml

A minority of participants from another study suggested that internal peer support may tamper with team relations, and that organisations should have a general, hospital-wide support service, although other participants argued that this was ineffective and a reflection of the hospital culture being insensitive to vulnerability (58). Several studies advocated for peer support being organisation-led instead of relying on individuals to take initiative (51, 71, 67), such as by stimulating intercollegiate conversations around support following an event (58). Follow-up conversations were also suggested by one study in order to account for differing long-term needs (52).

- One study suggested that counselling could be mandatory, as second victims need to address and open up about the event to be competent in the role moving forward (50), with another noting the potential prevalence of PTSD (57).

2. What support do staff receive to cope with Patient Safety Incidents (RQ2)?

2.1 Surveys (n=49; papers 1-49)

Thirty-four survey studies explored the support that staff experienced following an adverse event (1, 2, 3, 5, 6, 8, 10, 11, 12, 13, 16, 17, 20, 21, 22, 23, 24, 28, 30, 31, 33, 35, 36, 38, 39, 40, 41, 42, 43, 45, 46, 47, 48, 49).

Peer support was addressed in twenty-five studies (2, 3, 6, 8, 10, 11, 12, 13, 16, 17, 20, 21, 22, 23, 24, 30, 33, 35, 36, 38, 39, 41, 42, 49) and was rated as both the most experienced (8, 12, 21, 30, 35, 38, 41, 49) and most useful (6, 8, 11, 13, 16, 17, 21) by the majority of studies that ranked supports. Most of the studies that gave figures, reported between 49% and 100% of their samples experiencing peer support (12, 21, 30, 33, 35, 38, 39, 42, 49), although peer support was experienced by less than 1% of samples in two other studies (45, 47). Second victims were found to talk to direct colleagues (1, 2, 3, 21, 22, 23, 24, 35, 39, 41, 49), especially those at a similar rank (13, 20, 41) and those directly involved in the incident (41). Second victims seeking emotional support were primarily looking for peers who could understand the situation in order to manage their own stress, bring relief and prevent burnout (2, 6, 10, 49). Where effectiveness was measured, peer support was largely seen as useful and helpful, including in studies using statistical analyses (2, 6, 8, 17, 20, 35, 36) and quantitative analysis (12), and between 42% and

For peer review only - http://bmjopen.bmj.com/site/about/guidelines.xhtml

94.1% of samples found peer support to be useful. Conversely, one statistical sample reported mixed ratings (33).

Participants also reported experiencing a variety of institutional support strategies. Receiving support from supervisors and department leaders was commonly referenced (6, 13, 24, 30, 33, 35, 42, 43, 45, 49) and was found to be both the second most experienced and second most useful support in studies that ranked strategies (6, 13 24, 42, 43). Managerial support was rated as valuable in several studies (6, 13, 43), whilst others found it to be mixed (33) or insufficient (42, 45, 47). Mortality and Morbidity meetings or patient safety incident discussions were referenced in several studies (2, 17, 28, 43, 49), and were the joint second most commonly experienced support amongst studies that ranked strategies (3, 43, 49), with 67.3% of one sample finding them to be helpful for second victims (28). Debriefs were also experienced by staff (3, 8, 17, 35, 48) and were ranked as the joint second most helpful, with 89.4% to 100% of samples finding these to be useful (17, 35, 36, 48). However, others found the meetings to be unsupportive, neutral or negative to recovery (10, 28, 43). Counselling was also addressed in multiple studies and was reported as useful (11, 17, 20, 35), albeit less experienced than other support options (35, 49).

2.2 <u>Qualitative studies (n=23; papers 50-72)</u>

All twenty-three qualitative studies explored the support that staff received following a patient safety incident. Of the ten that reviewed the support provided, all found it to be insufficient or inconsistent (50, 53, 56, 70, 58, 61, 62, 63, 66, 67). One study reported 82% of its staff as having no support and an unfriendly atmosphere (63), whilst staff in another reported that 65% staff did not have all needs addressed, and that 37% had no support whatsoever (70). Support varied both between and within roles; several studies reported a lack of guidance or a consistent process (53, 69, 56, 70, 58, 61, 67), staff being unaware of the support available (70, 58, 61, 63, 66) and certain staff receiving different levels of support based on their position (52, 58, 64, 68).

Twenty-one studies reported experiences with peer support (50, 51, 52, 53, 55, 69, 56, 70, 71, 57, 58, 59, 60, 62, 72, 63, 64, 65, 66, 67, 68), and it was commonly utilised or seen as helpful in all studies when done in the right way. However there is definitely inconsistency in the way peer support is considered as a

For peer review only - http://bmjopen.bmj.com/site/about/guidelines.xhtml

'support': some studies imply that it is facilitated by the organisation, yet others imply that it is different from organisational support, and simply involves SVs talking to colleagues.

Seven viewed peer support as the most effective form of support or the support that they would typically/first turn to following an event (53, 70, 71, 62, 63, 64, 67). In several studies, the experience of the listening colleague was crucial to the effectiveness of peer support, with the ideal listener being able to fully understand the nature of the job and the incident itself (50, 69, 71, 57, 59, 60, 62, 72, 65, 66, 67, 68). Multiple participants also emphasised that to best support them, the listener should be non-judgemental and refrain from criticising or blaming the second victim (52, 66, 68). As well as providing emotional support (53), many of the healthcare staff members found that peer support could help the second victim with practical advice and reassure them about their capability in the role, which helped them in regaining professional confidence (53, 58, 59, 60, 62, 72, 65, 68). Other studies noted the benefits of peers sharing mistakes of their own (51, 72) and checking in with the second victim generally (51). Support from other second victims was reported as helpful, but only if they were understanding and non-judgemental of the event (68). Conversely, several participants emphasised that peer support had to be conducted in the right way, as colleagues who were too critical or judgmental (53, 59, 60, 65, 68), minimised the event (53, 60, 72, 68) or were overly reassuring (60, 68) could make the second victims feel worse. Attempts to solve the error (72), peers reaching out due to curiosity rather than compassion (68) and small comments that could make second victims feel in the wrong (65) were also seen as barriers to successful peer support.

Twelve qualitative studies addressed managerial support (50, 52, 69, 70, 71, 59, 60, 61, 63, 65, 66, 67), with four finding it to be helpful (52, 71, 59, 66) yet six others finding it to be insufficient (50, 60, 61, 67) or inconsistent (52, 70). In one study, 22% of participants said that informal emotional support from supervisors was the most helpful strategy, whilst 15% found informal emotional support from managers to be best (although 12% also said this was the least helpful support) (70). Examples of effective managerial support included giving staff space without chasing up incident reports (59), celebrating staff in team meetings to facilitate positivity, providing support via team meetings or individual conversations and generally being proactive and accessible after an event (52), which helped to resolve emotional stress (71, 59). Ineffective managerial support led to feelings of isolation (50, 60) and hindered recovery (61, 67)

For peer review only - http://bmjopen.bmj.com/site/about/guidelines.xhtml

Other organisational support methods included debriefs and other discussions around the event, which were referenced by six studies (52, 70, 57, 62, 64, 65). Four of these samples found debriefs to be ineffective due to inconsistencies in their implementation (70, 62, 65), certain staff not being involved and focusing on learning and blaming rather than emotional recovery (52, 65). In one study, 'defusing' was suggested, which was distinctive from formal debriefs and allowed for staff to voice their reactions and prepare for debrief (52). One group of participants referenced Mortality and Morbidity meetings, finding them to be ineffective due to a focus on learning and some staff not being invited (64). Learning was referenced by another six studies (55, 70, 60, 62, 72, 68), with staff finding meaning in discussing the case and evaluating what went wrong (55, 62, 68), teaching others about their experience (60, 68) and developing changes to prevent future incidents (70, 72). Some staff sought help with the administrative and legal aspects of an incident, which was appreciated (68).

Six studies referred to counselling and formal support (50, 55, 56, 70, 63, 68) which was used by staff in five (55, 56, 70, 63, 68). This was seen as helpful in two studies due to it being personalised, objective, empathetic and non-judgmental (56, 68), but seen as the least helpful support by 11% of another sample (70). When these services were not offered by the organisation, participants sought professional help themselves, with 40% of one sample seeking this (63). Taking time away from work helped staff distance themselves from the event in one study (50), but was refused by the organisation in another (54).

2.3 Additional information from qualitative studies related to what staff received

- Certain staff were not invited to Morbidity and Mortality meetings (64) therefore this was seen as an inadequate approach to support all those involved in a PSI.
- In many studies, peer support was seen as helpful for recovery and the emotional wellbeing of the staff involved (51, 52, 53, 55, 69, 56, 70, 71, 58). For some participants, informal peer support helped them feel heard (50, 72). Several studies reported that peer support could be detrimental to the recovery of second victims if done in the wrong way. In one paper, one third of participants experienced issues with their collegial relationships as a result of the failure of peer support, which sometimes led to dispute in the organisation (67). The failure to support the second victim generally was also found to damage working relationships and

For peer review only - http://bmjopen.bmj.com/site/about/guidelines.xhtml

leave the individual feeling unsupported and anxious (56). Multiple studies noted that colleagues 'minimising' the situation did not help, and that it was important for peers to be open and honest about the incident (53, 60, 72). Studies reported that conversations needed to address the coping needs of the physician at the time (60), and that physicians responded best to peers who listened attentively and showed empathy (72). Negative examples of peer support included criticism, blame, being made to feel incompetent (53, 60), attempts to solve the error (72) and peers reaching out due to curiosity, not compassion (68). In one case, small comments were enough to make second victims feel in the wrong (65). Being protected by friends (60) and having colleagues who were resistant to discussion (68) were also barriers to successful peer support.

- In one study, 86% of participants said they would typically turn to a colleague, and 48% received emotional intercollegiate support following a patient safety incident (70), with 69% seeking peer support in another study (71). and receiving empathy from colleagues was seen as beneficial (51, 59).
- Multiple findings emerged concerning the lack of formal structures in place in some institutions, meaning support was based on the individuals around the second victim (70, 61). The absence of structure was attributed as a source of staff isolation by one study (67).
- Some of the inconsistencies described across the support received were related to the positions of the staff involved. Some participants reported feeling unrepresented in the support process; one study found that support for nurses and ODPs was inadequate in comparison to clinicians involved, and that certain staff were not invited to Morbidity and Mortality meetings (64). In one case, healthcare professionals were excluded from investigations about their incidents, which was detrimental to their recovery (68). One study found that clinicians felt unrepresented in their organisation's support service, and that the social science professionals that worked in the service would be unable to understand the clinical context (58). A lack of conversation and discussion about an incident was sometimes reported (67), and the silence served as a barrier to reflection and learning (60). One study found many participants perceiving support as unengaging and minimal, leading to feelings of isolation (50). Another described organisational approaches as 'punitive' and inconsiderate to the systemic factors that contribute to incidents (62). Regarding legality, one study reported that rules and sanctions were barriers to support, and that organisational

For peer review only - http://bmjopen.bmj.com/site/about/guidelines.xhtml

interpretations of these previsions were biased against second victims (61). Conversely, one paper reported staff being supported and taken seriously by the organisation, with the service listening and guiding staff through an event (64).

- Several studies addressed the emotional element involved in support. One study found it to be the most relevant concern following an incident, and that its absence led to isolation for second victims, although it could also be unwanted by certain staff or in certain approaches, such as when it was too soothing or came from someone with minimal experience (68). For some, confidentiality rules made staff reliant on workplace support by preventing them from talking to outsiders, meaning that insufficient workplace support led to feelings of abandonment (65, 67). Emotional support contrasted with Morbidity and Mortality meeting and more practical responses to incidents (72) and was helpful, but organisations sometimes lacked in guidance around it (69) – some staff were unaware of who they could talk to, leading to ineffective support that 85% of one sample was unsatisfied with (63). A lack of emotional support was found to be one of the least helpful responses identified by one study (70).
- Some healthcare staff also turned to managers and supervisors in response to a patient safety incident. In one sample of student nurses, professors were the 'first and primary support' accessed (66), and several studies addressed participants reaching out to managers, supervisors or risk management for support (70, 71). In one study, 22% of participants said that informal emotional support from supervisors was the most helpful strategy, whilst 15% found informal emotional support from managers to be best (although 12% also said this was the least helpful support) (70). Effective support from managers helped to resolve emotional stress (71, 59), facilitate learning from the incident and provide reassurance surrounding competence (60). Several studies reported ineffective managerial support (67), resulting in second victims feeling unsupported and abandoned (50, 60, 66). Proactivity of managers was reported as beneficial in one paper, and included providing support via team meetings, individual conversations, formal peer support and generally being accessible after an event (52). Reactive or passive managers were reported to be too late or non-existent in their support, hindering second victim recovery (52, 61). Other examples of effective managerial support included giving staff space without chasing up incident reports (59) and celebrating staff in team meetings, facilitating trust and a culture of support (52). 'Defusing' was also

For peer review only

suggested, which was distinctive from formal debriefs, allowed for staff to voice their reactions and prepare for debrief (52).

- In some studies, debriefs were suggested as a method for staff to feel less isolated, more supported and help coping generally (64, 65), with incidents being discussed through seniors meetings and interrogations (57). However, the frequency and quality of debriefs varied widely. Debriefs were reported as rare in some studies (62), with only 13% of participants in one study receiving them promptly (70). One study found them to be inconsistent in frequency (sometimes too few, sometimes too many) and participation, with some staff not being invited or deciding not to attend (65). For second victims, this limited the effectiveness of the debriefs and their quality overall, preventing healthcare professionals from receiving guidance. In one case, midwives were found to commonly be excluded from investigations and interprofessional debriefing, with many of them subsequently perceiving investigations as scapegoating and attributing blame (52). Another study noted that debriefs sometimes turned to explicit or implicit blaming of staff for the incident (65), with a focus on learning neglecting the emotional need of second victims. Debriefs were also limited when seniors did not conduct the debriefs (as they only were expected to lead these sessions if they were involved in the incident), forcing healthcare staff to attempt conduction of the debrief themselves (65). 11% of one sample reported debriefs and crisis interventions as the least helpful support (64), and another study noted that any welfare extracted from debriefs is not beneficial to second victims unless later acted upon (64).
- Several studies addressed second victims using their experiences in incidents to facilitate learning and teach others. In one sample, 12% of staff were involved in a learning process, with 11% describing it as the most helpful support (70). Learning was enabled via intercollegiate conversations (60, 72) and analysis of the incident (62, 68), helping to facilitate processing of the incident (72). In one instance, midwives were often excluded from learning opportunities, serving as a barrier to their recovery (52). Some second victims further used their experiences and learning to teach colleagues and prevent future incidents of a similar nature (60, 72, 68). This not only gave second victims an outlet to talk about the experience, but also allowed them to develop system changes (72), and teaching helped facilitate their own recovery (60). Other suggested methods of learning included staff ensuring their skills were sufficient (62), supervision and case discussions (55). Mentoring

For peer review only - http://bmjopen.bmj.com/site/about/guidelines.xhtml

was suggested in one case, but effective mentoring was reported as rare (62). One study noted that a focus on learning (especially during Mortality and Morbidity meetings) was ineffective at supporting staff emotionally (64), with another noting that emotional vulnerability that was not dealt with served as a barrier to learning (65).

- Other methods of support were addressed by several studies, including the use of counselling or professional support. Some organisations reportedly provided staff with professional services, allowing serious impacts to be treated and time to adequately recover (50, 55, 56). When these services were not offered by the organisation, participants sought professional help themselves, with 40% of one sample seeking this (63). Counselling was beneficial for second victims as it was "personalised, objective, empathetic and non-judgemental" (68). Some staff reported that conversations with the patients and families involved in incidents was beneficial for recovery, allowing second victims to address the incident directly, apologise and find closure (60, 62, 72). Other staff sought leave to help distance themselves from the incident and recover (50), although one study reported the hospital refusing this request (54). Some nurses in one sample changed their work routines to facilitate processing of the experience, such as having back-up in case they decide they are unable to complete the shift (55). Having time for group reflection that focused on the emotional impact of an incident was suggested (71), as well as creating an atmosphere of shared responsibility and positivity (59). Some staff sought help with the administrative and legal aspects of an incident (68).
- One paper reported that a lack of support from colleagues and seniors could damage intercollegiate trust and relationships, leaving the second victim isolated and anxious (56).

<u>Reference List: Surveys and qualitative studies included at Stage 1 (n=72)</u>

Study ID	Study Reference	Explored what staff wanted	Explored what staff received

For peer review only - http://bmjopen.bmj.com/site/about/guidelines.xhtml

1	Baas MA, Scheepstra KW, Stramrood CA, Evers R, Dijksman LM, van Pampus MG. Work-related adverse events leaving their mark: a cross-sectional study among Dutch gynecologists. BMC Psychiatry. 2018 Dec;18:1-8.	X	X
2	Bañeras J, Jorge-Pérez P, Bonanad C, Lluva MT, Moll I, Kinori SG. Second victims and quality of support resources among cardiology professionals. Revista Espanola de Cardiologia (English ed.). 2022 Aug;75(8):686-8.		X
3	Berman L, Rialon KL, Mueller CM, Ottosen M, Weintraub A, Coakley B, Brandt ML, Heiss K. Supporting recovery after adverse events: an essential component of surgeon well-being. Journal of Pediatric Surgery. 2021 May 1;56(5):833-8.	X	X
4	Burlison JD, Scott SD, Browne EK, Thompson SG, Hoffman JM. The second victim experience and support tool (SVEST): validation of an organizational resource for assessing second victim effects and the quality of support resources. Journal of Patient Safety. 2017 Jun;13(2):93.	X	
5	Choi EY, Pyo J, Ock M, Lee H. Profiles of second victim symptoms and desired support strategies among Korean nurses: A latent profile analysis. Journal of Advanced Nursing. 2022 Sep;78(9):2872-83.	X	X
6	Draus C, Mianecki TB, Musgrove H, Bastien DJ, Greggs D, Halash C, Lewis A, Mackenzie W. Perceptions of Nurses Who Are Second Victims in a Hospital Setting. Journal of Nursing Care Quality. 2022 Apr 20;37(2):110-6.		X
7	Edrees HH, Paine LA, Feroli ER, Wu AW. Health care workers as second victims of medical errors. Pol Arch Med Wewn. 2011 Apr 1;121(4):101-8.	X	
8	Fatima S, Soria S, Esteban-Cruciani N. Medical errors during training: how do residents cope?: a descriptive study. BMC Medical Education. 2021 Dec;21(1):1-6.	X	X
9	Finney RE, Torbenson VE, Riggan KA, Weaver AL, Long ME, Allyse MA, Rivera-Chiauzzi EY. Second victim experiences of nurses in obstetrics and gynaecology: A second victim experience and support tool survey. Journal of Nursing Management. 2021 May;29(4):642-52	X	
10	Gupta K, Rivadeneira NA, Lisker S, Chahal K, Gross N, Sarkar U. Multispecialty physician online survey reveals that burnout related to adverse event involvement may be mitigated by peer support. Journal of Patient Safety. 2022 Sep 1;18(6):531-8.	X	X
11	Han K, Bohnen JD, Peponis T, Martinez M, Nandan A, Yeh DD, Lee J, Demoya M, Velmahos G, Kaafarani HM. The surgeon as the second victim? Results of the Boston Intraoperative Adverse Events Surgeons' Attitude (BISA)		X

For peer review only - http://bmjopen.bmj.com/site/about/guidelines.xhtml

	study. Journal of the American College of Surgeons. 2017 Jun 1;224(6):1048-56.		
12	Harrison R, Lee H, Sharma A. A survey of the impact of patient adverse events and near misses on anaesthetists in Australia and New Zealand. Anaesthesia and Intensive Care. 2018 Sep;46(5):510-5.	X	X
13	Harrison R, Sharma A, Walton M, Esguerra E, Onobrakpor S, Nghia BT, Chinh ND. Responding to adverse patient safety events in Viet Nam. BMC Health Services Research. 2019 Dec;19:1-8.		X
14	Heard GC, Thomas RD, Sanderson PM. In the aftermath: attitudes of anesthesiologists to supportive strategies after an unexpected intraoperative patient death. Anesthesia & Analgesia. 2016 May 1;122(5):1614-24.	X	
15	Huang R, Sun H, Chen G, Li Y, Wang J. Second-victim experience and support among nurses in mainland China. Journal of Nursing Management. 2022 Jan;30(1):260-7.	X	
16	Jain G, Sharma D, Agarwal P, Agrawal V, Yadav SK, Tenzin T, Alamgir MH, Manandhar K, Myint M, Chaudhary AM, Jami AA. "Second Victim" syndrome among the surgeons from South Asia. Indian Journal of Surgery. 2022 Feb;84(1):40-6.	X	X
17	Joesten L, Cipparrone N, Okuno-Jones S, DuBose ER. Assessing the perceived level of institutional support for the second victim after a patient safety event. Journal of Patient Safety. 2015 Jun 1;11(2):73-8.	X	X
18	Kaur AP, Levinson AT, Monteiro JF, Carino GP. The impact of errors on healthcare professionals in the critical care setting. Journal of Critical Care. 2019 Aug 1;52:16-21.	X	
19	Kerkman T, Dijksman LM, Baas MA, Evers R, van Pampus MG, Stramrood CA. Traumatic experiences and the midwifery profession: a cross-sectional study among dutch midwives. Journal of Midwifery and Women's Health. 2019 Jul;64(4):435-42.	X	
20	Khansa I, Pearson GD. Coping and recovery in surgical residents after adverse events: the second victim phenomenon. Plastic and Reconstructive Surgery Global Open. 2022 Mar;10(3).	X	X
21	Lin JS, Olutoye OO, Samora JB. To Err is human, but what happens when surgeons Err?. Journal of Pediatric Surgery. 2023 Mar 1;58(3):496-502.		X
22	Magaldi M, Perdomo JM, López-Baamonde M, Chanzá M, Sanchez D, Gomar C. Second victim phenomenon in a surgical area: online survey. Revista Española de Anestesiología y Reanimación (English Edition). 2021 Nov 1;68(9):504-12.		X

For peer review only - http://bmjopen.bmj.com/site/about/guidelines.xhtml

23	Martens J, Van Gerven E, Lannoy K, Panella M, Euwema M, Sermeus W, De Hert M, Vanhaecht K. Serious reportable events within the inpatient mental health care: Impact on physicians and nurses. Revista de Calidad Asistencial. 2016 Jul 1;31:26-33.		X
24	Mathebula LC, Filmalter CJ, Jordaan J, Heyns T. Second victim experiences of healthcare providers after adverse events: A cross-sectional study. Health SA Gesondheid (Online). 2022;27:1-6.	X	X
25	McLennan SR, Engel-Glatter S, Meyer AH, Schwappach DL, Scheidegger DH, Elger BS. The impact of medical errors on Swiss anaesthesiologists: a cross-sectional survey. Acta Anaesthesiologica Scandinavica. 2015 Sep;59(8):990-8.	X	
26	Mohamadi-Bolbanabad A, Moradi G, Piroozi B, Safari H, Asadi H, Nasseri K, Mohammadi H, Afkhamzadeh A. The second victims' experience and related factors among medical staff. International Journal of Workplace Health Management. 2019 May 15;12(3):134-45.	X	
27	Mok WQ, Chin GF, Yap SF, Wang W. A cross-sectional survey on nurses' second victim experience and quality of support resources in Singapore. Journal of Nursing Management. 2020 Mar;28(2):286-93.	X	
28	Nijs K, Seys D, Coppens S, Van De Velde M, Vanhaecht K. Second victim support structures in anaesthesia: a cross-sectional survey in Belgian anaesthesiologists. International Journal for Quality in Health Care. 2021 Apr 1;33(2):mzab058.		X
29	O'Meara S, D'Arcy F, Dowling C, Walsh K. The psychological impact of adverse events on urology trainees. Irish Journal of Medical Science (1971-). 2023 Aug;192(4):1819-24	X	
30	Rivera-Chiauzzi E, Finney RE, Riggan KA, Weaver AL, Long ME, Torbenson VE, Allyse MA. Understanding the second victim experience among multidisciplinary providers in obstetrics and gynecology. Journal of Patient Safety. 2022 Mar 1;18(2):e463-9.		X
31	Rodriquez J, Scott SD. When clinicians drop out and start over after adverse events. The Joint Commission Journal on Quality and Patient Safety. 2018 Mar 1;44(3):137-45.	X	X
32	Santana-Domínguez I, González-De La Torre H, Verdú-Soriano J, Berenguer-Pérez M, Suárez-Sánchez JJ, Martín-Martínez A. Feelings of being a second victim among Spanish midwives and obstetricians. Nursing Open. 2022 Sep;9(5):2356-69.	X	
33	Schrøder K, Edrees HH, Christensen RD, Jørgensen JS, Lamont RF, Hvidt NC. Second victims in the labor ward: Are Danish midwives and obstetricians getting the support they need?.		X

For peer review only

	International Journal for Quality in Health Care. 2019 Oct 31;31(8):583-9.		
34	Shuangjiang Z, Huanhuan H, Ling X, Qinghua Z, Mingzhao X. Second victim experience and support desire among nurses working at regional levels in China. Journal of Nursing Management. 2022 Apr;30(3):767-76.	X	
35	Strametz R, Fendel JC, Koch P, Roesner H, Zilezinski M, Bushuven S, Raspe M. Prevalence of Second Victims, Risk Factors, and Support Strategies among German Nurses (SeViD-II Survey). International Journal of Environmental Research and Public Health. 2021 Oct 10;18(20):10594.	X	X
36	Strametz R, Koch P, Vogelgesang A, Burbridge A, Rösner H, Abloescher M, Huf W, Ettl B, Raspe M. Prevalence of second victims, risk factors and support strategies among young German physicians in internal medicine (SeViD-I survey). Journal of Occupational Medicine and Toxicology. 2021 Dec;16(1):1-1.	X	X
37	Sun L, Deng J, Xu J, Ye X. Rumination’s role in second victim nurses’ recovery from psychological trauma: a cross-sectional study in China. Frontiers in Psychology. 2022 May 3;13:860902.	X	
38	Torbenson VE, Riggan KA, Weaver AL, Long ME, Finney RE, Allyse MA, Rivera-Chiauzzi E. Second victim experience among OBGYN trainees: what is their desired form of support? Southern Medical Journal. 2021 Apr;114(4):218.	X	X
39	Turner K, Bolderston H, Thomas K, Greville-Harris M, Withers C, McDougall S. Impact of adverse events on surgeons. British Journal of Surgery. 2022 Apr 2;109(4):308-10.		X
40	Van Gerven E, Bruyneel L, Panella M, Euwema M, Sermeus W, Vanhaecht K. Psychological impact and recovery after involvement in a patient safety incident: a repeated measures analysis. BMJ Open. 2016 Aug 1;6(8):e01140.		X
41	Vanhaecht K, Zeeman G, Schouten L, Bruyneel L, Coeckelberghs E, Panella M, Seys D, Dutch Peer Support Collaborative Research Group. Peer support by interprofessional health care providers in aftermath of patient safety incidents: A cross-sectional study. Journal of Nursing Management. 2021 Oct;29(7):2270-7.	X	X
42	Van Slambrouck L, Verschueren R, Seys D, Bruyneel L, Panella M, Vanhaecht K. Second victims among baccalaureate nursing students in the aftermath of a patient safety incident: An exploratory cross-sectional study. Journal of Professional Nursing. 2021 Jul 1;37(4):765-70.	X	X
43	Vinson AE, Mitchell JD. Assessing levels of support for residents following adverse outcomes: a national survey of		X

For peer review only

	anesthesia residency programs in the United States. Medical Teacher. 2014 Oct 1;36(10):858-66.		
44	Winning AM, Merandi J, Rausch JR, Liao N, Hoffman JM, Burlison JD, Gerhardt CA. Validation of the second victim experience and support tool-revised in the neonatal intensive care unit. Journal of Patient Safety. 2021 Dec 1;17(8):531-40.	X	
45	Wolf M, Smith K, Basu M, Heiss K. The Prevalence of Second Victim Syndrome and Emotional Distress in Pediatric Intensive Care Providers. Journal of Pediatric Intensive Care. 2021 Jul 1;12(02):125-30.	X	X
46	Yan L, Tan J, Chen H, Yao L, Li Y, Zhao Q, Xiao M. Experience and support of Chinese healthcare professionals as second victims of patient safety incidents: A cross-sectional study. Perspectives in Psychiatric Care. 2022 Apr;58(2):733-43.		X
47	Zhang X, Li Q, Guo Y, Lee SY. From organisational support to second victim-related distress: Role of patient safety culture. Journal of Nursing Management. 2019 Nov;27(8):1818-25.	X	X
48	Cutler A, Black JD, Sheth SS, Pathy S. When Things Go Wrong: Examining the Frequency and Aftermath of Critical Incidents Among OBGYN Residents. Connecticut Medicine. 2017 May 1;81(5).	X	X
49	Biggs S, Waggett HB, Shabbir J. Impact of surgical complications on the operating surgeon. Colorectal Disease. 2020 Sep;22(9):1169-74.		X
50	Buhlmann M, Ewens B, Rashidi A. Moving on after critical incidents in health care: A qualitative study of the perspectives and experiences of second victims. Journal of Advanced Nursing. Sep 2022;78(9):2960-2972.	X	X
51	Chang HE, Jang H, Bak YI. Clinical nurses' recovery experiences after adverse events in South Korea: A qualitative study. Collegian. 2022;29(4):456-464.	X	X
52	Christoffersen L, Teigen J, Ronningstad C, Following-up midwives after adverse incidents: How front-line management practices help second victims. Midwifery. Jun 2020;85:102669	X	X
53	De Boer J, van Rikxoort S, Bakker AB, Smit BJ. Critical incidents among intensive care unit nurses and their need for support: explorative interviews. Nursing in Critical Care. Jul 2014;19(4):166-74.	X	X
54	Ferrùs L, Silvestre C, Olivera G, Mira JJ. Qualitative study about the experiences of colleagues of health professionals involved in an adverse event. Journal of Patient Safety. 2021 Jan 1;17(1):36-43.	X	X
55	Ganahl S, Knaus M, Wiesenhuetter I, Klemm V, Jabinger EM, Strametz R. Second Victims in Intensive Care—Emotional		X

For peer review only

	Stress and Traumatization of Intensive Care Nurses in Western Austria after Adverse Events during the Treatment of Patients. International Journal of Environmental Research and Public Health. 2022 Mar 18;19(6):3611.		
56	Kable A, Kelly B, Adams J. Effects of adverse events in health care on acute care nurses in an Australian context: A qualitative study. Nursing and Health Sciences Jun 2018;20(2):238-246	X	X
57	Lee W, Pyo J, Jang SG, Choi JE, Ock M. Experiences and responses of second victims of patient safety incidents in Korea: a qualitative study. BMC Health Serv Res Feb 06 2019;19(1):100.	X	X
58	Leferink E, Bos A, Heringa MP, van Rensen EL, Zwart DL. The need and availability of support systems for physicians involved in a serious adverse event. Journal of Hospital Administration. 2018;7(2):23-30.	X	X
59	Luk LA, Lee FK, Lam CS, So HY, Wong YY, Lui WS. Healthcare professional experiences of clinical incident in Hong Kong: a qualitative study. Risk Management and Healthcare Policy. 2021 Mar 8:947-57.	X	X
60	May N, Plews-Ogan M. The role of talking (and keeping silent) in physician coping with medical error: A Qualitative study. Patient Education and Counseling Sep 2012;88(3):449-454.		X
61	Mokhtari Z, Hosseini MA, Khankeh HR, Fallahi-Khoshknab M, Nasrabadi AN. Barriers to support nurses as second victim of medical errors: A qualitative study. Australasian Medical Journal 2018;11(12):556-560.		X
62	Pinto A, Faiz O, Bicknell C, Vincent C. Surgical complications and their implications for surgeons' well-being. British Journal of Surgery. Dec 2013;100(13):1748-55	X	X
63	Rinaldi C, Leigheb F, Vanhaecht K, Donnarumma C, Panella M. Becoming a "second victim" in health care: Pathway of recovery after adverse event. Revista de Calidad Asistencial. 2016 Jul 1;31:11-9.	X	X
64	Serou N, Husband AK, Forrest SP, Slight RD, Slight SP. Support for Healthcare Professionals After Surgical Patient Safety Incidents: A Qualitative Descriptive Study in 5 Teaching Hospitals. J Patient Saf Aug 01 2021;17(5):335-340.	X	X
65	Schrøder K, Janssens A, Hvidt EA. Adverse events as transitional markers-Using liminality to understand experiences of second victims. Social Science and Medicine. 2021 Jan 1;268:113598.	X	X
66	Tavares APM, Barlem JGT, Silveira RSD, Dalmolin GL, Feijó GDS, Machado IA, Paloski GDR, Abreu IM. Support provided to nursing students in the face of patient safety incidents: a qualitative study. Rev Bras Enferm 2022;75(2):e20220009.	X	X

For peer review only - http://bmjopen.bmj.com/site/about/guidelines.xhtml

67	Ullström S, Sachs MA, Hansson J, Øvretveit J, Brommels M. Suffering in silence: a qualitative study of second victims of adverse events. BMJ Quality & Safety. 2014 Apr 1;23(4):325-31.	X	X
68	Van Gerven E, Deweer D, Scott SD, Panella M, Euwema M, Sermeus W, Vanhaecht K. Personal, situational and organizational aspects that influence the impact of patient safety incidents: a qualitative study. Revista de Calidad Asistencial. 2016 Jul 1;31:34-46.	X	X
69	Huang H, Chen J, Xiao M, Cao S, Zhao Q. Experiences and responses of nursing students as second victims of patient safety incidents in a clinical setting: A mixed-methods study. Journal of Nursing Management. 2020 Sep;28(6):1317-25.		X
70	Kobe C, Blouin S, Moltzan C, Koul R. The second victim phenomenon: perspective of Canadian radiation therapists. Journal of Medical Imaging and Radiation Sciences. 2019 Mar 1;50(1):87-97.	X	X
71	Kruper A, Domeyer-Klenske A, Treat R, Pilarski A, Kaljo K. Secondary traumatic stress in Ob-Gyn: A mixed methods analysis assessing physician impact and needs. Journal of Surgical Education. 2021 May 1;78(3):1024-34.	X	X
70	Kobe C, Blouin S, Moltzan C, Koul R. The second victim phenomenon: perspective of Canadian radiation therapists. Journal of Medical Imaging and Radiation Sciences. 2019 Mar 1;50(1):87-97.	X	X
71	Kruper A, Domeyer-Klenske A, Treat R, Pilarski A, Kaljo K. Secondary traumatic stress in Ob-Gyn: A mixed methods analysis assessing physician impact and needs. Journal of Surgical Education. 2021 May 1;78(3):1024-34.	X	X
72	Plews-Ogan M, May N, Owens J, Ardelt M, Shapiro J, Bell SK. Wisdom in Medicine: What Helps Physicians After a Medical Error? Academic Medicine. Feb 2016;91(2):233-41		X
	Total studies	52	57

For peer review only - http://bmjopen.bmj.com/site/about/guidelines.xhtml

Supplementary 5 File: Description of fourth and fifth (a) most wanted and (b) most frequently experienced forms of support (continued from main article)

(a) Continuation of most wanted forms of support

#4 most desired: Post-incident debrief to clarify and process what happened (n=18 studies):

Debriefs and team discussions with colleagues involved in the incident were wanted by some participants. However, it was not always clear how this was different to peer support, or whether it referred to an informal clinical debriefing or to more structured Critical Incident Stress Debriefing. Fourteen studies found that second victims wanted information about the incident, including an opportunity to explore any ethical issues, causes and preventative measures, and also to be involved in evaluation and learning around the incident which could contribute to post-traumatic growth.

#5 most desired: Supportive and learning-oriented culture (n=14 studies): Healthcare staff in many studies expressed their desire for a responsive organisational approach that supported them after a patient safety incident and a culture in which they felt protected, recognised and not blamed or criticised. In one study, participants ranked the promotion of an open reporting and learning culture as the most critically important form of support to aid recovery and avoid compounding psychological harm after involvement in a patient safety incident. Raising awareness of the impact of patient safety incidents/errors and preparing staff for involvement in such events, through courses and conferences within one's organisation, were also rated as highly desirable in one study.

(b) Continuation of most experienced forms of support

#4 most experienced: Professional mental health support (n=11): Counselling and other professional mental health support was also mentioned in multiple studies. However, this was experienced less often than other support options. In studies where organisations did not offer such services, participants reported seeking this help themselves (e.g. 40% of one sample; **63)**.

For peer review only

There were indications of stigma associated with this option, with almost a third of physicians (31%) in one mixed methods study (17) reporting feeling embarrassed about needing psychological support.

#5 most experienced: The Mortality and Morbidity meeting (n=7): The Mortality and Morbidity (M&M) meeting was referred to in several studies as a form of support after patient safety incidents. These were the joint second most commonly experienced form of support amongst studies that ranked strategies. Some challenges were noted with this meeting as a support approach, such as not all staff involved in an incident may be invited and the focus on logistics and learning may provide little space to discuss the emotional and professional impact.

For peer review only

Supplementary Table: Summary of included studies at Stage 2

First author (reference), country	Intervention name	Intervention description	Study Design (Quality appraisal score)	Sample, Setting	Outcome measure related to effectiveness (measure used)	Outcomes
Johnson [59]<br><br>UK	*No name. 'Resilience coaching intervention to prepare healthcare professionals for stressful healthcare events, particularly adverse events'*	*A psychologist-delivered, Cognitive Behaviour Therapy (CBT)-based, half day workshop (psycho-educational didactic teaching, small group discussion and experiential exercises) followed by a one-hour, individual coaching phone call.*	*Uncontrolled before-after study; mixed methods - semi-structured interviews & questionnaires.*<br><br>*No comparison of users/non-users.*<br><br>*Four data collection points: baseline, after workshop, after coaching call, 4-6 weeks post baseline.*<br>*(32)*	*Midwives, paramedics, paediatric consultants. Trainee obstetrics & gynaecology doctors, trainee paediatric doctors. Physician Associate students, sonography & mammography students (n=66).*<br><br>*Hospital & University.*	*Confidence in coping with adverse events* (3-item, purpose designed measure);<br><br>*Knowledge about resilience, coping strategies, personal strengths* (5-item purpose designed measure T1 & T2 only);<br><br>*Resilience to cope with future adverse events* (Brief Resilience Scale [85]).	*Compared with T1 baseline, statistically significant increases post intervention in (1) Confidence at T2 (unadj. β = 2.43, 95% CI 2.08–2.79, d = 1.55, p < .001), T3 (unadj. β = 2.81, 95% CI 2.42–3.21, d = 1.71, p < .001) and T4 (unadj. β = 2.75, 95% CI 2.31–3.19, d = 1.52, p < .001; (2) Knowledge at T2 (unadj. β = 1.14, 95% CI 0.82–1.46, d = 0.86, p < .001); (3) Resilience at T3 (unadj. β = 2.77, 95% CI 1.82–3.73, d = 0.90, p < .001) and T4 (unadj. β = 2.54, 95% CI 1.45–3.62, d = 0.65, p < .00).*
Mira [60]<br><br>Spain	*Mitigating Impact in Second Victims (MISE) online program*	*A flexible online programme: 'informative' package (information and research about patient safety incidents and second victimhood) and 'demonstrative' package (videos showing what to do/not do after patient safety incident, covering emotional, family, work consequences of adverse events, how to inform patient, access support, support others, how to be prepared/ what to do).*	*Uncontrolled before and after study; quantitative.*<br><br>*Survey/test issued pre and post completion.*<br><br>*No comparison of users/non-users.*<br>*(23)*	*Multi-disciplinary: physicians and nurses (n=292)*<br><br>*Online.*	*Knowledge changes regarding:*<br><br>*(1) Informative Package of the website* (12-item purpose designed test completed pre & post intervention);<br><br>*(2) Demonstrative package of the website* (8-item purpose designed test completed pre & post intervention).	*Significant differences between pre- and post- tests results.*<br><br>*Test 1 Informative package: Pre-intervention (M=6.9, SD =2.0) and post-intervention (M=8.8, SD=1.6; t265=–10.0, P<.001).*<br><br>*Test 2 Demonstrative package: Pre-intervention (M=6.3, SD=1.5) and post-intervention (M=7.2, SD=1.0; t265=–6.2, P<.001).*
Musunur [61]	*When Bad Things Happen: Training*	*A one-hour education session delivered within the curriculum at medical*	*Uncontrolled before-after study; quantitative.*	*Pre-clerkship medical students (n=300).*	*(1) awareness of resources to cope with and report medical error;*	*Overall responses to education session (all 4 domains): significant difference between*

For peer review only

USA	*Medical Students to Anticipate the Aftermath of Medical Errors*	*school (didactic presentation, video vignettes of seniors discussing error experiences, and guided small group discussion).*	*Survey/test issued pre and post education session, across 2 years – 2016-17.*<br><br>*No comparison of users/non-users.*<br>*(18)*	*University.*	*(2) confidence in ability to recognise & cope with medical error;*<br><br>*(3) knowledge of medical error leading to physician burnout;*<br><br>*(4) attitudes to error, including workplace culture, acknowledging responsibility*<br><br>(ALL: 14-item purpose designed pre and post intervention test/survey).	*post- and pre-session results in 2016 (p<0.001; by 0.34 units of mean change, SD=0.35 units) and 2017 (p<0.001, by 0.33 units of mean change, SD=0.03 units).*<br>*Responses by domain: Significant changes in pre and post session results for (1) awareness of resources and (2) confidence in coping with error - in 2016 and 2017. No significant changes in either 2016 or 2017 in pre and post results for (3) knowledge of medical error or (4) attitudes to error.*
Connors [62]<br><br>USA	*RISE program (Resilience in Stressful Events)*	*Peer support programme. Peers, trained in psychological first aid, respond to self- or colleague-referrals within 30 minutes of receiving notification, 24 hours a day, seven days a week. Peers are clinically-based, but tend not to be known to callers, and focus on how the staff member is feeling rather than event details, supporting them to identify coping strategies to aid recovery – usually in person but sometimes by phone.*	*Cross sectional survey; mixed methods – open and fixed response questionnaire.*<br><br>*Compared users/non-users.*<br>*(24)*	*Nurses and nurse leaders (n=412).*<br><br>*Hospital.*	*(1) Professional burnout* (5 items adapted from Emotional Exhaustion domain of Maslach Burnout Inventory [86]);<br><br>*(2) Job satisfaction* (3 items from previously validated questionnaire [87])*;*<br><br>*(3) Personal resilience* (2 of 4 items from Resilience domain in Second Victim Experience & Support Tool (SVEST) [88]).	*Staff Nurses: No significant differences between RISE users/non-users in (2) Job satisfaction (67% vs. 61%; p=0.45). Significant differences in burnout & resilience: (1) Burnout - more RISE users than non-users reported some level of burnout (93% vs 67%, p=0.038). Odds of indicating some level of burnout were 4.3 times greater for recent RISE-users than non-users (p=0.048). (3) Resilience: RISE users (past year) significantly more likely to report resilience (57% vs 36%, p=0.025).*<br>*Nurse Leaders: No significant differences between RISE activators/non-activators in: (1) burnout (40% activators vs 50% non-activators reported at least some burnout; p=0.42) and (2) job satisfaction (75% activators vs. 85% non-activators; p=0.315). Significant differences in (3) Resilience: Non-activators were*

For peer review only

						more resilient than activators (89% vs 50%, p=0.001). Odds of indicating strong resilience were 0.12 times lower for activators than never-activators (p=0.002).
Finney [63]<br><br>USA	*HELP (Healing Emotional Lives of Peers) program*	*Peer support programme. Based on/ informed by the University of Missouri Health Care 'forYOU' model [40]. Provides emotional support to individuals and groups, 24 hours a day, seven days a week. Grounded in Scott's three-tiered interventional model of support. Adaptations: peer supporters trained in all departments & job levels, embedding in every team, not just high-risk areas.*	*Uncontrolled before and after study: quantitative - Full department survey.*<br><br>*No comparison of users/non-users.*<br>*(26)*	*Paediatric healthcare professionals (n=371 [pre-194/373; post-177/403]).*<br><br>*Hospital Paediatric inpatient unit, ICU*	*Impact of programme on Second Victim experience, encompassing: psychological distress; physical distress; colleague support; supervisor support; institutional support; non-work support; professional self-efficacy; absenteeism; turnover intentions* (ALL: SVEST [88]).	*One-year post intervention launch, compared to pre-intervention: Significantly fewer staff agreed institutional support was 'inadequate' (10.9% vs. 0.6%, p<0.001); Significantly fewer reported turnover intentions (18.2% vs 9.2%, p=0.014 (n.b. in table 2 this is cited as p=0.09); No significant differences on any other SVEST dimensions.*
Klatt [64]<br><br>USA	*Supporting Our Staff (SOS) Peer Support Program*	*Peer support programme. Based on/informed by the University of Missouri Health Care 'forYOU' model [40]. Adaptations: (a) peer supporters trained in all departments and job levels, embedding in every team, not just high-risk areas.*	*Uncontrolled before-after study: quantitative – organisational survey.*<br><br>*Planned a baseline survey pre-launch but unable to do so. Issued 3&9 months post-launch.*<br><br>*No comparison of users/non-users: compared SVEST responses of those who reported experiencing a recent adverse event at 3- and 9-months.*<br>*(26)*	*Multi-disciplinary (n=unclear).*<br><br>*All clinical areas, inpatient and outpatient settings, hospital quaternary care campus.*	*Impact of program on Second victim experience, encompassing: psychological distress; physical distress; colleague support; supervisor support; institutional support; non-work support; professional self-efficacy; absenteeism; turnover intentions* (ALL: SVEST [88]).	*3 months post-launch, SVEST issued to 6,109 HCPs; n=1,786 responses. At 9 months post-launch, n=1,188 responses. Authors compared responses of those involved in adverse outcome/error in past 3 years at 3- (n=766) and 9-months (n=549).*<br>*No significant differences identified on any SVEST dimension.*
Moran [65]<br><br>USA	*Resilience in Stressful Events (RISE) program*	*Peer support programme. Peers, trained in psychological first aid, respond to self- or colleague-referrals within*	*Cross sectional survey and Markov modelling to identify cost-benefits of a hospital having versus not having RISE; quantitative.*	*Nurses (n=36).*<br><br>*Hospital.*	*Net monetary benefit & budget impact of having RISE.*<br>*Cost of running RISE program (*divided total financial cost to hospital of program by number of	*Cost of RISE = $656.25 per nurse user.*<br>*After RISE, decrease in turnover & sickness absence per nurse = $23,232.30.*

		30 minutes of receiving notification, 24 hours a day, seven days a week. Peers are clinically-based, but tend not to be known to callers, and focus on how the staff member is feeling rather than event details, supporting them to identify coping strategies to aid recovery – usually in person but sometimes by phone.	*(23)*		nurse users in 2015 (n=80) to get average cost per nurse*). Hospital costs for nurse turnover (*literature review) and *nurse sickness absence after involvement in patient safety incident (*hospital's Human Resources data).<br><br>*Probability of resigning and taking a sick day with/without RISE (*13-item purpose designed survey requesting estimations of outcomes from 2 hypothetical patient safety incident scenarios, e.g. How many days off do you think this nurse would take? How likely is it they would need mental health services or quit?).	*Net monetary benefit saving of having the RISE program after 1 year = $22,576.05 per nurse using RISE.*<br><br>*RISE could save a hospital $1.81 million per year. Having RISE provided a positive net monetary benefit compared to not having RISE in 49,987 of the 50,000 (>99%) Monte Carlo simulations, at a mean net monetary benefit $23,360 (95% confidence interval, $3006 - $161,278).*
Morris [66]<br><br>Australia	*'Always There' peer support program*	*Peer support programme. Based on/ informed by the University of Missouri Health Care 'forYOU' model [40]. Adaptations: (a) peer supporters trained in all departments and job levels, embedding in every team, not just high-risk areas; (b) slightly reconceptualised Tiers 1 and 2: Tier 1 a 'warm response' - for staff experiencing cumulative/ acute stress; Tier 2 a 'hot response' – practical and emotional support for individuals or teams after a traumatic adverse event (e.g., suicide attempt). Part of broader just culture transformation.*	*Uncontrolled before-after study: quantitative – organisation-wide survey.*<br><br>*Compared users/non-users – 2 years post-launch.*<br>*(19)*	*Clinical and non-clinical staff (n=unclear).*<br><br>*ER, inpatient and community settings, large public mental health service.*	*Second victim experiences of those engaging with program, encompassing: psychological distress; physical distress; colleague support; supervisor support; institutional support; non-work support; professional self-efficacy; absenteeism; turnover intentions* (Second Victim Experience and Support Tool [88], used within 'Voices of the Staff' survey).	*Compared survey results pre and post launch of engagers (n=38) and non-engagers (n=232). No significant differences between groups in all but one SVEST dimensions. Significant differences identified between groups in 2 out of 3 items in 'institutional support' dimension (i.e., not for the full dimension): item 18 "my organisation understands the needs of staff in the aftermath of critical incidents" (81.6% of users vs 57.8% of non-users; p=.005) and item 19 "provides more resources to assist with recovery" (78.9% users vs. 59.9% non-users; p=.024).*
Rivera-Chiauzzi [67]	*No name: 'Structured peer support program'.*	*Peer support programme. Based on/ informed by the University of Missouri Health Care 'forYOU'*	*Randomised trial; quantitative – departmental survey.*	*Physicians, Nurses, Physician Assistants (n=42: 23 intervention*	*To develop & evaluate peer support program.*	*<u>Effect on recovery stage:</u>* ***At baseline:*** *Most participants - 28/42 (13/23 enhanced; 15/19 routine) - were at Stage 6*

USA		model [40]. 4 peer supporters attended 'Second Victim train the trainer workshop' by forYOU team. Trained peers then contacted all staff involved in an adverse event within one week, had in-person, one-to-one confidential chat to assess wellbeing, provide information on second victim recovery stages and EAP. Peer supporters phoned staff randomised to 'Enhanced' group one week later, then monthly up to 6 months until no longer needed.	3 timepoints: baseline, 3 months and 6 months post intervention.<br><br>Compared users/non-users.<br>(20)	['enhanced care']; 19 control ['routine care']).<br><br>Obstetric and maternal foetal medicine department, Hospital.	Primary outcome: use of resources & perceptions of helpfulness.<br>Secondary outcome: effect on recovery stage after PSI and duration of support use (ALL: Survey, though unclear if questionnaire was adapted from forYOU toolkit or purpose designed. Included demographics, use of informal resources, perceived helpfulness, barriers, current recovery stage).	'Thriving'; 14/42 were at an earlier recovery stage: Stage 1-3 n=7 (enhanced 6/7; routine 1/7); Stage 4-6 [excluding those 'thriving'] n=7 (enhanced 4/7; routine 3/7). **At 6 months:** 100% of participants, regardless of group, reported that they were 'thriving'.<br><br>**Enhanced group:** Most needed less than 3 months' support. 62.5% declined follow-up after first contact; 91.3% declined follow-up after second contact. Significantly more likely than routine group to consider departmental leadership as one of the most helpful resources (p=0.02).
Thompson [68]	CRNA (Certified registered nurse anesthetists) Peer support Program	Peer support programme. Does not report being based on previously established models. 8 volunteers trained to provide peer support 24 hours a day. Support could be activated through self-, colleague- or lead CRNA-referral, or by the peer supporter noticing a colleague's distress.	Uncontrolled before and after study: quantitative – departmental survey.<br><br>Issued pre- and one-month after trial period to all CRNAs.<br><br>No comparison of users/non-users.<br>(22)	CRNAs. (n=83 [pre-n=51; post-n=32]).<br><br>CRNA Department, large academic medical centre.	Impact of second victim peer support protocol, compared to current post-adverse event hospital practices, on psychological distress; physical distress; colleague support; supervisor support; institutional support; non-work support; professional self-efficacy; absenteeism; turnover intentions (SVEST [88]).	No statistically significant differences were found between SVEST scores pre and post intervention (p>.05).
Wijaya [69]<br><br>Indonesia	No name 'second victim support program'	Peer support programme. Based on/ informed by the University of Missouri Health Care 'forYOU' model [40].	Controlled before and after study: quantitative – hospital-wide survey.<br><br>Issued: pre-launch; up to 1 year post launch; 1-2 years post-launch.<br><br>Compared users/non-users.<br>(17)	Doctors and nurses. (n=unclear. Unsure if exactly the same 87 participants surveyed across phases)<br><br>Hospital.	Perceptions of patient safety culture within the hospital (Hospital Survey on Patient Safety Culture (HSOPSC) [89]).	Phase 1: N=47 (/87; 54% response); Phase 2: N=60 (/same 87; 69% response); Phase 3: N=65 (/same 87; 75% response rate).<br>T1-T2: All HSOPSC ratings increased: Significant increase in patient safety culture (t=4.284, p=0.001, Cohen's d 0.854) large effect size). Compared to control hospital, significant increase in patient

For peer review only

				safety culture within intervention hospital (DID coefficient 0.738, SE= 0.258, p= 0.007). No significant differences at intervention hospital results between T 2 & T3 indicating sustained effect (t=0.378, p=0.713, Cohen's d 0.0476).

For peer review only
